# An integrative genomic and phenomic analysis to investigate the nature of plant species in *Escallonia* (Escalloniaceae)

**DOI:** 10.1038/s41598-021-03419-0

**Published:** 2021-12-14

**Authors:** Sarah J. Jacobs, Michael C. Grundler, Claudia L. Henriquez, Felipe Zapata

**Affiliations:** 1 Department of Ecology and Evolutionary Biology, University of California, Los Angeles, CA 90095 USA; 2 Department of Botany, California Academy of Sciences, San Francisco, CA 94118 USA

**Keywords:** Phylogenetics, Taxonomy

## Abstract

What we mean by species and whether they have any biological reality has been debated since the early days of evolutionary biology. Some biologists even suggest that plant species are created by taxonomists as a subjective, artificial division of nature. However, the nature of plant species has been rarely tested critically with data while ignoring taxonomy. We integrate phenomic and genomic data collected across hundreds of individuals at a continental scale to investigate this question in *Escallonia* (Escalloniaceae), a group of plants which includes 40 taxonomic species (the species proposed by taxonomists). We first show that taxonomic species may be questionable as they match poorly to patterns of phenotypic and genetic variation displayed by individuals collected in nature. We then use explicit statistical methods for species delimitation designed for phenotypic and genomic data, and show that plant species do exist in *Escallonia* as an objective, discrete property of nature independent of taxonomy. We show that such species correspond poorly to current taxonomic species ($< 20\%$) and that phenomic and genomic data seldom delimit congruent entities ($< 20\%$). These discrepancies suggest that evolutionary forces additional to gene flow can maintain the cohesion of species. We propose that phenomic and genomic data analyzed on an equal footing build a broader perspective on the nature of plant species by helping delineate different ‘types of species’. Our results caution studies which take the accuracy of taxonomic species for granted and challenge the notion of plant species without empirical evidence. Note: A version of the complete manuscript in Spanish is available in the Supplemental Materials.

## Introduction

**Figure 1:**
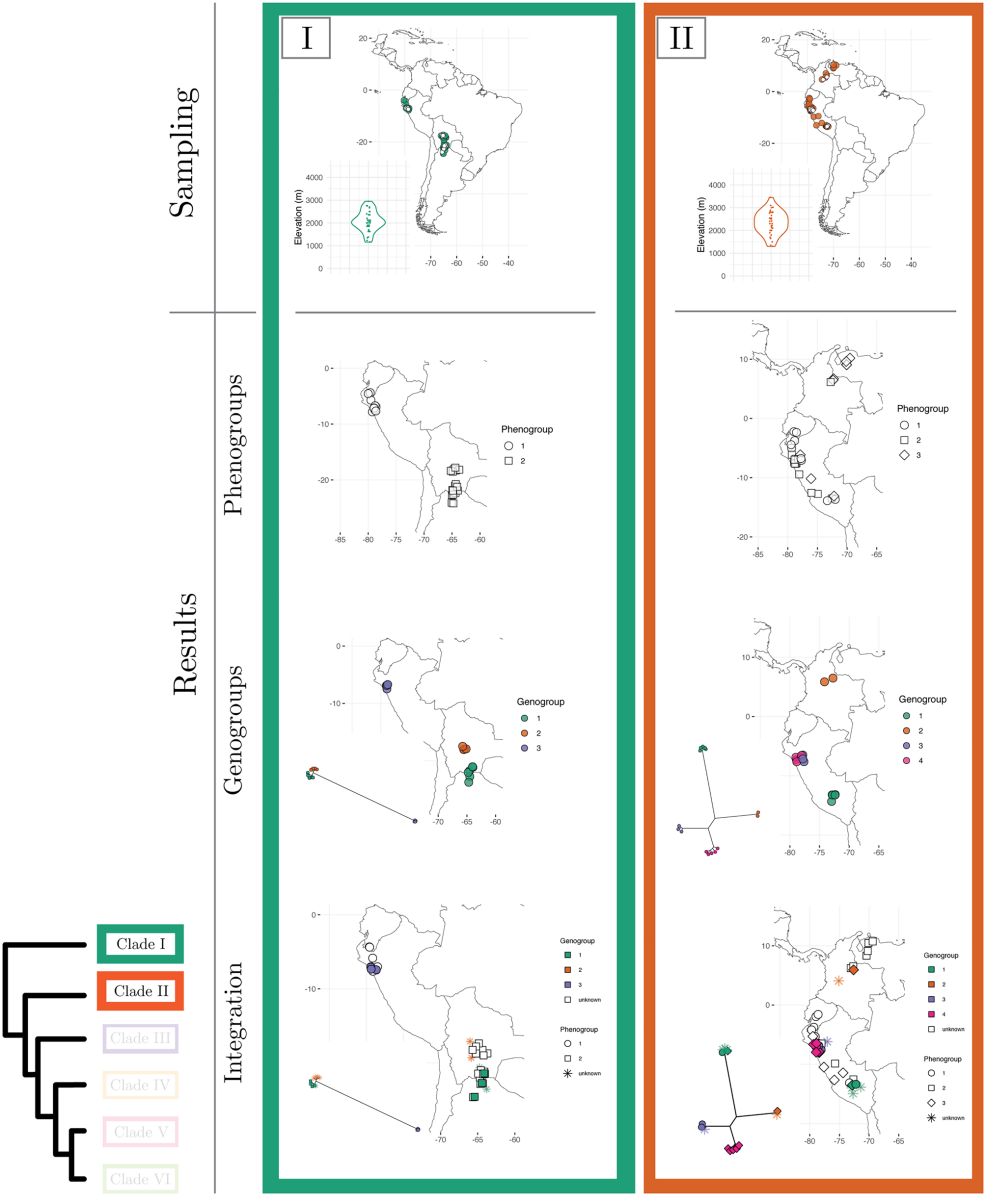

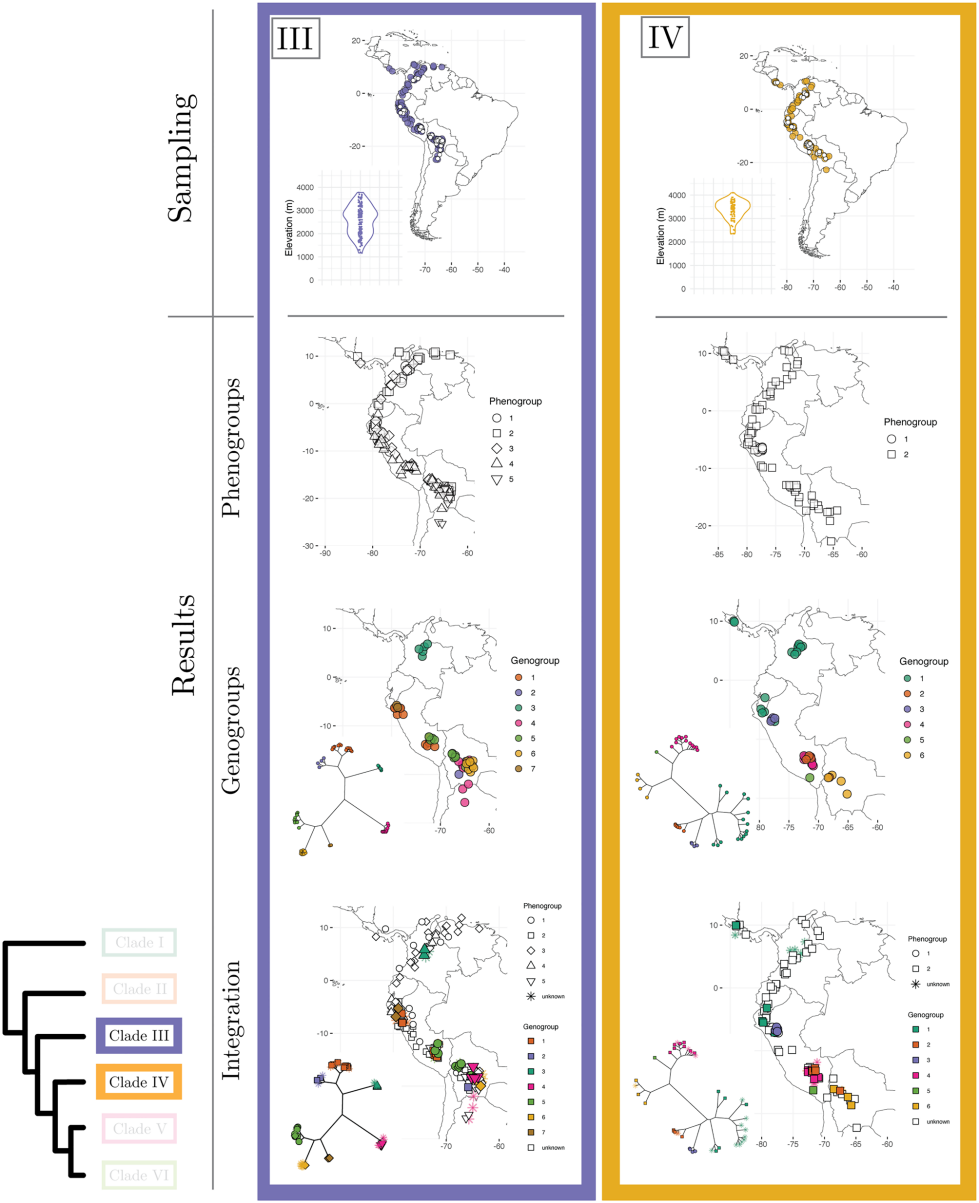

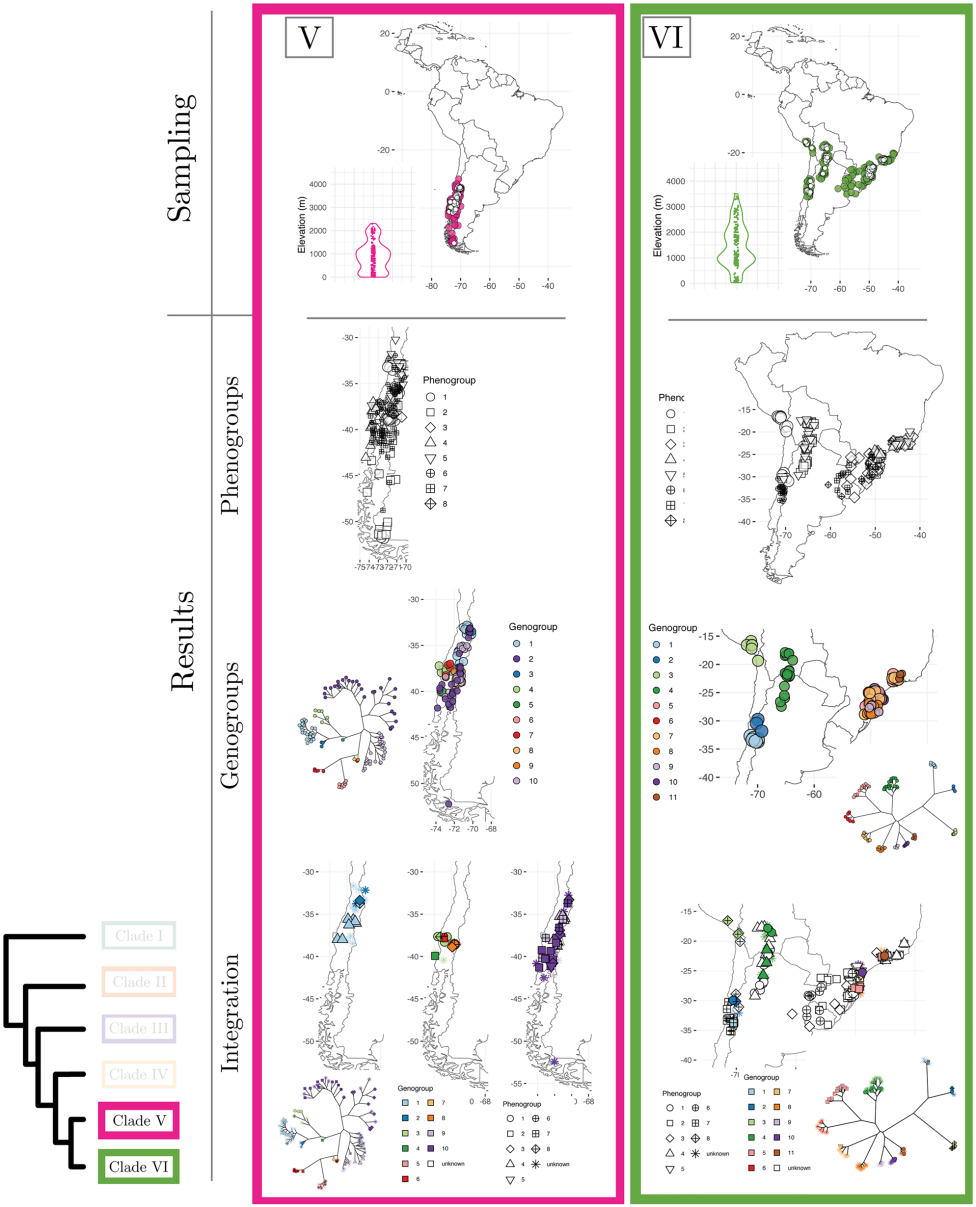
(Presented as three panels) Phylogenetic history, taxon sampling, and evolutionary model-based species delimitation. Maximum Likelihood (ML) tree of *Escallonia* based on genome wide data (bottom-left) with tips indicating the six focal clades (Clade I–VI) of our study. For each clade, the first row shows the taxon sampling, with filled symbols indicating specimens used in phenotypic analyses and empty symbols specimens used in genomic analyses; the insets show the distribution of specimens along elevation. The second row shows results of the best fit model for species delimitation with phenotypic data (i.e., phenogroups); phenogroups are shown with different shapes in geographic space. The third row shows results of the best fit model for species delimitation with genomic data (i.e., genogroups); genogroups are indicated with different colors as tips of unrooted ML trees based on matrices of concatenated loci and mapped in geographic space. The fourth row shows the integration of phenogroups and genogroups with evolutionary history and geographic distribution to elucidate the nature of plant species; specimens without overlapping phenotypic and genomic data are designated as unknown specimens. The phylogenetic trees were inferred in IQ-TREE v2.0.3 (http://www.iqtree.org). The maps were generated in R v4.1.1 using the libraries ggplot2 v3.3.5 (https://ggplot2.tidyverse.org/index.html) and maps v3.4.0 (https://cran.r-project.org/web/packages/maps/).

A perennial question in biology concerns the possibility that plant species are not real, but presumably constructs of the psyche of taxonomists^1–3^. Previous researchers investigating this question through phenotypic data have focused on validating taxonomic species (i.e., the species proposed by taxonomists)^3,4^. This means using taxonomic species as standard references to gauge the strength of the evidence in support of the reality of species when researchers analyze phenotypic data with numerical taxonomy methods to identify species^5^. In a highly influential paper, Rieseberg et al.^3^ compiled data across 400 studies which used numerical methods to identify plant and animal species with phenotypic data, and assessed how well the species delimited with statistical methods matched taxonomic species. This study revealed that validation of taxonomic species is low ($< 60\%$ of statistically identified discrete clusters are congruent with taxonomic species) even though discrete phenotypic groups apparently exist in most taxonomic groups^3^. However, by using a species validation approach, as opposed to a species discovery approach^6,7^, this study assumed that taxonomic species are present. Unfortunately, Rieseberg et al.^3^ did not have access to statistical approaches useful to assess the reality of species independent of taxonomy or to multilocus sequence data as an additional line of evidence to investigate the nature of species across taxa. As a consequence, the fundamental question about the reality of plant species independent of the influence of taxonomists is not well understood. To date, no studies integrating phenotypic and genome-wide DNA data have assessed the reality of plant species for a group including multiple hypothesized taxonomic species at a broad geographic scale. Here we investigate this question through high-density phenotypic (ca. 8300 quantitative measurements) and genome-wide (ca. 1,000,000 DNA sequences) species delimitation analyses of a large data set of 848 individuals in *Escallonia* (Escalloniaceae), a group of shrubs and trees spanning the montane region of South America (Fig. 1, panels 1–3; Supplementary Table S1).

Many studies incorporating the procedure of species delimitation present several shortcomings relevant to understanding the nature of plant species. First, most studies using phenotypic data rely on statistical approaches disconnected from biological theory and hence are compromised in detecting biologically meaningful species^8^. In particular, such studies typically use methods that rely on graphical analyses that convey little information on phenotype frequencies, exclude phenotypic traits potentially important for species detection, and use measures of central tendency which are inconsequential to assess species distinctiveness^8^. Second, many studies use explicit numerical procedures to analyze phenotypic data only when analyzing ‘problematic taxa’ (i.e., species complexes, hybrid swarms), and thus may provide a distorted general perspective on the nature of plant species. Third, some studies do not investigate the nature of plant species directly using genetic data which bear an explicit relationship to evolutionary divergence and gene flow, two relevant criteria in delineating species^9^. Conversely, other studies rely exclusively on genetic data which may fail to uncover species that maintain cohesion and independence via evolutionary forces additional to gene flow^10^. Lastly, several studies do not consider the evidence of species in a geographic context despite the central role of geography in the study of the nature of species^11,12^. We tackle these shortcomings in examining the nature of plant species by integrating multiple types of data and proper statistical approaches well grounded in evolutionary theory in *Escallonia*, a typical genus of flowering plants, seemingly composed of ‘good’ taxonomic species^13^.

Trees and shrubs of the genus *Escallonia* make an excellent case study for carrying out such analyses to investigate the nature of plant species. These plants occur in a variety of habitats throughout the Andes and the mountains of southeastern Brazil, as well as in isolated mountain ranges like the Sierra de Córdoba (Argentina), Sierra Nevada de Santa Marta (Colombia), and Cordillera de Talamanca (Costa Rica)^14,15^. Most taxonomic species have broad geographic ranges, with some species having populations separated by thousands of kilometers; a few narrowly distributed species span less than 200 km. Some taxonomic species are common locally, with approximately 30–40 plants per locality, while others are rare, few individuals being found in any one place (F. Zapata, pers. obs.). Several taxonomic species seem to segregate according to habitat or elevation, nevertheless the geographic ranges of many species overlap completely or partially, such that individuals of one taxonomic species can occur within the range of potential dispersal of gametes (seeds or pollen) of other taxonomic species (i.e., taxonomic species exhibit mosaic sympatry sensu)^16^.

In all taxonomic species, the fruit is a dry capsule that dehisces and releases the seeds, which fall out and are likely dispersed by wind or gravity. Little is known about the pollination biology of any taxonomic species^17^, and from circumstantial observations in the field, the flowers of different taxonomic species of *Escallonia* appear to be visited by a diverse group of local insects that also visit unrelated plant genera. Studies quantifying reproductive isolating barriers across *Escallonia* are necessary to understand the role of floral signals in speciation. Morphologically, the taxonomic species in *Escallonia* show substantial variation in leaf size and overall shape, likely associated with ecological conditions and habitat shifts (F. Zapata, unpublished). Taxonomic species can have either single flowers, or inflorescences with tens to hundreds of flowers. The flowers show considerable geographic variation in the size and shape of sepals, petals, and ovaries. Petal color varies from greenish-white to pink or deep red. Chromosome morphology and number ($\hbox {n} = 12$) are the same for all taxonomic species so far examined^18–20^, and horticulturists have generated artificial hybrids between morphologically distinct species that do not grow together in nature (e.g.^21^). However, there are no documented cases of hybrid speciation or stable hybrid zones in nature.

*Escallonia* thus appears to be a “typical” genus of flowering plants not considered unique or problematic taxonomically. From a genetic perspective, there are no studies using genomic data that include several individuals per taxonomic species for all species across the geographic range of *Escallonia* (i.e., the status of the taxonomic species from a multilocus perspective is not known). It is useful to remember, however, that there is no documented natural rampant hybridization or introgression, there are no known cases of polyploidy, and, to our knowledge, there is no agamospermy or apomixis in the genus. From a morphological perspective, taxonomic species appear to be more or less well defined; some variation exists, but the genus is not notable or unusual in this regard. Taken together, *Escallonia* offers a great opportunity for studying in detail the geographic patterns of variation in phenotypic traits and genomics to examine the nature of plant species.

Elucidating the nature of plant species has broader implications beyond taxonomy. In particular, determining whether species do exist as objective properties of nature can impact other areas of biology which use species as the unit of analysis. Moreover, comparing geographic patterns of variation in phenotypic and genetic data can begin to shed light on the evolutionary forces at work in the origin, evolution, and structuring biodiversity.

## Results and discussion

We present and discuss the major findings below in the context of the whole *Escallonia* radiation. Detailed results are presented in the Supplementary Material.

### The current state of taxonomic species

We first characterized the evolutionary history of *Escallonia* using different phylogenetic approaches with a subset of specimens spanning the geographic range of these plants across South America (Fig. 1, panels 1–3; Supplementary Figs. S1, S2). In all of these analyses, we consistently recover six groups of taxonomic species (hereafter, clades I–VI), in line with a previous study based on fewer loci^14^. All clades are markedly restricted to geographic regions, except clade VI; this clade is mainly restricted to southeastern Brazil, Uruguay, and northeastern Argentina, but includes some species in the Andes (Fig. 1, panels 1–3). A closer examination of the relationship between clade composition and the geographical as well as elevational distributions of clades reveals that when specimens from different clades co-occur in close spatial proximity (e.g., Clades I, II, III, IV in the Tropical Andes), clades are genetically distinct with no intermixing (Fig. 1, panels 1–3; Supplementary Figs. S1, S2). Further, all clades have consistent composition and receive strong statistical support when we use different approaches to phylogenetic analysis (see “Methods” section). However, when we include multiple specimens of the same taxonomic species, several of these specimens are not always each other’s closest relatives within clades (i.e., taxonomic species are either paraphyletic or polyphyletic; Supplementary Fig. S2). This result, along with the marked phylogenetic geographic concordance and consistent composition of clades, suggests that although clades are evolutionarily distinct, the limits of species boundaries within clades would benefit from closer attention^14^. Therefore, we focus our subsequent analyses of phenotypic and genome-wide variation to investigate the nature of species in *Escallonia* on a clade by clade basis.

To investigate the current state of taxonomic species in *Escallonia* through phenotypic data, we first asked whether taxonomic species are quantitatively distinct and then asked whether specimens which are hypothesized to belong to a taxonomic species occupy the morphospace delimited by the combination of traits defining each taxonomic species. For these analyses, we used the morphological characteristics—leaf and floral traits—provided in the taxonomic description of each species^13^. We focused on these traits because taxonomic descriptions include the characters useful in distinguishing all species and in comparing them with other species^22^. We acknowledge that by focusing on these traits alone, we may be excluding traits related to functional species differences (e.g., functional plant traits). However, the traits used in taxonomic descriptions provide a logical starting point to assess the nature of species. It is along such dimensions of the phenotype where taxonomists have previously hypothesized natural breaks and many of these traits (certainly the floral traits) have biological relevance with respect to reproductive function. Additionally, our examination of approximately 3500 herbarium specimens and extensive field work confirm substantial variation in leaf and floral traits across taxonomic species.

We first tabulated the maximum and minimum values of ten quantitative continuous traits provided in each species description (these values are derived from specimens not included in the current dataset). We then used these values as vertices of a 10-cube to represent each species geometrically in phenotypic space and estimated the pairwise overlap among all 10-cubes within clades. This analysis shows that taxonomic species within clades occupy distinct regions of 10-dimensional phenospace with little to no overlap (Table 1, Supplementary Figs. S5, S16, S27, S38, S49, S60). We followed these geometric-based analyses with a matching-prediction analysis whereby we assessed whether each specimen identified to a taxonomic species was inside or outside the 10-cube of its corresponding species based on quantitative measurements of the morphological traits defining the 10-cube (see “Methods” section). Contrary to expectations, these analyses show that the majority ($99.2\%$) of specimens fall outside their respective 10-cube. Furthermore, $98.4\%$ specimens fall outside any 10-cube (Table 1, Supplementary Figs. S5, S16, S27, S38, S49, S60). This means that most specimens had at least one measurement falling outside the range of variation provided in their taxonomic descriptions. The use of fixed ranges for trait values in species descriptions implies that species correspond to geometric shapes with sharp boundaries (e.g., 10-cubes). Given both the statistical and mathematical properties of high-dimensional spaces, once a specimen is beyond the limit imposed by even one dimension of the 10-cube corresponding to its taxonomic species, such specimen immediately falls outside of the whole 10-cube (e.g., the curse of dimensionality)^23,24^. Because most specimens examined here fall outside their respective 10-cube, we suggest that taxonomic species in *Escallonia* may have limited power to capture the multidimensional patterns of phenotypic variation displayed by organisms in nature.

**Table 1:** Current state of taxonomic species.

Clade	Taxonomic species	Specimens	Minimum proportion overlap among 10-cubes	Maximum proportion overlap among 10-cubes	Percent specimens matching any 10-cube	Percent specimens matching correct 10-cube
I	2	33	0	0.00	0.0	0.0
II	2	33	0	0.00	0.0	0.0
III	6	130	0	0.02	1.6	0.8
IV	2	74	0	0.00	0.0	0.0
V	7	214	0	0.13	0.0	0.0
VI	10	195	0	0.00	0.0	0.0

This result is not likely an artifact of the taxonomic monograph^13^ because the original species descriptions cite a large number of examined specimens which cover the known geographic range of all species. The specimens included in our analysis were collected in the same localities where monograph-cited specimens were collected; we even measured some of the herbarium specimens cited in the original species descriptions. Our findings highlight the need of including specimen-level data in taxonomic descriptions and monographs in the future, and using probabilistic approaches that incorporate the variance and covariance among traits to define species in order to capture the shape of species in nature. Although our results are limited to *Escallonia*, we speculate this may be a widespread phenomenon in other groups^25^ because plant species delimited and described with morphology are rarely based on explicit statistical analyses of phenotypic variation grounded on biological theory^26,27^. Therefore, we suggest that investigating the nature of plant species by relying on validating taxonomic species alone can be generally problematic.

### Evolutionary model-based evidence to identify species as objective entities

We used Gaussian finite mixture modeling (GFMM)^28^ within clades to determine both the number of species and the assignment of specimens to species using phenotypic data without prior information about taxonomy. This modeling framework is well-suited for this problem because it implements the evolutionary model underlying the use of quantitative, continuous phenotypic variation in species discovery and delimitation^8,29^. To perform this analysis, we used the same specimens and the same ten diagnostic morphological traits as in our previous analysis (see above). We rotated the original data matrix into orthogonal axes using robust covariance estimators and reduced the dimensionality of the orthogonal axes to only those that optimized the shape, orientation, and the number of phenotypic-based species (hereafter, phenogroups). We identified the best Gaussian Mixture Model—GMM (Naive model) in each clade in a Bayesian information criterion (BIC) and integrated complete-data likelihood (ICL) framework. In addition, we assessed support for alternative models in which we assigned specimens to groups defined a priori, including taxonomic species (Taxonomy model) as well as phenogroups we defined during specimen examination that were independent of taxonomy (Taxonomy Unaware model). The results from these analyses are shown in Fig. 1, panels 1–3, and Table 2. The Naive model was the best-supported model for five of the six clades ($\Delta \hbox {BIC}>8$), while one clade had support ($\Delta \hbox {BIC}<1$) even though the model was not the best supported for this clade (Supplementary Fig. S39). These results were insensitive to model-selection approach (BIC or ICL) (see Supplementary Material). The strong performance of the Naive model is not unexpected owing to the severe limitations of the competing, non-statistical approaches to delimit species without considering the shape, orientation, and arbitrary overlap of phenogroups in multidimensional phenotypic space^8^ (Supplementary Figs. S6, S17, S28, S39, S50, S61). This is also consistent with the prediction that nature is, in fact, discontinuous^30,31^ despite suggestions that species are not discrete objective entities^2^. Furthermore, because the majority of the identified phenogroups within clades co-occur locally in sympatry (Fig. 1, panels 1–3, Supplementary Figs. S6, S17, S28, S39, S50, S61), species status for these groups is granted under a wide range of species definitions^8,9,16,32^. Yet, phenogroups may conceal distinct species when similar phenotypes have evolved (or are evolving) independently^33^. Thus, incorporating phylogenetic information is beneficial in understanding the nature of species and deciding whether all phenogroups are distinct species.

**Table 2:** Gaussian finite mixture modeling (GFMM) for phenogroup delimitation and model selection using the Bayesian information criterion (BIC).

Clade	Model	Phenogroups	BIC	Rank	$\Delta$BIC
I	Naive	2	54.03099	1	0.00000
	Taxonomy	2	45.80586	2	8.22513
	Taxonomy unaware	1	33.45654	3	20.57445
II	Naive	3	71.72976	1	0.00000
	Taxonomy unaware	1	47.52785	2	24.20191
	Taxonomy	2	17.77346	3	53.95630
III	Naive	5	387.15280	1	0.00000
	Taxonomy unaware	4	170.83930	2	216.31350
	Taxonomy	6	53.38527	3	333.76753
IV	Taxonomy	2	${-}$ 115.00390	1	0.00000
	Taxonomy unaware	2	${-}$ 115.00390	1	0.00000
	Naive	3	${-}$ 115.89910	2	0.89520
V	Naive	8	${-}$ 516.72340	1	0.00000
	Taxonomy unaware	4	${-}$ 648.03900	2	131.31560
	Taxonomy	7	${-}$ 791.45350	3	274.73010
VI	Naive	8	231.24780	1	0.00000
	Taxonomy unaware	10	200.30380	2	30.94400
	Taxonomy	10	${-}$ 517.76350	3	749.01130

In order to identify species and assign specimens to species within clades using genetic data, we evaluated the fit of three common species delimitation models. These models implement three different species definitions, namely species defined as genotypic clusters^34,35^ (GC model), species defined as the transition point from cladogenesis to anagenesis^36,37^ (CA model), and species defined as reproductively isolated lineages^11,38^ (RI model). We note that these species definitions are not linked to any particular speciation mechanism. For instance, under different ecological or geographic speciation mechanisms species could be diagnosed as the transition from cladogenesis to anagenesis, or as isolated genetic pools. Our analysis is not an inference of the speciation process itself. Rather, our study is a search for patterns (i.e., species), which we then interpret in light of plausible speciation scenarios (see section below). For this analysis, we collected genome-wide data for a subset of the specimens used in our phenotypic analyses and compared competing species delimitation models in a Bayesian framework using Bayes factors^39^ to identify genomic-based species (hereafter, genogroups). Because neither taxonomic species nor any other *a priori* groups have been proposed based on genetic data, we did not assess support for any other alternative species delimitation models. Figure 1, panels 1–3, and Table 3 show the results of these analyses. In general, the CA model outperformed the alternative models; in five of six clades, the CA model was the best-supported model, while the GC model fit better for only one clade. Further, the CA model adequately captures the species we discovered here (Table S2). Across clades, the best fitting model identified the largest number of genogroups. The reason why the models with more genogroups fit better in all clades is likely the result of the higher genetic variation between genogroups than within genogroups, apparent as long branches in the species trees (Fig. 1, panels 1–3). This suggests that genogroups are divergent lineages on separate evolutionary trajectories, and is consistent with the hypothesis that such lineages are distinct species^7,9^. Moreover, several of these genogroups within clades co-occur locally in sympatry, and thus species status for such groups is granted under multiple species definitions^11,16,32^. However, in some clades genogroups form isolated, allopatric groups of specimens, which could presumably result from sparse geographic sampling within a single species^40^. Therefore, the weight of the evidence in support of the species status for these genogroups is weak and requires considering other lines of evidence on an equal footing.

**Table 3:** Genomic modeling for genogroup delimitation and model selection using Bayes factors (BF).

Clade	Model	Genogroups	Marginal Likelihood ($log_e$)	Rank	BF (2 x $log_e$)
I	GC	3	− 6580.495	1	
	AC	2	− 6754.495	2	348.000
	RI	2	− 6754.495	2	348.000
II	AC	4	− 13460.917	1	
	GC	3	− 15036.438	2	3151.042
	RI$^{\mathrm{a}}$	3	− 15036.438	2	3151.042
	RI$^{\mathrm{b}}$	2	− 18963.342	3	11004.850
III	AC	7	− 8985.782	1	
	RI$^{\mathrm{a}}$	5	− 10014.260	2	2056.955
	RI$^{\mathrm{b}}$	3	− 12233.131	3	6494.698
	GC	3	− 12233.131	3	6494.698
IV	AC	6	− 9601.514	1	
	GC	3	− 11546.649	2	3890.271
	RI$^{\mathrm{a}}$	2	− 12017.878	3	4832.728
	RI$^{\mathrm{b}}$	2	− 12017.878	3	4832.728
V	AC	10	− 4588.693	1	
	GC	6	− 5381.361	2	1585.336
	RI$^{\mathrm{a}}$	3	− 5601.058	3	2024.730
	RI$^{\mathrm{b}}$	2	− 6085.998	4	2994.610
VI	AC	11	− 2921.024	1	
	GC	7	− 3627.806	2	1413.564
	RI$^{\mathrm{a}}$	4	− 4661.351	3	3480.654
	RI$^{\mathrm{b}}$	4	− 4661.351	3	3480.654

$^{\mathrm{a}}$Specimens assigned to demes using MAVERICK.

$^{\mathrm{b}}$Specimens assigned to demes using STRUCTURE.

### Integrating phenotypic and genome-wide variation, spatial information, and evolutionary history

**Figure 2:**
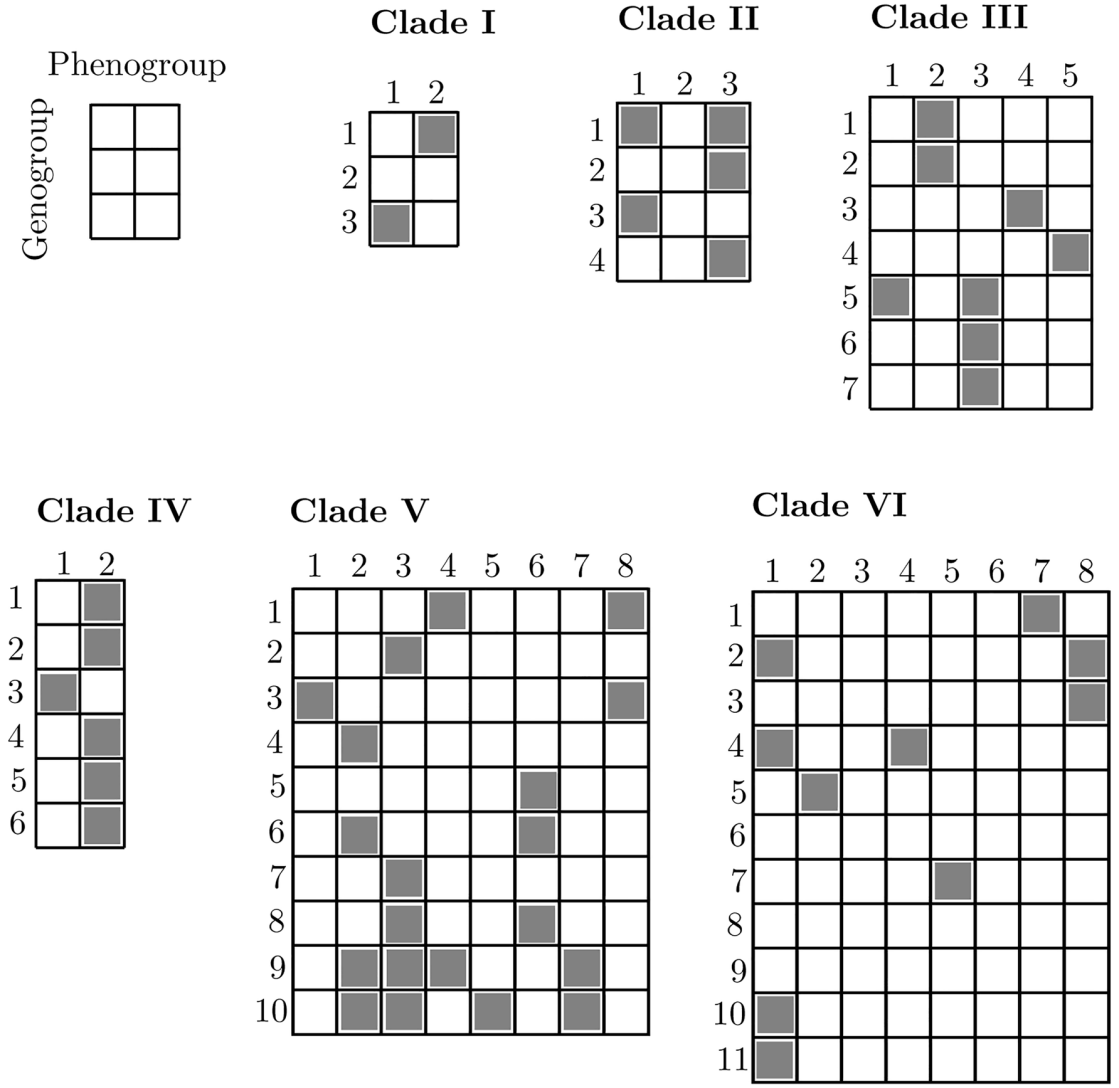
Integration of phenotypic and genome-wide variation to delimit species. For each clade (see panels of Fig. 1), we assigned specimens to their corresponding phenogroup and genogroup based on the best fit models for each type of data. Shaded cells show specimens assigned to a particular combination of best fit phenogroup and genogroup (i.e., each shaded cell is a species). Three types of species are recognized. First, specimens assigned uniquely to a single phenogroup and a single genogroup are recognized as ‘good species’ (e.g., phenogroup 4, genogroup 3 in Clade III). Second, specimens assigned to a single phenogroup across multiple genogroups are recognized as ‘phenotypic cryptic species’ (e.g., phenogroup 2, genogroups 1, 2 in Clade III). Third, specimens assigned to a single genogroup across multiple phenogroups are recognized as ‘genetic cryptic species’ (e.g., phenogroups 1, 3, genogroup 5, in Clade III). Empty rows or columns correspond to specimens which did not have overlapping phenotypic and genomic data and thus were assigned only to their corresponding phenogroup or genogroup, accordingly (e.g., genogroup 2 in Clade I).

With the phenogroups and genogroups derived from the evolutionary model-based analyses, we were able to examine the nature of species by integrating phenotypic and genome-wide data in an explicit spatial and evolutionary context (Fig. 1, panels 1–3; Supplementary Figs. S13, S24, S35, S46, S57, S68). For this analysis, we first assigned each specimen to its corresponding phenogroup and genogroup, akin to a two-way contingency table (Fig. 2). This assignment allowed the identification of congruence—or lack thereof—between phenotypic and genomic groups. Some specimens were incomplete (e.g., sterile) and could not be scored for all phenotypic traits, while other specimens failed during processing for genomic work (hereafter, unknown specimens); nevertheless, the geographic distribution of these unknown specimens in relation to the specimens with both kinds of data may inform the most parsimonious pheno- or genogroup assignment (for example, in Clade IV all the unknown specimens from northern South America likely belong to phenogroup 2 and genogroup 1; Fig. 1, panel 2). Overall, we found that only a small percentage of phenogroups correspond directly to unique genogroups ($15\%$), even assuming concordant group assignment for all unknown specimens ($18\%$). By contrast, we found that in most clades a given phenogroup occurs across multiple genogroups (for example, see phenogroup 2 in clade IV, Fig. 2), and less frequently that a given genogroup occurs across different phenogroups (for example, see genogroup 9 in clade V, Fig. 2). Taken together, our results suggest that the proportion of ‘good species’ (i.e., phenotypic and genomic distinct and congruent groups) in *Escallonia* is remarkably low, particularly given the widespread notion in biology that ‘good species’ are the norm, and suggest that other types of species, including ‘phenotypic cryptic species’^33^ (i.e., one phenogroup across multiple genogroups) and ‘genetic cryptic species’^10^ (i.e., one genogroup across multiple phenogroups), are more common. The existence of these different types of species is consistent with the idea that the properties of species, such as morphological distinguishability or genealogical exclusivity of alleles, may evolve at different times and sequential order owing to the heterogeneous nature of the speciation process^41,42^.

Interpreting the species that we identified in an explicit spatial and phylogenetic context can further elucidate the nature of plant species. Our motivation is to provide an interpretation of the type of species we uncovered (pattern) in light of plausible speciation mechanisms (process). We note, however, that further work with denser sampling and suitable analytical approaches is critical to infer the actual speciation process. Most ‘good species’ co-occur in local sympatry or segregate according to elevation with other species (Figs. 1, panels 1–3, 2, Supplementary Figs. S13, S24, S35, S46, S57, S68). This suggests that environmentally-mediated selection in sympatry or along elevational gradients in parapatry may be an important evolutionary force driving speciation^43^ or at least maintaining species differences in *Escallonia*. While these species can differ in floral and leaf traits, studies about reproductive biology and the role of other biotic and abiotic factors are needed to unravel how ‘good species’ in *Escallonia* originate and are maintained in nature. Alternatively, it is possible that these species are further along the speciation continuum and have accumulated enough differences^44,45^. Further sampling in combination with phylogenetic dating approaches and experimental data in *Escallonia* are needed to evaluate these hypotheses with increasing rigor.

When the genogroups of ‘phenotypic cryptic species’ are distantly related, a reasonable hypothesis to explain this pattern is the idea of convergent evolution in phenotypes in response to similar selective regimes, either in sympatry or allopatry^46^ (for example, see phenogroup 1, genogroups 2, 4, 10, 11, clade VI; Fig. 1, panel 3). *Escallonia* occurs in mountain habitats which show similar environmental conditions across separate geographic regions (e.g., the mountains of southeastern Brazil, the southern Andes, and the high elevation Tropical Andes)^14^. The possibility of replicated evolution of species with similar leaf and floral traits across separate geographic regions as a mountain archipelago is intriguing and should be investigated in detail. By contrast, when such genogroups are each other’s closest relatives and do not co-occur locally in sympatry (for example, see phenogroup 2, genogroups 1, 2, clade III; Fig. 1, panel 2), under some species definitions genogroups may correspond to allopatric populations within a single species^11^ rather than to distinct species resulting from recent speciation with little time for phenotypic differentiation, or speciation with niche conservatism^46,47^. Exhaustive geographic sampling is necessary before these hypotheses can be confronted confidently and the nature of these species in *Escallonia* is better understood.

In all the ‘genetic cryptic species’ that we identified, phenogroups do not show a strong geographic structure (for example, see genogroup 10, phenogroups 2, 3, 5, 7, clade V; Fig. 1, panel 3). This is consistent with the intriguing possibility that these otherwise phenotypically distinct species could potentially be interconnected via gene interchange^48,49^, likely facilitated by their broad overlap in geographical space^14^. Whether this pattern reflects speciation with gene flow or gene flow after secondary contact remains unknown. Our current sampling in *Escallonia* is not designed to untangle these possibilities and further analyses are required. However, we note that genomic evidence for this type of species is rapidly accumulating for other plants^50–52^ as well as various taxa across the tree of life^10,53^. In other taxonomic groups these type of species include both recently diverged species, which plausibly differentiate in the face of gene flow, as well as species with over 10–20 million years of divergence with subsequent gene flow occurring after secondary contact^54,55^. Yet, how these groups of species are initiated and persist, and what portion of their genomes is exchanged freely across species boundaries without species collapse needs to be studied in closer detail^56^. Furthermore, we argue that the discovery approach we employ here, where both phenotype and genotype contribute equally and independently to the pattern of species, is essential to detecting these types of species groups where they are otherwise unexpected. *Escallonia* makes an excellent case study for tackling these critical questions, yet additional genomic, phenomic, and geographic sampling are needed.

Alternatively, these ‘genetic cryptic species’ may be the result of rapid divergence events driven by strong factors influencing traits relevant for ecological isolation with little time for alleles to sort completely between sister species^57^. Because several phenogroups within a genogroup sometimes co-occur in mosaic sympatry^16^ or replace each other along elevation^14^ (Supplementary Results), it is plausible that rapid divergence in *Escallonia* has been prompted by new ecological opportunities owing to climatic cycles and mountain orogeny^58^. The lack of experimental studies about the functional ecology of leaf and floral traits in *Escallonia* precludes us from knowing what factors are responsible for maintaining the phenotypic divergence displayed by different phenogroups within a single genogroup. Some phenogroups may differ in floral traits which might bear a relationship with pollinators. Other phenogroups may vary more strongly in leaf traits which might relate to adaptation to local environments. Hence, it is plausible that different forms of selection maintain phenotypic differences and counteract the homogenizing effects of gene flow in nascent species, a possibility that requires further research. Further taxon and genome sampling in combination with explicit population genomic models that incorporate different forms of selection are thus required in *Escallonia* to isolate the signal of incomplete lineage sorting from hybridization^59^ and model the role of selection between sister species and non-sister species in secondary contact.

## Conclusion

In sum, our analyses of a large scale phenotypic and genome-wide dataset using state of the art model-based approaches for species discovery and delimitation reveal that plant species do exist in *Escallonia* as a property of nature independent of taxonomy^7,31^. However, the observed pattern of excessive discordance between species identified with phenotypic and genomic data suggests that in the absence of evidence the prevalent assumption that phenotypically (or genetically) distinct entities are necessarily ‘good species’ is not warranted. Furthermore, parallel signatures of such discordance across divergent clades in *Escallonia* suggest that this may be a widespread phenomenon, which is consistent with the emerging patterns about the nature of species across the tree of life^10,33,51–54^. The species discovery approach we use here, which explicitly considers both phenotypic and genetic data on an equal footing, is essential to revealing patterns useful to guide our inference of likely evolutionary processes at work in speciation. Previous studies have proposed that approximately 70% of plant taxonomic species represent ‘good species’^3^, but this is not supported in our study. Instead, our results suggest that the percentage of taxonomic species in *Escallonia* which correspond to ‘good species’ may be as low as 17% (Table 4, Supplementary Tables S4, S7, S10, S13, S16, S19). Because *Escallonia* appears to be a “typical” genus of flowering plants not considered unique or problematic taxonomically (see Introduction), this result is notable. We are not aware of datasets of similar magnitude for other plant groups, yet we speculate that our results may be widespread. To the extent that our findings capture any generalizable perspective about the nature of plant species, reinforced by the overall poor theoretical basis underlying plant species delimitation^26,27^, our results suggest that studies in other areas of biology which assume taxonomic species represent good, biologically real entities may need critical evaluation. Our results underscore the need for further comparative studies combining high-throughput phenotypic and genotypic data across taxa and across broad and narrow spatial scales to comprehensively understand the nature of plant species and shed light into the evolutionary forces at work in speciation and in maintaining species in nature^7^. Given the unprecedented advances in phenomics, genomics, and computation, there has never been a more thriving time to be a taxonomist than now.

**Table 4:** Correspondence between taxonomic species and best-fit phenogroups and genogroups.

Clade	Taxonomic species	Phenogroups	Perfect match taxonomic species to phenogroups	Genogroups	Perfect match taxonomic species to genogroups	Perfect match taxonomic species to phenogroup and genogroup
I	2	2	2	3	1	1
II	2	3	0	4	1	0
III	6	5	1	7	3	1
IV	2	2	2	6	1	1
V	7	8	0	10	0	0
VI	10	8	2	11	5	2

## Methods

### Taxon sampling and data collection

This study complies with local and national regulations. Collecting permits were obtained for field collections. A total of 848 specimens were included in this study (a mix of field collections and herbarium specimens). These specimens covered the entire geographic range of *Escallonia.* To assign specimens to taxonomic species, one of us (Felipe Zapata) identified all plant material using the dichotomous key provided by Sleumer^13^ as well as information on habit, habitat, geographic locality, and the available comparative material from ca. 3, 500 herbarium collections. *Escallonia* currently includes 40 taxonomic species^13,60^; the specimens included in this study belong to 29 taxonomic species. Complete voucher information for all specimens is available in Table S1. On these specimens, we measured 10 quantitative, continuous phenotypic traits (leaf length, leaf width, pedicel length, ovary length, length of calyx tube, length of calyx lobes, petal length, petal width, filament length, style length) to characterize the geographic pattern of phenotypic variation across *Escallonia*. We focused on these traits because these are the traits used in the taxonomic monograph to describe and distinguish all species^13^. All measurements were log-transformed prior to downstream analysis.

To examine the geographic pattern of genomic variation across *Escallonia*, we used double-digest Restriction-Site Associated DNA Sequencing (ddRAD)^61^ for 315 specimens (out of the 848 specimens). We first extracted DNA from silica-dried adult leaves or herbarium specimens and then prepared quadruple-indexed, triple-enzyme RADseq libraries using the *EcoRI*, *XbaI*, and *NheI* restriction enzymes^62^. All libraries were sequenced across multiple lanes of 100PE sequencing on the Illumina HiSeq 4000 Sequencing Platform. We assembled RAD loci and called variants using iPyrad v0.7.28 (https://ipyrad.readthedocs.io/en/master/)^63^, and filtered files for downstream analyses using VCFtools v0.1.14 (https://vcftools.github.io)^64^ and custom-made scripts. To assess the sensitivity of our results to the amount of missing data, we ran analyses using three matrices with different levels of missing data (25%, 50%, and 75% missing data). Detailed descriptions on sampling and data collection are provided in the Supplementary Material.

### The current state of *Escallonia* taxonomic species

We used a subset of specimens to reconstruct the phylogeny of *Escallonia*. We chose these specimens to represent the overall spectrum of morphological variation and the geographic range of each taxonomic species. We used *Valdivia gayana* as outgroup^14^. We built phylogenies with two and four specimens per taxonomic species using the three data matrices with different amounts of missing data. For each dataset, we inferred lineage trees using a matrix of concatenated full loci in IQ-TREE v2.0.3 (http://www.iqtree.org) and the edge-proportional partition model with 1000 ultrafast bootstrap replicates^65–68^. To infer species trees, we used SVDQuartets^69^ in PAUP* v4.0a168 (https://paup.phylosolutions.com)^70^ by evaluating all possible quartets. Confidence on species trees was assessed with a multilocus bootstrap analysis using 100 replicates. Both the lineage and species tree reconstructions across all subsets consistently recovered six well-supported clades (see “Results and discussion” section; clades I–VI). We conducted all downstream analyses within clades considering only ingroup samples.

To examine the state of taxonomic species through phenotypic data, we used the most recent taxonomic monograph of *Escallonia* to tabulate the minimum and maximum values reported for ten quantitative traits used to describe and delimit each taxonomic species^13^. The combination of these values predicts a hypervolume in phenotypic space occupied by each taxonomic species. Therefore, we used these values as vertices to construct a hypervolume (i.e., a 10-cube) to represent geometrically each species in 10 phenotypic dimensions. To determine the distinctiveness of each taxonomic species, we estimated the pairwise asymmetric proportion of overlap of all 10-cubes within clades. To assess whether the specimens that we measured in this study matched the prediction specified by the taxonomic description of each species (i.e., whether specimens were inside the space defined by the hypervolume in phenotypic space), we used the morphological measurements to ask whether specimens assigned to a taxonomic species were inside or outside the 10-cube of their corresponding taxonomic species. We used this approach because taxonomic descriptions include all the characters useful in distinguishing species and in comparing them with other species in multidimensional phenospace^22^. Therefore, our approach provides a reasonable assessment of the range of variation present in nature predicted to be partitioned by each taxonomic species. We refer to this analysis as ‘matching-prediction analysis’. We did not include meristic or qualitative traits in this analysis because we focused on the same traits that we analyzed using explicit methods for species discovery and delimitation with phenotypic data, which are grounded on evolutionary theory (see below). *Escallonia* currently includes 40 taxonomic species^13,60^; the specimens included in this study belong to 29 taxonomic species. We used the R packages grDevices^71^ and geometry v0.4.5^72^ to carry out these analyses. Further details are provided in the Supplementary Material.

### Model-based evidence for species using phenotypic data

To determine the number of phenotypic-based species (hereafter, phenogroups) and the assignment of specimens to phenogroups within clades, we applied the quantitative genetics model for the distribution of continuous quantitative traits within a species^29^. This model states that under the assumption of polygenic architecture for phenotypic traits and random mating, gene frequencies would be close to Hardy–Weinberg equilibrium and phenotypic variation among individuals of a single species would tend to be normally distributed^73^. While we do not know the genetic architecture of any of the traits included in our study, analyses in other plants show that some of these traits are indeed polygenic^74,75^. We assume that a similar genetic architecture is present in *Escallonia*, and therefore that the pattern of variation of such traits can be reasonably described with Gaussian distributions. We applied this Fisherian model employing Gaussian Finite Mixture Modeling (GFMM) to search for the mixture of normal distributions (i.e., phenogroups) that best explains the variation in the data^28^. GFMM is a powerful framework to model the phenotypic variation of species seen in nature because it can combine normal distributions of different shapes and orientations^8^. To define the phenotypic space for GFMM, we first used robust principal components analysis (rPCA)—an approach useful for high dimensional data when outliers could skew the orientation of the rotated axes markedly^76^—on our ten, log-transformed, quantitative traits. We then used automatic variable selection^77,78^ to select the most useful set of robust PC axes for GFMM using forward variable selection and no variable transformation. Lastly, we fitted different Gaussian Mixture Models (GMM) specifying 1 to $n + n/2$ number of phenogroups, where *n* is equal to the number of taxonomic species currently hypothesized to exist within each clade. This approach aimed to limit the number of phenogroups present in the mixture to a reasonable number informed by current taxonomy and minimize over-differentiation of phenogroups. We evaluated three competing models for phenogroup delimitation:

#### Naive model

The optimal GMM was determined without a priori assignment of specimens to phenogroups.

#### Taxonomy model

The GMM used specimens assigned a priori to taxonomic species (see above).

#### Taxonomy unaware model

The GMM used specimens assigned *a priori* to groups based on a comparative, non-explicit analysis of phenotypic variation (i.e., phenogroups were determined by eye).

##### Model selection

To determine the best fit model—including the number, orientation, and shape of phenogroups in the mixture as well as the assignment of specimens to phenogroups—, we used the Bayesian information criterion (BIC)^79^ and the integrated complete-data likelihood (ICL) criterion^80^. We used the R packages pcaPP v1.9-73^81^ and mclust v5.4.6^82^ to perform these analyses. Further details are provided in the Supplementary Material.

### Model-based evidence for species using genomic data

Because our sensitivity analyses were robust to the amount of missing data (see Supplementary Material), we performed the following analyses using the matrix with the lowest amount of missing data (25% missing data) for computational efficiency. To determine the number of genomic-based species (hereafter, genogroups) and the assignment of specimens to genogroups within clades, we evaluated three competing models for genogroup delimitation. In all analyses, we did not assign specimens to genogroups a priori.

#### GC model (genotypic clusters model)

This model is in essence the operational equivalent with genetic data of the Fisherian model described above. It states that the presence of two or more genotypic clusters in a sample of individuals provides evidence for more than one species because distinct genetic clusters are recognized by a deficit of intermediates, both at single and multiple loci^34^. To delimit these genogroups, we employed GFMM in genotypic space^35^. Using the matrix with a single nucleotide polymorphism (SNP) per locus, we first estimated the shared allele distance^83^, defined as one minus the proportion of alleles shared by 2 individuals averaged over loci. Loci with missing data were not considered in the pairwise distance calculation. To define the genotypic space for GFMM, we followed Huasdorf and Hennig^35^ and used non-metric multidimensional scaling (NMDS) to reduce the dimensionality. In all clades, we retained only two dimensions (stress $<15\%$). In this space, we fitted different GMM specifying 1 to $n + n/2$ number of phenogroups, where *n* is equal to the number of taxonomic species currently hypothesized to exist within each clade. To determine the best GMM, we used the Bayesian Information Criterion (BIC). We used the R package prabclus v2.3-2^84^ to carry out these analyses.

#### CA model (cladogenesis to anagenesis model)

This model states that species reside at the transition point between evolutionary relationships that are best represented cladogenetically (i.e., between-species branching events) and relationships that are best reflected anagenetically (i.e., within-species branching events)^36^. To delimit these genogroups, we applied an explicit phylogenetic model to identify significant changes in the pace of branching events on a phylogeny^37^. Under the assumption that the number of substitutions between species is significantly higher than the number of substitutions within species, these differences are reflected by branch lengths that represent the mean expected number of substitutions per site between two branching events (cladogenesis and anagenesis). We applied this model within clades employing multi-rate Poisson tree process modeling in the mPTP software v0.2.4 (https://github.com/Pas-Kapli/mptp)^37^. We used the concatenated matrix with complete sequences for all loci and generated a phylogenetic tree per clade using IQ-TREE v2.0.3 (http://www.iqtree.org) with ultrafast bootstrap approximation to assess branch support^66,67^. Because mPTP requires a rooted phylogeny, we mid-point rooted each phylogeny using the R package phytools v0.6-99^85^. We ran mPTP under both a maximum likelihood and a Bayesian framework with a minimum branch length threshold of 0.0001 for all analyses. For Bayesian runs, we used default priors and generated 500 million samples (i.e., independent delimitations), sampling every 1 million steps and ignoring the first 1 million as burn-in. We summarized all runs to indicate the percentage of delimitations in which a node was identified as a cladogenesis event (nodes with values closer to 1) or a transition to anagenesis (nodes with values closer to 0).

#### RI model (reproductive isolation model)

This model states that species are evolutionarily independent groups of individuals which do not exchange genes^11^. To delimit these genogroups, we used an explicit population genetic framework^86^ which, under the assumption of extremely limited to absent gene flow after speciation, models the evolution of gene trees within species and identifies groups of individuals in gene trees that are shared across loci^87^. We applied this model within clades employing a Bayesian modeling framework using the software BPP v4.0 (https://github.com/bpp/bpp)^88^ in the analysis mode A11^89^. Because BPP requires that specimens are assigned a priori to ‘genetic populations’ (i.e., demes), we used the matrix with one SNP per locus and employed model-based clustering for this initial step. This clustering approach uses multilocus genotypes to find demes that (as far as possible) are in Hardy–Weinberg or linkage equilibrium. We applied this model-based clustering approach in a Bayesian framework using the programs STRUCTURE v2.3.4 (https://web.stanford.edu/group/pritchardlab/structure.html)^90^ and rMaverick v1.0.5 (https://github.com/bobverity/rmaverick)^91^, which uses thermodynamic integration instead of the heuristic estimators used in STRUCTURE. For both analyses, we fitted different models specifying 1 to $n + n/2$ number of demes, where *n* is equal to the number of taxonomic species currently hypothesized to exist within each clade. To determine proper exploration across different species delimitation models, we used both algorithms (0 and 1) implemented in BPP^87^ and compared the results across replicated runs. For each run, we used a random starting tree and a chain with at least 500,000 steps, sampling every 10 step and discarding the first 1000 samples as burn-in. Further details are provided in the Supplementary Material.

##### Model selection

To determine the best fit model for genogroup delimitation—including the number of genogroups and the assignment of specimens to genogroups—, we used Bayes factor delimitation (*with genomic data; BFD*)^92^. For this analysis, we used an explicit population genetic model to compute the likelihood of a species tree directly from the SNP datasets, which bypasses the sampling of the gene trees at each locus^93^. To properly compare candidate species delimitation models, we applied the scaling of the marginal likelihood proposed by Leaché et al.^92^. We applied this framework employing the Bayesian Markov chain Monte Carlo (MCMC) sampler SNAPP v1.4.1 (https://www.beast2.org/snapp/)^93^, which we ran through the software BEAST v2.5.2 (http://www.beast2.org)^94^. BFD* uses path sampling to estimate the marginal likelihood of the species delimitation models^92^. We conducted path sampling with 24 steps, using an MCMC with 250,000 steps, sampling every 10 steps, and ignoring the first 12, 500 steps as burn-in. If each of the 24 steps achieved an effective sample sizes (ESS) $\geqslant 100$, we inferred convergence; otherwise, we ran a second path sampling with 24 more steps using an MCMC with 500,000 steps and 25,000 steps as burn-in. We compared competing models and chose the best model fit for genogroup delimitation using Bayes factors according to the framework provided by Kass and Raftery^95^. A Bayes factor (BF) statistic (2$\times$ $log_e$) > 10 provides decisive evidence favoring the highest ranked model. These analyses were followed by a model adequacy analysis using a goodness-of-fit approach to determine whether the genogroups we delineated could be generated by the best-fit model. To carry out these analyses, we ran BEAST v2.5.2 on the CIPRES Science Gateway v3.3.^96^. Further details are provided in the Supplementary Material.

### Integrating phenotypic and genome-wide variation, spatial information, and evolutionary history

Based on the best fit models for phenogroup and genogroup delimitation, we assigned all specimens to their corresponding phenogroup and genogroup. Because each specimen was necessarily assigned to a single phenogroup and a single genogroup, we determined three types of species according to the possible combinations of phenogroup and genogroup assignment. First, specimens assigned to a single phenogroup and a single genogroup delineated species that we determined as ‘good species’. Second, specimens assigned to a single phenogroup across multiple genogroups delineated species that we determined as ‘phenotypic cryptic species’. Third, specimens assigned to a single genogroup across multiple phenogroups delineated species that we determined as ‘genetic cryptic species’. Several specimens did not have overlapping phenotypic and genomic data (e.g., old herbarium specimens for which only phenotypic data were available, sterile specimens for which only genomic data were available). Therefore, we assigned these specimens only to their corresponding phenogroup or genogroup, accordingly. We referred to these specimens as ‘unknown specimens’. To interpret the different types of species and the ‘unknown specimens’ in an evolutionary context, we mapped the phenogroup and genogroup assignments onto the tips of the phylogenies inferred in the CA model analysis (see above). Similarly, we interpreted the different types of species and the ‘unknown specimens’ in a spatial context using the geolocation data available for each specimen. Both the evolutionary and spatial contexts provided insight into the nature of plant species by illustrating patterns of common ancestry and patterns of sympatry/allopatry across geography and elevation.

### Correspondence between taxonomic species and model-based species

To compare the taxonomic species with the species we delimited based on phenotypic and genomic data, we assigned all specimens to their corresponding taxonomic species, and to their best fit phenogroup and genogroup. Because each specimen was necessarily assigned to a single taxonomic species, phenogroup, and genogroup, we counted the number of ‘perfect matches’. A perfect match is defined as a symmetrical match between a unique taxonomic species and a unique phenogroup, genogroup, or combination of phenogroup and genogroup. For instance, specimens assigned to species *x* and uniquely to phenogroup *a* as well as assigned uniquely to phenogroup *a* and species *x* represent a perfect match. This assessment enabled us to determine the number of taxonomic species that represent ‘good species’.

## Supplementary Information

Supplementary Information.

## Data Availability

Raw FASTQ reads for this study have been deposited in the SRA under Bioproject accession number PRJNA760914. All other data, including raw morphological measurements and intermediate files are available in a public repository at: https://github.com/zapata-lab/ms_nature_of_species.

## Code availability

Code repository is available at: https://github.com/zapata-lab/ms_nature_of_species.

## References

[1] Lewis H (1959). The nature of plant species. J. Ariz. Acad. Sci..

[2] Levin DA (1979). The nature of plant species. Science.

[3] Rieseberg LH, Wood TE, Baack EJ (2006). The nature of plant species. Nature.

[4] Mayr E (1992). A local flora and the biological species concept. Am. J. Bot..

[5] Sneath PH, Sokal RR (1973). Numerical Taxonomy. The Principles and Practice of Numerical Classification.

[6] Carstens BC, Pelletier TA, Reid NM, Satler JD (2013). How to fail at species delimitation. Mol. Ecol..

[7] Barraclough TG (2019). The Evolutionary Biology of Species.

[8] Cadena CD, Zapata F, Jiménez I (2018). Issues and perspectives in species delimitation using phenotypic data: Atlantean evolution in Darwin’s finches. Syst. Biol..

[9] de Queiroz K, Harrison RG, Berlocher SH (1998). The general lineage concept of species, species criteria, and the process. Endless Forms: Species and Speciation.

[10] Cadena CD, Zapata F (2021). The genomic revolution and species delimitation in birds (and other organisms): Why phenotypes should not be overlooked. Auk.

[11] Mayr E (1970). Populations, Species, and Evolution: An Abridgment of Animal Species and Evolution.

[12] Levin DA (2000). The Origin, Expansion, and Demise of Plant Species.

[13] Sleumer, H. O. Die Gattung Escallonia. In *Verhandelingen der Koninklijke Nederlandse Akademie van Wetenschappen, Afd. Natuurkunde*, 1–149 (1968).

[14] Zapata F (2013). A multilocus phylogenetic analysis of *Escallonia* (Escalloniaceae): Diversification in montane South America. Am. J. Bot..

[15] Sede SM, Dürnhöfer SI, Morello S, Zapata F (2013). Phylogenetics of *Escallonia* (Escalloniaceae) based on plastid DNA sequence data. Bot. J. Linn. Soc..

[16] Mallet J (2008). Hybridization, ecological races and the nature of species: Empirical evidence for the ease of speciation. Philos. Trans. R. Soc. B Biol. Sci..

[17] Valdivia CE, Niemeyer HM (2006). Do floral syndromes predict specialisation in plant pollination systems? Assessment of diurnal and nocturnal pollination of *Escallonia myrtoidea*. NZ J. Bot..

[18] Zielinski QB (1955). Escallonia: The genus and its chromosomes. Bot. Gaz..

[19] Sanders RW, Stuessy TF, Rodriguez R (1983). Chromosome numbers from the flora of the Juan Fernandez islands. Am. J. Bot..

[20] Hanson L, Brown RL, Boyd A, Johnson MA, Bennett MD (2003). First nuclear DNA c-values for 28 angiosperm genera. Ann. Bot..

[21] Eastwood A (1929). The Escallonias in Golden Gate Park, San Francisco, California: With descriptions of new species. Calif. Acad. Sci..

[22] Winston JE (1999). Describing Species: Practical Taxonomic Procedure for Biologists.

[23] Bellman R (1958). Dynamic programming and stochastic control processes. Inf. Control.

[24] Hastie T, Tibshirani R, Friedman J (2009). The Elements of Statistical Learning: Data Mining, Inference, and Prediction.

[25] Pineda YM, Cortes AJ, Madrinan S, Jimenez I (2020). The nature of espeletia species. BioRxiv..

[26] McDade LA (1995). Species concepts and problems in practice: Insight from botanical monographs. Syst. Bot..

[27] Stevens PF (2000). Botanical systematics 1950–2000: Change, progress, or both?. Taxon.

[28] McLachlan GJ, Peel D (2004). Finite Mixture Models.

[29] Fisher RA (1919). The correlation between relatives on the supposition of mendelian inheritance. Earth Environ. Sci. Trans. R. Soc. Edinb..

[30] Dobzhansky T (1937). Genetics and the Origin of Species.

[31] Barraclough TG, Humphreys AM (2015). The evolutionary reality of species and higher taxa in plants: A survey of post-modern opinion and evidence. New Phytol..

[32] Coyne JA, Orr HA (2004). Speciation.

[33] Fišer C, Robinson CT, Malard F (2018). Cryptic species as a window into the paradigm shift of the species concept. Mol. Ecol..

[34] Mallet J (1995). A species definition for the modern synthesis. Trends Ecol. Evol..

[35] Hausdorf B, Hennig C (2010). Species delimitation using dominant and codominant multilocus markers. Syst. Biol..

[36] Baum DA, Shaw KL, Hoch PC (1995). Genealogical perspectives on the species problem. Experimental and Molecular Approaches to Plant Biosystematics.

[37] Kapli P (2017). Multi-rate Poisson tree processes for single-locus species delimitation under maximum likelihood and Markov chain Monte Carlo. Bioinformatics.

[38] Yang Z, Rannala B (2010). Bayesian species delimitation using multilocus sequence data. Proc. Natl. Acad. Sci..

[39] Leaché AD, Fujita MK, Minin VN, Bouckaert RR (2014). Species delimitation using genome-wide SNP data. Syst. Biol..

[40] Mason NA, Fletcher NK, Gill BA, Funk WC, Zamudio KR (2020). Coalescent-based species delimitation is sensitive to geographic sampling and isolation by distance. Syst. Biodivers..

[41] Baum DA (1998). Individuality and the existence of species through time. Syst. Biol..

[42] De Queiroz K (2007). Species concepts and species delimitation. Syst. Biol..

[43] Filatov DA, Osborne OG, Papadopulos AS (2016). Demographic history of speciation in a senecio altitudinal hybrid zone on Mt. Etna. Mol. Ecol..

[44] Weir JT, Price TD (2011). Limits to speciation inferred from times to secondary sympatry and ages of hybridizing species along a latitudinal gradient. Am. Nat..

[45] Singhal S, Moritz C (2013). Reproductive isolation between phylogeographic lineages scales with divergence. Proc. R. Soc. B Biol. Sci..

[46] Struck TH (2018). Finding evolutionary processes hidden in cryptic species. Trends Ecol. Evol..

[47] Wiens JJ (2004). Speciation and ecology revisited: Phylogenetic niche conservatism and the origin of species. Evolution.

[48] Lotsy J (1925). Species or linneon. Genetica.

[49] Cronk QC, Suarez-Gonzalez A (2018). The role of interspecific hybridization in adaptive potential at range margins. Mol. Ecol..

[50] Novikova PY (2016). Sequencing of the genus Arabidopsis identifies a complex history of nonbifurcating speciation and abundant trans-specific polymorphism. Nat. Genet..

[51] Cannon CH, Petit RJ (2020). The oak syngameon: More than the sum of its parts. New Phytol..

[52] Wang X, He Z, Shi S, Wu C-I (2020). Genes and speciation: Is it time to abandon the biological species concept?. Natl. Sci. Rev..

[53] Mallet J, Besansky N, Hahn MW (2016). How reticulated are species?. BioEssays.

[54] Barth JM (2020). Stable species boundaries despite ten million years of hybridization in tropical eels. Nat. Commun..

[55] Hipp AL (2020). Genomic landscape of the global oak phylogeny. New Phytol..

[56] Harrison RG, Larson EL (2014). Hybridization, introgression, and the nature of species boundaries. J. Hered..

[57] Rundell RJ, Price TD (2009). Adaptive radiation, nonadaptive radiation, ecological speciation and nonecological speciation. Trends Ecol. Evol..

[58] Nevado B, Contreras-Ortiz N, Hughes C, Filatov DA (2018). Pleistocene glacial cycles drive isolation, gene flow and speciation in the high-elevation andes. New Phytol..

[59] Edelman NB (2019). Genomic architecture and introgression shape a butterfly radiation. Science.

[60] Zapata F, Villarroel D (2019). A new species of *Escallonia* (Escalloniaceae) from the inter-Andean tropical dry forests of Bolivia. PeerJ.

[61] Peterson BK, Weber JN, Kay EH, Fisher HS, Hoekstra HE (2012). Double digest RADseq: An inexpensive method for de novo SNP discovery and genotyping in model and non-model species. PLoS ONE.

[62] Bayona-Vásquez NJ (2019). Adapterama III: Quadruple-indexed, double/triple-enzyme RADseq libraries (2RAD/3RAD). PeerJ.

[63] Eaton DA, Overcast I (2020). Ipyrad: Interactive assembly and analysis of RADseq datasets. Bioinformatics.

[64] Danecek P (2011). The variant call format and VCFtools. Bioinformatics.

[65] Minh BQ, Hahn MW, Lanfear R (2020). New methods to calculate concordance factors for phylogenomic datasets. Mol. Biol. Evol..

[66] Hoang DT, Chernomor O, Von Haeseler A, Minh BQ, Vinh LS (2018). UFBoot2: Improving the ultrafast bootstrap approximation. Mol. Biol. Evol..

[67] Minh BQ (2020). IQ-TREE 2: New models and efficient methods for phylogenetic inference in the genomic era. Mol. Biol. Evol..

[68] Kalyaanamoorthy S, Minh BQ, Wong TK, von Haeseler A, Jermiin LS (2017). ModelFinder: Fast model selection for accurate phylogenetic estimates. Nat. Methods.

[69] Chifman J, Kubatko L (2014). Quartet inference from SNP data under the coalescent model. Bioinformatics.

[70] Swofford, D. L. *PAUP*: Phylogenetic Analysis Using Parsimony (and Other Methods) Version 4.0 beta* (2003).

[71] R Core Team. *R: A Language and Environment for Statistical Computing*. (R Foundation for Statistical Computing, 2020).

[72] Habel, K., Grasman, R., Gramacy, R. B., Mozharovskyi, P. & Sterratt, D. C. *Geometry: Mesh Generation and Surface Tessellation* (2019).

[73] Templeton AR (2006). Population Genetics and Microevolutionary Theory.

[74] Chitwood DH (2013). A quantitative genetic basis for leaf morphology in a set of precisely defined tomato introgression lines. Plant Cell.

[75] Qian M (2021). Genome-wide association study and transcriptome comparison reveal novel QTL and candidate genes that control petal size in rapeseed. J. Exp. Bot..

[76] Croux C, Filzmoser P, Oliveira MR (2007). Algorithms for projection-pursuit robust principal component analysis. Chemom. Intell. Lab. Syst..

[77] Raftery AE, Dean N (2006). Variable selection for model-based clustering. J. Am. Stat. Assoc..

[78] Maugis C, Celeux G, Martin-Magniette M-L (2009). Variable selection in model-based clustering: A general variable role modeling. Comput. Stat. Data Anal..

[79] Fraley C, Raftery AE (1998). How many clusters? Which clustering method? Answers via model-based cluster analysis. Comput. J..

[80] Biernacki C, Celeux G, Govaert G (2000). Assessing a mixture model for clustering with the integrated completed likelihood. IEEE Trans. Pattern Anal. Mach. Intell..

[81] Filzmoser, P., Fritz, H. & Kalcher, K. *pcaPP: Robust PCA by Projection Pursuit* (2018).

[82] Scrucca L, Fop M, Murphy TB, Raftery AE (2016). mclust 5: Clustering, classification and density estimation using Gaussian finite mixture models. R J..

[83] Bowcock AM (1994). High resolution of human evolutionary trees with polymorphic microsatellites. Nature.

[84] Hennig, C. & Hausdorf, B. *Prabclus: Functions for Clustering of Presence-Absence, Abundance and Multilocus Genetic Data* (2019).

[85] Revell LJ (2012). Phytools: An r package for phylogenetic comparative biology (and other things). Methods Ecol. Evol..

[86] Rannala B, Yang Z (2003). Bayes estimation of species divergence times and ancestral population sizes using DNA sequences from multiple loci. Genetics.

[87] Yang Z, Rannala B, Edwards SV (2010). Bayesian species delimitation using multilocus sequence data. Proc. Natl. Acad. Sci..

[88] Flouri T, Jiao X, Rannala B, Yang Z (2018). Species tree inference with BPP using genomic sequences and the multispecies coalescent. Mol. Biol. Evol..

[89] Yang Z, Rannala B (2014). Unguided species delimitation using DNA sequence data from multiple loci. Mol. Biol. Evol..

[90] Pritchard JK, Stephens M, Donnelly P (2000). Inference of population structure using multilocus genotype data. Genetics.

[91] Verity R, Nichols RA (2016). Estimating the number of subpopulations (k) in structured populations. Genetics.

[92] Leache AD, Fujita MK, Minin VN, Bouckaert RR (2014). Species delimitation using genome-wide SNP data. Syst. Biol..

[93] Bryant D, Bouckaert R, Felsenstein J, Rosenberg NA, RoyChoudhury A (2012). Inferring species trees directly from biallelic genetic markers: Bypassing gene trees in a full coalescent analysis. Mol. Biol. Evol..

[94] Bouckaert R (2014). BEAST 2: A software platform for bayesian evolutionary analysis. PLoS Comput. Biol..

[95] Kass RE, Raftery AE (1995). Bayes factors. J. Am. Stat. Assoc..

[96] Miller, M. A., Pfeiffer, W. & Schwartz, T. Creating the CIPRES science gateway for inference of large phylogenetic trees. In *2010 Gateway Computing Environments Workshop (GCE)*, 1–8 (IEEE, 2010).

