# Supplementary material for: An integrative genomic and phenomic analysis to investigate the nature of plant species in Escallonia (Escalloniaceae)
Source: Sci Rep. 2021 Dec 14;11:24013. doi: 10.1038/s41598-021-03419-0 (PMC8671583; doi:10.1038/s41598-021-03419-0)
Supplement: Supplementary file 1 — Supplementary Information. [file 41598_2021_3419_MOESM1_ESM.pdf]

Supplementary Materials for

An integrative genomic and phenomic analysis to investigate the nature of plant species in *Escallonia* (Escalloniaceae)

Sarah J. Jacobs, Michael C. Grundler, Claudia L. Henriquez, Felipe Zapata

Contents

1 Materials and Methods **5**
1.1 Sampling 5
1.2 Phenomics 6
1.3 Genomics 7
1.4 Data Integration 8

2 Results **9**
2.1 Current state of taxonomic species using genomics data 9
2.2 Clade I 9
2.2.1 Sampling 9
2.2.2 Phenomics 9
2.2.2.1 Current state of taxonomic species 9
2.2.2.2 Model-based species discovery 9
2.2.3 Genomics 9
2.2.3.1 Sensitivity Tests 9
2.2.3.2 Model-based species discovery 10
2.2.4 Data Integration 10
2.2.5 Correspondence between taxonomic species and model-based species 11
2.3 Clade II 11
2.3.1 Sampling 11
2.3.2 Phenomics 11
2.3.2.1 Current state of taxonomic species 11
2.3.2.2 Model-based species discovery 11
2.3.3 Genomics 11
2.3.3.1 Sensitivity Tests 11
2.3.3.2 Model-based species discovery 12
2.3.4 Data Integration 12
2.3.5 Correspondence between taxonomic species and model-based species 13
2.4 Clade III 13
2.4.1 Sampling 13
2.4.2 Phenomics 13

2.4.2.1 Current state of taxonomic species 13
2.4.2.2 Model-based species discovery 13
2.4.3 Genomics 13
2.4.3.1 Sensitivity Tests 13
2.4.3.2 Model-based species discovery 14
2.4.4 Data Integration 14
2.4.5 Correspondence between taxonomic species and model-based species 15
2.5 Clade IV 15
2.5.1 Sampling 15
2.5.2 Phenomics 15
2.5.2.1 Current state of taxonomic species 15
2.5.2.2 Model-based species discovery 15
2.5.3 Genomics 16
2.5.3.1 Sensitivity Tests 16
2.5.3.2 Model-based species discovery 16
2.5.4 Data Integration 16
2.5.5 Correspondence between taxonomic species and model-based species 17
2.6 Clade V 17
2.6.1 Sampling 17
2.6.2 Phenomics 17
2.6.2.1 Current state of taxonomic species 17
2.6.2.2 Model-based species discovery 17
2.6.3 Genomics 18
2.6.3.1 Sensitivity Tests 18
2.6.3.2 Model-based species discovery 18
2.6.4 Data Integration 18
2.6.5 Correspondence between taxonomic species and model-based species 19
2.7 Clade VI 20
2.7.1 Sampling 20
2.7.2 Phenomics 20
2.7.2.1 Current state of taxonomic species 20
2.7.2.2 Model-based species discovery 20
2.7.3 Genomics 20
2.7.3.1 Sensitivity Tests 20
2.7.3.2 Model-based species discovery 20
2.7.4 Data Integration 21
2.7.5 Correspondence between taxonomic species and model-based species 22

3 Tables **23**
3.0.1 Table S1: Taxon Sampling 23
3.0.2 Table S2: Model Adequacy 35
3.1 Clade I 35
3.1.0.1 Table S2: Genomic dataset details 36
3.1.0.2 Table S3: Genogroup delimitation 37
3.1.0.3 Table S4: Correspondence between taxonomic species, phenogroups, and genogroups 38
3.2 Clade II 39
3.2.0.1 Table S5: Genomic dataset details 39
3.2.0.2 Table S6: Genogroup delimitation 40

3.2.0.3 Table S7: Correspondence between taxonomic species, phenogroups, and genogroups . . . 41
3.3 Clade III . . . 42
3.3.0.1 Table S8: Genomic dataset details . . . 42
3.3.0.2 Table S9: Genogroup delimitation . . . 43
3.3.0.3 Table S10: Correspondence between taxonomic species, phenogroups, and genogroups . . . 44
3.4 Clade IV . . . 45
3.4.0.1 Table S11: Genomic dataset details . . . 45
3.4.0.2 Table S12: Genogroup delimitation . . . 46
3.4.0.3 Table S13: Correspondence between taxonomic species, phenogroups, and genogroups . . . 47
3.5 Clade V . . . 48
3.5.0.1 Table S14: Genomic dataset details . . . 48
3.5.0.2 Table S15: Genogroup delimitation . . . 49
3.5.0.3 Table S16: Correspondence between taxonomic species, phenogroups, and genogroups . . . 50
3.6 Clade VI . . . 51
3.6.0.1 Table S17: Genomic dataset details . . . 51
3.6.0.2 Table S18: Genogroup delimitation . . . 52
3.6.0.3 Table S19: Correspondence between taxonomic species, phenogroups, and genogroups . . . 53

4 Figures **54**
4.1 Species Trees . . . 54
4.1.1 Fig S1: Phylogenetic trees (two specimens per taxonomic species) . . . 54
4.1.2 Fig S2: Phylogenetic trees (four specimens per taxonomic species) . . . 55
4.2 Clade I . . . 56
4.2.1 Fig S3: Taxon sampling . . . 56
4.2.2 Fig S4: Geographic distribution . . . 57
4.2.3 Fig S5: Current state of taxonomic species with phenotypic data . . . 58
4.2.4 Fig S6: Phenogroup delimitation: Gaussian finite mixture modeling . . . 59
4.2.5 Fig S7: Sensitivity tests with 75% missing data . . . 60
4.2.6 Fig S8: Sensitivity tests with 50% missing data . . . 61
4.2.7 Fig S9: Sensitivity tests with 25% missing data . . . 62
4.2.8 Fig S10: Genogroup delimitation: Genotypic cluster model . . . 63
4.2.9 Fig S11: Genogroup delimitation. Cladogenesis to anagenesis model . . . 64
4.2.10 Fig S12: Genogroup delimitation: Reproductive isolation model . . . 65
4.2.11 Fig S13: Data integration . . . 66
4.3 Clade II . . . 67
4.3.1 Fig S14: Taxon sampling . . . 67
4.3.2 Fig S15: Geographic distribution . . . 68
4.3.3 Fig S16: Current state of taxonomic species with phenotypic data . . . 69
4.3.4 Fig S17: Phenogroup delimitation: Gaussian finite mixture modeling . . . 70
4.3.5 Fig S18: Sensitivity tests with 75% missing data . . . 71
4.3.6 Fig S19: Sensitivity tests with 50% missing data . . . 72
4.3.7 Fig S20: Sensitivity tests with 25% missing data . . . 73
4.3.8 Fig S21: Genogroup delimitation: Genotypic cluster model . . . 74
4.3.9 Fig S22: Genogroup delimitation. Cladogenesis to anagenesis model . . . 75
4.3.10 Fig S23: Genogroup delimitation: Reproductive isolation model . . . 76

4.3.11 Fig S24: Data integration 77
4.4 Clade III 78
4.4.1 Fig S25: Taxon sampling 78
4.4.2 Fig S26: Geographic distribution 79
4.4.3 Fig S27: Current state of taxonomic species with phenotypic data 80
4.4.4 Fig S28: Phenogroup delimitation: Gaussian finite mixture modeling 81
4.4.5 Fig S29: Sensitivity tests with 75% missing data 82
4.4.6 Fig S30: Sensitivity tests with 50% missing data 83
4.4.7 Fig S31: Sensitivity tests with 25% missing data 84
4.4.8 Fig S32: Genogroup delimitation: Genotypic cluster model 85
4.4.9 Fig S33: Genogroup delimitation. Cladogenesis to anagenesis model 86
4.4.10 Fig S34: Genogroup delimitation: Reproductive isolation model 87
4.4.11 Fig S35: Data integration 88
4.5 Clade IV 89
4.5.1 Fig S36: Taxon sampling 89
4.5.2 Fig S37: Geographic distribution 90
4.5.3 Fig S38: Current state of taxonomic species with phenotypic data 91
4.5.4 Fig S39: Phenogroup delimitation: Gaussian finite mixture modeling 92
4.5.5 Fig S40: Sensitivity tests with 75% missing data 94
4.5.6 Fig S41: Sensitivity tests with 50% missing data 95
4.5.7 Fig S42: Sensitivity tests with 25% missing data 96
4.5.8 Fig S43: Genogroup delimitation: Genotypic cluster model 97
4.5.9 Fig S44: Genogroup delimitation. Cladogenesis to anagenesis model 98
4.5.10 Fig S45: Genogroup delimitation: Reproductive isolation model 99
4.5.11 Fig S46: Data integration 100
4.6 Clade V 101
4.6.1 Fig S47: Taxon sampling 101
4.6.2 Fig S48: Geographic distribution 102
4.6.3 Fig S49: Current state of taxonomic species with phenotypic data 103
4.6.4 Fig S50: Phenogroup delimitation: Gaussian finite mixture modeling 104
4.6.5 Fig S51: Sensitivity tests with 75% missing data 105
4.6.6 Fig S52: Sensitivity tests with 50% missing data 106
4.6.7 Fig S53: Sensitivity tests with 25% missing data 107
4.6.8 Fig S54: Genogroup delimitation: Genotypic cluster model 108
4.6.9 Fig S55: Genogroup delimitation. Cladogenesis to anagenesis model 109
4.6.10 Fig S56: Genogroup delimitation: Reproductive isolation model 110
4.6.11 Fig S57: Data integration 111
4.7 Clade VI 112
4.7.1 Fig S58: Taxon sampling 112
4.7.2 Fig S59: Geographic distribution 113
4.7.3 Fig S60: Current state of taxonomic species with phenotypic data 114
4.7.4 Fig S61: Phenogroup delimitation: Gaussian finite mixture modeling 115
4.7.5 Fig S62: Sensitivity tests with 75% missing data 116
4.7.6 Fig S63: Sensitivity tests with 50% missing data 117
4.7.7 Fig S64: Sensitivity tests with 25% missing data 118
4.7.8 Fig S65: Genogroup delimitation: Genotypic cluster model 119
4.7.9 Fig S66: Genogroup delimitation. Cladogenesis to anagenesis model 120
4.7.10 Fig S67: Genogroup delimitation: Reproductive isolation model 121
4.7.11 Fig S68: Data integration 122

5 References 124

A Appendix A: Manuscript in Spanish 130

B Appendix B: Analysis Session Information 143

1 Materials and Methods

1.1 Sampling

Taxon sampling All 848 specimens included in the study were assigned to 29 taxonomic species according to the only genus-wide taxonomic monograph.[1] These specimens covered the geographic range of all taxonomic species whenever possible (i.e., specimens came from well-spaced localities across the geographic range of each species). Specimens assigned to *E. virgata* and *E. pulverulenta* were excluded from our study because in initial phylogenetic analyses these taxonomic species did not show stable and well supported phylogenetic relationships. Specimens assigned to *E. callcottiae, E. chlorophylla, E. cordobensis, E. gayana, E. harrisii, E. hispida, E. obtusissima, E. salicifolia*, and *E. serrata* were not included in our study because we could not find enough herbarium specimens to reliably investigate the nature of these taxonomic species, we failed to extract ancient DNA from old herbarium specimens, or we could not locate any populations of these taxonomic species in the field.

Phenomic data collection We meaured leaf length, leaf width, pedicel length, ovary length, length of calyx tube, length of calyx lobes, petal length, petal width, filament length, style length to characterize the geographic pattern of phenotypic variation across *Escallonia*. We chose those traits because they have been used in previous taxonomic studies to characterize and delimit all taxonomic species in this genus.[1,See also 2] Furthermore, after our careful study of ca. 3,500 herbarium collections, we confirmed these traits are variable across the entire geographic range of this genus. We used only mature leaves and flowers in all specimens to measure each phenotypic trait. All traits were measured using a digital caliper (Digimatic CD-6" CS, Mitutoyo Japan). Vegetative traits were measured on dried specimens. Floral traits were measured on flowers that were rehydrated and examined on a stereoscopic dissecting microscope (SMZ645, Nikon USA). All measurements were recorded from three different structures (i.e., three flowers) for each specimen whenever possible, and then averaged to generate character measurements for each specimen.

Genomic data collection Genomic DNA extraction and purification was performed following a modified version of the CTAB extraction protocol[3,4] that incorporates a pre-wash step[5] to aid in the removal of polyphenols and proteins prior to extraction. Genomic DNA quantification was performed using a Qubit fluorometer v.3.0 (Invitrogen by Thermo Fisher Scientific, Carlsbad, CA, USA) and an Agilent 2200 TapeStation (Agilent Technologies, Santa Clara, CA, USA). Prior to sequencing, size selection for fragment length of $375 - 525$ bp was performed on a PippenPrep (Sage Science, Beverly, MA, USA). Libraries were pooled in groups of 96 specimens and sequenced across multiple lanes of 100PE sequencing on the Illumina HiSeq4000 Sequencing Platform at the Broad Stem Cell Research Center at the University of California, Los Angeles. To determine potential batch effects, we prepared and sequenced a subset of the same specimens across different lanes. Though we recovered different number of sequence reads across replicated runs, the loci were largely repeatable and duplicated specimens always clustered together in preliminary phylogenetic analyses. Therefore, we inferred no noticeable batch effects. We used `iPyrad` v0.7.28[6] to demultiplex, filter, assemble, and call variants. We filtered reads with a Phred score less than 33 at more than five positions and required a minimum of 10 reads for statistical base calling. We used a clustering threshold of 0.90 sequence similarity to cluster a maximum of 5,000 reads into loci. We required at least four specimens to include a locus to generate an initial data matrix. We used `VCFtools` v0.1.14[7]

and custom-made scripts to further filter this data matrix. We first filtered specimens with missing data from 95% of loci, retained only biallelic sites, and then chose the single nucleotide polymorphism (SNP) closest to the center of each locus to minimize the effects of linkage disequilibrium for some analyses. For other analyses, we used the full sequence per locus (See below). We used the same approach to generate three matrices with different amounts of missing data by first filtering loci missing from 25%, 50%, and 75% of the specimens and then applying the same filters described above. For analysis within clades, we removed the outgroup (*Valdivia gayana*) and applied our assembly and filtering strategy for each clade independently (See Results).

1.2 Phenomics

Current state of taxonomic species Based on the most recent taxonomic monograph,[1] we extracted the minimum and maximum values reported for ten quantitative traits used in the descriptions of all taxonomic species. In a few selected cases, the taxonomic description provided a single value for a given trait. Because it is impossible for any quantitative continuous trait to be fixed with no variation (or measurement error) in nature, we conservatively added and subtracted 0.5 units (in our case mm.) to the reported value to create a range. When this adjustment resulted in a minimum value $\leqslant 0$, we adjusted this value to be > 0 according to the precision of our measurement tool (0.1 mm.). For example, when the reported measurement was 0.5 mm., the standard procedure would indicate the range to be $[0, 1]$ mm. Therefore, we adjusted this range to be $[0.1, 1]$ mm. Of the 39 taxonomic species described in the taxonomic monograph,[1] 11 included subspecies. Because we only considered variation at the level of species, we subsumed subspecific variation into cumulative values at the species level. Hence, we conservatively made the range of species with subspecies as inclusive as possible.

Model-based evidence for species The fundamental model for the distribution of continuous quantitative traits within a species is well-grounded in quantitative genetics and evolutionary theory.[8] In addition to the assumption of polygenic inheritance and random mating, it also assumes that phenotypic differences between species do not reflect ontogenetic or environmentally-induced variation.[9] Because we only measured mature organs (See above), we inferred that any phenotypic differences did not reflect ontogenetic variation. Additionally, though the genetic architecture of the phenotypic traits we measured in *Escallonia* is not known, previous studies in other angiosperms have shown that these vegetative[10–12] and reproductive[13–15] traits are polygenic, with limited environmental effects. Therefore, we assumed the the same genetic architecture for the traits used here. Lastly, no formal studies on the reproductive biology of *Escallonia* are available; however, observations of populations in the field do not indicate any skew in reproductive mode (pers. obs.). Therefore, we assumed these plants display random mating. The Fisherian model, of course, has stronger explanatory power in an explicit spatial context, particularly in situations that afford the opportunity for species to come into contact.[16] Because our continent-wide sampling includes specimens from multiple populations in contact, we could examine phenotypic variation when species co-occur.

In addition to the information criteria used to determine the best fit model, we used a likelihood ratio test (LRT) to assess whether models with nested numbers of phenogroups of equal shape and orientation as the best fit model were appropriate. To carry out this analysis, we specified the parameters of the best fit model, sequentially increased the number of phenogroups, and estimated the LRT statistics (LRTs) comparing the simple model (i.e., fewer phenogroups) *vs.* the more complex model (i.e., more phenogroups). A large value of the LRTs provides evidence against the simpler model. To assess significance of the LRT, we created a null distribution of LRTs using bootstrap simulations with 999 replicates. For this analysis, we used `mclust` v5.4.5.[17]

1.3 Genomics

Model-based evidence for species

Sensitivity Tests In order to assess the sensitivity of our analyses to the amount of missing data, we used the three matrices (25%, 50%, 75% missing data) in each clade for three kinds of analysis. First, we used a concatenated matrix with complete sequences for all loci to run a phylogenetic analysis in `IQ-TREE` v2.0.3 with ultrafast bootstrap approximation to assess branch support.[18,19] Second, we used our matrix with one SNP per locus to run Principal Component Analysis (PCA)[20] and visually detect clusters. For this analysis, we used the R package `SNPRelate` v4.0.[21] Third, using the same matrix we used for PCA, we ran model-based clustering to detect ancestry of specimens.[22] For this analysis, we ran `ADMIXTURE`[22] independently 10 times specifying 1 to $n + n/2$ number of genomic clusters, where n is equal to the number of taxonomic species currently hypothesized to exist within each clade.

RI model (reproductive isolation model) `BPP` requires that specimens are assigned to demes *a priori.* To infer the number of demes and the assignment of specimens to demes, we first ran the software `STRUCTURE` v2.3.4[23] with default priors for 10 replicates in parallel.[24] In each replicate, we fitted different models specifying 1 to $n + n/2$ number of demes, where n is equal to the number of taxonomic species currently hypothesized to exist within each clade. We recorded $1,000,000$ samples from the posterior probability after applying a burnin of 10%, summarized the results across replicates with `StructureHarvester` v0.6.94,[25] and used the ΔK statistic to select the best fit model.[26] Based on the best supported model, we assigned specimens to demes using the software `CLUMPP` v1.1.2.[27] Second, we used the software `rMaverick` v1.0.5[28] which uses thermodynamic integration instead of the heuristic estimators used in `STRUCTURE` to infer the number of demes and the assignment of specimens to demes. For this analysis, we used the same data matrix and fitted the same models specifying different number of demes as with `STRUCTURE`. We used an admixture model ($\alpha = 0.1$) with default priors and ran 20 rungs. Each rung used $30,000$ sample iterations and 20% samples as burn-in. To assess convergence of the results, we plotted the weighted log-likelihoods for each rung and assessed for smooth transitions between rungs. We chose the best fit model based on the highest posterior probability.

In addition to deme assignments, `BPP` requires specifying prior probabilities for the species delimitation/species tree models and prior probabilities within each of these models for the population sizes parameter (θ) and the age of the root in each species tree (τ_0). For the species delimitation/species tree models, we used the default prior (Prior 1), which assigns equal probabilities to the rooted species trees.[29] Within each species delimitation/species tree model, we assigned the inverse-gamma prior ($\alpha = 2$, $\beta = 0.001$ or $\beta = 0.01$) for both θ and τ_0. We assigned the same priors across all clades.

`BPP` uses a reversible-jump Markov chain Monte Carlo (rjMCMC) algorithm to move between different species delimitation models.[30] To determine proper mixing of the rjMCMC, we ran a series of replicated analyses within each clade. When we assigned specimens to two demes, we ran four replicates. When we assigned specimens to three or more demes, we ran eight replicates. Across replicates, we changed the algorithms (0 and 1) implemented in `BPP`[30] and the random starting tree. We further replicated the same series of analyses but using ca. 5% of the loci randomly sampled without replacement. Because analyses with fewer loci are less susceptible to mixing problems, we used results from these analyses to indicate potential issues with the larger datasets.

Model selection It was computationally infeasible to use BFD* in `SNAPP` with the full datasets given the large number of specimens and loci within each clade (See Results). Therefore, we subsampled each genogroup to two specimens maximizing the data available (genomics, phenomics, and geographic distribution) and then reduced the number of loci to a computationally tractable size. To accomplish this, we used a concordance factor analysis as implemented `IQ-TREE` v2.0.3.[32] For this analysis, we first inferred phylogenies for each locus using `IQ-TREE` (coupled with model selection).[33] We then used the

phylogeny generated with the concatenated matrix per clade to calculate gene concordance factors (gCF: the fraction of decisive gene trees concordant for a branch) and the site concordance factors (sCF: the fraction of decisive alignment sites supporting a branch).[19] We ranked loci according to sCF and selected sites with sCF $\geqslant 0.5$. From the resulting list of loci, we applied our filters to choose one SNP per locus as described above.

`SNAPP` requires specifying prior probabilities for the speciation rate (λ) and the expected divergence (Θ). We used a Γ distribution as hyperprior to accommodate uncertainty in λ. To set the parameters α and β describing the Γ distribution, we used `pylue` (https://github.com/joaks1/pyule) to estimate the expected height of the species trees. Based on these analyses, we used a diffused setting for $\alpha = 2$ and adjusted β such that the mean of the Γ distribution centered around the expected height of the species trees. Similarly, we assigned a Γ prior on Θ using $\alpha = 2$ and adjusted β such that the mean of the Γ distribution centered around the average pairwise sequence divergence among all individuals within clades.

Model adequacy Because genogroups inferred with `mPTP`[35] were consistently favored by Bayes Factor delimitation in all but one case, we performed a goodness-of-fit test to determine if the model used by `mPTP` was an adequate fit to the data. `mPTP` delimits species by using the empirical distribution of branch lengths (measured as substitutions per site) to identify transition points from a between species branching process to a within species branching process. Specifically, for each delimited species `mPTP` fits the branching events to a distinct exponential distribution. We therefore assessed the adequacy of `mPTP` by asking whether or not the branching events within each delimited species could plausibly have originated from the fitted exponential distribution. We used the Kolmogorov-Smirnov test to compare the empirical cumulative distribution function of branch lengths to the theoretical cumulative distribution function under the hypothesis that the data were generated from an exponential distribution. A large discrepancy between these two distributions (revealed by a small P-value) indicates that the observed data are unlikely to have been generated from the fitted exponential distribution and that `mPTP` is inadequately modeling the coalescent process within delimited species. Results from these tests are shown in (Table S2).

1.4 Data Integration

To quantify the association between phenogroups and genogroups within clades, we used the Goodman Kruskal's tau ($GK\tau$) statistic.[36] This is an asymmetric association measure between two categorical variables with values ranging from zero (no association between variables) to one (perfect association between variables). The asymmetry of $GK\tau$ is particularly useful because in many instances the variability in variable x that is explainable by variations in y may be different from the variability in y that is explainable by variations in x. Therefore, we estimated the association between phenogroups and genogroups ($GK\tau_{pg}$) and the association between genogroups and phenogroups ($GK\tau_{gp}$) within each clade using all specimens that had both types of data. When the value of either of these indices is equal to 1, there is perfect association between one grouping category and the other grouping category, implying that knowledge of one group membership perfectly predicts membership of the other type of grouping. For instance, $GK\tau_{pg} = 1$ means that knowledge of phenogroup membership perfectly predicts genogroup membership. Therefore, if a clade only contains 'good species', $GK\tau_{pg}$ and $GK\tau_{gp}$ are equal to 1. By contrast, values of $GK\tau < 1$ imply that knowledge of one grouping category provides lower predictive ability of the alternative grouping category. For instance, $GK\tau_{pg} < 1$ means that knowledge of phenogroup membership provides lower ability to predict genogroup membership. Thus, a clade with $GK\tau_{pg} < 1$ implies the presence of 'phenotypic cryptic species'. Similarly, a clade with $GK\tau_{gp} < 1$ implies the presence of 'genetic cryptic species'. The $GK\tau$ statistic is calculated on specimens with both phenotypic and genetic data available, therefore it does not incorporate information from unknown specimens. To calculate $GK\tau$, we used the `R` package `GoodmanKruskal` v0.0.2.[37]

2 Results

2.1 Current state of taxonomic species using genomics data

The lineage and species trees showed that the phylogeny of *Escallonia* consisted of six well-supported clades (hereafter, Clades I-VI) (Figure S1). When we included more than two specimens per taxonomic species, we recovered the same six clades, yet not all specimens determined to the same taxonomic species were each other's closest relatives (Figure S2).

2.2 Clade I

2.2.1 Sampling

A total of 39 specimens were included in this clade (Figure S3). We measured phenotypic traits on 37 specimens; 33 were fertile specimens (thus having both leaf and floral traits) and 4 were sterile specimens (thus having leaf traits only). Only fertile specimens were included in downstream phenomic analyses. We collected genomic data for 14 specimens; 8 specimens were fertile, 4 were sterile, and 2 were not available for phenotypic analyses. All 14 specimens were included in downstream genomic analyses except in our model selection analysis (See below). The 39 specimens covered the geographic range of this clade and belonged to two taxonomic species (Figure S4, Table S1).

2.2.2 Phenomics

2.2.2.1 Current state of taxonomic species The 10-cubes defining the taxonomic species that belong to this clade did not overlap in 10-dimensional phenospace (Figure S5). The matching-prediction analysis showed that 0% of the specimens fall inside any 10-cube, and no specimens fall inside their correct 10-cube (Figure S5).

2.2.2.2 Model-based species discovery We found four principal components to be most useful for group discrimination. The naive model specifying two distinct phenogroups of equal shape and volume received the strongest support using both the BIC and ICL criteria (BIC= 54.03, ICL= 53.86). Support for alternative models was considerably lower, including the Taxonomy model[1] specifying two phenogroups (ΔBIC=8.225) and the Taxonomy Unaware model specifying one phenogroup (ΔBIC= 20.574) (Figure S6). A LRT further corroborated that a model with two phenogroups of equal shape and volume was a better fit to the data than models with alternative phenogroup composition (p-value < 0.05).

2.2.3 Genomics

2.2.3.1 Sensitivity Tests In total, we recovered 44,630 loci across 14 sequenced individuals. 22,999 loci were present in at least four individuals. The number of loci in the three matrices with different levels of missing data is presented in Table S3. Results from Principal Components Analysis, phylogenetic analysis, and genomic clustering did not differ (or differed minimally) across the three matrices, suggesting that our data were robust to the amount of missing data (Figures S7, S8, S9). Therefore, we performed all downstream analyses using the smallest data matrix for computational efficiency.

2.2.3.2 Model-based species discovery **Genotypic clusters (GC model)** We found two dimensions to be most useful to faithfully represent the genotypic data in fewer dimensions (2 dimensions = 0.0079% stress). In this reduced space, a model specifying three genotypic clusters received the highest support (BIC= 431.5805, next best model ΔBIC= 5.4468, three clusters; Figure S10).

Transition between cladogenesis and anagenesis (CA model) Given the phylogeny reconstructed with the concatenated alignment of full sequences per locus, a model with two genogroups was the best fit to the data (Figure S11). Across all independent runs, all nodes identifying genogroups strongly identified such nodes as transitions between cladogenesis and anagenesis (Fig S11; See values closer to 0 subtending red groups).

Reproductive isolation (RI model) A model specifying two demes received the strongest support based on the ΔK statistic using `STRUCTURE`. An identical model specifying two demes using `MAVERICK` had the highest posterior probability ($pp > 0.99$). Using these deme assignments, we found consistent and strong support for a model specifying two genogroups, for both the full and reduced datasets ($pp = 1.0$, Figure S12).

Model Selection We filtered our matrix and retained 2,320 loci to conduct this analysis (Table S3). Of the four models described above, three were identical. The CA model and the two RI models (using deme assignments based on `STRUCTURE` and `MAVERICK`) each identified and assigned the same specimens to the same two genogroups. We compared this model with the GC model using Bayes Factors. Each model required more than 500,000 samples to stabilize (as estimated from ESS values for each step). In total, each analysis used 24 steps. The GC model (three genogroups) was the top-ranked model with decisive support relative to the other model[38] (Table S4).

2.2.4 Data Integration

Based on the best fitting models for species discovery using phenotypic and genome-wide variation, we assigned each specimen to its corresponding phenogroup and genogroup (Figure S13). In total, we assigned specimens to two species (See main text, Figure 2). These two species matched uniquely one phenogroup to one genogroup (Figure S13; See for example phenogroup 1 - genogroup 3). Therefore, these two species are recognized as 'good species'. We also discovered one genogroup with specimens for which we did not have phenotypic data available (unknown specimens) (Figure S13, genogroup 2; See below). The measure of association of phenogroups to genogroups was $GK\tau_{pg} = 1.0$ and the association of genogroups to phenogroups was $GK\tau_{gp} = 1.0$. This indicated that genogroup membership is a perfect predictor of phenogroup membership (and *vice versa*), and is reflected in the fact that this clade only includes 'good species'.

We examined all the specimens in their phylogenetic and geographic contexts to gain insight into the plausible species identity of the unknown specimens and the nature of species in this clade. Given that specimens in both phenogroups 1 and 2 do not co-occur locally, we speculate that the unknown specimens assigned to phenogroup 1 most likely belong to genogroup 3, and that the unknown specimens assigned to phenogroup 2 most likely belong to either genogroup 1 or 2 (Figure S13). Given this plausible assignment and that genogroups 1 and 2 are sister lineages, our reasoning implies that species in phenogroup 2 and genogroups 1 and 2 are 'phenotypic cryptic species' (Figure S13). However, it is also plausible that genogroups 1 and 2 correspond to isolated populations of a single species, which is genetically structured along elevation. These uncertainties aside, our results showed that at the spatial scale of our study, the 'good species' do not co-occur in close geographic proximity with one another suggesting complete differentiation in allopatry (Figure S13; See phenogroup 1 - genogroup 3 and phenogroup 2 - genogroup 3).

2.2.5 Correspondence between taxonomic species and model-based species

We assigned all specimens to their corresponding taxonomic species, phenogroup, and genogroup. Only specimens assigned to one taxonomic species (*E. micrantha*) showed perfect correspondence to a single phenogroup and a single genogroup. All other specimens in this clade were assigned to the taxonomic species *E. millegrana*, a single phenogroup (phenogroup 2), and multiple genogroups (See main text, Table 4; Table S5).

2.3 Clade II

2.3.1 Sampling

A total of 38 specimens were included in this clade (Figure S14). We measured phenotypic traits on 38 specimens; 33 were fertile specimens (thus having both leaf and floral traits) and 5 were sterile specimens (thus having leaf traits only). Only fertile specimens were included in downstream phenomic analyses. We collected genomic data for 15 specimens; 10 specimens were fertile, 5 were sterile, and 0 were not avaialable for phenotypic analyses. All 15 specimens were included in downstream genomic analyses except in our model selection analysis (See below). The 38 specimens covered the geographic range of this clade and belonged to two taxonomic species (Figure S15, Table S1).

2.3.2 Phenomics

2.3.2.1 Current state of taxonomic species The 10-cubes defining the taxonomic species that belong to this clade did not overlap in 10-dimensional phenospace (Figure S16). The matching-prediction analysis showed that 0% of the specimens fall inside any 10-cube, with no specimens falling inside their correct 10-cube (Figure S16).

2.3.2.2 Model-based species discovery We found four principal components to be most useful for group discrimination. The naive model specifying three distinct phenogroups, of equal shape, volume, and orientation received the strongest support using both the BIC and ICL criteria (BIC= 71.729, ICL= 69.411). Support for alternative models was considerably lower, including the Taxonomy model[1] specifying two phenogroups (ΔBIC= 53.956) and the Taxonomy Unaware model specifying one phenogroup (ΔBIC= 24.201) (Figure S17). A LRT further corroborated that a model with three phenogroups of equal shape, volume, and orientation was a better fit to the data than models with more phenogroups (p-value < 0.05)

2.3.3 Genomics

2.3.3.1 Sensitivity Tests In total, we recovered 66,064 loci across 15 sequenced individuals. 30,440 loci were present in at least four individuals. The number of loci in the three matrices with different levels of missing data is presented in Table S6. Results from Principal Components Analysis, phylogenetic analysis, and genomic clustering did not differ (or differed minimally) across the three matrices, suggesting that our data were robust to the amount of missing data (Figures S18, S19, S20). Therefore, we performed all downstream analyses using the smallest data matrix for computational efficiency.

2.3.3.2 Model-based species discovery

Genotypic clusters (GC model) We found two dimensions to be most useful to faithfully represent the genotypic data in fewer dimensions (2 dimensions = 0.0082% stress). In this reduced space, a model specifying three genotypic clusters received the highest support (BIC= 347.3602, next best model ΔBIC= 41.7965, three clusters; Figure S21).

Transition between cladogenesis and anagenesis (CA model) Given the phylogeny reconstructed with the concatenated alignment of full sequences per locus, a model with four genogroups was the best fit to the data (Figure S22). Across all independent runs, all nodes identifying genogroups strongly identified such nodes as transitions between cladogenesis and anagenesis (Fig S22; See values closer to 0 subtending red groups).

Reproductive isolation (RI model) A model specifying two demes received the strongest support based on the ΔK statistic using `STRUCTURE`. Using this deme assignment, we found consistent and strong support for a model specifying two genogroups, for both the full and reduced datasets (*pp* = 1.0, Figure S23). A model specifying three demes using `MAVERICK` had the highest posterior probability (*pp* > 0.99). Using this deme assignment, we found consistent and strong support for a model specifying three genogroups, for both the full and reduced datasets (*pp* = 1.0, Figure S23). All replicated analyses used to identify genogroups with both deme assignments showed consistent support for two and three genogroups, respectively.

Model Selection We filtered our matrix and retained 3,005 loci to conduct this analysis (Table S6). Of the four models described above, the CA model identified four genogroups, the GC model and the RI models using deme assignments based on `MAVERICK`) both identified and assigned the same specimens to the same three genogroups, and the RI model using `STRUCTURE` for deme assignment recognized two genogroups (Table S7). We compared all models using Bayes Factors and found that each model required more than 500,000 samples to stabilize (as estimated from ESS values for each step), with the exception of the RI model based on `STRUCTURE` assignments which required 250,000 samples to stabilize. In total, each analysis used 24 steps. The CA model (four genogroups) was the top-ranked model with decisive support relative to the other models[38] (Table S7).

2.3.4 Data Integration

Based on the best fitting models, we assigned specimens with overlapping phenotypic and genomic data to five species (See main text, Figure 2). None of these assignments matched uniquely one phenogroup to one genogroup, indicating there are no ‘good species’ in this clade (Figure S24). The specimens assigned to phenogroup 1 were asssigned to genogroups 1 and 3, and the specimens assigned to phenogroup 3 were assigned to genogroups 1, 2, and 4. Therefore, we recognized these sets as ‘phenotypic cryptic species’. Other specimens assigned to genogroup 1 were assigned to phenogroups 1 and 3, which indicates there are also ‘genetic cryptic species’ in this clade (Figure S24). We did not have genomic data available for the specimens that we assigned to phenogroup 2 (unknown specimens). The measure of association of phenogroups to genogroups was $GK\tau_{pg} = 0.322$, while the association of genogroups to phenogroups was $GK\tau_{gp} = 0.762$. This indicated that genogroup membership is a better predictor of phenogroup membership than the phenogroup membership is of genogroup membership. In other words, this shows there are more ‘phenotypic cryptic species’ in this clade than ‘genotypic cryptic species’.

We examined all the specimens in their phylogenetic and geographic contexts to gain insight into the plausible species identity of the unknown specimens and the nature of species in this clade. Considering the geographic distribution of the unknown specimens assigned to genogroup 2 and genogroup 3 as well as our extensive field and herbarium work, we speculate that these specimens likely belong to phenogroups 3 and 1, respectively. However, it is difficult to speculate the likely assignment of the unknown specimens in genogroup 1 given that this genogroup includes specimens from phenogroups 1 and 3, which do not show a clear pattern of geographic structure. Indeed, given the overall lack of geographic structure of

phenogroups 1 and 3, it is not possible to suggest the genogroup assignment of all the unknwon specimens in these phenogroups (See also estimates of $GK\tau_{pg}$ above). These uncertainties aside, our results showed that at the spatial scale of our study all species co-occur in close geographic proximity with at least one other species (Figure S24). Our phylogenetic and geographic results also showed that the 'phenotypic cryptic species' are allopatric and not sister to each other suggesting convergent evolution in phenotype perhaps driven by niche conservatism[39] (See for example phenogroup 3; all specimens are restricted to dry inter-andean valleys). However, extensive sampling is needed to discern the nature of species in this clade with increased rigor.

2.3.5 Correspondence between taxonomic species and model-based species

We assigned all specimens to their corresponding taxonomic species, phenogroup, and genogroup. The specimens assigned to either taxonomic species did not show perfect correspondence to a single phenogroup and a single genogroup. Instead, all specimens were assigned to phenogroups and genogroups which were shared across both taxonomic species (See main text, Table 4; Table S8).

2.4 Clade III

2.4.1 Sampling

A total of 174 specimens were included in this clade (Figure S25). We measured phenotypic traits on 171 specimens; 130 were fertile specimens (thus having both leaf and floral traits) and 41 were sterile specimens (thus having leaf traits only). Only fertile specimens were included in downstream phenomic analyses. We collected genomic data for 53 specimens; 27 specimens were fertile, 23 were sterile, and 3 were not avaialable for phenotypic analyses. All 53 specimens were included in downstream genomic analyses except in our model selection analysis (See below). The 174 specimens covered the geographic range of this clade and belonged to six taxonomic species (Figure S26, Table S1)

2.4.2 Phenomics

2.4.2.1 Current state of taxonomic species The 10-cubes defining the taxonomic species that belong to this clade did not overlap in 10-dimensional phenospace. The matching-prediction analysis showed that only 0.0026% of the specimens fall inside any 10-cube, with one specimen falling inside its correct 10-cubes (Figure S27).

2.4.2.2 Model-based species discovery We found eight principal components to be most useful for group discrimination. The naive model specifying five distinct phenogroups of equal shape and orientation received the strongest support using both the BIC and ICL criteria (BIC= 387.153, ICL= 382.608). Support for alternative models was considerably lower, including the Taxonomy model[1] specifying six phenogroups (ΔBIC= 333.767) and the Taxonomy Unaware model specifying four phenogroups (ΔBIC= 216.314) (Figure S28). A LRT further corroborated that a model with five phenogroups of equal shape and orientation was a better fit to the data than models with more phenogroups (p-value < 0.05)

2.4.3 Genomics

2.4.3.1 Sensitivity Tests In total, we recovered $91,032$ loci across 53 sequenced individuals. $43,597$ loci were present in at least four individuals. The number of loci in the three matrices with different

levels of missing data is presented in Table S9. Results from Principal Components Analysis, phylogenetic analysis, and genomic clustering did not differ (or differed minimally) across datasets, suggesting that our data are robust to the amount of missing data (Figures S29, S30, S31). Therefore, we performed all downstream analyses using the smallest data matrix for computational efficiency.

2.4.3.2 Model-based species discovery

Genotypic clusters (GC model) We found two dimensions to be most useful to faithfully represent the genotypic data in fewer dimensions (2 dimensions = 0.009% stress). In this reduced space, a model specifying three genotypic clusters received the highest support (BIC= 1668.27, next best model ΔBIC= 5.2501, four clusters; Figure S32).

Transition between cladogenesis and anagenesis (CA model) Given the phylogeny reconstructed with the concatenated alignment of full sequences per locus, a model with seven genogroups was the best fit to the data (Figure S33). Across all independent runs, all nodes identifying genogroups strongly identified such nodes as transitions between cladogenesis and anagenesis (Fig S33; See values closer to 0 subtending red groups).

Reproductive isolation (RI model) A model specifying three demes received the strongest support based on the ΔK statistic using `STRUCTURE`. Using this deme assignment, we found consistent and strong support for a model specifying three genogroups ($pp = 1.0$, Figure S34), for both the full and reduced datasets (See Methods for details). A model specifying five demes using `MAVERICK` had the highest posterior probability ($pp > 0.99$). Using this deme assignment, we found consistent and strong support for a model specifying five genogroups ($pp = 1.0$, Figure S34), for both the full and reduced datasets. Although all our analyses used to identify genogroups with both deme assignments showed some mixing issues, every replicated analysis consistently supported three and five genogroups, respectively.

Model Selection We filtered our matrix and retained $1,993$ loci to conduct this analysis (Table S9). We compared the four models described above using Bayes Factors. The GC model and both RI models using population assignments based on `STRUCTURE` and `MAVERICK` each identified and assigned the same individuals to the same three genogroups. The GC model and the RI model using `STRUCTURE` required $250,000$ samples to stabilize (as estimated from ESS values for each step), while the CA model and the RI model using population assignments based on `MAVERICK` required more than $500,000$ samples. In total, each analysis used 24 steps. The CA model (seven genogroups) was the top-ranked model with decisive support relative to the two other models[38] (Table S10).

2.4.4 Data Integration

Based on the best fitting models, we assigned each specimen to its corresponding phenogroup and genogroup (Figure S35). In total, we assigned specimens to eight species (See main text, Figure 2). Two of these species matched uniquely one phenogroup to one genogroup (Figure S35); See for example phenogroup 4 - genogroup 3). Therefore, these two species are recognized as 'good species'. Five species belonged to two phenogroups across five genogroups (Figure S35); See for example phenogroup 2 - genogroups 1, 2). These five species are thus recognized as 'phenotypic cryptic species'. Two species belonged to one genogroup across two phenogroups (Figure S35); See for example phenogroup 1, 3 - genogroup 5). These two species are recongnized as 'genetic cryptic species'. The association of phenogroups to genogroups was $GK\tau_{pg} = 0.731$, while the association of genogroups to phenogroups was $GK\tau_{gp} = 0.913$. This indicates that genogroup membership is slightly better predictor of phenogroup membership than phenogroup membership is of genogroup membership (i.e., there are more 'phenotypic cryptic species' in this clade than 'genetic cryptic species').

We examined all the specimens in their phylogenetic and geographic contexts to gain insight into the plausible species identity of the unknown specimens and the nature of species in this clade. Considering

the geographic distribution of the unknown specimens assigned to phenogroup 4, in concert with our extensive field and herbarium work, we speculate that these specimens most likely belong to genogroup 3. Similarly, we speculate that the unknown specimens assigned to genogroup 3 most likely belong to phenogroup 4. Applying the same reasoning, we speculate that the unknown specimens in phenogroups 1 and 5 most likely belong to genogroups 5 and 4, respectively. Likewise, we suggest that the unknown specimens in genogroups 4 and 5 most likely belong to phenogroups 5 and 1, respectively. For the unknown specimens in genogroups 1, 2, and 6, we speculate that these specimens most likely belong to phenogroups 2, and 3. However, we cannot speculate to which genogroups the unknown specimens in phenogroups 2 and 3 belong (See also $GK\tau_{pg}$ above). These uncertainties aside, together our results showed that at the spatial scale of our study all species co-occur in close geographic proximity with at least one other species (Figure S35). Our phylogenetic and geographic results also showed that while the 'good species' are allopatric, they co-occur with other species which are not their closest relatives. By contrast, the 'phenotypic cryptic species' and 'genetic cryptic species' largely co-occur with their closest relatives.

2.4.5 Correspondence between taxonomic species and model-based species

We assigned all specimens to their corresponding taxonomic species, phenogroup, and genogroup. Only specimens assigned to one taxonomic species (*E. schreiteri*) showed perfect correspondence to a single phenogroup and a single genogroup. All other specimens are assigned to phenogroups and genogroups shared across multiple taxonomic species (See main text, Table 4; Table S11).

2.5 Clade IV

2.5.1 Sampling

A total of 91 specimens were included in this clade (Figure S36). We measured phenotypic traits on 84 specimens; 74 were fertile specimens (thus having both leaf and floral traits) and 10 were sterile specimens (thus having leaf traits only). Only fertile specimens were included in downstream phenomic analyses. We collected genomic data for 42 specimens; 25 specimens were fertile, 10 were sterile, and 7 were not avaialable for phenotypic analyses. All 42 specimens were included in downstream genomic analyses except in our model selection analysis (See below). The 91 specimens covered the geographic range of this clade and belonged to two taxonomic species (Figure S37, Table S1)

2.5.2 Phenomics

2.5.2.1 Current state of taxonomic species The 10-cubes defining the taxonomic species that belong to this clade did not overlap in 10-dimensional phenospace. The matching-prediction analysis showed that 0% of the specimens fall inside any 10-cube, with no specimens falling inside their correct 10-cube (Figure S38).

2.5.2.2 Model-based species discovery We found five principal components to be most useful for group discrimination. The Taxonomy[1] and Taxonomy Unaware models, both specifying the same two phenogroups, received the strongest support using the BIC criterion (BIC= 115.0039). We could not use the ICL criterion because the best fit model was not the naive model and `mclust` does not implement the ICL criterion for models with *a priori* classification. Support for the naive model specifying three distinct phenogroups of equal volume, elliptical, and identical orientation was only slightly lower (ΔBIC= 0.8951). Because the difference in BIC scores between the best fit and competing models is not significant,[38] we

present results for both models (Figure S39). As expected, the LRT is consistent with the BIC model selection framework and showed that a model with three distinct phenogroups was a better fit to the data than models with more (or less) phenogroups (p=value < 0.05).

2.5.3 Genomics

2.5.3.1 Sensitivity Tests In total, we recovered 79,865 loci across 42 sequenced individuals. 31,840 loci were present in at least four individuals. The number of loci in the three matrices with different levels of missing data is presented in Table S12. Results from Principal Components Analysis, phylogenetic analysis, and genomic clustering did not differ (or differed minimally) across datasets, suggesting that our data are robust to the amount of missing data (Figures S40, S41, S42). Therefore, we performed all downstream analyses using the smallest data matrix for computational efficiency.

2.5.3.2 Model-based species discovery **Genotypic clusters (GC model)** We found two dimensions to be most useful to faithfully represent the genotypic data in fewer dimensions (2 dimensions = 1.2967% stress). In this reduced space, a model specifying three genotypic clusters received the highest support (BIC= 973.8316, next best model ΔBIC= 3.6340, three clusters; Figure S43).

Transition between cladogenesis and anagenesis (CA model) Given the phylogeny reconstructed with the concatenated alignment of full sequences per locus, a model with six genogroups was the best fit to the data (Figure S44). Across all independent runs, all nodes identifying genogroups strongly identified such nodes as transitions between cladogenesis and anagenesis (Fig S44; See values closer to 0 subtending red groups).

Reproductive isolation (RI model) A model specifying two demes received the strongest support based on the ΔK statistic using `STRUCTURE`. A model specifying three demes using `MAVERICK` had the highest posterior probability ($pp > 0.99$); however, one of these demes never received a majority posterior assignment for any specimen. Because we used a majority posterior assignment to assign specimens to demes, we thus used a model with two demes, which was identical to the model specified using `STRUCTURE`. Therefore, the RI model used the same deme assignment (two demes) with both `STRUCTURE` and `MAVERICK`. Using this deme assignment, we found consistent and strong support for a model specifying two genogroups ($pp = 1.0$, Figure S45), for both the full and reduced datasets (See Methods for details).

Model Selection We filtered our matrix and retained 2,245 loci to conduct this analysis (Table S12). We compared the three models specified above using Bayes Factors. Each model required approximately 250,000 samples to stabilize (as estimated from ESS values for each step) with the exception of the CA model which required more than 500,000 samples to stabilize. In total, each analysis used 24 steps. The CA model (six genogroups) was the top-ranked model with decisive support relative to the other models[38] (Table S13).

2.5.4 Data Integration

Based on the best fitting models, we assigned each specimen to its corresponding phenogroup and genogroup (Figure S46). In total, we assigned specimens to six species (See main text, Figure 2). One species matched uniquely a single phenogroup and a single genogroup (Figure S46; phenogroup 1 - genogroup 3). This species is thus recognized as a 'good species'. The remaining specimens were assigned to a single phenogroup and five genogroups (Figure S46; phenogroup 2 - genogroups 1, 2, 4, 5, and 6). Therefore, these species are collectively recongnized as 'phenotypic cryptic species'. The association of phenogroups to genogroups was $GK\tau_{pg} = 0.214$, while the association of genogroups to phenogroups was $GK\tau_{gp} = 1.0$.

This indicates that genogroup membership is a perfect predictor of phenogroup membership (i.e., there are no 'genetic cryptic species') but phenogroup membership is a poor predictor of genogroup membership (i.e., there are many 'phenotypic cryptic species' in this clade).

We examined all the specimens in their phylogenetic and geographic contexts to gain insight into the plausible species identity of the unknown specimens and the nature of species in this clade. Based on the geographic distribution of the unknown specimens assigned to genogroups 1, 2, 4, and 6, combined with our extensive field and herbarium work, we infer that all these specimens belong to phenogroup 2 (See also $GK\tau_{gp}$ above). Likewise, we suggest that the unknown specimens assigned to phenogroup 1 most likely belong to genogroup 3. However, it is not possible to infer the plausible genogroup assignment for all the unknown specimens in phenogroup 2 (See also $GK\tau_{pg}$ above). This is particularly challenging south of latitude $-10°$ where genogroups 2, 4, 5, and 6 co-occurr and do not display any clear pattern of geographic structure. These uncertainties aside, our results showed that at the spatial scale of our study all species largely co-occur in close geographic proximity with other species. The closely related species in genogroups 1 and 3 are restricted to latitudes north of $-8°$, whereas their closely related species in genogroup 2 co-occurs with the group of related species in genogroups 4, 5, and 6 south of $-10°$. Notably, the closely related 'phenotypic cryptic species' in genogroups 4, 5, and 6 all co-occur locally, which indicates that these species are isolated in sympatry despite sharing phenotypic similarities (Figure S46).

2.5.5 Correspondence between taxonomic species and model-based species

We assigned all specimens to their corresponding taxonomic species, phenogroup, and genogroup. Only specimens assigned to one taxonomic species (*E. polifolia*) showed perfect correspondence to a single phenogroup and a single genogroup. The other specimens, all of which were assigned to the taxonomic species *E. myrtilloides*, shared a single phenogroup across multiple genogroups (See main text, Table 4; Table S14).

2.6 Clade V

2.6.1 Sampling

A total of 257 specimens were included in this clade (Figure S47). We measured phenotypic traits on 256 specimens; 216 were fertile specimens (thus having both leaf and floral traits) and 40 were sterile specimens (thus having leaf traits only). Only fertile specimens were included in downstream phenomic analyses. We collected genomic data for 109 specimens; 78 specimens were fertile, 30 were sterile, and 1 were not available for phenotypic analyses. All 109 specimens were included in downstream genomic analyses except in our model selection analysis (See below). The 257 specimens covered the geographic range of this clade and belonged to seven taxonomic species (Figure S48, Table S1)

2.6.2 Phenomics

2.6.2.1 Current state of taxonomic species The 10-cubes based defining the taxonomic species that belong to this clade did not overlap in 10-dimensional phenospace. The matching-prediction analysis showed that 0% of the specimens fall inside any 10-cube, with no specimens falling inside their correct 10-cube (Figure S49).

2.6.2.2 Model-based species discovery We found five principal components to be most useful for group discrimination. The naive model specifying eight distinct ellipsoidal phenogroups of equal

volume, shape, and orientation received the strongest support using both the BIC and ICL criteria (BIC= 516.723, ICL= 552.033). Support for alternative models was considerably lower, including the Taxonomy model[1] specifying seven phenogroups (ΔBIC= 274.73) and the Taxonomy Unaware model specifying four phenogroups (ΔBIC= 131.315) (Figure S50). A LRT further corroborated that a model with eight phenogroups of equal shape and orientation was a better fit to the data than models with more phenogroups (p-value < 0.05)

2.6.3 Genomics

2.6.3.1 Sensitivity Tests In total, we recovered 133,181 loci across 109 sequenced individuals. 50,898 loci were present in at least four individuals. The number of loci in the three matrices with different levels of missing data is presented in Table S15). Results from Principal Components Analysis, phylogenetic analysis, and genomic clustering did not differ (or differed minimally) across datasets, suggesting that our data are robust to the amount of missing data (Figures S51, S52, S53). Therefore, we performed all downstream analyses using the smallest data matrix for computational efficiency.

2.6.3.2 Model-based species discovery **Genotypic clusters (GC model)** We found two dimensions to be most useful to faithfully represent the genotypic data in fewer dimensions (2 dimensions = 15.6% stress). In this reduced space, a model specifying six genotypic clusters received the highest support (BIC= 583.3511, next best model ΔBIC= 1.7043, six clusters; Figure S54).

Transition between cladogenesis and anagenesis (CA model) Given the phylogeny reconstructed with the concatenated alignment of full sequences per locus, a model with ten genogroups was the best fit to the data (Figure S55). Across all independent runs, all nodes identifying genogroups strongly identified such nodes as transitions between cladogenesis and anagenesis (Fig S55; See values closer to 0 subtending red groups).

Reproductive isolation (RI model) A model specifying two demes received the strongest support based on the ΔK statistic using `STRUCTURE` (Figure S56). Using this deme assignment, we found consistent support for a model specifying two genogroups, for both the full and reduced datasets despite some mixing issues ($pp = 1.0$, Figure S56). A model specifying four demes using `MAVERICK` had the highest posterior probability ($pp > 0.99$); however, one of these demes never received a majority posterior assignment for any specimen. Because we used a majority posterior assignment to assign specimens to demes, we used a model with three demes. Using this deme assignment, we found consistent support for a model specifying three genogroups ($pp = 1.0$, Figure S56), for both the full and reduced datasets.

Model Selection We filtered our matrix and retained 742 loci to conduct this analysis (Table S15). We compared the four models specified above using Bayes Factors. The RI models required 250,000 samples to stabilize (as estimated from ESS values for each step), while the GC and CA models required 500,000. In total, each analysis used 24 steps. The CA model (ten genogroups) was the top-ranked model with decisive support relative to the other models[38] (Table S16).

2.6.4 Data Integration

Based on the best fitting models, we assigned each specimen to its corresponding phenogroup and genogroup (Figure S57). In total, we assigned specimens to 20 species (See main text, Figure 2). No species matched uniquely one phenogroup to one genogroup, showing there are no 'good species' in this clade (Figure S57). Specimens assigned to six of the eight phenogroups were assigned to multiple genogroups (Figure S57; See for example phenogroup 2 and genogroups 4, 6, 9, and 10). This indicates

there are multiple 'phenotypic cryptic species' in this clade. Similarly, specimens assigned to six of the ten genogroups were assigned to different phenogroups (Figure S57; See for example genogroup 9 and phenogroups 2, 3, 4, and 7). This in turn indicates that several of the 'phenotypic cryptic species' in this clade are also 'genetic cryptic species'. The measure of association between phenogroups and genogroups reflects this result. The association between phenogroups and genogroups was $GK\tau_{pg} = 0.488$, while the association between genogroups and phenogroups was $GK\tau_{gp} = 0.467$. This shows that genogroup membership is a poor predictor of phenogroup membership and that phenogroup membership is also poor predictor of genogroup membership. This is a consequence of the large number of 'phenotypic cryptic' and 'genetic cryptic' species in this clade.

We examined all the specimens in their phylogenetic and geographic contexts to gain insight into the plausible species identity of the unknown specimens and the nature of species in this clade. Because all phenogroups and genogroups showed extensive overlap and limited geographic strucuture, it is difficult to infer the likely assignment of the unknown specimens based on geographic information alone (Figure S57). In addition, the predictability of each grouping category to infer the other category is extremely low (See estimates of $GK\tau$ above). Nonetheless, based on our extensive field and herbarium work, we speculate that the unknown specimens in genogroup 1 most likely belong to phenogroups 4 and 8, and the unknown specimens in genogroup 2 most likely belong to phenogroup 3. We also speculate that the unknown specimens in genogroup 5 likely belong to phenogroup 6. There were no other clear cases of plausible phenogroup assignment based on genogroup information. Inferring genogroup based on phenogroup membership was particularly difficult, with very few exceptions. For instance, we could only speculate that the unknown specimens in phenogroup 1 likely belong to genogroup 3 (Figure S57). Our phylogenetic and geographic results also showed that three genogroups are widespread across latitude and elevation (genogroups 1, 9, and 10) while the remaining genogroups are seemingly less widespread (Figure S57). Notably, each widespread genogroup is part of different 'genetic cryptic species' (i.e., one genogroup across multiple phenogroups). Because the specimens assigned to the different phenogroups within each of the widespread genogroups are closely related and co-occur locally in sympatry, our results are consistent with the intriguing possibility that these 'genetic cryptic species' correspond to phenotypically distinct species interconnected by gene exchange–so-called syngameons[40,41] (Figure S57; See for example genogroup 9). Further sampling and proper population genetic models are required to test this hypothesis with increased rigor. Our results also showed that genogroups 5, 6, and 8 are a group of closely related 'phenotypic cryptic species' which co-occur in broad sympatry. Specimens assigned to phenogroups 2 and 3 and to genogroup 7 are nested within or sister to the genogroups 5, 6, and 8. Because phenogroups 2 and 3 are common in the widespread genogroup 10, we speculate the specimens belonging to these phenogroups which are nested or sister to the group of 'phenotypic cryptic species' likely represent cases of local hybridization between different genogroups (Figure S57). Clearly, extensive sampling is necessary in the region of sympatry to discern the nature of these plant species.

2.6.5 Correspondence between taxonomic species and model-based species

We assigned all specimens to their corresponding taxonomic species, phenogroup, and genogroup. There were no cases of perfect correspondence between a taxonomic species and a single phenogroup and genogroup. Instead, all specimens were assigned to phenogroups and genogroups that were shared across multiple taxonomic species (See main text, Table 4; Table S17).

2.7 Clade VI

2.7.1 Sampling

A total of 250 specimens were included in this clade (Figure S58). We measured phenotypic traits on 243 specimens; 195 were fertile specimens (thus having both leaf and floral traits) and 48 were sterile specimens (thus having leaf traits only). Only fertile specimens were included in downstream phenomic analyses. We collected genomic data for 82 specimens; 32 specimens were fertile, 43 were sterile, and 7 were not available for phenotypic analyses. All 82 specimens were included in downstream genomic analyses except in our model selection analysis (See below). The 250 specimens covered the geographic range of this clade and belonged to ten taxonomic species (Figure S59, Table S1)

2.7.2 Phenomics

2.7.2.1 Current state of taxonomic species The 10-cubes defining the taxonomic species belonging to this clade did not overlap in 10-dimensional phenospace. The matching-prediction analysis showed that only 0% of the specimens fall inside any 10-cube, with no specimens falling inside their correct 10-cube (Figure S60).

2.7.2.2 Model-based species discovery We found nine principal components to be most useful for group discrimination. The naive model specifying eight phenogroups of equal shape and orientation received the strongest support using both the BIC and ICL criteria (BIC= 231.247, ICL= 224.2705). Support for alternative models was considerably lower, including the Taxonomy model[1] specifying ten phenogroups (ΔBIC= 749.011) and the Taxonomy Unaware model specifying ten phenogroups (ΔBIC= 30.944) (Figure S61). A LRT further corroborated that a model with eight phenogroups of equal shape and orientation was a better fit to the data than models with more phenogroups (p-value < 0.05)

2.7.3 Genomics

2.7.3.1 Sensitivity Tests In total, we recovered $133,123$ loci across 82 sequenced individuals. $49,105$ loci were present in at least four individuals. The number of loci in the three matrices with different levels of missing data is presented in Table S18. Results from Principal Components Analysis, phylogenetic analysis, and genomic clustering did not differ (or differed minimally) across datasets, suggesting that our data are robust to the amount of missing data (Figures S62, S63, S64). Therefore, we performed all downstream analyses using the smallest data matrix for computational efficiency.

2.7.3.2 Model-based species discovery **Genotypic clusters (GC model)** We found two dimensions to be most useful to faithfully represent the genotypic data in fewer dimensions (2 dimensions = 9.34% stress). In this reduced space, a model specifying seven genotypic clusters received the highest support (BIC= 1061.975, next best model ΔBIC= 1.6393, seven clusters; Figure S65).

Transition between cladogenesis and anagenesis (CA model) Given the phylogeny reconstructed with the concatenated alignment of full sequences per locus, a model with eleven genogroups was the best fit to the data (Figure S66). Across all independent runs, all nodes identifying genogroups strongly identified such nodes as transitions between cladogenesis and anagenesis (Fig S66; See values closer to 0 subtending red groups).

Reproductive isolation (RI model) A model specifying four demes received the strongest support based on the ΔK statistic using `STRUCTURE`. A model specifying five demes using `MAVERICK` had the highest posterior probability ($pp > 0.99$); however, one of these demes never received a majority posterior assignment for any specimen. Because we used a majority posterior assignment to assign specimens to demes, we thus used a model with four demes, which was identical to the model specified using `STRUCTURE`. Therefore, the RI model used the same deme assignment (four demes) with both `STRUCTURE` and `MAVERICK`. Using this deme assignment, we found consistent and strong support for a model specifying four genogroups ($pp = 1.0$, Figure S67), for both the full and reduced datasets (See Methods for details).

Model Selection We filtered our matrix and retained 915 loci to conduct this analysis (Table S18). We compared the three models specified above using Bayes Factors. Each model required approximately 250,000 samples to stabilize (as estimated from ESS values for each step) with the exception of the CA model which required more than 500,000 samples to stabilize. In total, each analysis used 24 steps. The CA model (eleven genogroups) was the top-ranked model with decisive support relative to the other models[38] (Table S19).

2.7.4 Data Integration

Based on the best fitting models, we assigned each specimen to its corresponding phenogroup and genogroup (Figure S68). In total, we assigned specimens to 10 species (See main text, Figure 2). Three species matched uniquely a single phenogroup to a single genogroup (Figure S68; See for example phenogroup 2 - genogroup 5). Therefore, these species are recognized as 'good species'. Six species belonged to two phenogroups across five genogroups (Figure S68; See for example phenogroup 1 - genogroups 2, 4, 10, 11). These species are collectively recongnized as 'phenotypic cryptic species'. Four species belonged to two genogroups across two phenogroups (Figure S68; See for example genogroup 4 - phenogroups 1, 4). These species are collectively recognized as 'genotypic cryptic species'. We also discovered two phenogroups for which we had no genomic data (Figure S68; See phenogroups 3 and 6), and three genogroups for which we had no phenotypic data (Figure S68; See genogroups 6, 8, and 9). The association of phenogroups to genogroups was $GK\tau_{pg} = 0.733$, while the association of genogroups to phenogroups was $GK\tau_{gp} = 0.845$. This indicates that genogroup membership is a slightly better predictor of phenogroup membership than phenogroup membership is of genogroup membership. In other words, this shows there are more 'phenotypic cryptic species' in this clade than 'genetic cryptic species'.

We examined all the specimens in their phylogenetic and geographic contexts to gain insight into the plausible species identity of the unknown specimens and the nature of species in this clade. In contrast to other clades, phenogroups and genogroups in clade VI show an overall strong pattern of geographic structure (Figure S68). Considering the geographic distribution of the unknown specimens assigned to genogroup 4, in combination with our extensive field and herbarium work, we speculate that these specimens most likely belong to phenogroups 1 or 4. In addition, given the narrow longitudinal range of genogroup 4, we further speculate that the unknown specimens assigned to phenogroups 1 or 4 that occur around longitude $-65°$ likely belong to genogroup 4. These assignments would indicate that the closely related phenogroups 1 and 4 within genogroup 4 segregate largely along elevation (Figure S68). Similarly, genogroups 1, 2, and 3 have a narrow longitudinal range with phenogroup 7 largely restricted to lower elevations (Figure S68). Therefore, we suggest that the unknown specimens in genogroup 1 most likely belong to phenogroup 7, and the unknown specimens in phenogroup 7 likely belong to genogroup 1. By contrast, owing to our limited sampling of phenotypic data for specimens east of longitude $-60°$ as well as the broad sympatry of all species in this geographic region, it is difficult to infer the possible phenogroup membership of the unknown specimens in genogroups 5, 6, 7, 8, and 9. Given our extensive field and herbarium work, we can only speculate that the unknown specimens in genogroups 10 and 11 most likely belong to phenogroup 1. These uncertainties aside, together our results showed that at the

spatial scale of our study, groups of species co-occur in close geographic proximity with at least one other closely related species (Figure S68). Our phylogenetic and geographic results also showed that while the 'good species' are allopatric with one another, they co-occur in broad sympatry with other species which are not their closest relatives. The species in one group of 'phenotypic cryptic species' (phenogroup 1) are not all closely related and are allopatric, suggesting convergent evolution in phenotypes, perhaps driven by niche conservatism.[39] Both groups of 'genetic cryptic species' (genogroups 2 and 4) co-occur in broad sympatry, yet their respective phenogroups seeminlgy segregate along elevation (Figure S68; See genogroup 4 - phenogroups 1, 4). This pattern is consistent with the hypothesis of ecological speciation along elevation gradients, but extensive sampling and modeling is needed to test this hypothesis.

2.7.5 Correspondence between taxonomic species and model-based species

We assigned all specimens to their corresponding taxonomic species, phenogroup, and genogroup. Only specimens assigned to two taxonomic species (*E. bifida* and *E. farinacea*; Figure S68; See phenogroup 2 - genogroup 5, and phenogroup 5 - genogroup 7, respectively) showed perfect correspondence to a single phenogroup and a single genogroup. All other specimens were assigned to phenogroups and genogroups shared across multiple taxonomic species (See main text, Table 4; Table S20).

3 Tables

3.0.1 Table S1: Taxon Sampling

Table S1: Specimens used in this study. Vouchers are deposited in different herbaria (See https://github.com/zapata-lab/ms_nature_of_species for further details)

SpecimenID	Taxonomic species	Taxonomy unaware group	CladeID	Latitude	Longitude	Elevation	Phenomics	Genomics	Sampled for BFD	Phenogroup	Genogroup
AG22671	micrantha	16	I	-5.82	-79.52	2000	yes	no		1	unk
AS15247	micrantha	16	I	-7.47	-78.78	2100	yes	no		1	unk
AS8791	micrantha	16	I	-7.08	-79.05	1850	yes	no		1	unk
AS9297	micrantha	16	I	-7.38	-78.90	2500	yes	no		1	unk
BK493	micrantha	16	I	-4.68	-79.72	1900	yes	no		1	unk
FZ241	micrantha	16	I	-7.07	-79.05	2124	yes	yes	yes	1	3
FZ242	micrantha	16	I	-7.07	-79.05	2124	yes	yes	no	1	3
FZ243	micrantha	16	I	-7.07	-79.05	2118	yes	yes	no	1	3
FZ245A	micrantha	16	I	-7.07	-79.05	2118	yes	yes	yes	1	3
MD6482	micrantha	16	I	-7.42	-78.72	1900	yes	no		1	unk
PJ98	micrantha	16	I	-4.09	-79.93	2400	yes	no		1	unk
AJ1598	millegrana	16	I	-18.33	-64.48	1385	yes	no		2	unk
AS148	millegrana	16	I	-22.30	-64.68	1850	yes	no		2	unk
AS34715	millegrana	16	I	-23.08	-64.87	2200	yes	no		2	unk
FZU10219A	millegrana	16	I	-24.23	-65.08	1650	yes	yes	yes	2	1
FZU10362A	millegrana	16	I	-22.31	-64.71	1625	yes	yes	yes	2	1
FZU10398A	millegrana	16	I	-21.44	-64.38	2360	yes	yes	no	2	1
FZU10398C	millegrana	16	I	-21.44	-64.38	2360	yes	no		2	unk
FZU10413	millegrana	16	I	-21.46	-64.14	1160	yes	yes	yes	2	1
IV108	millegrana	16	I	-18.42	-64.12	1900	yes	no		2	unk
IV940	millegrana	16	I	-18.52	-64.09	2050	yes	no		2	unk
JG492	millegrana	16	I	-20.73	-64.50	1850	yes	no		2	unk
JS10229	millegrana	16	I	-21.90	-64.68	2000	yes	no		2	unk
JS10315	millegrana	16	I	-21.48	-64.33	2050	yes	no		2	unk
JS17904	millegrana	16	I	-17.70	-64.87	2950	yes	no		2	unk
JW11737	millegrana	16	I	-18.20	-64.98	2000	yes	no		2	unk
JW8314	millegrana	16	I	-21.88	-64.90	2100	yes	no		2	unk
JW8984	millegrana	16	I	-18.85	-65.22	2500	yes	no		2	unk
MBL1130	millegrana	16	I				yes	no		2	unk
MS7323	millegrana	16	I	-21.76	-64.31	1696	yes	no		2	unk
RK3415	millegrana	16	I	-24.17	-65.23	1350	yes	no		2	unk
RR71	millegrana	16	I	-18.55	-64.63	1228	yes	no		2	unk
WE25045	millegrana	16	I	-17.98	-65.60	2700	yes	no		2	unk
FZ289	millegrana	16	I	-17.84	-65.46	2756		yes	yes	unk	2
FZ290	millegrana	16	I	-17.84	-65.46	2756		yes	no	unk	2
FZ291	millegrana	16	I	-17.85	-65.46	2752		yes	no	unk	2
FZ311	millegrana	16	I	-17.75	-64.93	2663		yes	no	unk	2
FZU10362C	millegrana		I	-22.31	-64.71	1625		yes	no	unk	1
FZU10398D	millegrana		I	-21.44	-64.38	2360		yes	no	unk	1
AG75032	pendula	20	II	-4.58	-79.67	2300	yes	no		1	unk
AH21395	pendula	20	II	-4.00	-79.30	2000	yes	no		1	unk
AS8172	herrerae	20	II	-5.24	-79.45	1800	yes	no		1	unk
FZ190	herrerae	20	II	-13.47	-72.50	2820	yes	yes	yes	1	1
FZ206	pendula	20	II	-6.87	-78.11	3100	yes	yes	yes	1	3
FZ207	pendula	20	II	-6.86	-78.12	3019	yes	yes	yes	1	3
JB2640	pendula	20	II	-2.46	-79.00	3000	yes	no		1	unk
WC4029	pendula	20	II	-2.38	-78.95	2500	yes	no		1	unk
WG5539	herrerae	20	II	-13.60	-72.90	3450	yes	no		1	unk
FW34334	pendula	20	II	-9.78	-76.08	2200	yes	no		2	unk
IH6604	pendula	20	II	-13.07	-72.37	2420	yes	no		2	unk
JA1012	pendula	20	II	-5.88	-77.94	2150	yes	no		2	unk
JC9871	pendula	20	II	6.75	-72.70	2100	yes	no		2	unk
JH714	pendula	20	II	6.34	-72.68	2000	yes	no		2	unk
JL5220	pendula	20	II	9.31	-70.18	1650	yes	no		2	unk
JS104901	pendula	20	II	9.82	-70.07	1700	yes	no		2	unk
LA4940	pendula	20	II	9.92	-69.38	1300	yes	no		2	unk
RL13025	pendula	20	II	9.23	-70.25	1500	yes	no		2	unk
AN414	pendula	20	II	5.83	-72.97	2400	yes	yes	yes	3	2
CD9872	pendula	20	II	-5.67	-79.32	2000	yes	no		3	unk
DS10848	pendula	20	II	-9.53	-77.85	1900	yes	no		3	unk
FZ186	herrerae	20	II	-13.47	-72.50	2768	yes	yes	yes	3	1

FZ228	pendula	20	II	-7.27	-78.51	2540	yes	yes	yes	3	4
FZ229	pendula	20	II	-7.27	-78.51	2540	yes	no		3	unk
FZ230	pendula	20	II	-7.27	-78.51	2540	yes	yes	no	3	4
FZ231	pendula	20	II	-7.27	-78.51	2540	yes	yes	no	3	4
FZ232	pendula	20	II	-7.27	-78.51	2540	yes	no		3	unk
FZ233	pendula	20	II	-7.29	-78.51	2223	yes	no		3	unk
FZ234	pendula	20	II	-7.32	-78.80	2007	yes	no		3	unk
FZ235	pendula	20	II	-7.32	-78.80	2007	yes	yes	no	3	4
FZ244	pendula	20	II	-7.07	-79.05	2118	yes	yes	yes	3	4
MW5820	herrerae	20	II	-12.32	-74.82	2885	yes	no		3	unk
MW7204	pendula	20	II	-12.87	-76.06	1350	yes	no		3	unk
FZ187	herrerae	20	II	-13.47	-72.50	2794		yes	no	unk	1
FZ188	herrerae	20	II	-13.47	-72.50	2805		yes	no	unk	1
FZ189	herrerae	20	II	-13.47	-72.50	2810		yes	no	unk	1
FZ227	pendula	20	II	-6.82	-77.95	2306		yes	no	unk	3
FZ90	pendula	20	II	4.89	-74.00	2660		yes	yes	unk	2
BH6180	paniculata	19	III	8.63	-82.12	1200	yes	no		1	unk
CR1019	paniculata	19	III	3.52	-76.15	2000	yes	no		1	unk
DN14917	paniculata	19	III	-3.97	-79.07	1900	yes	no		1	unk
EK20578	paniculata	19	III	7.38	-72.56	2800	yes	no		1	unk
FZ200	paniculata	19	III	-13.35	-71.62	3492	yes	yes	no	1	5
FZ203	paniculata	19	III	-13.18	-71.60	3186	yes	yes	yes	1	5
FZ204	paniculata	19	III	-13.18	-71.60	3186	yes	yes	no	1	5
FZ267	paniculata	19	III	-16.31	-67.81	2984	yes	yes	no	1	5
FZ269	paniculata	19	III	-16.32	-67.81	2950	yes	yes	no	1	5
FZ270	paniculata	19	III	-16.29	-67.81	2790	yes	no		1	unk
FZ272	paniculata	19	III	-16.28	-67.79	2432	yes	yes	no	1	5
GC1765	paniculata	19	III	-12.87	-72.53	2150	yes	no		1	unk
HV4434	paniculata	19	III	0.82	-78.13	1860	yes	no		1	unk
Hv8197	paniculata	19	III	8.25	-71.55	2250	yes	no		1	unk
JP2912	paniculata	19	III	-5.09	-78.84	2002	yes	no		1	unk
JS10701	paniculata	19	III	-16.30	-67.82	3000	yes	no		1	unk
JS18731	paniculata	19	III	-16.12	-68.07	2200	yes	no		1	unk
JT2968	paniculata	19	III	11.11	-74.03	2300	yes	no		1	unk
JW11465	paniculata	19	III	-17.79	-64.72	2200	yes	yes	no	1	5
KV5689	paniculata	19	III	-12.47	-72.00	2831	yes	no		1	unk
KY4092	paniculata	19	III	-7.00	-77.00	2300	yes	no		1	unk
LU4163	paniculata	19	III	5.83	-75.53	2400	yes	no		1	unk
MC1182	paniculata	19	III	9.42	-70.33	2100	yes	no		1	unk
MD2620	paniculata	19	III	-9.55	-75.98	1950	yes	no		1	unk
MW2000_891	paniculata	19	III	-6.21	-77.72	2200	yes	no		1	unk
PA6612	paniculata	19	III	-17.22	-65.80	2600	yes	no		1	unk
RR3379	paniculata	19	III	-10.62	-75.30	2300	yes	no		1	unk
RV25391	paniculata	19	III	-6.38	-77.51	1500	yes	no		1	unk
AG23305	resinosa	24	III	-14.47	-73.24	3220	yes	no		2	unk
AS10370	piurensis	24	III	-7.36	-78.90	3300	yes	no		2	unk
AS3821	piurensis	24	III	-7.37	-79.01	3230	yes	no		2	unk
CD5089	resinosa	24	III	-17.68	-65.10	2890	yes	no		2	unk
CO527	resinosa	24	III	-11.72	-75.11	3200	yes	no		2	unk
CP1050	resinosa	24	III	-2.86	-78.94	2600	yes	no		2	unk
DS10236	resinosa	24	III	-9.63	-77.20	3450	yes	no		2	unk
DS10599	resinosa	24	III	-9.02	-77.05	3600	yes	no		2	unk
DS10955	resinosa	24	III	-9.45	-77.85	2879	yes	no		2	unk
DS3356	resinosa	24	III	-8.33	-78.08	3400	yes	no		2	unk
EB572	resinosa	24	III	-21.48	-65.03	2800	yes	no		2	unk
EBB6926	resinosa	24	III	-13.38	-73.88	3300	yes	no		2	unk
FP14405	resinosa	24	III	-11.43	-76.61	3200	yes	no		2	unk
FW5273	resinosa	24	III	-9.81	-75.88	2200	yes	no		2	unk
FZ163	resinosa	24	III	-13.50	-71.98	3265	yes	no		2	unk
FZ165	resinosa	24	III	-13.50	-71.98	3698	yes	no		2	unk
FZ167	resinosa	24	III	-13.54	-71.94	3317	yes	yes	no	2	1
FZ174	resinosa	24	III	-14.08	-71.37	3553	yes	yes	yes	2	1
FZ180	resinosa	24	III	-13.16	-72.28	3694	yes	yes	no	2	1
FZ182	resinosa	24	III	-13.18	-72.29	3566	yes	yes	no	2	1
FZ183	resinosa	24	III	-13.18	-72.29	3566	yes	no		2	unk
FZ191	resinosa	24	III	-13.30	-71.60	2942	yes	no		2	unk
FZ205	resinosa	24	III	-13.21	-71.63	3383	yes	yes	no	2	1
FZ211	piurensis	24	III	-6.78	-77.94	2818	yes	yes	no	2	1
FZ236	piurensis	24	III	-7.33	-78.81	2709	yes	yes	no	2	1
FZ239	piurensis	24	III	-7.33	-78.81	2709	yes	no		2	unk
FZ240	piurensis	24	III	-7.33	-78.81	2709	yes	yes	yes	2	1
FZ287	resinosa	24	III	-17.84	-65.45	2800	yes	no		2	unk
FZ310	resinosa	24	III	-17.83	-64.78	3045	yes	yes	yes	2	2
HS10647	resinosa	24	III	-13.60	-72.85	3000	yes	no		2	unk

JS15148	resinosa	24	III	-16.57	-67.75	3500	yes	no		2	unk
JS15923	resinosa	24	III	-17.67	-65.15	3200	yes	no		2	unk
JW7805	resinosa	24	III	-19.15	-65.33	2900	yes	yes	yes	2	2
MC4122	resinosa	24	III	-18.98	-65.37	3200	yes	no		2	unk
ML35050	resinosa	24	III	-16.92	-67.17	3000	yes	no		2	unk
ML37126	resinosa	24	III	-16.97	-67.22	3350	yes	no		2	unk
MM2032	resinosa	24	III	-17.95	-65.32	2250	yes	no		2	unk
NA763	resinosa	24	III	-17.58	-66.27	2860	yes	no		2	unk
NR751	resinosa	24	III	-17.31	-66.30	3020	yes	no		2	unk
SL123	piurensis	24	III	-8.00	-78.65	2500	yes	no		2	unk
AC13359	paniculata	19	III	7.53	-72.45	2250	yes	no		3	unk
AG48084	paniculata	19	III	3.53	-76.60	2250	yes	no		3	unk
AJ3357	reticulata	25	III	-17.81	-64.63	2400	yes	no		3	unk
AL360	reticulata	25	III	-19.81	-63.72	1540	yes	yes	yes	3	6
AS14179	paniculata	19	III	-7.07	-77.92	2200	yes	no		3	unk
BH18666	paniculata	19	III	9.48	-83.68	2000	yes	no		3	unk
BR7415	paniculata	19	III	10.22	-73.00	2470	yes	no		3	unk
CD9839	paniculata	19	III	-6.52	-79.15	2200	yes	no		3	unk
DB56	paniculata	19	III	10.35	-67.04	1168	yes	no		3	unk
EK17141	paniculata	19	III	7.37	-72.91	2500	yes	no		3	unk
EP5779	paniculata	19	III	2.19	-76.70	1800	yes	no		3	unk
FZ245	paniculata	19	III	-5.37	-79.58	2045	yes	yes	yes	3	7
FZ246	paniculata	19	III	-5.37	-79.58	2045	yes	no		3	unk
FZ247	paniculata	19	III	-5.37	-79.58	2068	yes	yes	yes	3	7
FZ248	paniculata	19	III	-5.37	-79.58	2068	yes	no		3	unk
FZ254	paniculata	19	III	-5.38	-79.57	2485	yes	no		3	unk
FZ271	paniculata	19	III	-16.28	-67.79	2432	yes	yes	yes	3	5
FZ299	reticulata	25	III	-18.18	-63.84	1858	yes	no		3	unk
GD19829	paniculata	19	III	10.10	-64.10	2300	yes	no		3	unk
GH2022	reticulata	25	III	-18.19	-63.73	1300	yes	no		3	unk
HG12283	paniculata	19	III	4.88	-75.13	2500	yes	no		3	unk
HS1748	paniculata	19	III	11.10	-74.03	2000	yes	no		3	unk
Hv_8023	paniculata	19	III	9.67	-70.42	1750	yes	no		3	unk
IS4929	paniculata	19	III	-6.57	-78.80	2150	yes	no		3	unk
IV1724	reticulata	25	III	-18.52	-64.10	2050	yes	no		3	unk
IV745	reticulata	25	III	-18.12	-63.62	1450	yes	no		3	unk
JC28192	paniculata	19	III	9.32	-70.33	2300	yes	no		3	unk
JS127754	paniculata	19	III	10.45	-67.35	2100	yes	no		3	unk
JS8203	reticulata	25	III	-18.17	-63.66	1500	yes	no		3	unk
LH18441	paniculata	19	III	-0.37	-78.80	1800	yes	no		3	unk
MS4032	reticulata	25	III	-17.12	-63.62	1300	yes	no		3	unk
MS4946	reticulata	25	III	-18.08	-63.92	1880	yes	no		3	unk
PJ169	paniculata	19	III	-4.06	-79.89	2700	yes	no		3	unk
RB19296	reticulata	25	III	-18.75	-63.83	2100	yes	no		3	unk
RK541	paniculata	19	III	8.87	-70.69	1650	yes	no		3	unk
SB9811	reticulata	25	III	-19.81	-63.72	1600	yes	no		3	unk
SY359	paniculata	19	III	2.34	-76.49	2500	yes	no		3	unk
TC21834	paniculata	19	III	10.53	-66.88	2000	yes	no		3	unk
VZ2355	paniculata	19	III	-0.22	-78.80	2100	yes	no		3	unk
AN394	paniculata	19	III	4.90	-74.12	2700	yes	no		4	unk
EK17990	discolor	19	III	7.33	-72.91	2500	yes	no		4	unk
EK18727	discolor	19	III	7.37	-72.91	3300	yes	no		4	unk
FZ83	discolor	19	III	4.96	-74.16	2776	yes	yes	yes	4	3
FZ84	discolor	19	III	4.98	-74.15	2776	yes	yes	no	4	3
GL2651	paniculata	19	III	-3.47	-79.60	2950	yes	no		4	unk
JC13460	discolor	19	III	7.23	-72.45	2880	yes	no		4	unk
JH1146	paniculata	19	III	-4.35	-79.16	2600	yes	no		4	unk
JW594	paniculata	19	III	-6.22	-77.82	2200	yes	no		4	unk
PH6683	paniculata	19	III	-4.65	-79.73	2800	yes	no		4	unk
FV7657	schreiteri	29	III	-24.88	-65.70	1750	yes	no		5	unk
FZ292	schreiteri	29	III	-17.85	-65.45	2724	yes	no		5	unk
FZ312	schreiteri	29	III	-17.74	-64.96	2814	yes	yes	yes	5	4
FZ313	schreiteri	29	III	-17.74	-64.96	2885	yes	yes	no	5	4
FZ314	schreiteri	29	III	-17.74	-64.97	2954	yes	yes	yes	5	4
JS10947	schreiteri	29	III	-21.42	-64.28	1850	yes	no		5	unk
JS8624	schreiteri	29	III	-17.73	-65.16	2800	yes	no		5	unk
LN3807	schreiteri	29	III	-24.71	-65.50	1700	yes	no		5	unk
MC5281	schreiteri	29	III	-19.05	-65.20	2700	yes	no		5	unk
NL58	schreiteri	29	III	-17.86	-64.63	1600	yes	yes	no	5	4
SB9352	schreiteri	29	III	-19.35	-64.25	2400	yes	no		5	unk
WB6793	schreiteri	29	III	-17.33	-66.13	2800	yes	no		5	unk
WR1529	schreiteri	29	III	-13.52	-72.57	2800	yes	no		5	unk
FZ128	discolor		III	5.89	-73.06	3081		yes	yes	unk	3
FZ129	discolor		III	5.90	-73.06	3177		yes	no	unk	3
FZ164	resinosa	24	III	-13.49	-71.99	3776		yes	no	unk	1

FZ202	paniculata	19	III	-13.18	-71.60	3186		yes	no	unk	5
FZ208	piurensis	24	III	-6.78	-77.94	2818		yes	no	unk	1
FZ210	piurensis	24	III	-6.78	-77.94	2818		yes	no	unk	1
FZ237	piurensis	24	III	-7.33	-78.81	2709		yes	no	unk	1
FZ238	piurensis	24	III	-7.33	-78.81	2709		yes	no	unk	1
FZ266	paniculata	19	III	-16.30	-67.80	3020		yes	no	unk	5
FZ277	resinosa	24	III	-17.79	-65.49	3352		yes	no	unk	2
FZ283	schreiteri	29	III	-17.84	-65.45	2876		yes	no	unk	4
FZ284	schreiteri	29	III	-17.84	-65.45	2876		yes	no	unk	4
FZ286	schreiteri	29	III	-17.84	-65.45	2757		yes	no	unk	4
FZ293	reticulata	25	III	-18.17	-63.84	1707		yes	no	unk	6
FZ294	reticulata	25	III	-18.18	-63.84	1701		yes	no	unk	6
FZ295	reticulata	25	III	-18.18	-63.82	1885		yes	no	unk	6
FZ296	reticulata	25	III	-18.18	-63.82	1868		yes	no	unk	6
FZ297	reticulata	25	III	-18.18	-63.83	1701		yes	no	unk	6
FZ298	reticulata	25	III	-18.18	-63.82	1941		yes	no	unk	6
FZ300	reticulata	25	III	-18.18	-63.84	1860		yes	yes	unk	6
FZ301	reticulata	25	III	-18.18	-63.84	1862		yes	no	unk	6
FZ315	resinosa	24	III	-17.75	-65.02	3024		yes	no	unk	2
FZ85	discolor		III	4.98	-74.20	3160		yes	no	unk	3
FZU10132A	schreiteri	29	III	-24.89	-65.68	1625		yes	no	unk	4
FZU10272	schreiteri	29	III	-23.65	-64.94	1670		yes	yes	unk	4
FZU10403	schreiteri	29	III	-21.46	-64.34	2050		yes	no	unk	4
AJ2794	reticulata	25	III	-17.86	-64.36	2400		no		unk	unk
DN15369	paniculata	19	III	-4.48	-79.13	2570		no		unk	unk
FZ162	resinosa	24	III	-13.50	-71.98	3629		no		unk	unk
FZ166	resinosa	24	III	-13.55	-71.94	3350		no		unk	unk
FZ173	resinosa	24	III	-14.08	-71.37	3553		no		unk	unk
FZ175	resinosa	24	III	-14.08	-71.37	3553		no		unk	unk
FZ185	resinosa	24	III	-13.20	-72.30	3212		no		unk	unk
FZ209	piurensis	24	III	-6.78	-77.94	2818		no		unk	unk
FZ268	paniculata	19	III	-16.31	-67.81	2970		no		unk	unk
FZ278	resinosa	24	III	-17.79	-65.49	3352		no		unk	unk
FZ279	resinosa	24	III	-17.79	-65.49	3352		no		unk	unk
FZ282	resinosa	24	III	-17.81	-65.47	3150		no		unk	unk
FZ285	schreiteri	29	III	-17.84	-65.45	2876		no		unk	unk
FZ288	schreiteri	29	III	-17.84	-65.46	2756		no		unk	unk
FZ302	reticulata	25	III	-18.18	-63.84	1877		no		unk	unk
FZ303	resinosa	24	III	-17.85	-64.63	2598		no		unk	unk
FZ309	resinosa	24	III	-17.82	-64.77	3030		no		unk	unk
RS7875	paniculata	19	III	1.11	-77.40	2500		no		unk	unk
APs.n.	leucantha	14	V	-41.63	-73.55	50	yes	no		1	unk
Bs.n.	leucantha	14	V	-40.26	-73.08	50	yes	no		1	unk
CJ1848	leucantha	14	V	-38.87	-73.30	100	yes	no		1	unk
CJ5993	leucantha	14	V	-37.95	-73.33	300	yes	no		1	unk
EK2349	leucantha	14	V	-38.37	-72.00	520	yes	no		1	unk
FZ383	leucantha	14	V	-37.81	-73.14	707	yes	yes	no	1	3
FZ384	leucantha	14	V	-37.81	-73.14	706	yes	yes	yes	1	3
FZ385	leucantha	14	V	-37.81	-73.14	707	yes	yes	no	1	3
FZ387	leucantha	14	V	-37.81	-73.15	702	yes	no		1	unk
FZ389	leucantha	14	V	-37.68	-73.29	132	yes	no		1	unk
HC988	leucantha	14	V	-39.58	-72.25	300	yes	no		1	unk
HG1490.4	leucantha	14	V	-39.88	-73.43	150	yes	no		1	unk
R1225	leucantha	14	V	-37.12	-73.14	200	yes	no		1	unk
TKsn	leucantha	14	V	-39.88	-73.43	150	yes	no		1	unk
TP14306	leucantha	14	V	-42.71	-73.92	180	yes	no		1	unk
WL606	leucantha	14	V	-39.97	-72.67	200	yes	no		1	unk
AS2882	rubra	27	V	-42.86	-71.58	800	yes	no		2	unk
BH5892	rubra	27	V	-41.13	-72.52	300	yes	no		2	unk
CC2	rubra	27	V	-41.40	-73.83	10	yes	yes	yes	2	10
EK1038	rubra	27	V	-39.40	-73.22	51	yes	no		2	unk
EW336	rubra	27	V	-39.68	-72.35	150	yes	no		2	unk
EWs.n.	rubra	27	V	-41.20	-72.55	60	yes	no		2	unk
FZ106	rubra	27	V	-41.23	-72.64	62	yes	yes	no	2	10
FZ107	rubra	27	V	-39.50	-72.15	201	yes	yes	no	2	10
FZ366	rosea	27	V	-37.60	-72.80	774	yes	yes	no	2	6
FZ406	rubra	27	V	-37.39	-71.46	803	yes	yes	no	2	10
FZ425	rubra	27	V	-38.54	-71.68	798	yes	yes	no	2	10
FZ447	rubra	27	V	-38.58	-71.62	1150	yes	yes	no	2	9
FZ475	rubra	27	V	-35.60	-71.04	1605	yes	no		2	unk
FZ527	rosea	27	V	-40.21	-73.40	592	yes	yes	yes	2	4
FZ533	rubra	27	V	-39.68	-73.35	35	yes	yes	no	2	10
FZ97	rubra	27	V	-39.96	-73.57	27	yes	yes	no	2	10
HBsn	rubra	27	V	-46.27	-71.92	500	yes	no		2	unk
HGs.n.	rubra	27	V	-39.87	-73.43	100	yes	no		2	unk

HM81	rubra	27	V	-41.88	-73.88	50	yes	no		2	unk
JM17536	rubra	27	V	-41.33	-72.98	50	yes	no		2	unk
JV3203	rubra	27	V	-39.42	-71.42	1000	yes	no		2	unk
JW4704	rubra	27	V	-41.20	-72.55	100	yes	no		2	unk
MG296	rosea	27	V	-35.86	-71.10	1374	yes	no		2	unk
MG4006	rubra	27	V	-40.77	-72.20	1100	yes	no		2	unk
MG5482	rubra	27	V	-34.98	-70.77	750	yes	no		2	unk
MW6806	rubra	27	V	-40.73	-71.41	830	yes	no		2	unk
OZ8860	rubra	27	V	-40.59	-73.74	45	yes	no		2	unk
PA33372	rubra	27	V	-35.02	-70.80	1000	yes	no		2	unk
PB260	rubra	27	V	-42.82	-72.73	8	yes	no		2	unk
PB368	rubra	27	V	-41.22	-72.68	150	yes	no		2	unk
PHV317	rubra	27	V	-40.68	-71.70	700	yes	no		2	unk
PHV318	rubra	27	V	-40.68	-71.70	700	yes	yes	no	2	10
PHV850	rubra	27	V	-38.81	-73.39	54	yes	no		2	unk
RF5795	rubra	27	V	-42.17	-71.37	600	yes	no		2	unk
WE24417	rubra	27	V	-49.09	-72.55	416	yes	no		2	unk
AS3469	rosea	27	V	-42.75	-72.00	550	yes	no		3	unk
CE10571	alpina	27	V	-40.75	-72.15	1050	yes	no		3	unk
CE10626	alpina	27	V	-43.42	-73.36	600	yes	no		3	unk
CHsn	rosea	27	V	-46.80	-74.07	450	yes	no		3	unk
EW1264	alpina	27	V	-38.70	-71.72	1300	yes	no		3	unk
EW1305	alpina	27	V	-36.90	-71.57	2100	yes	no		3	unk
EW667	alpina	27	V	-41.75	-72.40	1200	yes	no		3	unk
F15998	alpina	27	V	-39.38	-71.98	1200	yes	no		3	unk
FZ331	alpina	27	V	-33.30	-70.32	2033	yes	yes	yes	3	10
FZ332	alpina	27	V	-33.30	-70.32	2033	yes	yes	no	3	10
FZ334	alpina	27	V	-33.30	-70.32	2034	yes	yes	no	3	10
FZ337	alpina	27	V	-33.30	-70.32	2005	yes	yes	no	3	2
FZ338	alpina	27	V	-33.30	-70.31	1980	yes	yes	yes	3	10
FZ400	alpina	27	V	-36.91	-71.40	1912	yes	yes	no	3	10
FZ401	alpina	27	V	-36.91	-71.40	1914	yes	no		3	unk
FZ416	alpina	27	V	-37.38	-71.36	1256	yes	yes	no	3	10
FZ422	alpina	27	V	-37.38	-71.36	1259	yes	yes	no	3	10
FZ424	alpina	27	V	-37.38	-71.36	1255	yes	yes	no	3	10
FZ445	rubra	27	V	-38.58	-71.62	1130	yes	yes	no	3	9
FZ446	rubra	27	V	-38.58	-71.62	1140	yes	yes	no	3	9
FZ451	florida	7	V	-38.58	-71.62	1156	yes	yes	no	3	8
FZ452	florida	7	V	-38.58	-71.62	1156	yes	yes	yes	3	7
FZ518	alpina	27	V	-40.77	-72.20	1130	yes	yes	yes	3	10
FZ523	alpina	27	V	-40.78	-72.19	1276	yes	yes	no	3	10
GIII52	alpina	27	V	-33.33	-70.25	2300	yes	no		3	unk
GS2641	alpina	27	V	-50.87	-72.80	600	yes	no		3	unk
GS49	rosea	27	V	-44.33	-72.53	500	yes	no		3	unk
JK20	alpina	27	V	-45.87	-71.25	700	yes	no		3	unk
JS4381	alpina	27	V	-36.83	-71.42	1200	yes	no		3	unk
JW5159	alpina	27	V	-33.08	-70.77	2100	yes	no		3	unk
KR4614	alpina	27	V	-41.10	-71.58	1650	yes	no		3	unk
M2048	alpina	27	V	-38.58	-71.62	1200	yes	no		3	unk
MB369	rubra	27	V	-40.34	-71.41	1000	yes	no		3	unk
MF6193	alpina	27	V	-51.13	-73.13	50	yes	no		3	unk
MG6031	alpina	27	V	-50.98	-72.85	500	yes	no		3	unk
MG6904	alpina	27	V	-39.02	-71.69	1357	yes	no		3	unk
OZ8086	alpina	27	V	-40.75	-72.15	1000	yes	no		3	unk
OZ8661	rosea	27	V	-50.33	-72.50	185	yes	no		3	unk
PB84	alpina	27	V	-35.95	-70.51	1750	yes	no		3	unk
PB972	alpina	27	V	-38.62	-71.60	1663	yes	no		3	unk
PHV335	alpina	27	V	-51.57	-72.62	140	yes	no		3	unk
PM69850	alpina	27	V	-50.47	-72.48	800	yes	no		3	unk
RF4349	alpina	27	V	-37.83	-71.09	1940	yes	no		3	unk
RWs.n.	alpina	27	V	-41.22	-71.50	1850	yes	no		3	unk
ST2285	alpina	27	V	-33.82	-70.08	2300	yes	no		3	unk
TP14391	alpina	27	V	-45.89	-72.13	1000	yes	no		3	unk
WE24190	alpina	27	V	-51.73	-72.52	50	yes	no		3	unk
ZR138	alpina	27	V	-38.10	-70.92	1776	yes	no		3	unk
Bs.n.	revoluta	26	V	-32.53	-71.45	140	yes	no		4	unk
CJ1847	revoluta	26	V	-38.87	-73.30	100	yes	no		4	unk
EK5372	revoluta	26	V	-31.88	-71.48	20	yes	no		4	unk
EW361	revoluta	26	V	-39.68	-72.35	190	yes	no		4	unk
FZ359	revoluta	26	V	-37.64	-72.79	399	yes	yes	yes	4	1
FZ360	revoluta	26	V	-37.65	-72.76	375	yes	yes	no	4	1
FZ392	rosea	27	V	-36.92	-71.43	1472	yes	no		4	unk
FZ395	rosea	27	V	-36.91	-71.41	1662	yes	yes	no	4	9
FZ428	rosea	27	V	-38.58	-71.63	1091	yes	yes	yes	4	9
FZ456	rosea	27	V	-38.29	-71.77	1056	yes	yes	no	4	9

FZ464	rosea	27	V	-35.47	-70.98	1328	yes	no		4	unk
FZ466	rosea	27	V	-35.47	-70.98	1328	yes	yes	no	4	9
FZ467	rosea	27	V	-35.47	-70.98	1329	yes	yes	no	4	9
FZ470	rosea	27	V	-35.47	-70.97	1312	yes	no		4	unk
FZ477	rosea	27	V	-35.60	-71.02	1661	yes	yes	no	4	9
FZ478	rosea	27	V	-35.60	-71.02	1660	yes	yes	yes	4	9
FZ491	revoluta	26	V	-35.65	-71.25	438	yes	yes	no	4	1
FZ495	revoluta	26	V	-35.65	-71.26	437	yes	yes	no	4	1
FZ496	myrtoidea	26	V	-35.92	-71.37	342	yes	yes	no	4	1
FZ497	myrtoidea	26	V	-35.92	-71.37	342	yes	yes	no	4	1
FZ499	revoluta	26	V	-35.64	-71.48	266	yes	yes	no	4	1
LL4440	revoluta	26	V	-36.92	-72.67	200	yes	no		4	unk
MG291	rosea	27	V	-35.86	-71.10	1374	yes	no		4	unk
MG300	revoluta	26	V	-35.87	-71.12	758	yes	no		4	unk
MG4676	revoluta	26	V	-36.83	-72.68	250	yes	no		4	unk
MG5084	revoluta	26	V	-32.57	-71.45	116	yes	no		4	unk
MG5475	revoluta	26	V	-34.98	-70.77	750	yes	no		4	unk
MG85	rosea	27	V	-35.47	-70.98	1341	yes	no		4	unk
MG86	rosea	27	V	-35.47	-70.98	1341	yes	no		4	unk
OZ17557	rubra	27	V	-33.02	-71.27	500	yes	no		4	unk
OZ9278	myrtoidea	26	V	-34.95	-70.43	2000	yes	no		4	unk
PB1175	revoluta	26	V	-35.67	-71.25	424	yes	no		4	unk
PB455	revoluta	26	V	-37.39	-71.44	1000	yes	no		4	unk
PB487	revoluta	26	V	-37.81	-72.90	670	yes	no		4	unk
PB71	myrtoidea	26	V	-35.91	-70.64	1470	yes	no		4	unk
QH526	revoluta	26	V	-38.67	-72.00	500	yes	no		4	unk
TP14215	rosea	27	V	-38.33	-72.06	500	yes	no		4	unk
FZ103	rubra	27	V	-41.14	-72.40	204	yes	yes	no	5	10
EPIII64	florida	7	V	-37.30	-71.95	1200	yes	no		6	unk
FZ362	leucantha	14	V	-37.77	-72.80	773	yes	yes	no	6	6
FZ363	leucantha	14	V	-37.79	-72.82	773	yes	yes	no	6	6
FZ367	leucantha	14	V	-37.60	-72.80	775	yes	yes	yes	6	6
FZ368	leucantha	14	V	-37.60	-72.80	775	yes	yes	yes	6	6
FZ431	florida	7	V	-38.58	-71.63	1055	yes	yes	no	6	5
FZ432	florida	7	V	-38.58	-71.63	1070	yes	yes	no	6	5
FZ433	florida	7	V	-38.58	-71.63	1070	yes	yes	yes	6	5
FZ434	florida	7	V	-38.58	-71.63	1100	yes	yes	no	6	5
FZ435	florida	7	V	-38.58	-71.62	1121	yes	yes	yes	6	5
FZ436	florida	7	V	-38.58	-71.62	1149	yes	yes	yes	6	8
FZ437	florida	7	V	-38.58	-71.62	1149	yes	yes	no	6	8
FZ438	florida	7	V	-38.58	-71.62	1164	yes	yes	no	6	8
FZ448	florida	7	V	-38.58	-71.62	1156	yes	yes	no	6	8
FZ449	florida	7	V	-38.58	-71.62	1156	yes	yes	yes	6	8
FZ450	florida	7	V	-38.58	-71.62	1156	yes	yes	no	6	8
FZ453	florida	7	V	-38.58	-71.62	1114	yes	no		6	unk
GM2701	florida	7	V	-38.55	-71.68	1150	yes	no		6	unk
GS2237	alpina	27	V	-38.87	-70.48	1900	yes	no		6	unk
MG4975	florida	7	V	-37.78	-72.82	800	yes	no		6	unk
MG5549	florida	7	V	-37.72	-71.22	1040	yes	no		6	unk
OZ2302	florida	7	V	-37.28	-71.72	2000	yes	no		6	unk
PB479	florida	7	V	-37.40	-71.49	1150	yes	no		6	unk
PB835	florida	7	V	-37.61	-72.77	624	yes	no		6	unk
PB838	florida	7	V	-37.61	-72.77	624	yes	no		6	unk
CT10396	rosea	27	V	-37.67	-73.00	300	yes	no		7	unk
FZ119	rubra	27	V	-33.73	-70.47	954	yes	yes	yes	7	10
FZ379	rosea	27	V	-37.81	-73.06	984	yes	yes	no	7	9
FZ394	rosea	27	V	-36.91	-71.41	1662	yes	no		7	unk
FZ426	rosea	27	V	-38.54	-71.68	798	yes	yes	no	7	9
FZ427	rosea	27	V	-38.54	-71.68	798	yes	yes	no	7	9
FZ439	rubra	27	V	-38.58	-71.62	1165	yes	yes	no	7	9
FZ440	rubra	27	V	-38.58	-71.62	1166	yes	yes	no	7	9
FZ441	rubra	27	V	-38.58	-71.62	1130	yes	yes	no	7	9
FZ442	rubra	27	V	-38.58	-71.62	1130	yes	yes	yes	7	9
FZ443	rubra	27	V	-38.58	-71.62	1130	yes	yes	no	7	9
FZ444	rubra	27	V	-38.58	-71.62	1130	yes	yes	yes	7	9
FZ457	rosea	27	V	-38.25	-71.75	1068	yes	yes	no	7	9
FZ479	alpina	27	V	-35.60	-71.01	1913	yes	yes	no	7	9
FZ489	alpina	27	V	-35.60	-71.01	1921	yes	yes	yes	7	9
FZ500	rubra	27	V	-36.60	-71.47	528	yes	yes	no	7	10
FZ510	rosea	27	V	-40.77	-72.27	725	yes	yes	yes	7	9
FZ512	rosea	27	V	-40.77	-72.27	720	yes	no		7	unk
FZ531	rosea	27	V	-40.18	-73.44	899	yes	no		7	unk
FZ532	rosea	27	V	-40.18	-73.44	900	yes	yes	yes	7	9
HC80	rubra	27	V	-33.05	-71.60	31	yes	no		7	unk
JB32862	rubra	27	V	-51.67	-72.53	50	yes	no		7	unk

JW3985	rubra	27	V	-33.10	-71.68	200	yes	no		7	unk
LL4342	rubra	27	V	-33.33	-71.67	80	yes	no		7	unk
LL5968	rubra	27	V	-33.33	-71.67	100	yes	no		7	unk
MC12841	rosea	27	V	-40.12	-73.57	840	yes	no		7	unk
MG3452	rosea	27	V	-41.17	-72.50	200	yes	no		7	unk
MG4409	rosea	27	V	-35.50	-71.17	1500	yes	no		7	unk
OB6523	rubra	27	V	-33.29	-71.64	170	yes	no		7	unk
OB6527	rubra	27	V	-33.14	-71.70	111	yes	no		7	unk
OZ5556	rubra	27	V	-37.41	-71.61	1200	yes	no		7	unk
OZ9429	rubra	27	V	-33.27	-71.65	100	yes	no		7	unk
PHV75	rubra	27	V	-40.34	-72.25	400	yes	no		7	unk
TP14473	alpina	27	V	-51.58	-72.76	20	yes	no		7	unk
CT11000	revoluta	26	V	-35.85	-71.25	900	yes	no		8	unk
FZ390	leucantha	14	V	-37.68	-73.29	132	yes	yes	no	8	3
FZ542	myrtoidea	26	V	-33.73	-70.47	920	yes	yes	no	8	1
FZ543	myrtoidea	26	V	-33.73	-70.47	920	yes	no		8	unk
FZ544	myrtoidea	26	V	-33.73	-70.47	920	yes	yes	yes	8	1
MG381	myrtoidea	26	V	-36.58	-71.46	811	yes	no		8	unk
MG384	myrtoidea	26	V	-36.58	-71.46	811	yes	no		8	unk
MG5137	myrtoidea	26	V	-33.33	-70.37	1270	yes	no		8	unk
MG532	myrtoidea	26	V	-36.58	-71.46	811	yes	no		8	unk
MG5444	revoluta	26	V	-33.07	-70.95	800	yes	no		8	unk
MG5492	myrtoidea	26	V	-34.98	-70.77	750	yes	no		8	unk
MG568	myrtoidea	26	V	-37.68	-73.23	485	yes	no		8	unk
MG6977	revoluta	26	V	-39.88	-73.16	1	yes	no		8	unk
OB6473	myrtoidea	26	V	-33.00	-71.01	1200	yes	no		8	unk
OZ5577	myrtoidea	26	V	-29.97	-70.93	1000	yes	no		8	unk
OZ8726	revoluta	26	V	-31.98	-71.45	880	yes	no		8	unk
PB644	myrtoidea	26	V	-33.32	-70.32	1700	yes	no		8	unk
YM7858	myrtoidea	26	V	-35.05	-70.57	1240	yes	no		8	unk
FZ100	leucantha	14	V	-39.96	-73.35	171		yes	yes	unk	3
FZ101	rubra	27	V	-41.31	-72.98	20		yes	no	unk	10
FZ102	rubra	27	V	-41.13	-72.42	50		yes	no	unk	10
FZ104	rosea	27	V	-41.14	-72.54	908		yes	no	unk	9
FZ105	rosea	27	V	-41.14	-72.53	922		yes	no	unk	9
FZ110	rubra	27	V	-39.65	-72.32	232		yes	no	unk	10
FZ112	alpina	27	V	-40.78	-72.20	1050		yes	no	unk	10
FZ113	rosea	27	V	-40.77	-72.27	721		yes	no	unk	9
FZ114	rosea	27	V	-40.77	-72.27	721		yes	no	unk	9
FZ115	rosea	27	V	-40.77	-72.27	721		yes	no	unk	9
FZ116	leucantha	14	V	-40.00	-73.00	90		yes	no	unk	3
FZ125	revoluta	26	V	-33.00	-71.03	1642		yes	no	unk	1
FZ126	myrtoidea	26	V	-32.99	-71.03	1650		yes	no	unk	1
FZ330	myrtoidea	26	V	-33.31	-70.33	1945		yes	no	unk	1
FZ333	myrtoidea	26	V	-33.30	-70.32	2034		yes	no	unk	1
FZ335	alpina	27	V	-33.30	-70.32	2037		yes	no	unk	10
FZ336	alpina	27	V	-33.30	-70.32	2008		yes	yes	unk	2
FZ347	myrtoidea	26	V	-33.30	-70.32	2086		yes	no	unk	1
FZ348	myrtoidea	26	V	-33.30	-70.32	2060		yes	no	unk	1
FZ352	myrtoidea	26	V	-33.32	-70.33	1901		yes	no	unk	1
FZ365	revoluta	26	V	-37.60	-72.80	773		yes	no	unk	1
FZ413	revoluta	26	V	-37.39	-71.46	781		yes	no	unk	1
FZ429	florida	7	V	-38.58	-71.63	1049		yes	no	unk	5
FZ430	florida	7	V	-38.58	-71.63	1055		yes	no	unk	5
FZ454	revoluta	26	V	-38.35	-71.85	664		yes	no	unk	1
FZ501	revoluta	26	V	-36.60	-71.47	530		yes	no	unk	1
FZ503	revoluta	26	V	-36.60	-71.47	529		yes	no	unk	1
FZ92	alpina	27	V	-33.30	-70.32	2085		yes	yes	unk	2
FZ94	alpina	27	V	-33.30	-70.31	2165		yes	no	unk	10
FZ98	rubra	27	V	-39.94	-73.58	12		yes	no	unk	10
PHV336	alpina		V	-51.57	-72.62	140		yes	no	unk	10
Bs.n.	leucantha	14	V	-40.26	-73.08	50	yes	no		unk	unk
Bs.n.	revoluta	26	V	-32.53	-71.45	140	yes	no		unk	unk
FZ120	myrtoidea	26	V	-33.73	-70.47	954		no		unk	unk
FZ346	myrtoidea	26	V	-33.30	-70.32	2102		no		unk	unk
FZ349	myrtoidea	26	V	-33.31	-70.32	1989		no		unk	unk
FZ350	myrtoidea	26	V	-33.31	-70.32	1989		no		unk	unk
FZ351	myrtoidea	26	V	-33.31	-70.33	1943		no		unk	unk
FZ382	leucantha	14	V	-37.81	-73.14	707		no		unk	unk
FZ91	myrtoidea	26	V	-33.32	-70.33	1892		no		unk	unk
FZ93	myrtoidea	26	V	-33.30	-70.32	2089		no		unk	unk
PB500	myrtoidea	26	V	-37.68	-73.30	120		no		unk	unk
TP14476	alpina	27	V	-51.58	-72.76	20		no		unk	unk
AB10830	laevis	12	VI	-22.47	-43.05	2100	yes	no		1	unk
AH22218	tucumanensis	31	VI	-28.23	-65.95	2800	yes	no		1	unk

B19021	laevis	12	VI	-22.48	-45.08	2000	yes	no		1	unk
BLs.n.	laevis	12	VI	-22.38	-44.66	2750	yes	no		1	unk
DS152	hypoglauca	31	VI	-17.84	-64.74	2750	yes	no		1	unk
EB383	laevis	12	VI	-25.30	-48.88	866	yes	no		1	unk
EF16	laevis	12	VI	-22.40	-44.65	2300	yes	no		1	unk
EF3538	hypoglauca	31	VI	-17.80	-64.77	2900	yes	no		1	unk
EJ75	laevis	12	VI	-22.36	-42.58	2000	yes	no		1	unk
EU1886	petrophila	21	VI	-26.50	-50.50	1020	yes	no		1	unk
FB6142	tucumanensis	31	VI	-21.40	-64.27	1600	yes	no		1	unk
FV6838	tucumanensis	31	VI	-27.13	-65.50	1250	yes	no		1	unk
FZ304	hypoglauca	31	VI	-17.83	-64.72	2843	yes	yes	no	1	4
FZ307	hypoglauca	31	VI	-17.84	-64.73	2928	yes	no		1	unk
FZU10377C	tucumanensis	31	VI	-22.33	-64.72	1690	yes	no		1	unk
GB1080	tucumanensis	31	VI	-23.70	-64.90	1750	yes	no		1	unk
GH61252	petrophila	21	VI	-29.15	-50.12	800	yes	no		1	unk
GH71660	laevis	12	VI	-28.13	-49.50	1700	yes	no		1	unk
JC1636	laevis	12	VI	-25.25	-48.85	1740	yes	no		1	unk
JK1720	laevis	12	VI	-22.47	-43.05	2150	yes	no		1	unk
JS10129	tucumanensis	31	VI	-22.20	-64.53	1000	yes	no		1	unk
JT229	hypoglauca	31	VI	-17.84	-64.73	2969	yes	no		1	unk
JV135	laevis	12	VI	-22.47	-43.06	2263	yes	no		1	unk
KK10696	tucumanensis	31	VI	-27.40	-65.94	1400	yes	no		1	unk
LF86	laevis	12	VI	-25.24	-48.83	1823	yes	yes	yes	1	10
LF87	laevis	12	VI	-25.24	-48.83	1823	yes	yes	yes	1	10
LS1492	laevis	12	VI	-22.39	-44.62	2150	yes	no		1	unk
M 4544	tucumanensis	31	VI	-26.81	-65.38	1200	yes	no		1	unk
MN33903	hypoglauca	31	VI	-18.57	-64.05	2200	yes	yes	no	1	4
MS5087	tucumanensis	31	VI	-22.01	-64.59	2396	yes	no		1	unk
PC584	tucumanensis	31	VI	-28.74	-66.98	1850	yes	no		1	unk
PDs.n.	laevis	12	VI	-22.38	-44.66	2750	yes	no		1	unk
PL5936C	tucumanensis	31	VI	-23.47	-65.00	1700	yes	no		1	unk
PR2962	laevis	12	VI	-28.14	-49.53	1860	yes	no		1	unk
RF1966	tucumanensis	31	VI	-24.02	-65.39	1700	yes	no		1	unk
RW18130	angustifolia	4	VI	-29.95	-70.55	2400	yes	yes	yes	1	2
SV10256	tucumanensis	31	VI	-26.70	-65.45	1500	yes	no		1	unk
SV1421	tucumanensis	31	VI	-26.87	-65.38	1100	yes	no		1	unk
SV2114	tucumanensis	31	VI	-26.87	-65.36	800	yes	no		1	unk
SV3985	tucumanensis	31	VI	-27.42	-66.08	2000	yes	no		1	unk
T4987	tucumanensis	31	VI	-22.31	-64.67	1750	yes	no		1	unk
WP167	laevis	12	VI	-22.48	-45.08	2205	yes	yes	yes	1	11
WP168	laevis	12	VI	-22.48	-45.08	2216	yes	yes	no	1	11
AB11510	bifida	2	VI	-22.45	-43.05	1900	yes	no		2	unk
AG6890	bifida	2	VI	-22.26	-42.55	1200	yes	no		2	unk
AK22941	bifida	2	VI	-31.50	-52.38	100	yes	no		2	unk
AK23025	bifida	2	VI	-28.68	-50.97	800	yes	no		2	unk
AK35523	bifida	2	VI	-25.33	-49.06	940	yes	no		2	unk
AVEsc2	bifida	2	VI	-25.45	-49.02	980	yes	no		2	unk
BR3907	bifida	2	VI	-30.88	-55.52	170	yes	no		2	unk
CC507	bifida	2	VI	-22.45	-43.00	1700	yes	no		2	unk
CO6589	bifida	2	VI	-31.55	-56.10	250	yes	no		2	unk
EP7047	bifida	2	VI	-22.38	-44.65	2300	yes	no		2	unk
EP8355	bifida	2	VI	-26.17	-49.85	850	yes	no		2	unk
FM2376	bifida	2	VI	-26.17	-55.33	250	yes	no		2	unk
GH18306	bifida	2	VI	-25.27	-51.10	780	yes	no		2	unk
GH18331	bifida	2	VI	-25.29	-51.35	1150	yes	no		2	unk
GH18425	bifida	2	VI	-25.31	-49.05	916	yes	no		2	unk
GH26215	bifida	2	VI	-25.53	-49.38	884	yes	no		2	unk
GH31430	bifida	2	VI	-20.45	-41.77	1900	yes	no		2	unk
HL550	bifida	2	VI	-22.38	-44.60	2000	yes	no		2	unk
JB5177	bifida	2	VI	-29.57	-51.36	80	yes	no		2	unk
JK1721	bifida	2	VI	-22.47	-43.05	2150	yes	no		2	unk
JM14715	bifida	2	VI	-26.53	-54.73	205	yes	no		2	unk
LF16	bifida	2	VI	-25.49	-49.04	914	yes	yes	no	2	5
LF18	bifida	2	VI	-25.49	-49.04	915	yes	yes	yes	2	5
LF33	bifida	2	VI	-27.67	-49.21	709	yes	yes	no	2	5
LF39	bifida	2	VI	-28.06	-49.49	1194	yes	yes	no	2	5
LF46	bifida	2	VI	-28.06	-49.37	1130	yes	yes	no	2	5
LF6	bifida	2	VI	-25.45	-49.24	900	yes	yes	no	2	5
LF61	bifida	2	VI	-27.89	-50.14	1116	yes	yes	no	2	5
LS10571	bifida	2	VI	-26.20	-49.23	1000	yes	no		2	unk
LS11641	bifida	2	VI	-26.40	-53.13	1000	yes	no		2	unk
MR221	bifida	2	VI	-22.70	-45.58	1700	yes	no		2	unk
PJ34248	bifida	2	VI	-27.48	-55.48	220	yes	no		2	unk
PR11764	bifida	2	VI	-26.82	-51.00	800	yes	no		2	unk
PR12237	bifida	2	VI	-27.30	-50.55	900	yes	no		2	unk

PR6686	bifida	2	VI	-28.03	-49.61	1099	yes	no		2	unk
RB93	bifida	2	VI	-22.72	-45.45	1800	yes	no		2	unk
RD10799	bifida	2	VI	-34.05	-54.60	70	yes	no		2	unk
RD1744	bifida	2	VI	-26.17	-55.83	250	yes	no		2	unk
RW423	bifida	2	VI	-29.45	-50.63	830	yes	no		2	unk
RW5162	bifida	2	VI	-29.22	-51.37	700	yes	no		2	unk
RW5166	bifida	2	VI	-29.17	-51.08	780	yes	no		2	unk
WP155	bifida	2	VI	-22.77	-45.54	1872	yes	yes	no	2	5
WP163	bifida	2	VI	-22.61	-45.56	1556	yes	yes	no	2	5
WP169	bifida	2	VI	-22.48	-45.08	2194	yes	yes	yes	2	5
AB5112	megapotamica	15	VI	-32.25	-60.61	40	yes	no		3	unk
AK42001	megapotamica	15	VI	-27.86	-50.80	990	yes	no		3	unk
AL2215	megapotamica	15	VI	-27.85	-50.22	1000	yes	no		3	unk
ARs.n.	megapotamica	15	VI	-21.93	-46.42	900	yes	no		3	unk
BR49592	megapotamica	15	VI	-27.85	-50.22	900	yes	no		3	unk
CK42	megapotamica	15	VI	-26.21	-51.10	800	yes	no		3	unk
GH15362	megapotamica	15	VI	-25.61	-50.69	800	yes	no		3	unk
HF72	megapotamica	15	VI	-34.35	-57.68	67	yes	no		3	unk
IT20026	megapotamica	15	VI	-31.57	-52.62	240	yes	no		3	unk
LS367	megapotamica	15	VI	-29.12	-51.24	731	yes	no		3	unk
MS6428	megapotamica	15	VI	-30.54	-53.48	350	yes	no		3	unk
PR14398	megapotamica	15	VI	-27.50	-51.47	800	yes	no		3	unk
RW842	megapotamica	15	VI	-28.90	-50.38	900	yes	no		3	unk
AC1193	hypoglauca	31	VI	-19.57	-64.32	2552	yes	no		4	unk
AS75	laevis	12	VI	-22.38	-44.70	2000	yes	no		4	unk
BL734	laevis	12	VI	-23.23	-44.95	1262	yes	no		4	unk
CD3771	hypoglauca	31	VI	-17.59	-65.27	2900	yes	no		4	unk
FZU10117A	tucumanensis	31	VI	-25.73	-65.48	1900	yes	yes	no	4	4
FZU10330	hypoglauca	31	VI	-23.61	-64.91	2810	yes	yes	no	4	4
FZU10426A	hypoglauca	31	VI	-21.46	-64.87	3033	yes	no		4	unk
FZU10426D	hypoglauca	31	VI	-21.46	-64.87	3033	yes	yes	no	4	4
FZU10434A	hypoglauca	31	VI	-21.47	-64.89	3398	yes	yes	yes	4	4
GH35823	laevis	12	VI	-22.38	-44.63	2000	yes	no		4	unk
HF5850	hypoglauca	31	VI	-23.45	-65.47	2800	yes	no		4	unk
HS3739	hypoglauca	31	VI	-22.27	-65.07	3300	yes	no		4	unk
HS763	laevis	12	VI	-22.38	-44.69	2400	yes	no		4	unk
JG587	hypoglauca	31	VI	-20.75	-64.54	2710	yes	no		4	unk
JW10607	hypoglauca	31	VI	-18.59	-64.04	2300	yes	no		4	unk
JW8943	hypoglauca	31	VI	-17.80	-66.42	3500	yes	no		4	unk
JW9552	hypoglauca	31	VI	-21.47	-64.89	3300	yes	yes	no	4	4
KF2441	hypoglauca	31	VI	-21.46	-64.86	2800	yes	no		4	unk
LS1493	laevis	12	VI	-22.39	-44.62	2150	yes	no		4	unk
MC6241	hypoglauca	31	VI	-17.63	-65.73	2900	yes	no		4	unk
MG5665	illinita	4	VI	-30.17	-70.65	1510	yes	no		4	unk
MS5081	hypoglauca	31	VI	-22.01	-64.59	2396	yes	no		4	unk
Ns.n.	laevis	12	VI	-22.75	-44.68	1543	yes	no		4	unk
PB4540	laevis	12	VI	-20.44	-41.81	2740	yes	no		4	unk
PR62989	laevis	12	VI	-22.47	-43.05	2100	yes	no		4	unk
Ss.n.	laevis	12	VI	-20.40	-41.79	2000	yes	no		4	unk
SV9001	hypoglauca	31	VI	-23.72	-65.67	2500	yes	no		4	unk
TM3492	tucumanensis	31	VI	-29.22	-66.86	2000	yes	no		4	unk
WP166	laevis	12	VI	-22.48	-45.08	2202	yes	no		4	unk
AK42087	farinacea	6	VI	-28.06	-50.07	1100	yes	no		5	unk
BR49669	farinacea	6	VI	-27.85	-50.22	1000	yes	no		5	unk
GH10795	farinacea	6	VI	-25.54	-49.89	850	yes	no		5	unk
GH11940	farinacea	6	VI	-24.32	-49.67	860	yes	no		5	unk
GH17979	farinacea	6	VI	-25.13	-49.97	1030	yes	no		5	unk
GH18054	farinacea	6	VI	-25.07	-49.40	950	yes	no		5	unk
GH18440	farinacea	6	VI	-26.00	-49.65	830	yes	no		5	unk
GH25426	farinacea	6	VI	-24.41	-49.86	1060	yes	no		5	unk
JS38	farinacea	6	VI	-25.39	-49.98	812	yes	no		5	unk
LF10	farinacea	6	VI	-25.51	-49.05	927	yes	yes	yes	5	7
LS9075	farinacea	6	VI	-26.66	-50.96	1250	yes	no		5	unk
MK902	farinacea	6	VI	-25.58	-49.25	890	yes	no		5	unk
NS5746	farinacea	6	VI	-22.74	-45.59	1650	yes	no		5	unk
PD15954	farinacea	6	VI	-24.25	-49.70	850	yes	no		5	unk
SX262	farinacea	6	VI	-22.71	-45.57	1650	yes	no		5	unk
VN108	farinacea	6	VI	-25.03	-49.03	1050	yes	no		5	unk
WP159	farinacea	6	VI	-22.77	-45.53	1810	yes	no		5	unk
BR49366	megapotamica	15	VI	-29.45	-50.58	880	yes	no		6	unk
GS2702	megapotamica	15	VI	-31.66	-56.01	149	yes	no		6	unk
H1015	megapotamica	15	VI	-32.68	-58.13	50	yes	no		6	unk
JI165	megapotamica	15	VI	-29.27	-57.57	60	yes	no		6	unk
JW1950	megapotamica	15	VI	-28.03	-51.18	890	yes	no		6	unk
LL3886	megapotamica	15	VI	-25.75	-49.25	900	yes	no		6	unk

LS293	megapotamica	15	VI	-25.43	-50.52	800	yes	no		6	unk
NT2455	megapotamica	15	VI	-31.56	-58.17	30	yes	no		6	unk
RG5802	megapotamica	15	VI	-33.43	-55.90	100	yes	no		6	unk
RV3718	megapotamica	15	VI	-28.44	-56.02	70	yes	no		6	unk
SR3089	megapotamica	15	VI	-27.75	-55.75	170	yes	no		6	unk
SR3711	megapotamica	15	VI	-29.12	-57.92	85	yes	no		6	unk
TP15595	megapotamica	15	VI	-29.07	-57.35	80	yes	no		6	unk
TP5371	megapotamica	15	VI	-29.28	-57.73	70	yes	no		6	unk
CB984	illinita	11	VI	-33.05	-71.52	200	yes	no		7	unk
CSs.n.	illinita	11	VI	-34.25	-70.57	780	yes	no		7	unk
EK5378	illinita	11	VI	-31.42	-71.58	40	yes	no		7	unk
EK5390	illinita	11	VI	-33.35	-71.58	180	yes	no		7	unk
EK838	illinita	11	VI	-35.00	-70.82	700	yes	no		7	unk
EW579	illinita	11	VI	-35.20	-70.80	1500	yes	no		7	unk
FZ539	illinita	11	VI	-33.73	-70.47	920	yes	yes	no	7	1
FZ540	illinita	11	VI	-33.73	-70.47	920	yes	yes	yes	7	1
FZ541	illinita	11	VI	-33.73	-70.47	920	yes	no		7	unk
GH405A	illinita	11	VI	-32.87	-70.42	1100	yes	no		7	unk
GH405B	illinita	11	VI	-32.87	-70.42	1100	yes	no		7	unk
GL5551	illinita	11	VI	-33.18	-70.60	900	yes	no		7	unk
JW5200	illinita	11	VI	-32.97	-71.27	400	yes	no		7	unk
LL5949	illinita	11	VI	-33.71	-70.32	1200	yes	no		7	unk
MG25	illinita	11	VI	-35.11	-71.03	668	yes	no		7	unk
MG5435	illinita	11	VI	-33.07	-70.95	600	yes	no		7	unk
MG5655	illinita	11	VI	-30.22	-71.33	120	yes	no		7	unk
OZ6524	illinita	11	VI	-33.87	-70.40	1500	yes	no		7	unk
PB4	illinita	11	VI	-32.95	-71.08	700	yes	no		7	unk
EW183	angustifolia	4	VI	-29.85	-70.39	1600	yes	no		8	unk
FZ322	angustifolia	1	VI	-16.56	-71.45	2594	yes	yes	yes	8	3
FZ323	angustifolia	1	VI	-16.56	-71.45	2579	yes	yes	no	8	3
FZ324	angustifolia	1	VI	-16.56	-71.45	2585	yes	yes	no	8	3
FZ325	angustifolia	1	VI	-16.56	-71.45	2582	yes	no		8	unk
FZ326	angustifolia	1	VI	-16.56	-71.45	2590	yes	yes	yes	8	3
FZ327	angustifolia	1	VI	-16.56	-71.44	2684	yes	yes	no	8	3
GH72506	ledifolia	13	VI	-28.06	-49.59	1150	yes	no		8	unk
IJ5864	angustifolia	4	VI	-28.90	-70.07	2050	yes	no		8	unk
MG6297	angustifolia	1	VI	-18.66	-69.58	3280	yes	no		8	unk
MG6302	angustifolia	1	VI	-18.83	-69.75	1800	yes	yes	no	8	3
MG6562	angustifolia	1	VI	-20.08	-69.36	1988	yes	no		8	unk
OZ3990	angustifolia	4	VI	-29.93	-70.30	1800	yes	no		8	unk
PB512	illinita	4	VI	-30.14	-70.05	2650	yes	yes	no	8	2
RK4485	ledifolia	13	VI	-27.85	-50.27	950	yes	no		8	unk
RK7653	angustifolia	4	VI	-31.08	-69.58	2100	yes	no		8	unk
FZ123	illinita	11	VI	-33.73	-70.47	960		yes	yes	unk	1
FZ124	illinita	11	VI	-33.73	-70.47	950		yes	no	unk	1
FZ127	illinita	11	VI	-33.01	-70.90	722		yes	no	unk	1
FZ281	hypoglauca	31	VI	-17.79	-65.49	3352		yes	no	unk	4
FZ305	hypoglauca	31	VI	-17.83	-64.72	2846		yes	no	unk	4
FZ306	hypoglauca	31	VI	-17.83	-64.72	2850		yes	no	unk	4
FZ308	hypoglauca	31	VI	-17.84	-64.73	2928		yes	no	unk	4
FZ353	illinita	11	VI	-33.32	-70.33	1872		yes	no	unk	1
FZU10003A	tucumanensis		VI	-27.06	-65.67	900		yes	yes	unk	4
FZU10003B	tucumanensis	31	VI	-27.06	-65.67	900		yes	no	unk	4
FZU10117C	tucumanensis		VI	-25.73	-65.48	1900		yes	no	unk	4
FZU10377A	tucumanensis	31	VI	-22.33	-64.72	1690		yes	no	unk	4
FZU10406A	tucumanensis		VI	-21.43	-64.27	1670		yes	no	unk	4
FZU10406C	tucumanensis		VI	-21.43	-64.27	1670		yes	no	unk	4
IA1263	tucumanensis		VI	-22.17	-64.68	1300		yes	no	unk	4
JW11630	tucumanensis		VI	-21.42	-64.30	2000		yes	no	unk	4
LF15	farinacea	6	VI	-25.49	-49.04	914		yes	no	unk	7
LF42	petrophila	21	VI	-28.06	-49.37	1130		yes	yes	unk	9
LF44	petrophila	21	VI	-28.06	-49.37	1128		yes	yes	unk	9
LF45	petrophila	21	VI	-28.06	-49.37	1131		yes	no	unk	9
LF51	ledifolia	13	VI	-27.84	-49.65	1108		yes	no	unk	8
LF52	ledifolia	13	VI	-27.84	-49.65	1110		yes	yes	unk	8
LF53	ledifolia	13	VI	-27.84	-49.65	1109		yes	no	unk	8
LF54	ledifolia	13	VI	-27.84	-49.65	1109		yes	no	unk	8
LF55	ledifolia	13	VI	-27.84	-49.65	1109		yes	no	unk	8
LF56	ledifolia	13	VI	-27.84	-49.65	1108		yes	no	unk	8
LF57	ledifolia	13	VI	-27.84	-49.65	1109		yes	no	unk	8
LF59	ledifolia	13	VI	-27.84	-49.65	1109		yes	yes	unk	8
LF60	ledifolia	13	VI	-27.84	-49.65	1109		yes	no	unk	8
LF63	farinacea	6	VI	-27.89	-50.14	1116		yes	no	unk	7
LF64	farinacea	6	VI	-27.89	-50.14	1117		yes	no	unk	7
LF72	megapotamica	15	VI	-26.10	-49.83	780		yes	yes	unk	6

LF73	megapotamica	15	VI	-26.10	-49.83	780		yes	no	unk	6
LF75	megapotamica	15	VI	-25.88	-50.38	761		yes	yes	unk	6
LF76	megapotamica	15	VI	-25.88	-50.38	761		yes	no	unk	6
LF77	megapotamica	15	VI	-25.88	-50.38	760		yes	no	unk	6
LF78	megapotamica	15	VI	-25.88	-50.38	760		yes	no	unk	6
LF80	farinacea	6	VI	-25.47	-49.77	958		yes	no	unk	7
LF81	farinacea	6	VI	-25.47	-49.77	960		yes	no	unk	7
LF82	laevis	12	VI	-25.24	-48.83	1823		yes	no	unk	10
LF83	laevis	12	VI	-25.24	-48.83	1821		yes	no	unk	10
LF84	laevis	12	VI	-25.24	-48.83	1823		yes	no	unk	10
LF85	laevis	12	VI	-25.24	-48.83	1824		yes	no	unk	10
RK10198	angustifolia		VI	-31.38	-69.70	2000		yes	yes	unk	2
WP152	farinacea	6	VI	-22.76	-45.55	1796		yes	yes	unk	7
WP154	farinacea	6	VI	-22.76	-45.55	1796		yes	no	unk	7
WP158	farinacea	6	VI	-22.77	-45.54	1872		yes	no	unk	7
WP160	farinacea	6	VI	-22.77	-45.53	1808		yes	no	unk	7
WP164	laevis	12	VI	-22.48	-45.08	2149		yes	no	unk	11
WP165	laevis	12	VI	-22.48	-45.08	2173		yes	yes	unk	11
BR49389	petrophila	21	VI	-29.45	-50.58	890		no		unk	unk
BR50074	petrophila	21	VI	-29.45	-50.58	891		no		unk	unk
FZ280	hypoglauca	31	VI	-17.79	-65.49	3352		no		unk	unk
FZU1046A	tucumanensis	31	VI	-21.43	-64.27	1670		no		unk	unk
LF43	petrophila	21	VI	-28.06	-49.37	1130		no		unk	unk
DS4998	polifolia	22	IV	-6.75	-77.80	3000	yes	no		1	unk
FZ223	polifolia	22	IV	-6.71	-77.85	3170	yes	yes	yes	1	3
FZ224	polifolia	22	IV	-6.71	-77.85	3170	yes	yes	no	1	3
FZ225	polifolia	22	IV	-6.71	-77.85	3170	yes	yes	yes	1	3
FZ226	polifolia	22	IV	-6.71	-77.85	3170	yes	yes	no	1	3
JB1810	polifolia	22	IV	-6.74	-77.87	3500	yes	no		1	unk
JL11384	polifolia	22	IV	-6.75	-77.80	3000	yes	no		1	unk
JL5574	polifolia	22	IV	-6.75	-77.77	2900	yes	no		1	unk
PHV5511	polifolia	22	IV	-6.71	-77.76	3100	yes	no		1	unk
AB3728	myrtilloides	17	IV	7.27	-72.92	3100	yes	no		2	unk
AC3134	myrtilloides	17	IV	4.57	-74.03	3500	yes	no		2	unk
AF8711	myrtilloides	17	IV	-14.65	-68.96	3300	yes	no		2	unk
AL7565	myrtilloides	17	IV	-9.37	-77.24	3800	yes	no		2	unk
AM8010	myrtilloides	17	IV	-10.20	-75.38	3250	yes	no		2	unk
BO8614	myrtilloides	17	IV	0.33	-78.00	3700	yes	no		2	unk
CU1445	myrtilloides	17	IV	-3.10	-79.22	3319	yes	yes	no	2	1
CU1449	myrtilloides	17	IV	-2.94	-78.71	2926	yes	no		2	unk
DS10115	myrtilloides	17	IV	-9.70	-77.32	4100	yes	no		2	unk
FG3239	myrtilloides	17	IV	5.65	-73.32	3000	yes	no		2	unk
FL4756	myrtilloides	17	IV	2.52	-76.19	3450	yes	no		2	unk
FL7708	myrtilloides	17	IV	2.52	-76.19	3500	yes	no		2	unk
FZ169	myrtilloides	17	IV	-14.06	-71.33	3902	yes	yes	yes	2	4
FZ170	myrtilloides	17	IV	-14.06	-71.33	3900	yes	yes	no	2	4
FZ176	myrtilloides	17	IV	-13.16	-72.28	3732	yes	yes	no	2	4
FZ177	myrtilloides	17	IV	-13.16	-72.28	3727	yes	yes	yes	2	4
FZ178	myrtilloides	17	IV	-13.16	-72.28	3697	yes	yes	no	2	4
FZ181	myrtilloides	17	IV	-13.18	-72.29	3566	yes	yes	no	2	4
FZ184	myrtilloides	17	IV	-13.18	-72.29	3566	yes	yes	no	2	4
FZ192	myrtilloides	17	IV	-13.20	-71.64	3445	yes	yes	no	2	2
FZ193	myrtilloides	17	IV	-13.20	-71.64	3445	yes	yes	yes	2	2
FZ195	myrtilloides	17	IV	-13.20	-71.64	3448	yes	yes	no	2	2
FZ197	myrtilloides	17	IV	-13.20	-71.62	3490	yes	yes	no	2	4
FZ213	myrtilloides	17	IV	-6.76	-77.89	3398	yes	no		2	unk
FZ219	myrtilloides	17	IV	-6.74	-77.88	3600	yes	yes	yes	2	1
FZ252	myrtilloides	17	IV	-5.38	-79.56	2351	yes	yes	yes	2	1
FZ253	myrtilloides	17	IV	-5.38	-79.56	2360	yes	yes	no	2	1
FZ263	myrtilloides	17	IV	-16.31	-67.92	3416	yes	yes	yes	2	2
FZ275	myrtilloides	17	IV	-17.74	-65.51	3676	yes	no		2	unk
FZ318	myrtilloides	17	IV	-16.20	-68.12	3830	yes	yes	yes	2	6
FZ328	myrtilloides	17	IV	-16.39	-71.44	3827	yes	yes	yes	2	5
GD10288	myrtilloides	17	IV	8.81	-82.54	3100	yes	no		2	unk
GL3124	myrtilloides	17	IV	-3.72	-79.34	3550	yes	no		2	unk
GL3349	myrtilloides	17	IV	-4.11	-79.18	3000	yes	no		2	unk
HS3834	myrtilloides	17	IV	-22.28	-64.87	3550	yes	no		2	unk
IH8484	myrtilloides	17	IV	-14.69	-71.28	3800	yes	no		2	unk
IS4874	myrtilloides	17	IV	-6.26	-78.70	2500	yes	no		2	unk
JB42556	myrtilloides	17	IV	0.23	-78.17	3250	yes	no		2	unk
JB42865	myrtilloides	17	IV	-1.12	-78.92	3590	yes	no		2	unk
JC23138	myrtilloides	17	IV	4.80	-75.42	3800	yes	no		2	unk
JC24502	myrtilloides	17	IV	10.78	-73.50	3260	yes	no		2	unk
JC25054	myrtilloides	17	IV	10.36	-72.95	3100	yes	no		2	unk
JC27370	myrtilloides	17	IV	2.83	-76.20	3800	yes	no		2	unk

JL12782	myrtilloides	17	IV	1.12	-77.67	3750	yes	no		2	unk
JL13004	myrtilloides	17	IV	4.70	-75.33	3700	yes	no		2	unk
JS17503	myrtilloides	17	IV	-16.58	-67.72	3700	yes	no		2	unk
JS52404	myrtilloides	17	IV	-0.21	-78.55	3400	yes	no		2	unk
JW7766	myrtilloides	17	IV	-18.97	-65.40	3400	yes	yes	yes	2	6
LA2584	myrtilloides	17	IV	8.59	-71.00	3200	yes	no		2	unk
LV5937	myrtilloides	17	IV	-12.55	-72.00	3000	yes	no		2	unk
LV854	myrtilloides	17	IV	-12.94	-72.84	3400	yes	no		2	unk
MD2942	myrtilloides	17	IV	-7.20	-78.57	3550	yes	no		2	unk
ML38831	myrtilloides	17	IV	-16.80	-67.28	3300	yes	no		2	unk
NL221	myrtilloides	17	IV	-17.79	-64.72	2900	yes	no		2	unk
NR2822	myrtilloides	17	IV	-17.27	-66.33	3650	yes	yes	no	2	6
PG560	myrtilloides	17	IV	6.42	-72.30	2900	yes	no		2	unk
PHV7207	myrtilloides	17	IV	-17.67	-70.00	3370	yes	no		2	unk
PJ92172	myrtilloides	17	IV	-4.73	-79.42	3450	yes	no		2	unk
RD2188	myrtilloides	17	IV	8.59	-71.02	3400	yes	no		2	unk
RG102	myrtilloides	17	IV	9.88	-83.91	2520	yes	no		2	unk
RO334	myrtilloides	17	IV	10.12	-84.10	2900	yes	yes	no	2	1
RW10639	myrtilloides	17	IV	10.19	-84.23	2700	yes	no		2	unk
TN78	myrtilloides	17	IV	-0.03	-78.25	3750	yes	no		2	unk
WD1937	myrtilloides	17	IV	3.80	-75.93	3300	yes	no		2	unk
WF547	myrtilloides	17	IV	-13.26	-71.61	3650	yes	no		2	unk
BH23603	myrtilloides	17	IV	9.60	-83.83	2850		yes	no	unk	1
FZ130	myrtilloides		IV	5.90	-73.08	3511		yes	no	unk	1
FZ131	myrtilloides		IV	5.91	-73.08	3509		yes	no	unk	1
FZ132	myrtilloides		IV	5.90	-73.06	3678		yes	no	unk	1
FZ134	myrtilloides		IV	5.93	-73.08	3698		yes	no	unk	1
FZ154	myrtilloides		IV	5.93	-73.08	3718		yes	no	unk	1
FZ168	myrtilloides	17	IV	-14.06	-71.34	3806		yes	no	unk	4
FZ171	myrtilloides	17	IV	-14.07	-71.34	3796		yes	no	unk	4
FZ172	myrtilloides	17	IV	-14.07	-71.34	3796		yes	no	unk	4
FZ179	myrtilloides	17	IV	-13.16	-72.28	3694		yes	no	unk	4
FZ194	myrtilloides	17	IV	-13.20	-71.64	3445		yes	no	unk	2
FZ201	myrtilloides	17	IV	-13.20	-71.61	3489		yes	no	unk	2
FZ250	myrtilloides	17	IV	-5.38	-79.56	2353		yes	no	unk	1
FZ321	myrtilloides	17	IV	-16.11	-68.07	3835		yes	no	unk	6
FZ86	myrtilloides	17	IV	5.00	-74.18	3197		yes	yes	unk	1
FZ87	myrtilloides		IV	4.57	-74.00	3360		yes	no	unk	1
FZ89	myrtilloides		IV	4.59	-74.00	2660		yes	no	unk	1

3.0.2 Table S2: Model Adequacy

Table S2: Kolmogorov's D Statistic and associated p-values calculated for each genogroup identified within each clade.

Clade	Genogroup	D.statistic	P.value
I	1	0.2370537	0.2251456
I	2	0.3447276	0.3842594
II	1	0.3891588	0.1336416
II	2	0.5908517	0.3348047
II	3	0.3955999	0.4514865
II	4	0.3423166	0.2430962
III	1	0.2488771	0.0852463
III	2	0.3339053	0.4214294
III	3	0.3535451	0.2121071
III	4	0.2008581	0.4080089
III	5	0.1482744	0.7707755
III	6	0.2830028	0.1258734
III	7	0.6147238	0.2968756
IV	1	0.2529787	0.0457106
IV	2	0.2548498	0.4600200
IV	3	0.3440382	0.3865766
IV	4	0.2498736	0.1071131
IV	5	0.6321206	0.7357589
IV	6	0.4468047	0.1304242
V	1	0.1884224	0.0325221
V	2	0.1865531	0.8163975
V	3	0.2694986	0.0046801
V	4	0.3176207	0.7145160
V	5	0.6321206	0.7357589
V	6	0.3354475	0.2637089
V	7	0.3437764	0.0905212
V	8	0.6321206	0.7357589
V	9	0.2616221	0.3252607
V	10	0.1261724	0.3082440
VI	1	0.2325980	0.5746831
VI	2	0.3919480	0.4633090
VI	3	0.3390222	0.1579317
VI	4	0.2350191	0.0311922
VI	5	0.2516498	0.1718372
VI	6	0.3422221	0.1506186
VI	7	0.2696386	0.1203399
VI	8	0.2553783	0.2083885
VI	9	0.4487310	0.2961447
VI	10	0.3167529	0.2167244
VI	11	0.2997568	0.5560520

3.1 Clade I

Table S3: Counts of specimens, loci, and sites associated with each data matrix.

	Specimens	Loci	Sites
**iPyrad Assembly**			
total prefiltered loci	14	44630	-
total filtered loci	14	22999	82447
specimens with >= 95% missing data	0	-	-
**Filtering with VCFTOOLS**			
largest data matrix (75% missing data)	14	17422	80740
middle sized data matrix (50% missing data)	14	12503	62092
smallest data matrix (25% missing data)	14	5618	31847
most informative loci (via gCF/sCF analyses)	14	4974	-
loci used for BFD*	6	2320	-

3.1.0.1 Table S2: Genomic dataset details Return to Clade I Genomics

Table S4: Genomic modeling for genogroup delimitation and model selection using Bayes factors (BF)

Clade	Number.of.loci	Model	Genogroups	Marginal.likelihood	Rank	Bayes.Factor
I	2320	GC	3	-6580.495	1	-
		RI[a]/RI[b]/CA	2	-6754.495	2	348

[a] specimens assigned to demes using MAVERICK
[b] specimens assigned to demes using STRUCTURE

3.1.0.2 Table S3: Genogroup delimitation Return to Clade I Model-based species discovery

Table S5: Correspondence between taxonomic species and best-fit phenogroups and genogroups. Shaded cells show specimens assigned to a particular combination of taxonomic species, best fit phenogroup, and best fit genogroup.

<table>
<tr><th></th><th colspan="2">Phenogroups</th><th colspan="3">Genogroups</th></tr>
<tr><th>taxonomic species</th><th>p1</th><th>p2</th><th>g1</th><th>g2</th><th>g3</th></tr>
<tr><td>micrantha</td><td style="background:#bbb">■</td><td></td><td></td><td></td><td style="background:#bbb">■</td></tr>
<tr><td>millegrana</td><td></td><td style="background:#bbb">■</td><td style="background:#bbb">■</td><td style="background:#bbb">■</td><td></td></tr>
</table>

3.1.0.3 Table S4: Correspondence between taxonomic species, phenogroups, and genogroups

Return to Clade I Correspondence between taxonomic species and model-based species

3.2 Clade II

Table S6: Counts of specimens, loci, and sites associated with each data matrix.

	Specimens	Loci	Sites
**iPyrad Assembly**			
total prefiltered loci	15	66064	
total filtered loci	15	30440	157469
specimens with >= 95% missing data	0	-	-
**Filtering with VCFTOOLS**			
largest data matrix (75% missing data)	15	26466	153826
middle sized data matrix (50% missing data)	15	15284	99103
smallest data matrix (25% missing data)	15	8086	53485
most informative loci (via gCF/sCF analyses)	15	6917	-
loci used for BFD*	8	3005	-

3.2.0.1 Table S5: Genomic dataset details Return to Clade II Genomics

Table S7: Genomic modeling for genogroup delimitation and model selection using Bayes factors (BF)

Clade	Number.of.loci	Model	Genogroups	Marginal.likelihood	Rank	Bayes.Factor
II	3005	CA	4	-13460.92	1	-
		RI[a]/GC	3	-15036.44	2	3151.0418
		RI[b]	2	-18963.34	3	11004.85

[a] specimens assigned to demes using MAVERICK
[b] specimens assigned to demes using STRUCTURE

3.2.0.2 Table S6: Genogroup delimitation

Return to Clade II Model-based species discovery

Table S8: Correspondence between taxonomic species and best-fit phenogroups and genogroups. Shaded cells show specimens assigned to a particular combination of taxonomic species, best fit phenogroup, and best fit genogroup.

	Phenogroups			Genogroups			
taxonomic species	p1	p2	p3	g1	g2	g3	g4
herrerae	■			■			
pendula	■	■	■		■	■	■

3.2.0.3 Table S7: Correspondence between taxonomic species, phenogroups, and genogroups Return to Clade II Correspondence between taxonomic species and model-based species

3.3 Clade III

Table S9: Counts of specimens, loci, and sites associated with each data matrix.

	Specimens	Loci	Sites
**iPyrad Assembly**			
total prefiltered loci	53	91032	
total filtered loci	53	43597	284453
specimens with >= 95% missing data	0	-	-
**Filtering with VCFTOOLS**			
largest data matrix (75% missing data)	53	15690	161981
middle sized data matrix (50% missing data)	53	6724	82514
smallest data matrix (25% missing data)	53	3150	38903
most informative loci (via gCF/sCF analyses)	53	3084	-
loci used for BFD*	15	1993	-

3.3.0.1 Table S8: Genomic dataset details Return to Clade III Genomics

Table S10: Genomic modeling for genogroup delimitation and model selection using Bayes factors (BF)

Clade	Number.of.loci	Model	Genogroups	Marginal.likelihood	Rank	Bayes.Factor
III	1993	CA	7	-8985.782	1	-
		RI[a]	5	-10014.260	2	2056.9554
		RI[b]/GC	3	-12233.131	3	6494.6984

[a] specimens assigned to demes using MAVERICK
[b] specimens assigned to demes using STRUCTURE

3.3.0.2 Table S9: Genogroup delimitation Return to Clade III Model-based species discovery

Table S11: Correspondence between taxonomic species and best-fit phenogroups and genogroups. Shaded cells show specimens assigned to a particular combination of taxonomic species, best fit phenogroup, and best fit genogroup.

	Phenogroups					Genogroups						
taxonomic species	p1	p2	p3	p4	p5	g1	g2	g3	g4	g5	g6	g7
discolor				■				■				
paniculata	■		■	■						■		■
piurensis		■				■						
resinosa		■				■	■					
reticulata			■								■	
schreiteri					■				■			

3.3.0.3 Table S10: Correspondence between taxonomic species, phenogroups, and genogroups

Return to Clade III Correspondence between taxonomic species and model-based species

3.4 Clade IV

Table S12: Counts of specimens, loci, and sites associated with each data matrix.

	Specimens	Loci	Sites
**iPyrad Assembly**			
total prefiltered loci	43	79865	
total filtered loci	43	31840	205346
specimens with >= 95% missing data	1	-	-
**Filtering with VCFTOOLS**			
largest data matrix (75% missing data)	42	14260	123096
middle sized data matrix (50% missing data)	42	7019	69598
smallest data matrix (25% missing data)	42	3762	38404
most informative loci (via gCF/sCF analyses)	42	3337	-
loci used for BFD*	12	2245	-

3.4.0.1 Table S11: Genomic dataset details Return to Clade IV Genomics

Table S13: Genomic modeling for genogroup delimitation and model selection using Bayes factors (BF)

Clade	Number.of.loci	Model	Genogroups	Marginal.likelihood	Rank	Bayes.Factor
IV	2245	CA	6	-9601.514	1	-
		GC	3	-11546.649	2	3890.2706
		RI[a]/RI[b]	2	-12017.878	3	4832.7284

[a] specimens assigned to demes using MAVERICK
[b] specimens assigned to demes using STRUCTURE

3.4.0.2 Table S12: Genogroup delimitation Return to Clade IV Model-based species discovery

Table S14: Correspondence between taxonomic species and best-fit phenogroups and genogroups. Shaded cells show specimens assigned to a particular combination of taxonomic species, best fit phenogroup, and best fit genogroup.

	Phenogroups		Genogroups					
taxonomic species	p1	p2	g1	g2	g3	g4	g5	g6
myrtilloides								
polifolia								

3.4.0.3 Table S13: Correspondence between taxonomic species, phenogroups, and genogroups

Return to Clade IV Correspondence between taxonomic species and model-based species

3.5 Clade V

Table S15: Counts of specimens, loci, and sites associated with each data matrix.

	Specimens	Loci	Sites
**iPyrad Assembly**			
total prefiltered loci	112	133181	
total filtered loci	112	50898	325996
specimens with >= 95% missing data	3	-	-
**Filtering with VCFTOOLS**			
largest data matrix (75% missing data)	109	9843	108815
middle sized data matrix (50% missing data)	109	3818	46136
smallest data matrix (25% missing data)	109	1154	15311
most informative loci (via gCF/sCF analyses)	109	1015	-
loci used for BFD*	26	742	-

3.5.0.1 Table S14: Genomic dataset details Return to Clade V Genomics

Table S16: Genomic modeling for genogroup delimitation and model selection using Bayes factors (BF)

Clade	Number.of.loci	Model	Genogroups	Marginal.likelihood	Rank	Bayes.Factor
V	742	CA	10	-4588.693	1	-
		GC	6	-5381.361	2	1585.3362
		RI[a]	3	-5601.058	3	2024.7296
		RI[b]	2	-6085.998	4	2994.61

[a] specimens assigned to demes using MAVERICK
[b] specimens assigned to demes using STRUCTURE

3.5.0.2 Table S15: Genogroup delimitation

Return to Clade V Model-based species discovery

Table S17: Correspondence between taxonomic species and best-fit phenogroups and genogroups. Shaded cells show specimens assigned to a particular combination of taxonomic species, best fit phenogroup, and best fit genogroup.

	Phenogroups								Genogroups									
taxonomic species	p1	p2	p3	p4	p5	p6	p7	p8	g1	g2	g3	g4	g5	g6	g7	g8	g9	g10
alpina																		
florida																		
leucantha																		
myrtoidea																		
revoluta																		
rosea																		
rubra																		

3.5.0.3 Table S16: Correspondence between taxonomic species, phenogroups, and genogroups

Return to Clade V Correspondence between taxonomic species and model-based species

3.6 Clade VI

Table S18: Counts of specimens, loci, and sites associated with each data matrix.

	Specimens	Loci	Sites
**iPyrad Assembly**			
total prefiltered loci	91	133123	
total filtered loci	91	49105	353116
specimens with >= 95% missing data	9	-	-
**Filtering with VCFTOOLS**			
largest data matrix (75% missing data)	82	9174	138001
middle sized data matrix (50% missing data)	82	4027	68198
smallest data matrix (25% missing data)	82	1641	28865
most informative loci (via gCF/sCF analyses)	82	1607	-
loci used for BFD*	22	915	-

3.6.0.1 Table S17: Genomic dataset details Return to Clade VI Genomics

Table S19: Genomic modeling for genogroup delimitation and model selection using Bayes factors (BF)

Clade	Number.of.loci	Model	Genogroups	Marginal.likelihood	Rank	Bayes.Factor
VI	915	CA	11	-2921.024	1	-
		GC	7	-3627.806	2	1413.5644
		RI[a]/RI[b]	4	-4661.351	3	3480.6544

[a] specimens assigned to demes using MAVERICK
[b] specimens assigned to demes using STRUCTURE

3.6.0.2 Table S18: Genogroup delimitation Return to Clade VI Model-based species discovery

Table S20: Correspondence between taxonomic species and best-fit phenogroups and genogroups. Shaded cells show specimens assigned to a particular combination of taxonomic species, best fit phenogroup, and best fit genogroup.

	Phenogroups								Genogroups										
taxonomic species	p1	p2	p3	p4	p5	p6	p7	p8	g1	g2	g3	g4	g5	g6	g7	g8	g9	g10	g11
angustifolia	■							■		■	■								
bifida		■											■						
farinacea					■										■				
hypoglauca	■			■								■							
illinita				■			■	■	■	■									
laevis	■			■														■	■
ledifolia								■								■			
megapotamica			■			■								■					
petrophila	■																■		
tucumanensis	■			■								■							

3.6.0.3 Table S19: Correspondence between taxonomic species, phenogroups, and genogroups Return to Clade VI Correspondence between taxonomic species and model-based species

4 Figures

4.1 Species Trees

4.1.1 Fig S1: Phylogenetic trees (two specimens per taxonomic species)

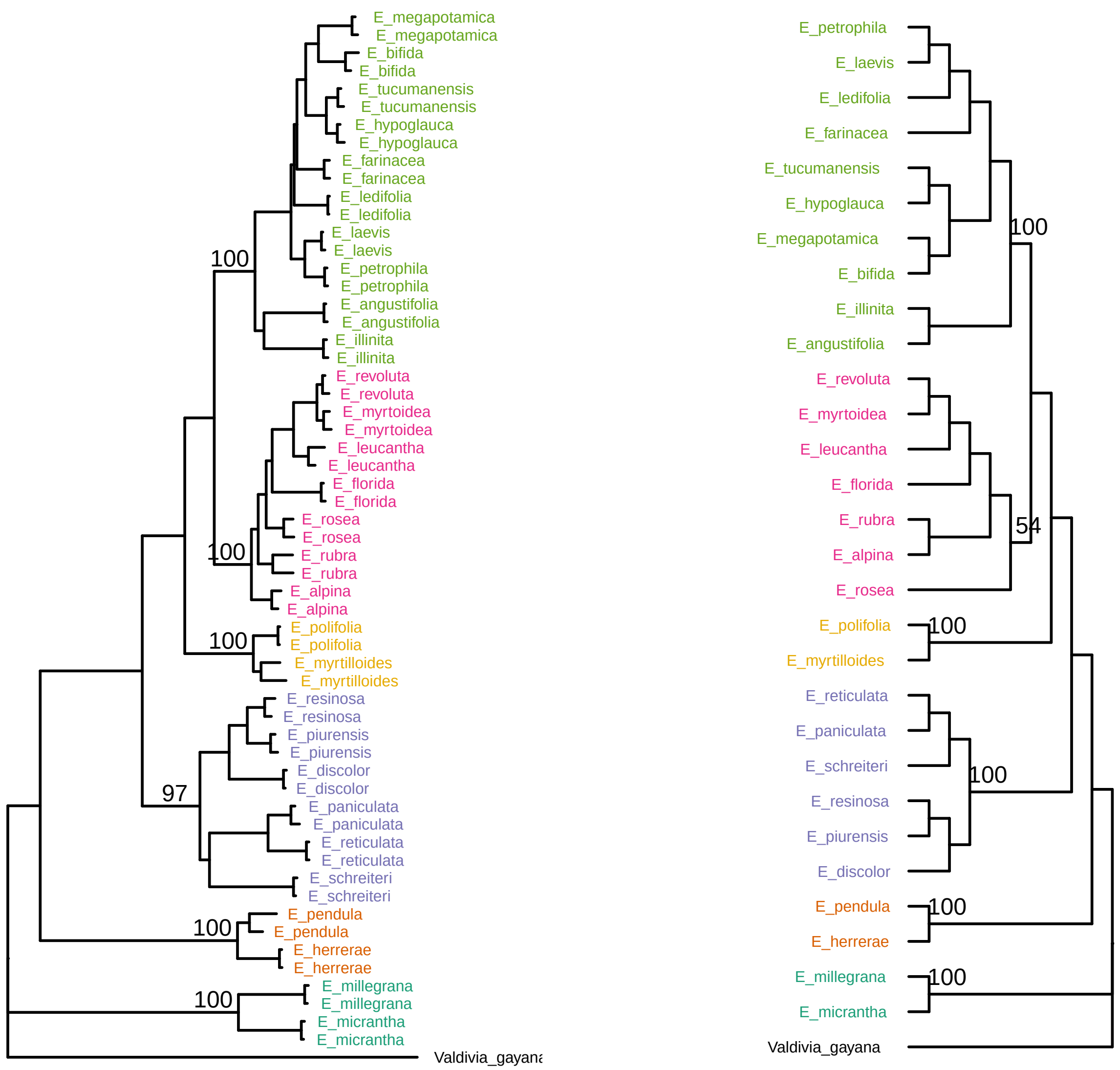

Figure S1: Phylogenetic trees of *Escallonia*. Left: Maximum likelihood tree using two specimens per taxonomic species and 364 concatenated loci. Right: Quartet-based species tree. In both trees, colors indicate clades I to VI from bottom to top. Bootstrap support values for focal clades is shown above corresponding bipartition.

Return to Current state of taxonomic species using genomics data

4.1.2 Fig S2: Phylogenetic trees (four specimens per taxonomic species)

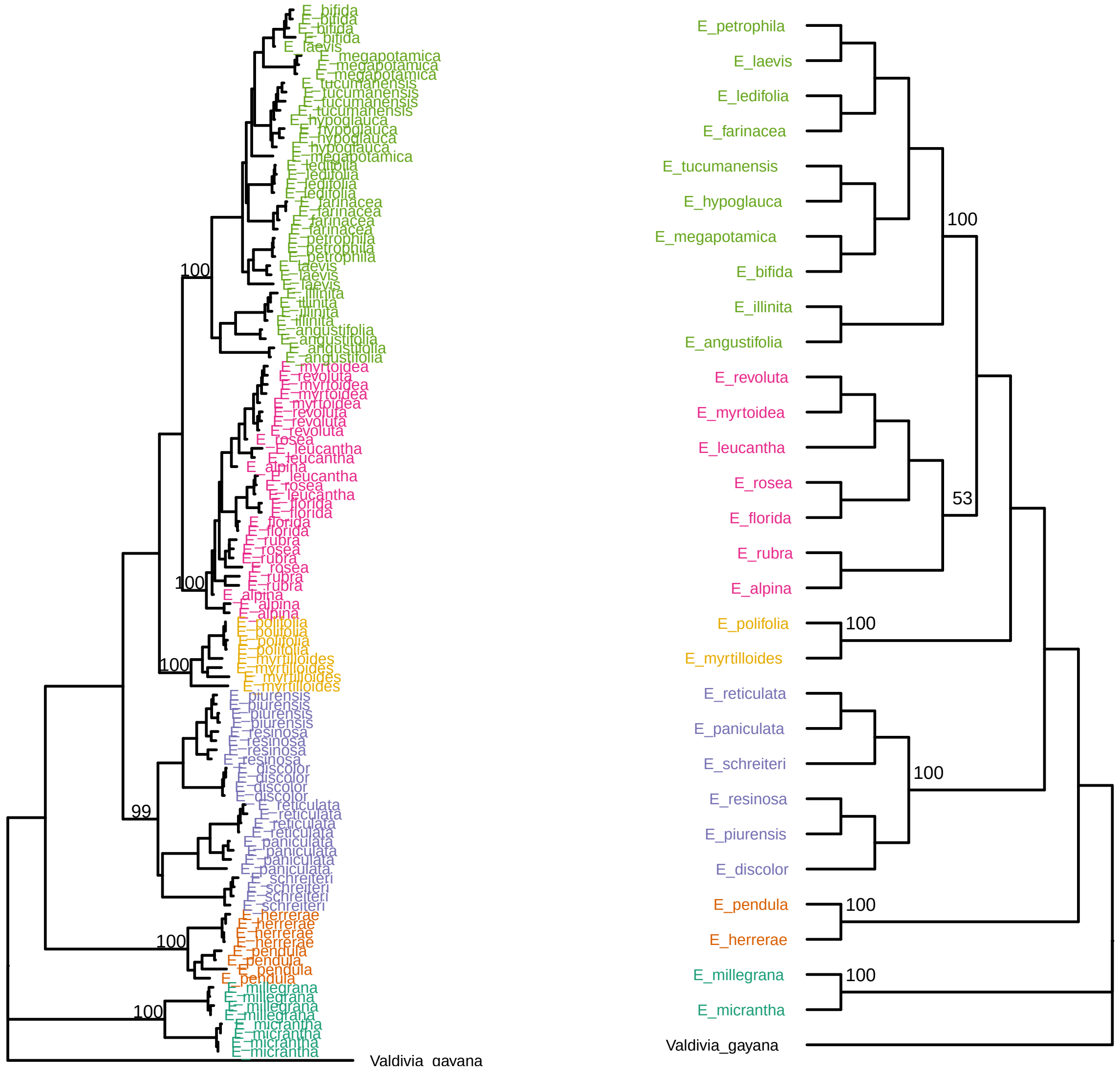

Figure S2: Phylogenetic trees of *Escallonia*. Maximum likelihood tree using four specimens per taxonomic species and 181 concatenated loci (left). Quartet-based species tree (right). In both trees, colors indicate clades I to VI from bottom to top. Bootstrap support values for focal clades is presented (all other bipartitions were also well-supported)

Return to Current state of taxonomic species using genomics data

4.2 Clade I

4.2.1 Fig S3: Taxon sampling

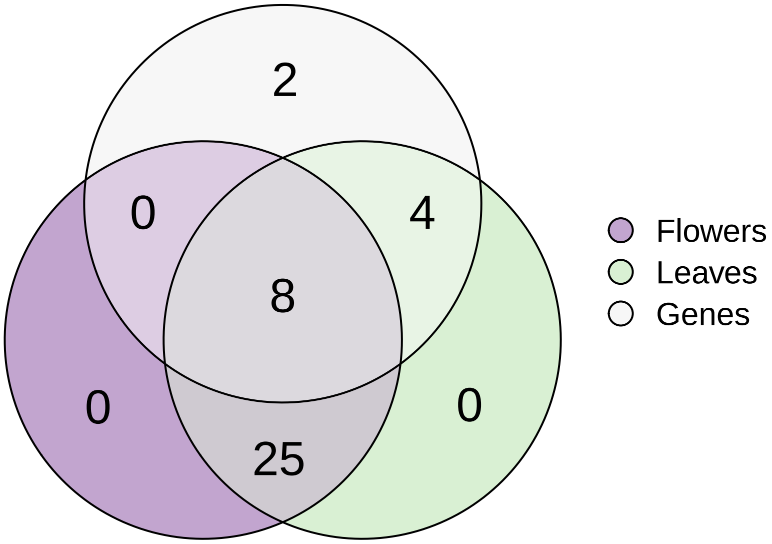

Figure S3: Specimens sampled according to three types of data. Specimens outside the Flowers category represent sterile specimens.

Return to Clade I Sampling

4.2.2 Fig S4: Geographic distribution

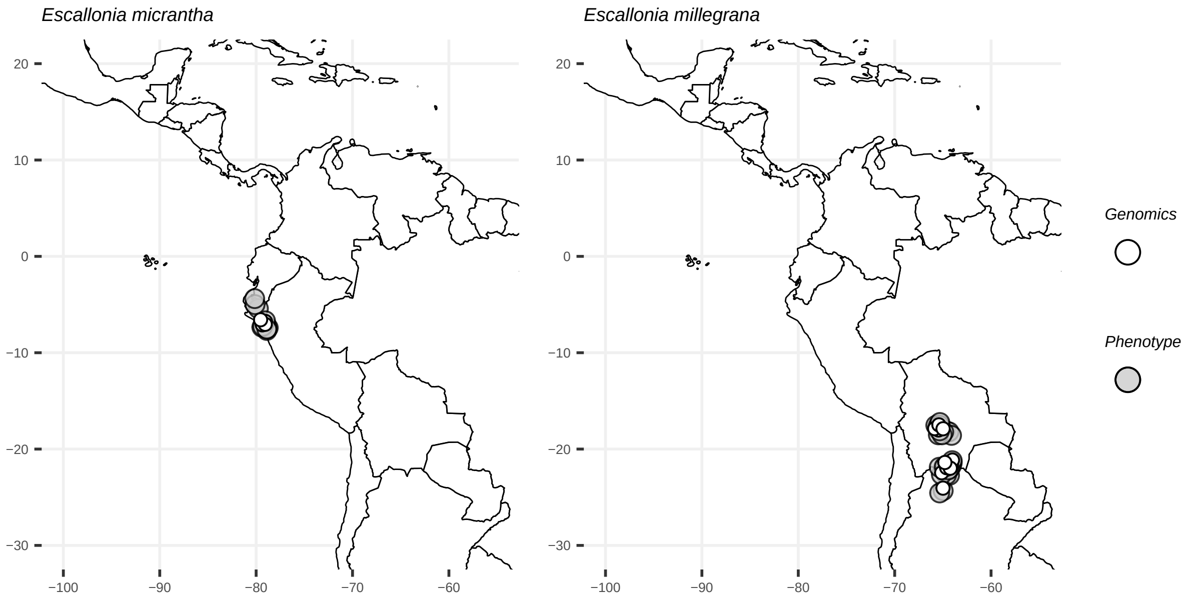

Figure S4: Geographic distribution of specimens sampled for this clade according to taxonomic species. Filled symbols indicate specimens used in phenotypic analyses and empty symbols specimens used in genomic analyses.

Return to Clade I Sampling

4.2.3 Fig S5: Current state of taxonomic species with phenotypic data

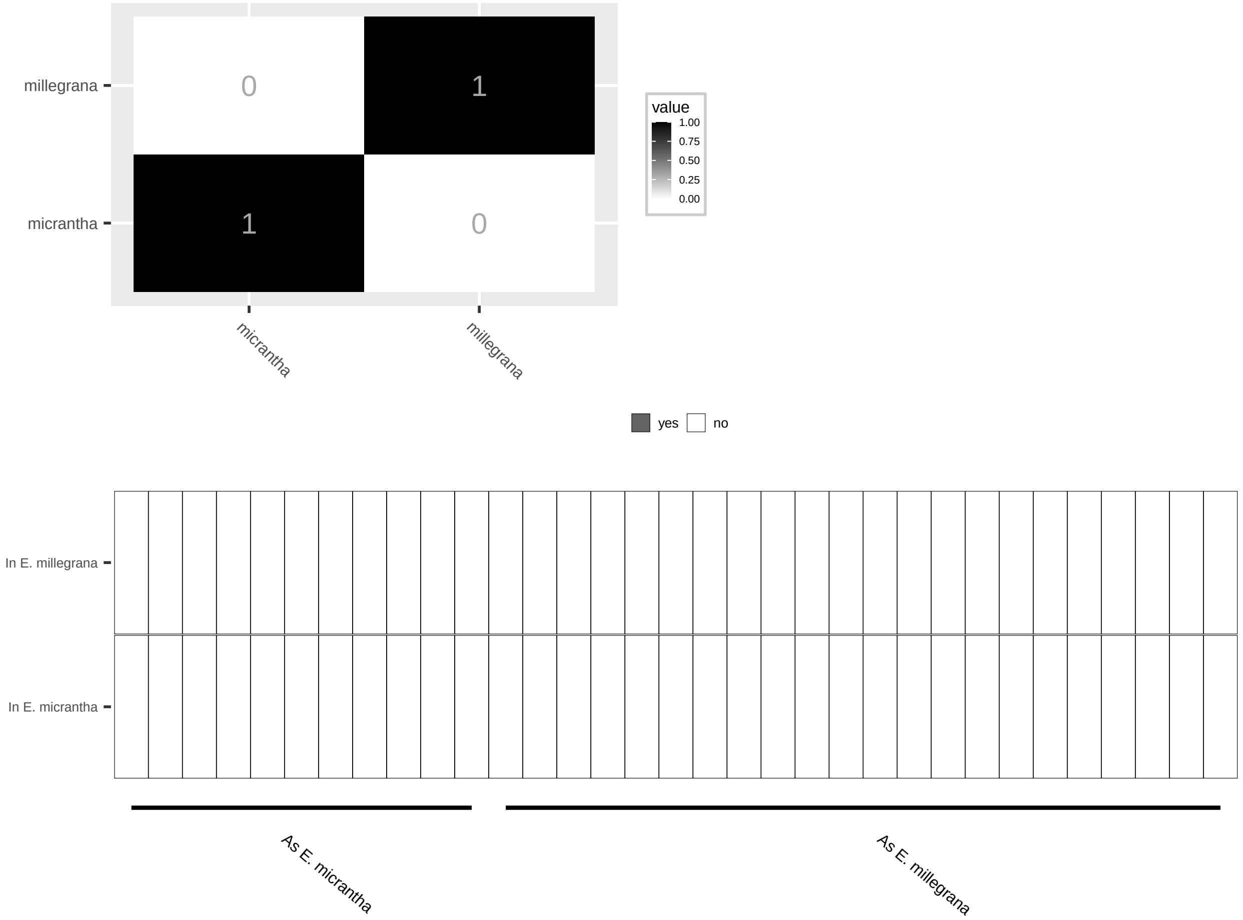

Figure S5: Assessment of current state of taxonomic species with phenotypic data. Top panel: Pairwise overlap among 10-cubes describing geometrically each taxonomic species in 10-dimensional phenospace. Bottom panel: Matching-prediction analysis with each cell along the x-axis representing specimens sorted according to taxonomic species and the 10-cubes corresponding to each taxonomic species along the y-axis. If a specimen matches the prediction of the monograph (i.e., it is inside a 10-cube), the corresponding cell is shaded. If the specimen does not match the prediction, the cell is empty.

Return to Clade I Current state of taxonomic species

4.2.4 Fig S6: Phenogroup delimitation: Gaussian finite mixture modeling

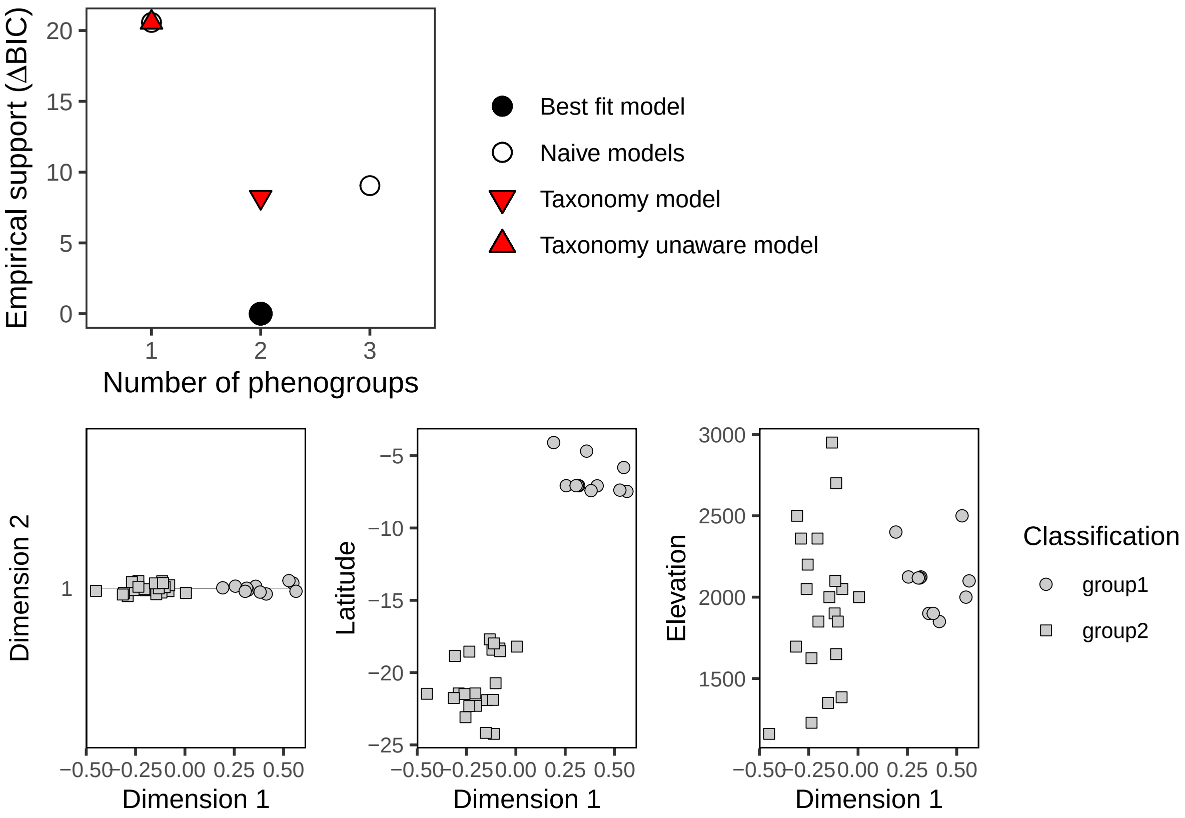

Figure S6: Gaussian finite mixture modeling (GFMM) for phenogroup delimitation and model selection using the Bayesian information criterion (BIC). Top panel: empirical support (ordinate) for Gaussian mixture models (GMM) assuming distinct number of phenogroups (abscissa). Each GMM specifies different number of phenogroups (shapes). Empirical support was measured as difference in BIC relative to the best model (ΔBIC $= 0$). Bottom panel: Visualization of the phenogroups (shapes) identified by the best fit GMM; left panel shows phenogroups in the space defined by two axes obtained by linear discriminat analysis (to maximize separation and visualization), middle panel shows phenogroups in the space defined by discriminant axis 1 and latitude, and right panel shows phenogroups in the space defined by discriminant axis 1 and elevation.

Return to Clade I Phenomics: model-based species discovery

4.2.5 Fig S7: Sensitivity tests with 75% missing data

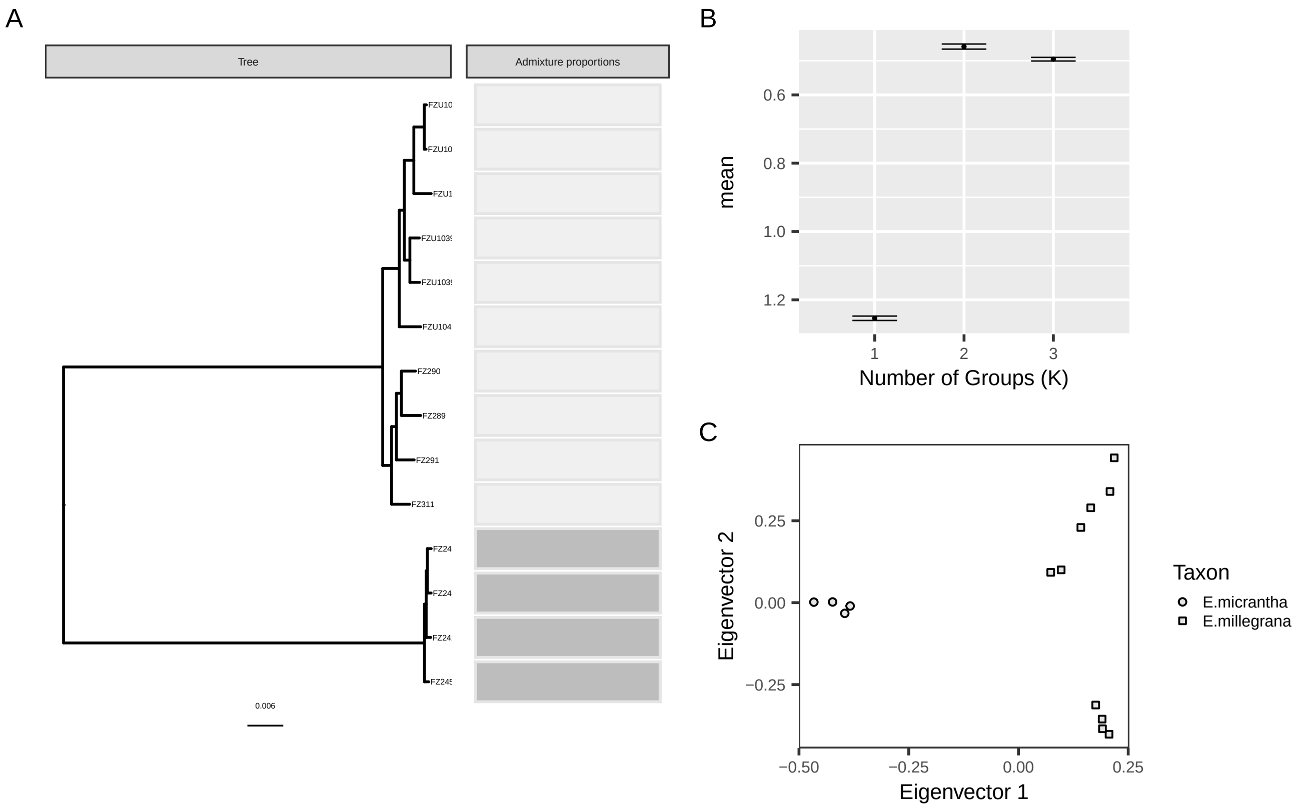

Figure S7: Impact of missing data (75%) on data analysis. A) maximum likelihood phylogenetic tree of specimens assigned to demes according to best `ADMIXTURE` run. B) Mean cross validation error with 95% confidence interval (ordinate) for ten replicate runs of `ADMIXTURE` assuming differnt number of demes (K) (abscissa); C) Scatterplot of Principal Component Analysis (PCA) projected along the first two axes.

Return to Clade I Genomics: sensitivity tests

4.2.6 Fig S8: Sensitivity tests with 50% missing data

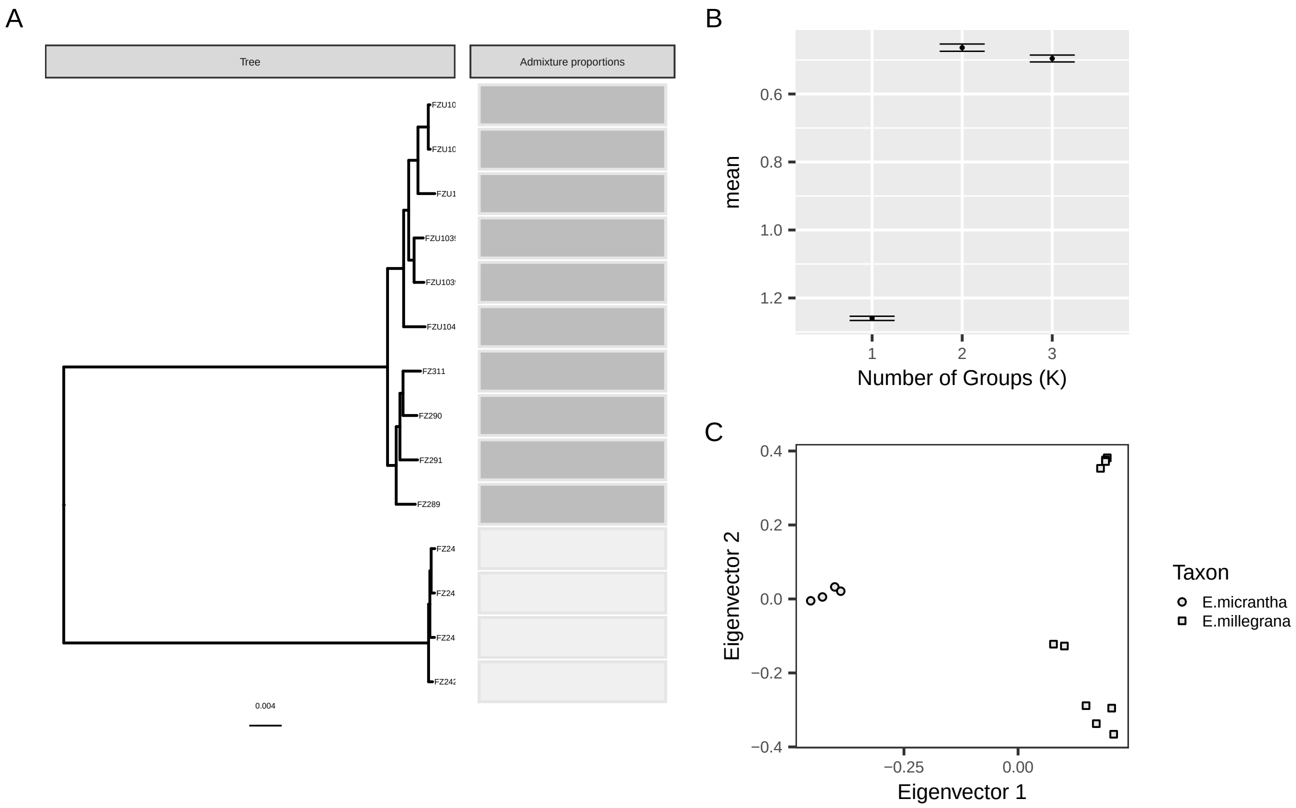

Figure S8: Impact of missing data (50%) on data analysis. A) maximum likelihood phylogenetic tree of specimens assigned to demes according to best `ADMIXTURE` run. B) Mean cross validation error with 95% confidence interval (ordinate) for ten replicate runs of `ADMIXTURE` assuming differnt number of demes (K) (abscissa); C) Scatterplot of Principal Component Analysis (PCA) projected along the first two axes.

Return to Clade I Genomics: sensitivity tests

4.2.7 Fig S9: Sensitivity tests with 25% missing data

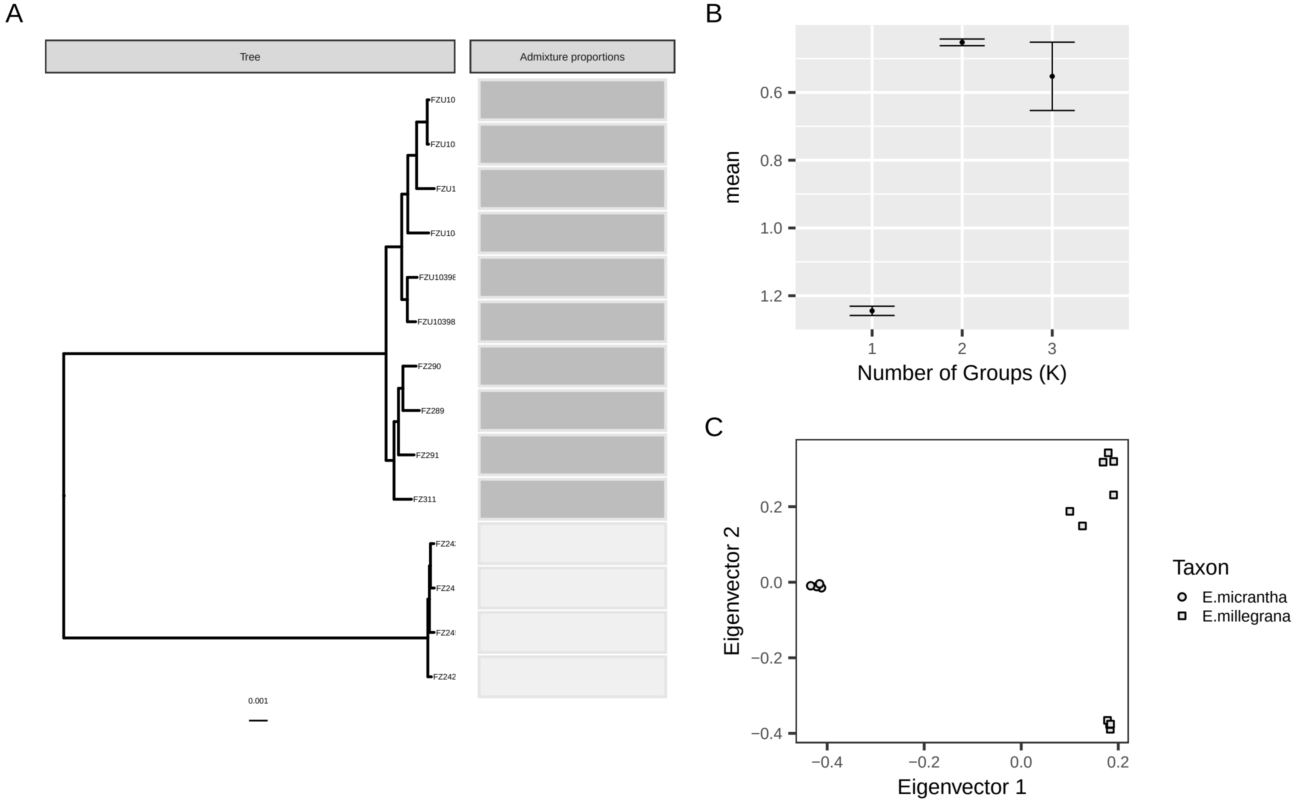

Figure S9: Impact of missing data (25%) on data analysis. A) maximum likelihood phylogenetic tree of specimens assigned to demes according to best `ADMIXTURE` run. B) Mean cross validation error with 95% confidence interval (ordinate) for ten replicate runs of `ADMIXTURE` assuming differnt number of demes (K) (abscissa); C) Scatterplot of Principal Component Analysis (PCA) projected along the first two axes.

Return to Clade I Genomics: sensitivity tests

4.2.8 Fig S10: Genogroup delimitation: Genotypic cluster model

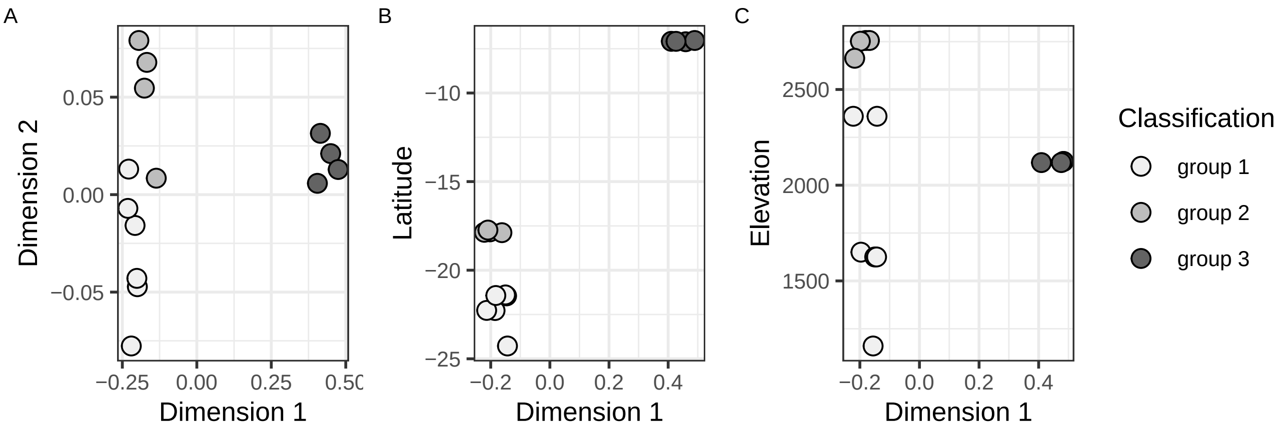

Figure S10: Gaussian finite mixture modeling (GFMM) for genogroup delimitation. Visualization of the genogroups (shades) identified by the best fit Gaussian mixture model (GMM). A) genogroups in the space defined by two axes obtained by non-metric multidimensional scaling (NMDS); B) genogroups in the space defined by NMDS axis 1 and latitude; C) genogroups in the space defined by NMDS axis 1 and elevation.

Return to Clade I Genomics: model-based species discovery

4.2.9 Fig S11: Genogroup delimitation. Cladogenesis to anagenesis model

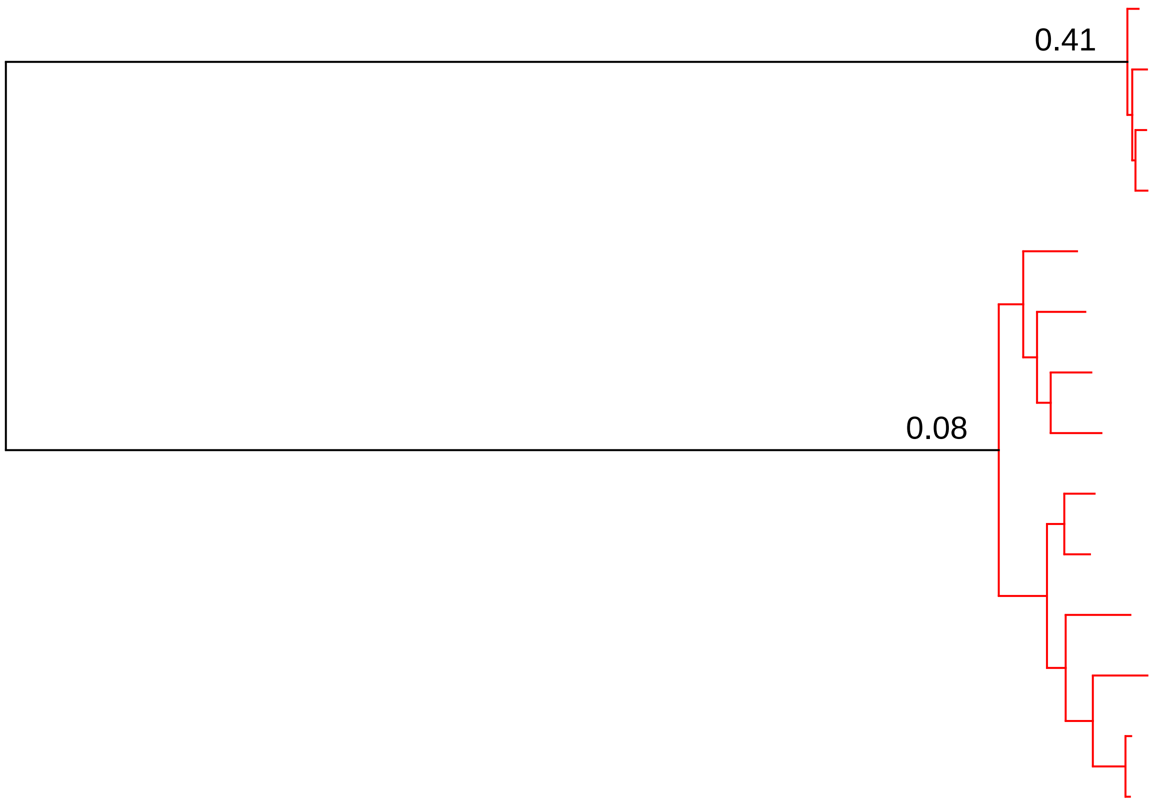

Figure S11: Phylogenetic modeling for genogroup delimitation. Midpoint-rooted phylogenetic tree showing genogroups in red. Values correspond to nodes at the transition point between cladogenesis (between species) to anagenesis (within species). Values closer to 0 indicate that the node was identified as a transition to anagenesis summarized over 500 delimitations.

Return to Clade I Genomics: model-based species discovery

4.2.10 Fig S12: Genogroup delimitation: Reproductive isolation model

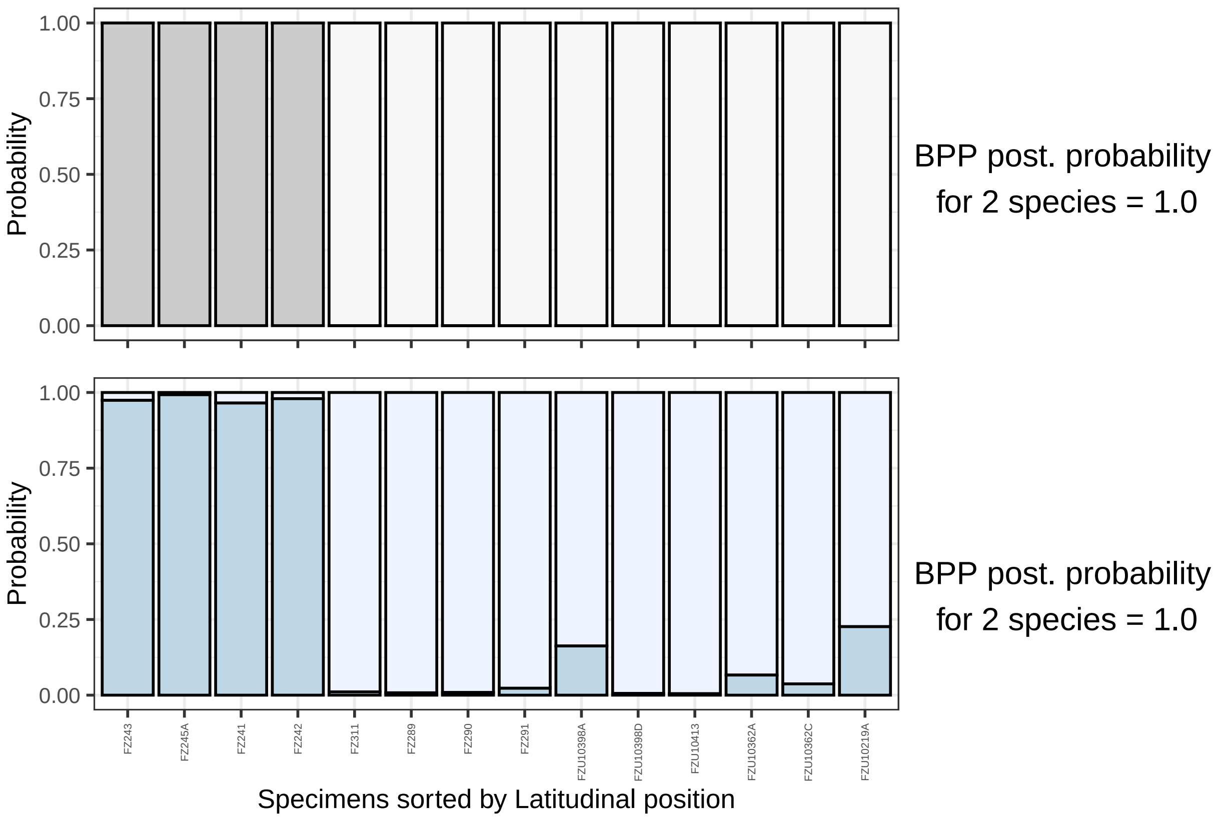

Figure S12: Population genetic modeling for genogroup delimitation. Top panel: assignment of specimens to demes according to `STRUCTURE` and posterior probability of species delimitation modeling according to `BPP` using these demes. Bottom panel: assignment of specimens to demes according to `MAVERICK` and posterior probability of species delimitation modeling according to `BPP` using these demes. Specimens are sorted from north (left) to south (right) according to locality of collection.

Return to Clade I Genomics: model-based species discovery

4.2.11 Fig S13: Data integration

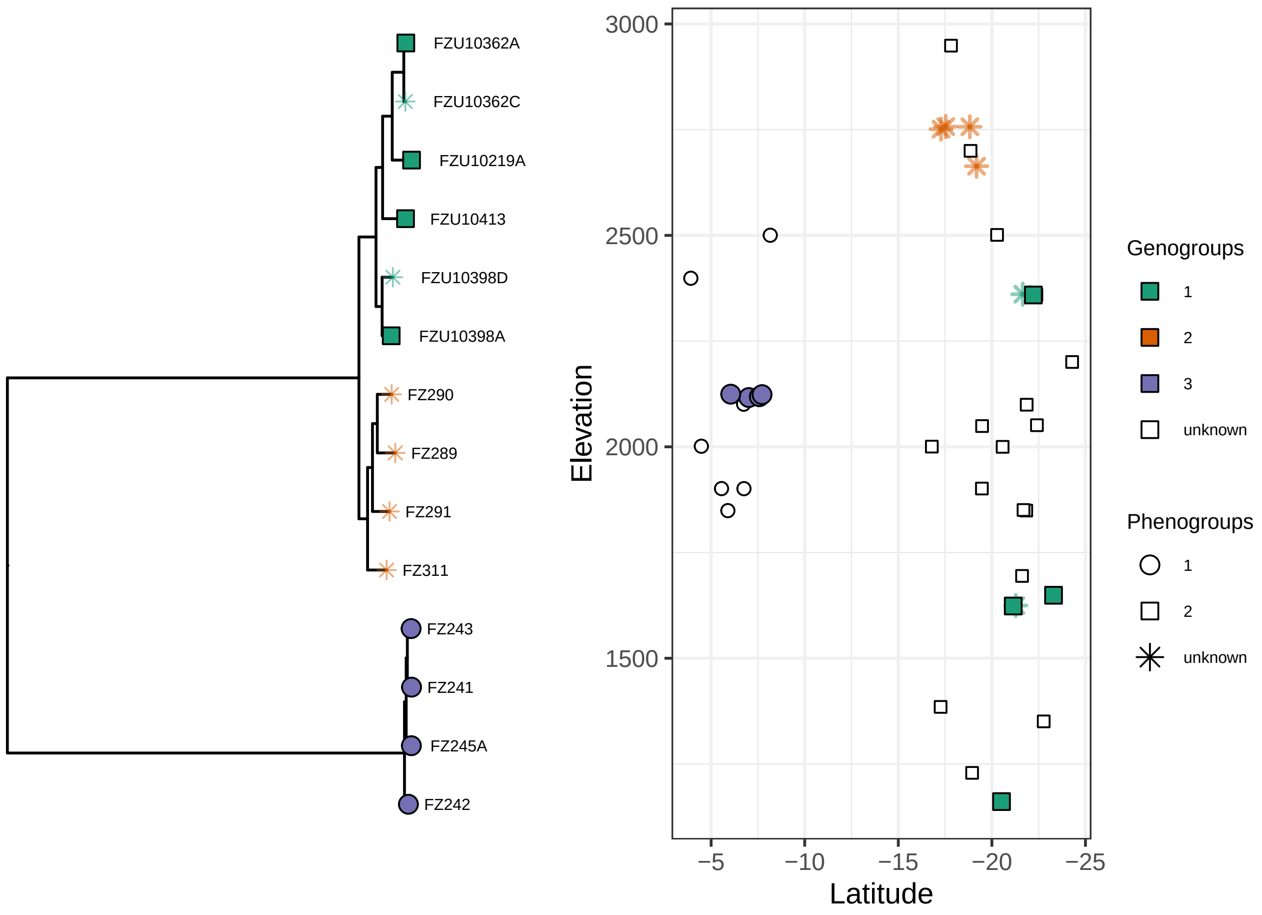

Figure S13: Integration of phenotypic and genomic data with spatial information and evolutionary history. All specimens are assigned to their corresponding best fit phenogroup (shapes) and genogroup (colors). Specimens without phenotypic or genomic data (unknown specimens) are shown as asterisks and empty shapes, accordingly. Specimens are shown as tips of the maximum likelihood tree (left) used in the CA model analysis and mapped along latitude and elevation (right). Specimens assigned to a single phenogroup and a single genogroup delineate species that we determined as 'good species'. Specimens assigned to a single phenogroup across multiple genogroups delineate species that we determined as 'phenotypic cryptic species'. Specimens assigned to a single genogroup across multiple phenogroups delineate species that we determined as 'genetic cryptic species'.

Return to Clade I Data integration

4.3 Clade II

4.3.1 Fig S14: Taxon sampling

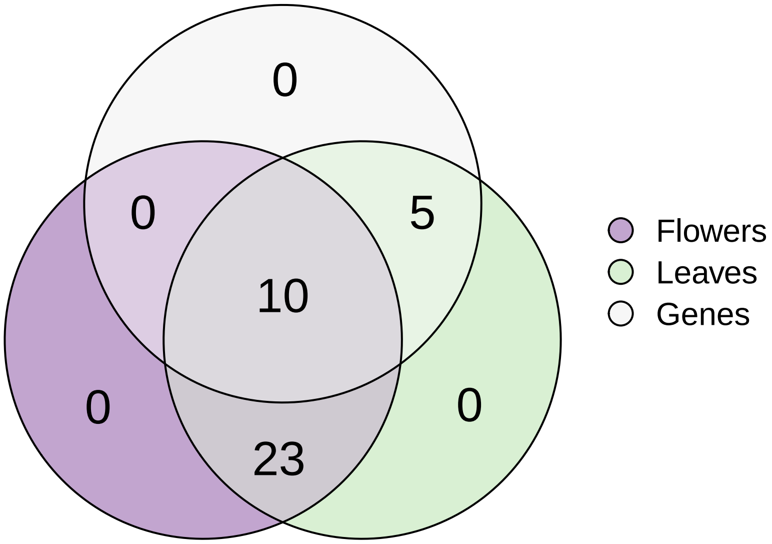

Figure S14: Specimens sampled according to three types of data. Specimens outside the Flowers category represent sterile specimens.

Return to Clade II Sampling

4.3.2 Fig S15: Geographic distribution

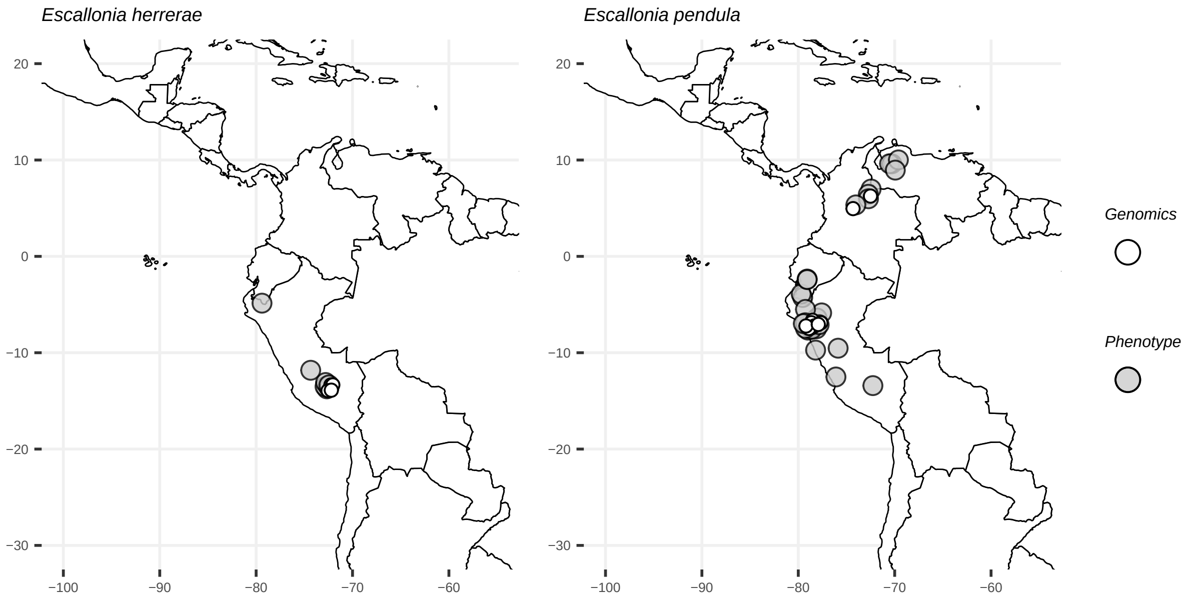

Figure S15: Geographic distribution of specimens sampled for this clade according to taxonomic species. Filled symbols indicate specimens used in phenotypic analyses and empty symbols specimens used in genomic analyses.

Return to Clade II Sampling

4.3.3 Fig S16: Current state of taxonomic species with phenotypic data

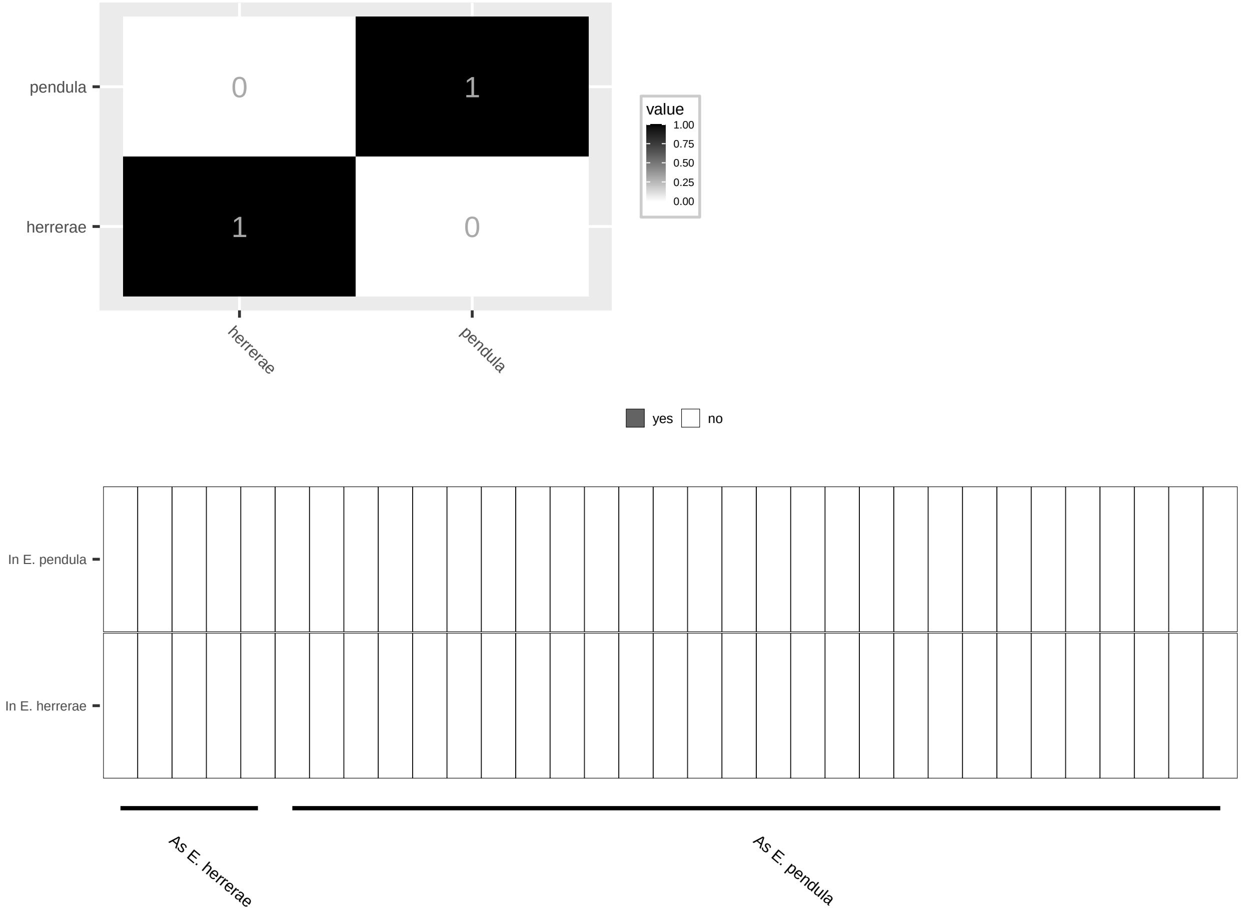

Figure S16: Assessment of current state of taxonomic species with phenotypic data. Top panel: Pairwise overlap among 10-cubes describing geometrically each taxonomic species. Bottom panel: Matching-prediction analysis with each cell along the x-axis representing specimens sorted according to taxonomic species and the 10-cubes corresponding to each taxonomic species along the y-axis. If a specimen matches the prediction of the monograph (i.e., it is inside a 10-cube), the corresponding cell is shaded. If the specimen does not match the prediction, the cell is empty.

Return to Clade II Current state of taxonomic species

4.3.4 Fig S17: Phenogroup delimitation: Gaussian finite mixture modeling

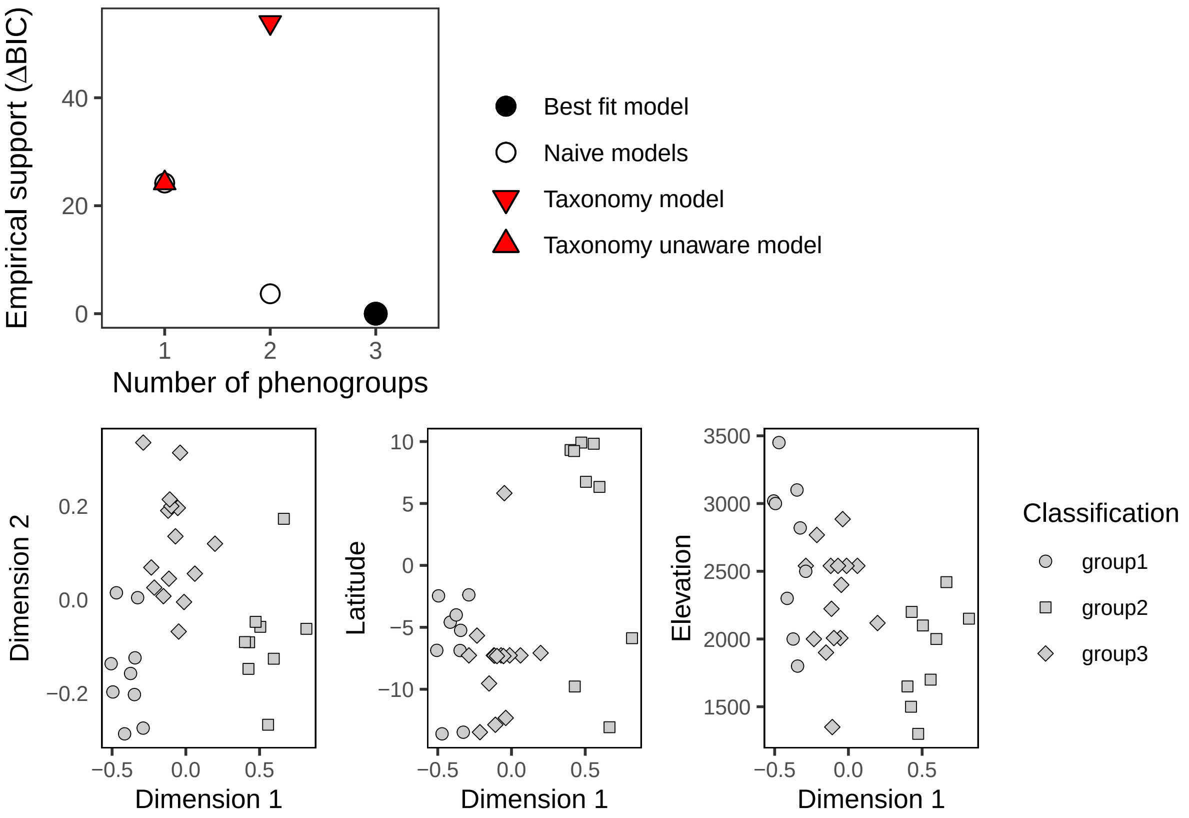

Figure S17: Gaussian finite mixture modeling (GFMM) for phenogroup delimitation and model selection using the Bayesian information criterion (BIC). Top panel: empirical support (ordinate) for Gaussian mixture models (GMM) assuming distinct number of phenogroups (abscissa). Each GMM specifies different number of phenogroups (shapes). Empirical support was measured as difference in BIC relative to the best model ($\Delta BIC = 0$). Bottom panel: Visualization of the phenogroups (shapes) identified by the best fit GMM; left panel shows phenogroups in the space defined by two axes obtained by linear discriminat analysis (to maximize separation and visualization), middle panel shows phenogroups in the space defined by discriminant axis 1 and latitude, and right panel shows phenogroups in the space defined by discriminant axis 1 and elevation.

Return to Clade II Phenomics: model-based species discovery

4.3.5 Fig S18: Sensitivity tests with 75% missing data

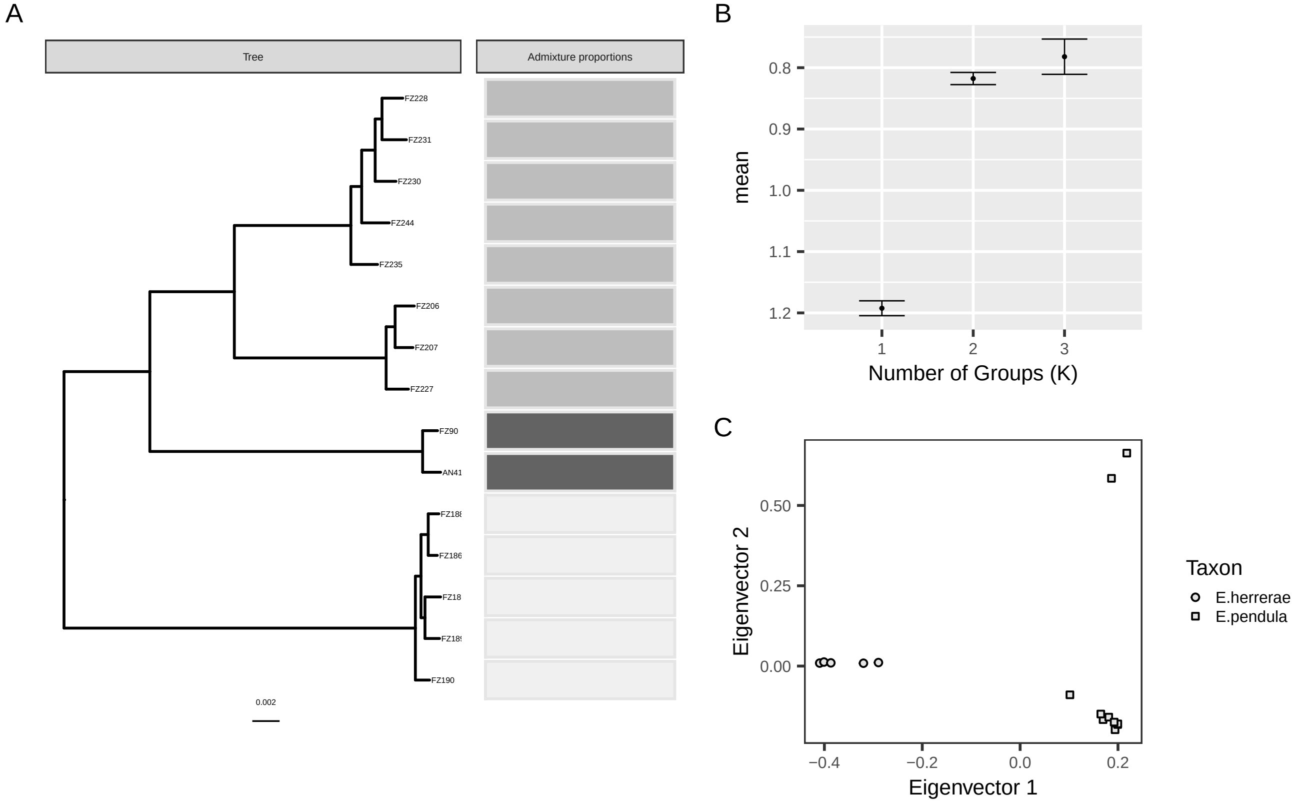

Figure S18: Impact of missing data (75%) on data analysis. A) maximum likelihood phylogenetic tree of specimens assigned to demes according to best `ADMIXTURE` run. B) Mean cross validation error with 95% confidence interval (ordinate) for ten replicate runs of `ADMIXTURE` assuming differnt number of demes (K) (abscissa); C) Scatterplot of Principal Component Analysis (PCA) projected along the first two axes.

Return to Clade II Genomics: sensitivity tests

4.3.6 Fig S19: Sensitivity tests with 50% missing data

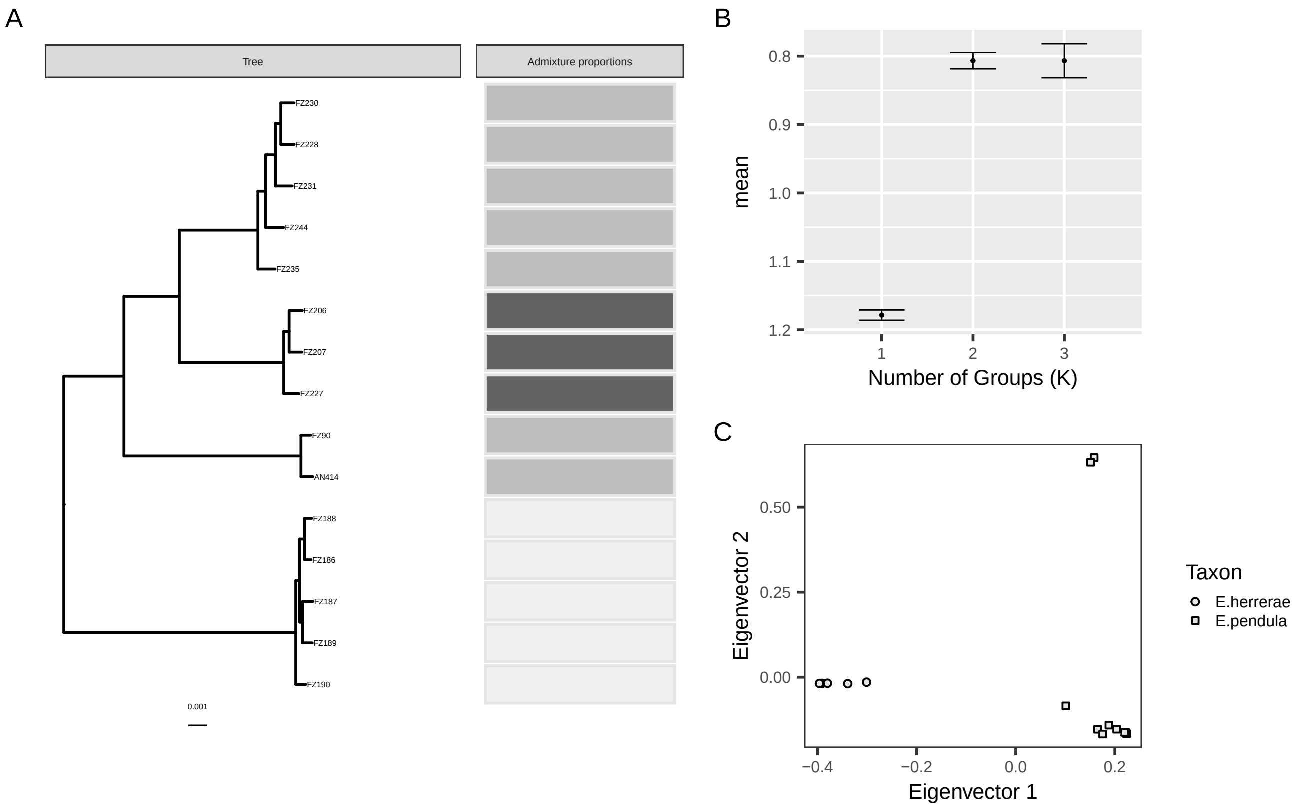

Figure S19: Impact of missing data (50%) on data analysis. A) maximum likelihood phylogenetic tree of specimens assigned to demes according to best `ADMIXTURE` run. B) Mean cross validation error with 95% confidence interval (ordinate) for ten replicate runs of `ADMIXTURE` assuming differnt number of demes (K) (abscissa); C) Scatterplot of Principal Component Analysis (PCA) projected along the first two axes.

Return to Clade II Genomics: sensitivity tests

4.3.7 Fig S20: Sensitivity tests with 25% missing data

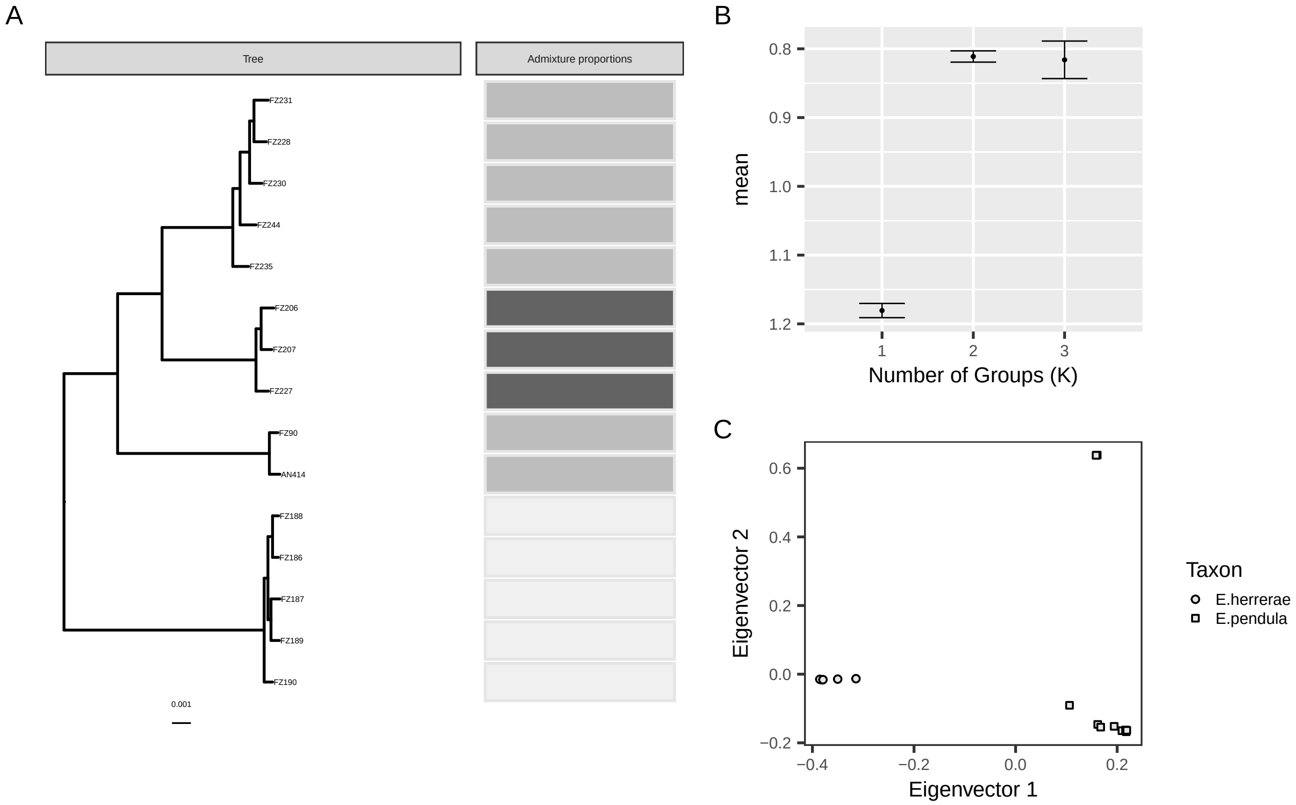

Figure S20: Impact of missing data (25%) on data analysis. A) maximum likelihood phylogenetic tree of specimens assigned to demes according to best `ADMIXTURE` run. B) Mean cross validation error with 95% confidence interval (ordinate) for ten replicate runs of `ADMIXTURE` assuming differnt number of demes (K) (abscissa); C) Scatterplot of Principal Component Analysis (PCA) projected along the first two axes.

Return to Clade II Genomics: sensitivity tests

4.3.8 Fig S21: Genogroup delimitation: Genotypic cluster model

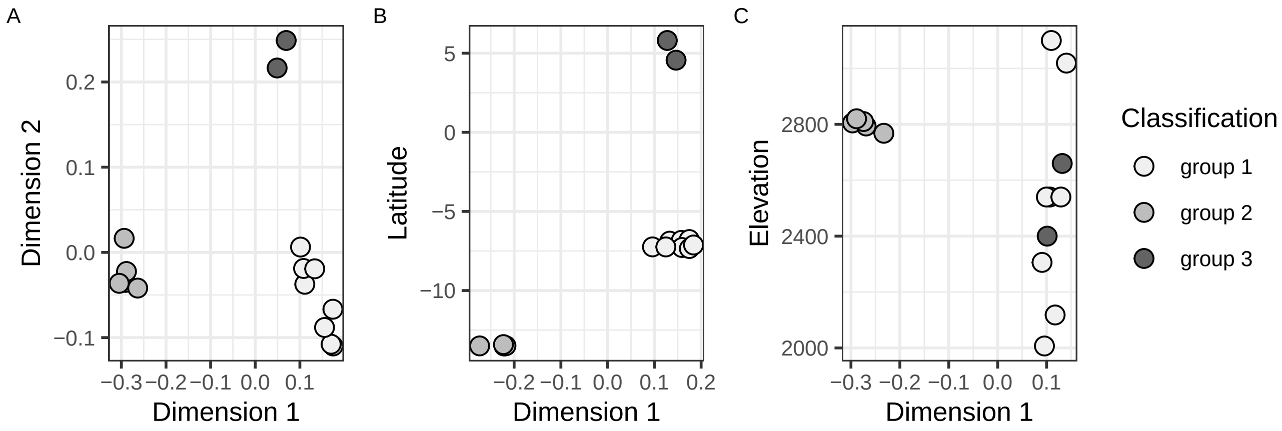

Figure S21: Gaussian finite mixture modeling (GFMM) for genogroup delimitation. Visualization of the genogroups (shades) identified by the best fit Gaussian mixture model (GMM). A) genogroups in the space defined by two axes obtained by non-metric multidimensional scaling (NMDS); B) genogroups in the space defined by NMDS axis 1 and latitude; C) genogroups in the space defined by NMDS axis 1 and elevation.

Return to Clade II Genomics: model-based species discovery

4.3.9 Fig S22: Genogroup delimitation. Cladogenesis to anagenesis model

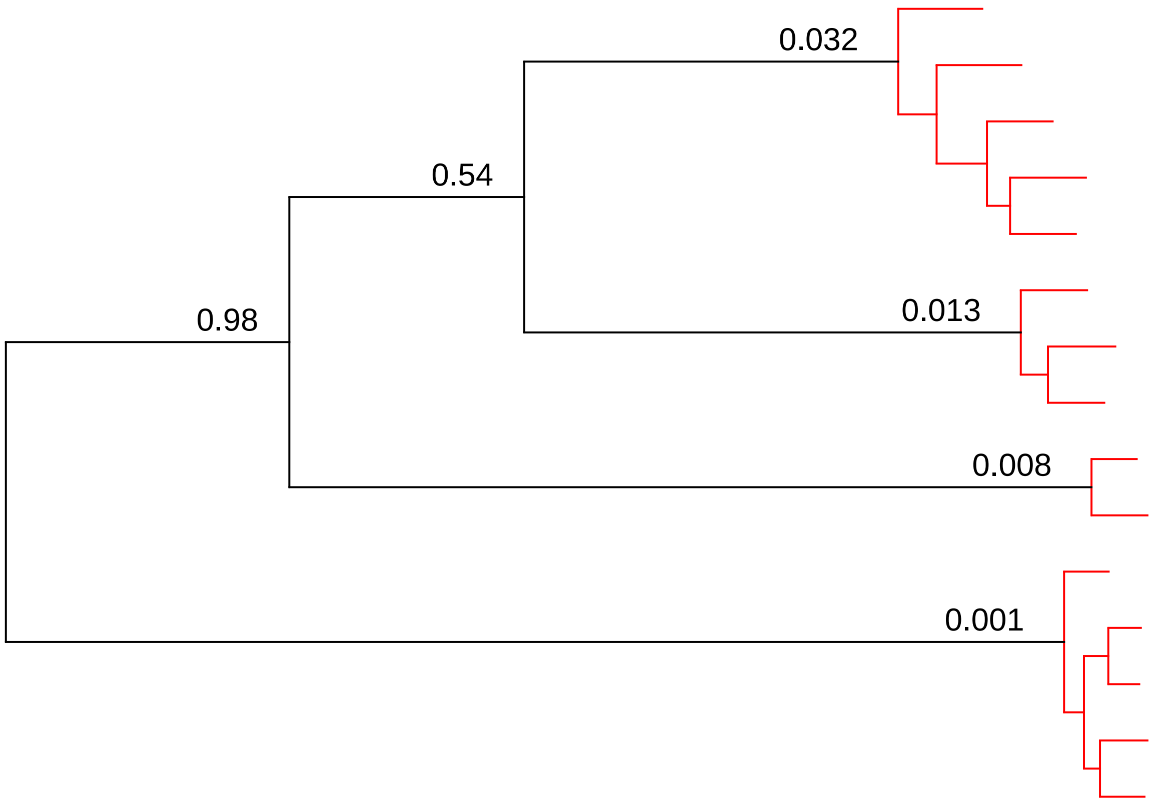

Figure S22: Phylogenetic modeling for genogroup delimitation. Midpoint-rooted phylogenetic tree showing genogroups in red. Values correspond to nodes at the transition point between cladogenesis (between species) to anagenesis (within species). Values closer to 0 indicate that the node was identified as a transition to anagenesis summarized over 500 delimitations.

Return to Clade II Genomics: model-based species discovery

4.3.10 Fig S23: Genogroup delimitation: Reproductive isolation model

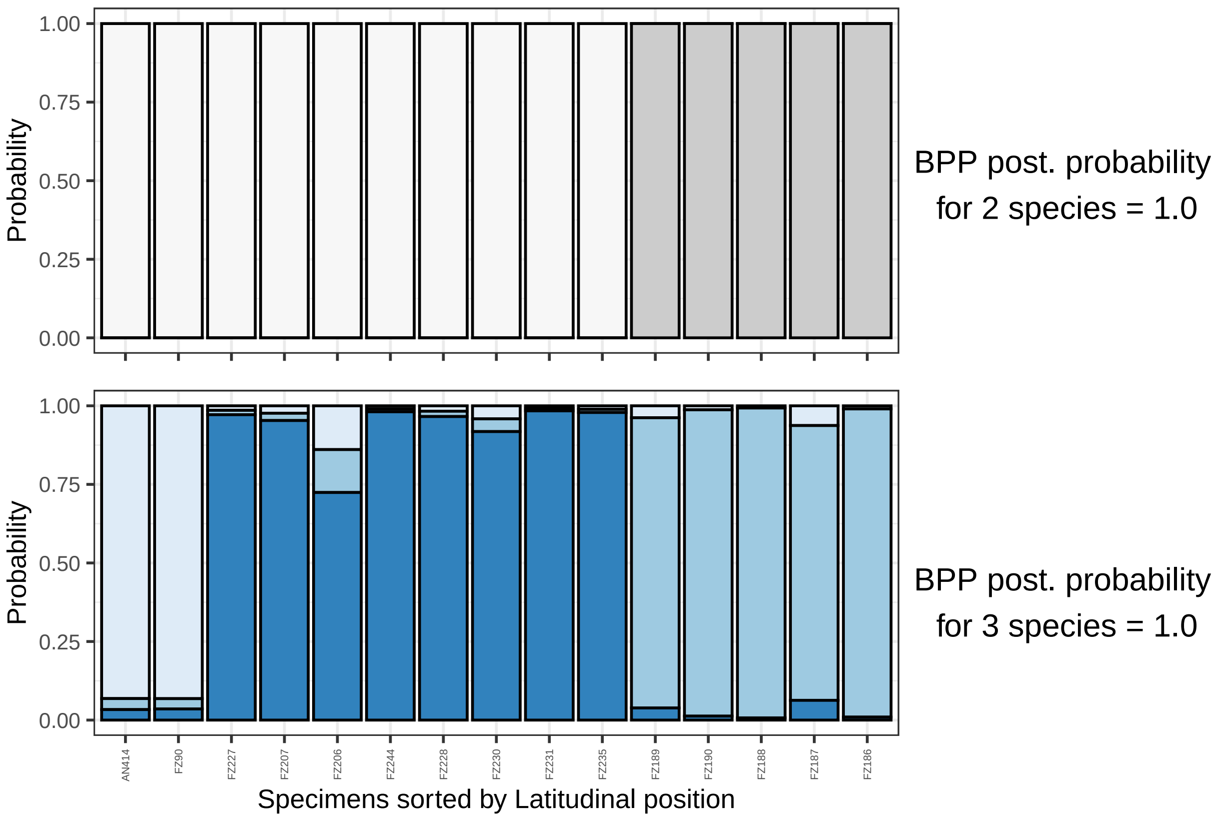

Figure S23: Population genetic modeling for genogroup delimitation. Top panel: assignment of specimens to demes according to `STRUCTURE` and posterior probability of species delimitation modeling according to `BPP` using these demes. Bottom panel: assignment of specimens to demes according to `MAVERICK` and posterior probability of species delimitation modeling according to `BPP` using these demes. Specimens are sorted from north (left) to south (right) according to locality of collection.

Return to Clade II Genomics: model-based species discovery

4.3.11 Fig S24: Data integration

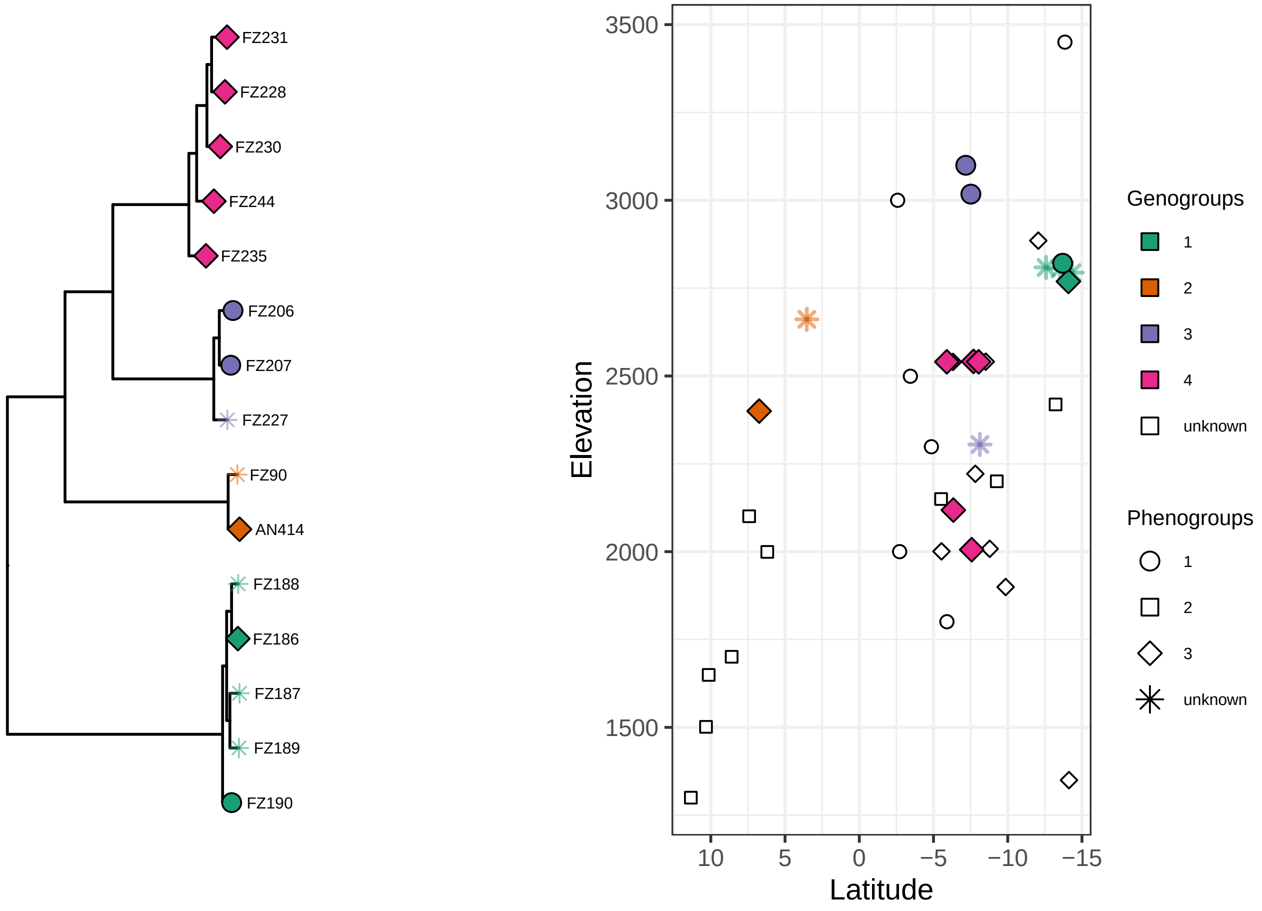

Figure S24: Integration of phenotypic and genomic data with spatial information and evolutionary history. All specimens are assigned to their corresponding best fit phenogroup (shapes) and genogroup (colors). Specimens without phenotypic or genomic data (unknown specimens) are shown as asterisks and empty shapes, accordingly. Specimens are shown as tips of the maximum likelihood tree (left) used in the CA model analysis and mapped along latitude and elevation (right). Specimens assigned to a single phenogroup and a single genogroup delineate species that we determined as 'good species'. Specimens assigned to a single phenogroup across multiple genogroups delineate species that we determined as 'phenotypic cryptic species'. Specimens assigned to a single genogroup across multiple phenogroups delineate species that we determined as 'genetic cryptic species'.

Return to Clade II Data integration

4.4 Clade III

4.4.1 Fig S25: Taxon sampling

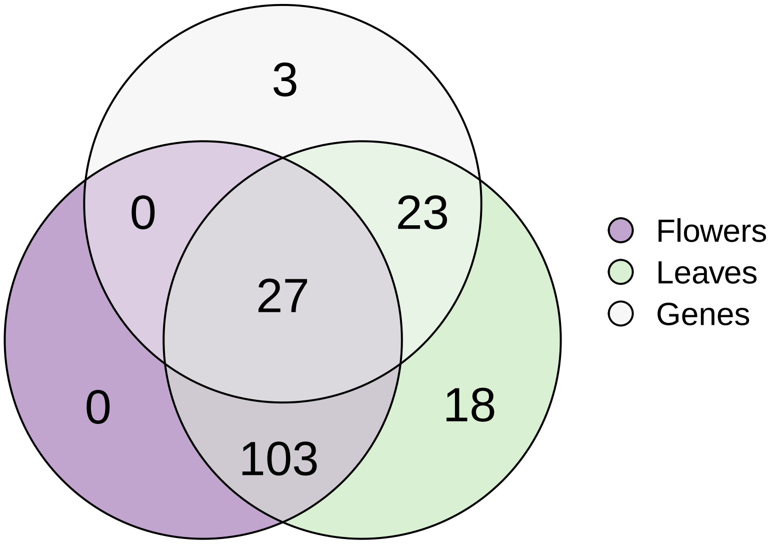

Figure S25: Specimens sampled according to three types of data. Specimens outside the Flowers category represent sterile specimens.

Return to Clade III Sampling

4.4.2 Fig S26: Geographic distribution

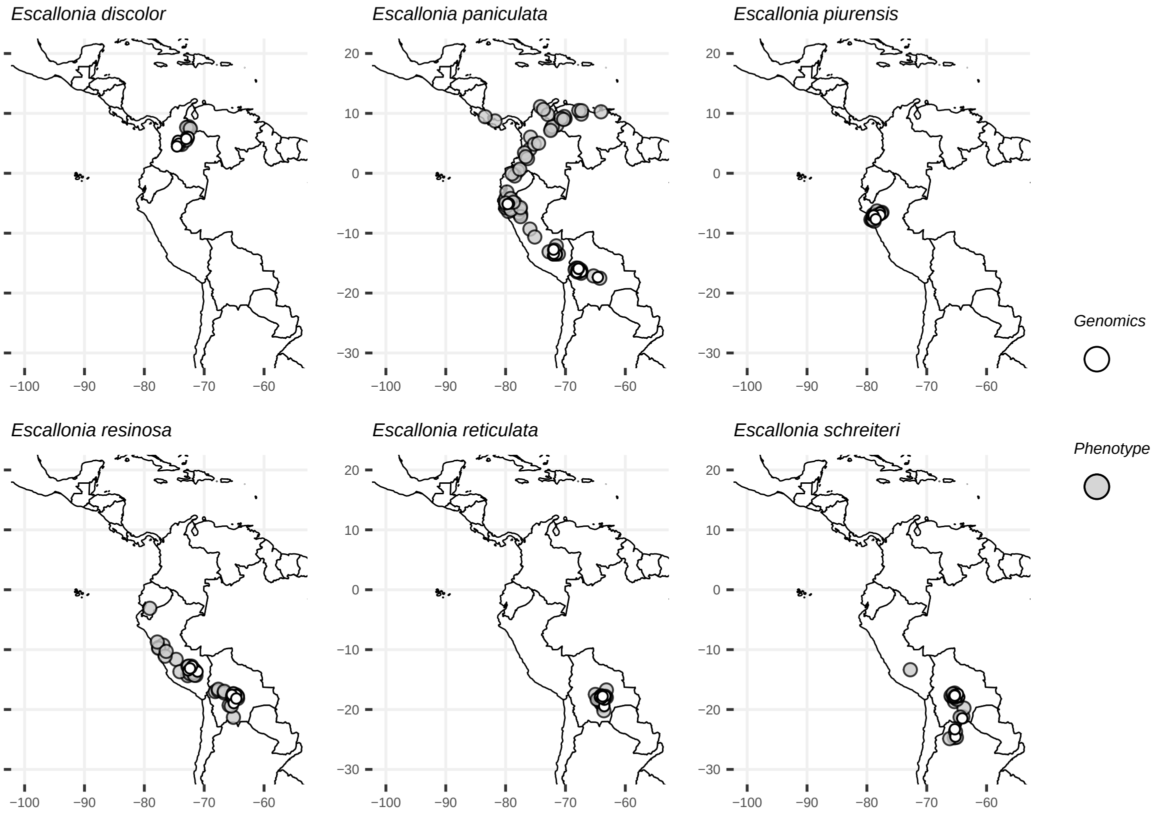

Figure S26: Geographic distribution of specimens sampled for this clade according to taxonomic species. Filled symbols indicate specimens used in phenotypic analyses and empty symbols specimens used in genomic analyses.

Return to Clade III Sampling

4.4.3 Fig S27: Current state of taxonomic species with phenotypic data

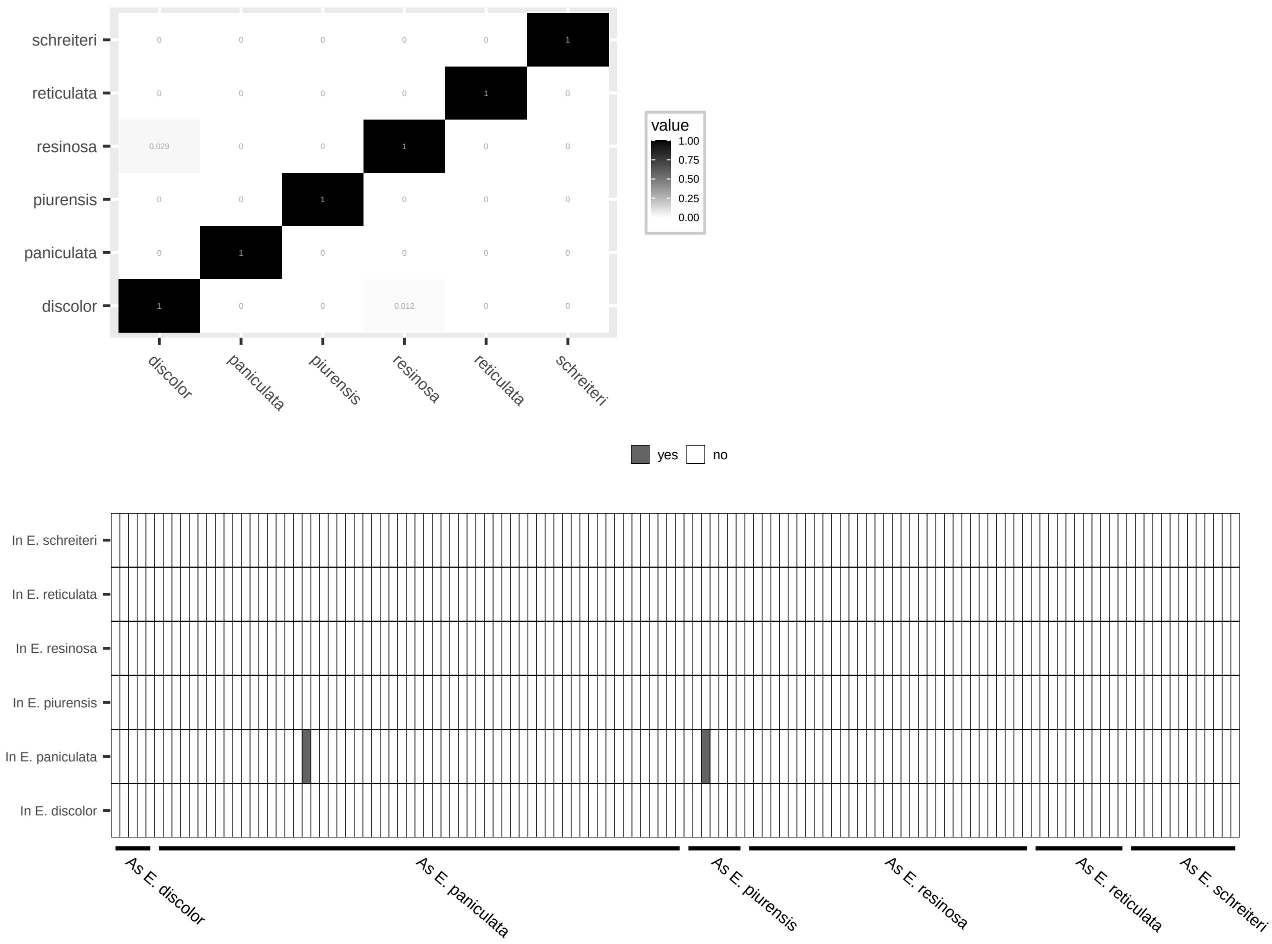

Figure S27: Assessment of current state of taxonomic species with phenotypic data. Top panel: Pairwise overlap among 10-cubes describing geometrically each taxonomic species. Bottom panel: Matching-prediction analysis with each cell along the x-axis representing specimens sorted according to taxonomic species and the 10-cubes corresponding to each taxonomic species along the y-axis. If a specimen matches the prediction of the monograph (i.e., it is inside a 10-cube), the corresponding cell is shaded. If the specimen does not match the prediction, the cell is empty.

Return to Clade III Current state of taxonomic species

4.4.4 Fig S28: Phenogroup delimitation: Gaussian finite mixture modeling

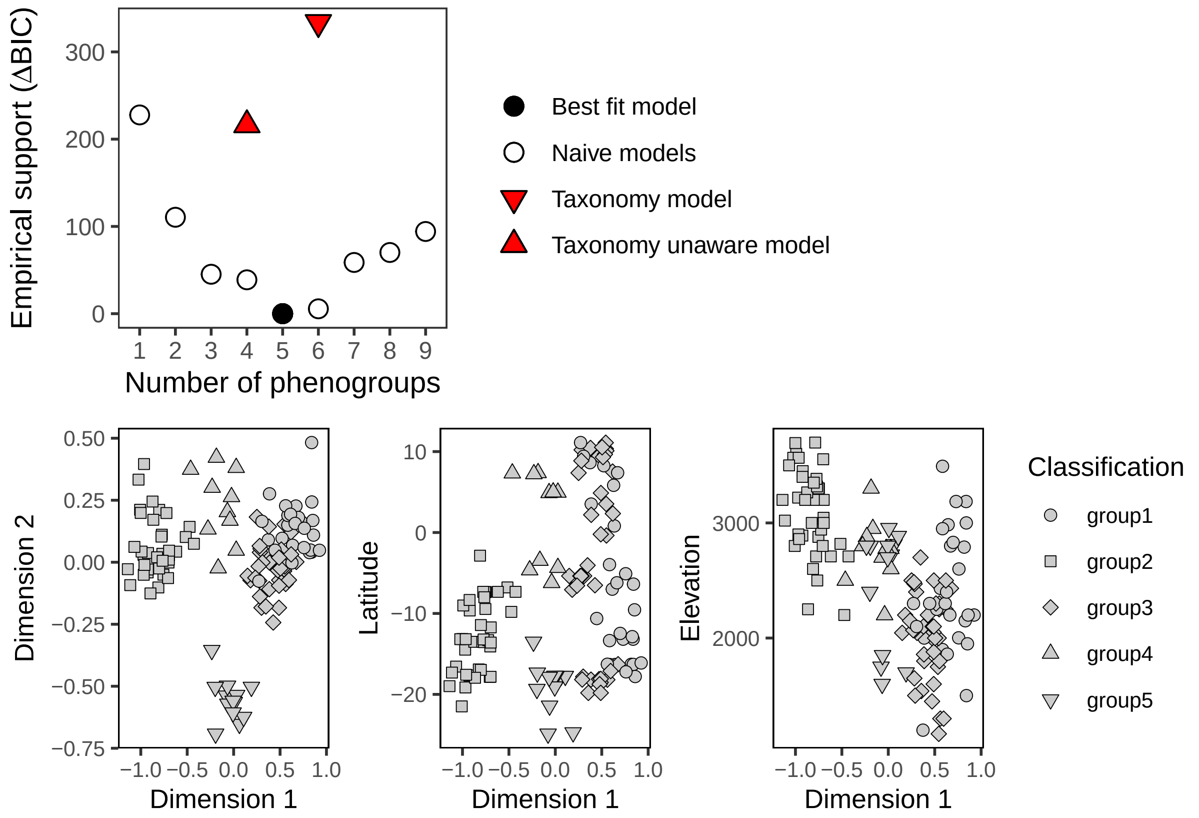

Figure S28: Gaussian finite mixture modeling (GFMM) for phenogroup delimitation and model selection using the Bayesian information criterion (BIC). Top panel: empirical support (ordinate) for Gaussian mixture models (GMM) assuming distinct number of phenogroups (abscissa). Each GMM specifies different number of phenogroups (shapes). Empirical support was measured as difference in BIC relative to the best model ($\Delta\text{BIC} = 0$). Bottom panel: Visualization of the phenogroups (shapes) identified by the best fit GMM; left panel shows phenogroups in the space defined by two axes obtained by linear discriminat analysis (to maximize separation and visualization), middle panel shows phenogroups in the space defined by discriminant axis 1 and latitude, and right panel shows phenogroups in the space defined by discriminant axis 1 and elevation.

Return to Clade III Phenomics: model-based species discovery

4.4.5 Fig S29: Sensitivity tests with 75% missing data

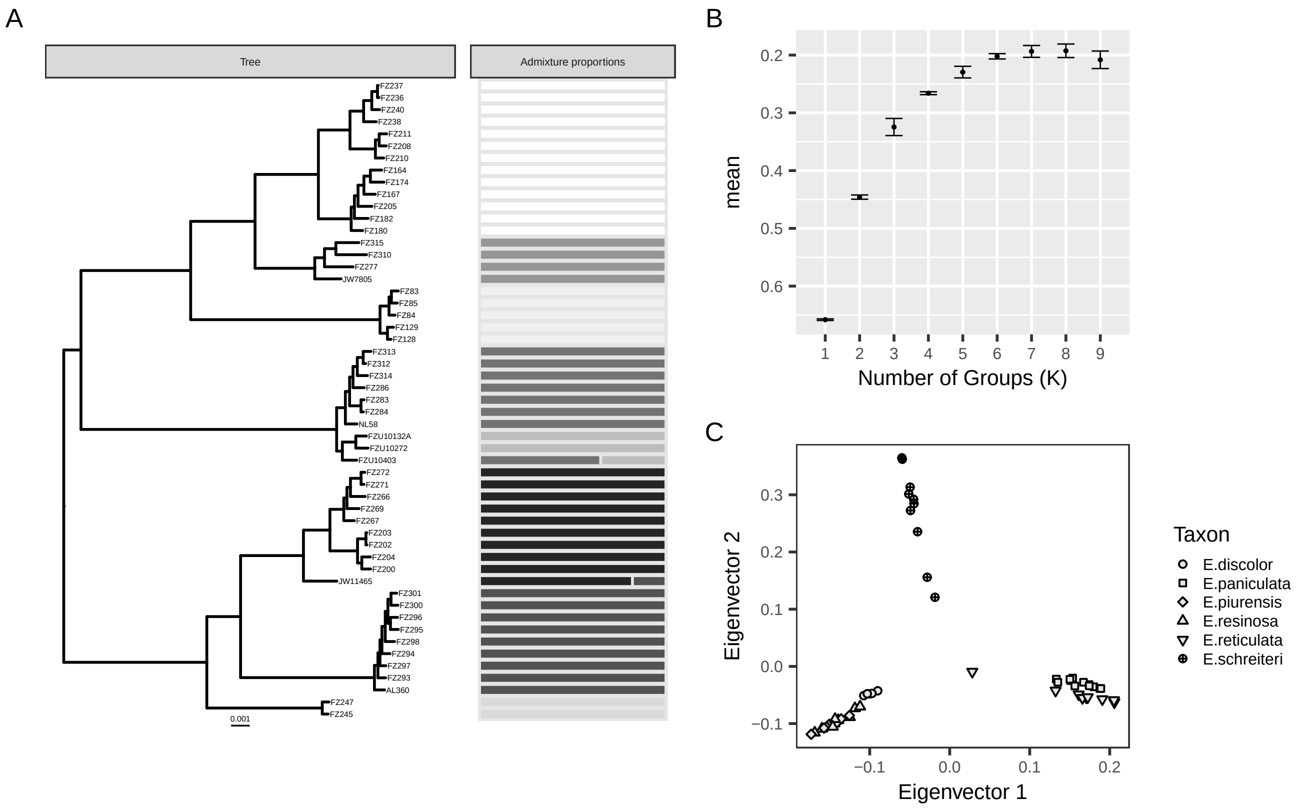

Figure S29: Impact of missing data (75%) on data analysis. A) maximum likelihood phylogenetic tree of specimens assigned to demes according to best `ADMIXTURE` run. B) Mean cross validation error with 95% confidence interval (ordinate) for ten replicate runs of `ADMIXTURE` assuming differnt number of demes (K) (abscissa); C) Scatterplot of Principal Component Analysis (PCA) projected along the first two axes.

Return to Clade III Genomics: sensitivity tests

4.4.6 Fig S30: Sensitivity tests with 50% missing data

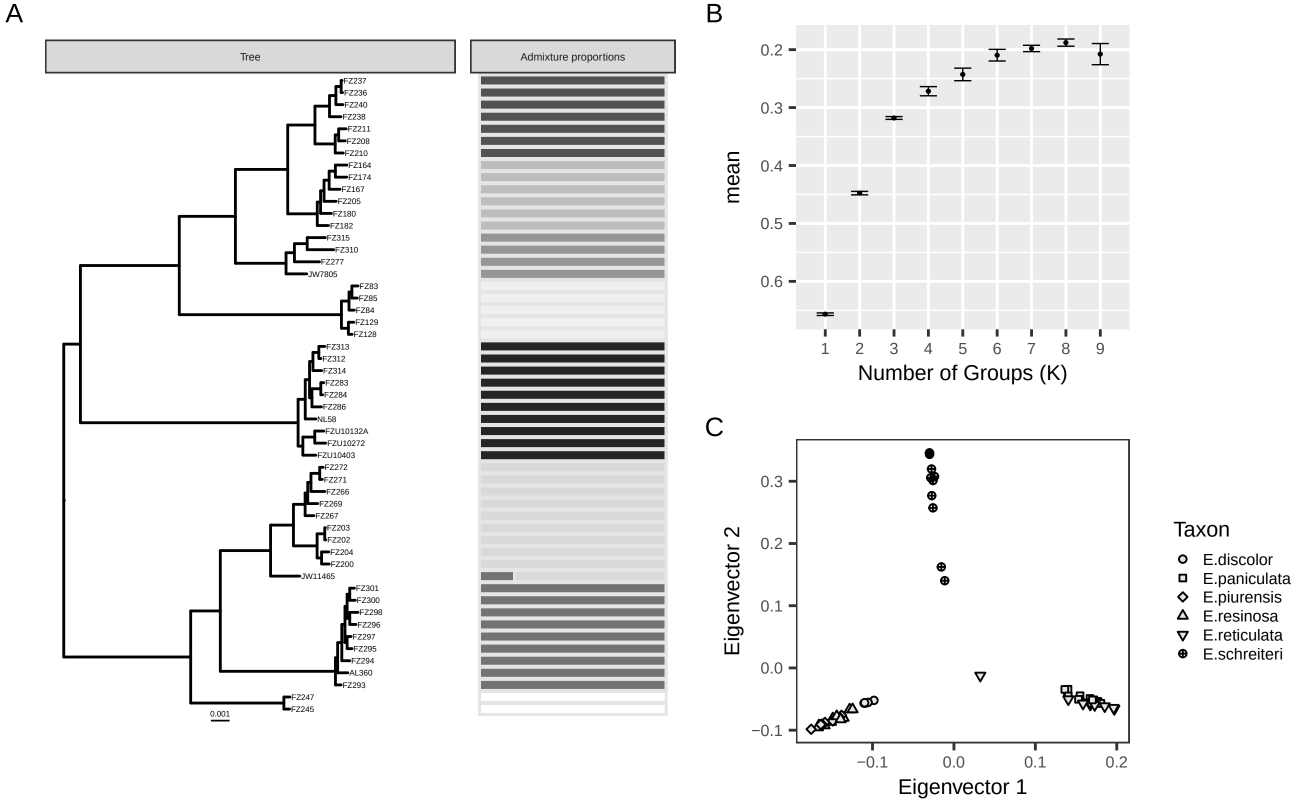

Figure S30: Impact of missing data (50%) on data analysis. A) maximum likelihood phylogenetic tree of specimens assigned to demes according to best `ADMIXTURE` run. B) Mean cross validation error with 95% confidence interval (ordinate) for ten replicate runs of `ADMIXTURE` assuming differnt number of demes (K) (abscissa); C) Scatterplot of Principal Component Analysis (PCA) projected along the first two axes.

Return to Clade III Genomics: sensitivity tests

4.4.7 Fig S31: Sensitivity tests with 25% missing data

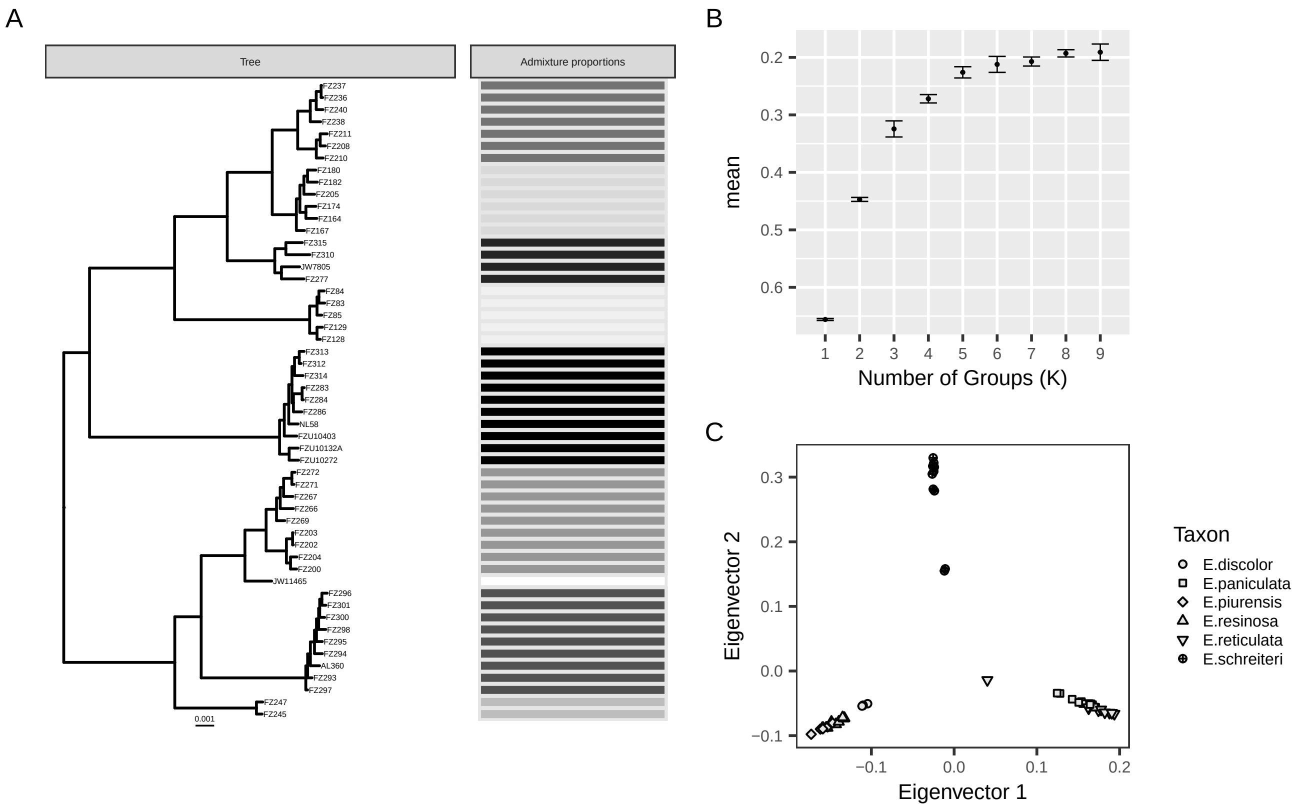

Figure S31: Impact of missing data (25%) on data analysis. A) maximum likelihood phylogenetic tree of specimens assigned to demes according to best `ADMIXTURE` run. B) Mean cross validation error with 95% confidence interval (ordinate) for ten replicate runs of `ADMIXTURE` assuming differnt number of demes (K) (abscissa); C) Scatterplot of Principal Component Analysis (PCA) projected along the first two axes.

Return to Clade III Genomics: sensitivity tests

4.4.8 Fig S32: Genogroup delimitation: Genotypic cluster model

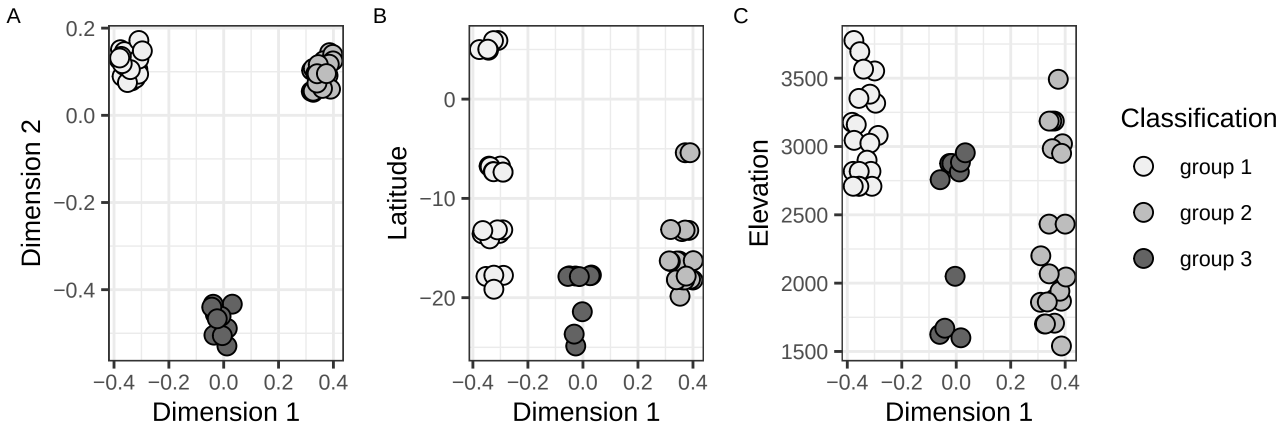

Figure S32: Gaussian finite mixture modeling (GFMM) for genogroup delimitation. Visualization of the genogroups (shades) identified by the best fit Gaussian mixture model (GMM). A) genogroups in the space defined by two axes obtained by non-metric multidimensional scaling (NMDS); B) genogroups in the space defined by NMDS axis 1 and latitude; C) genogroups in the space defined by NMDS axis 1 and elevation.

Return to Clade III Genomics: model-based species discovery

4.4.9 Fig S33: Genogroup delimitation. Cladogenesis to anagenesis model

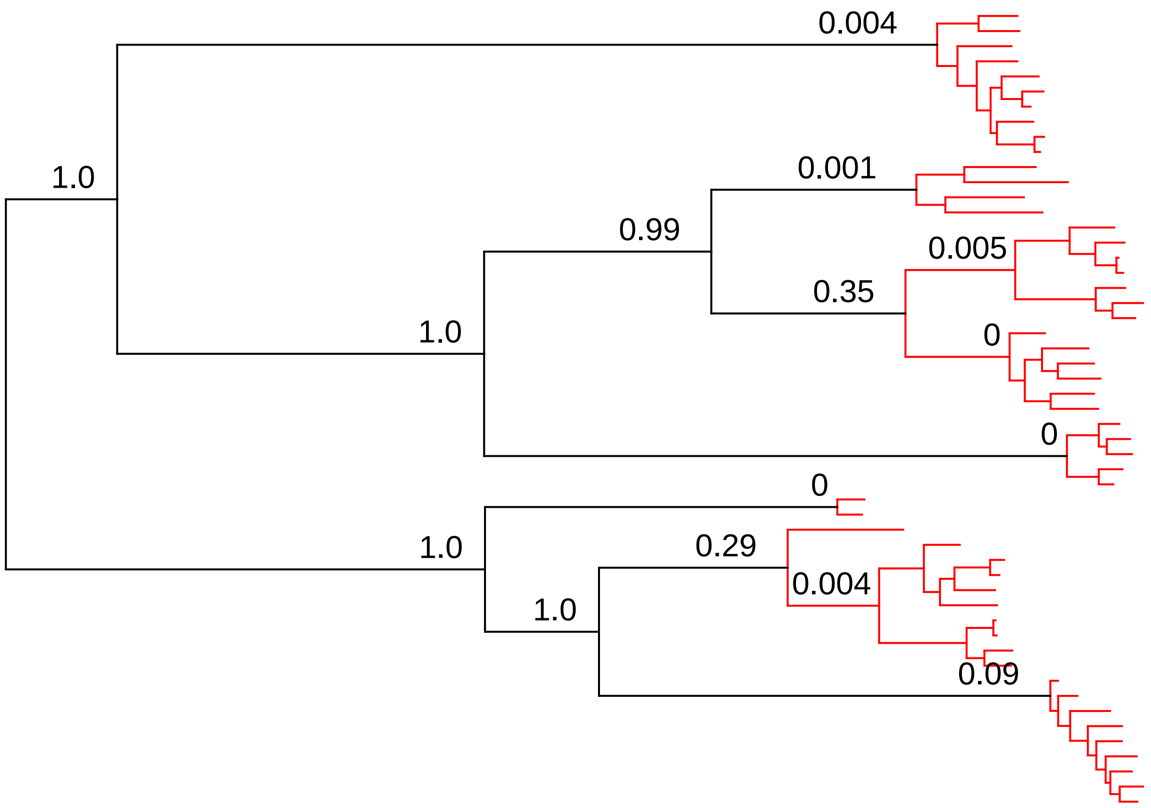

Figure S33: Phylogenetic modeling for genogroup delimitation. Midpoint-rooted phylogenetic tree showing genogroups in red. Values correspond to nodes at the transition point between cladogenesis (between species) to anagenesis (within species). Values closer to 0 indicate that the node was identified as a transition to anagenesis summarized over 500 delimitations.

Return to Clade III Genomics: model-based species discovery

4.4.10 Fig S34: Genogroup delimitation: Reproductive isolation model

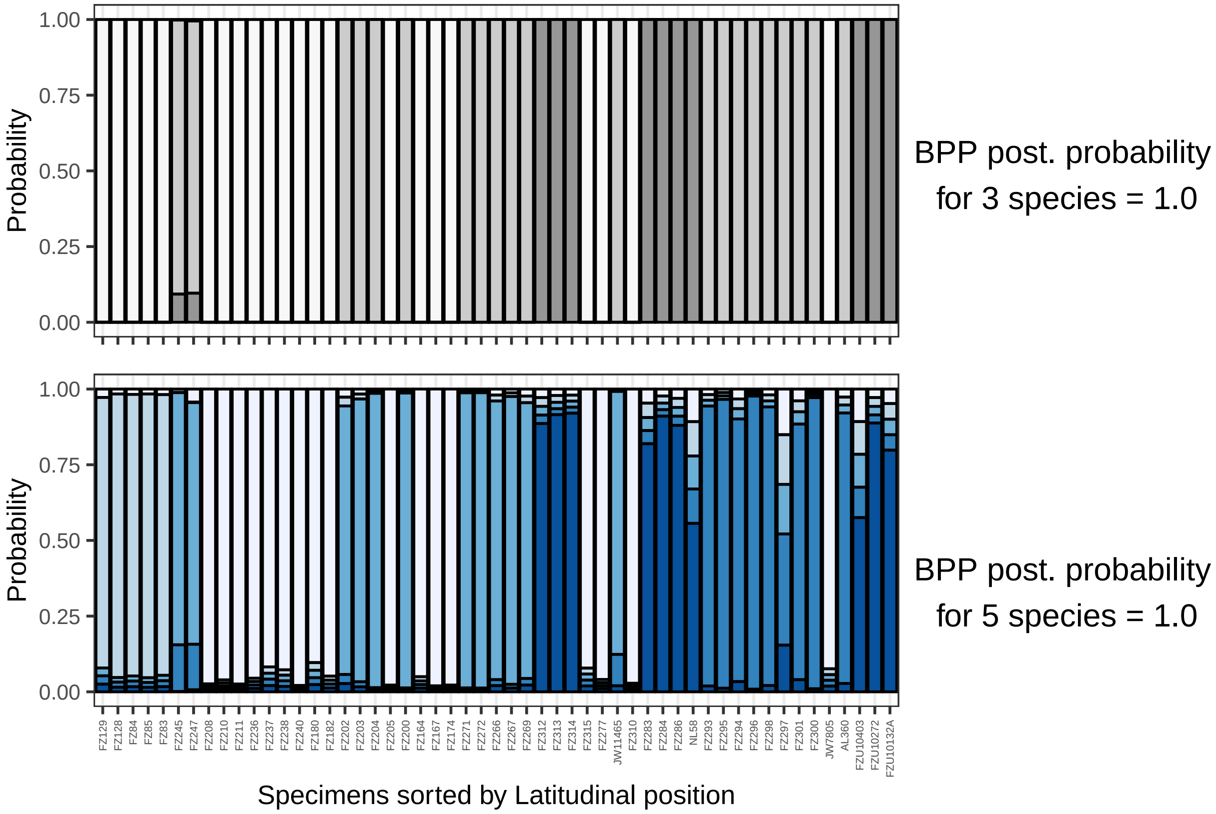

Figure S34: Population genetic modeling for genogroup delimitation. Top panel: assignment of specimens to demes according to `STRUCTURE` and posterior probability of species delimitation modeling according to `BPP` using these demes. Bottom panel: assignment of specimens to demes according to `MAVERICK` and posterior probability of species delimitation modeling according to `BPP` using these demes. Specimens are sorted from north (left) to south (right) according to locality of collection.

Return to Clade III Genomics: model-based species discovery

4.4.11 Fig S35: Data integration

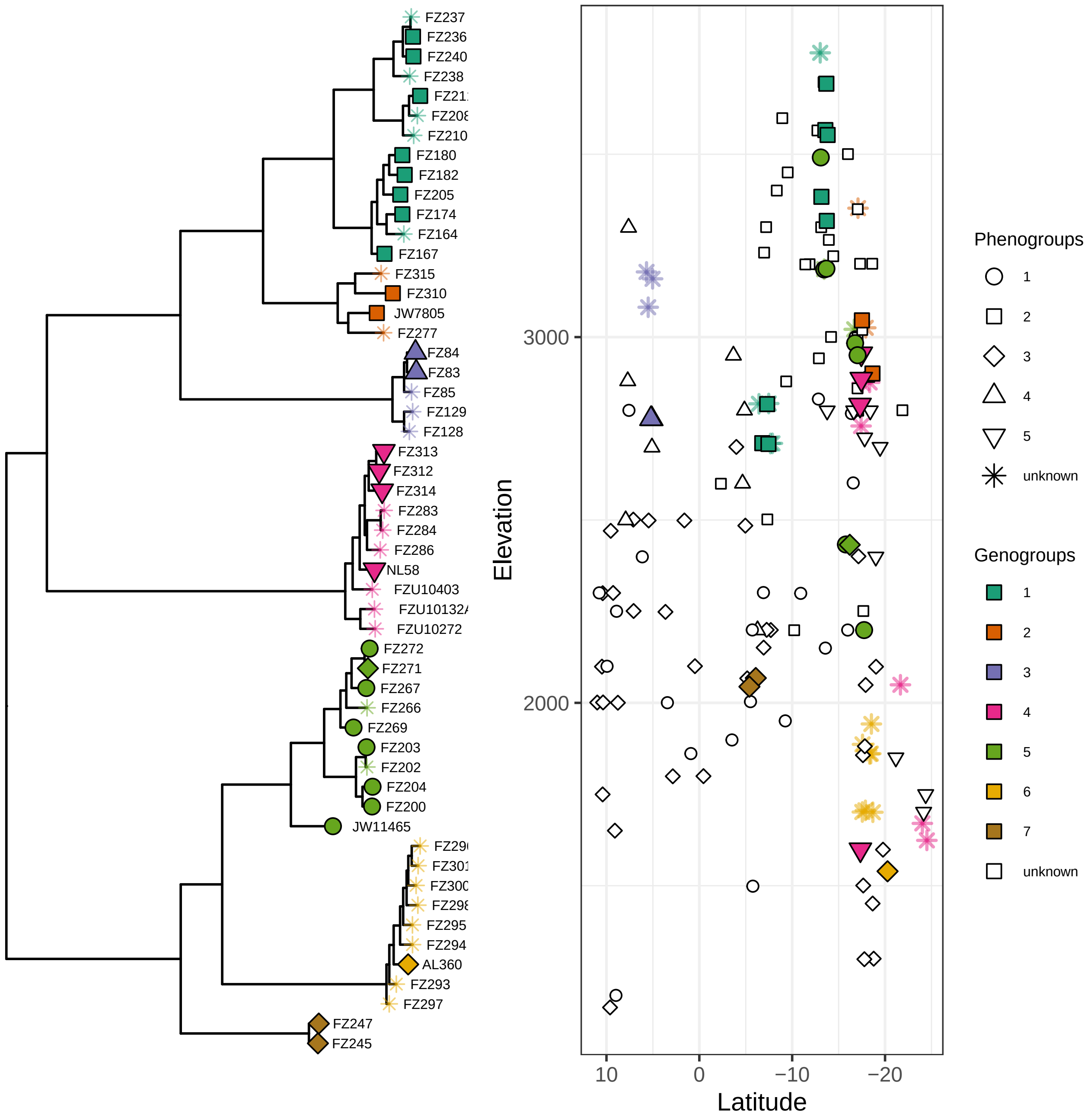

Figure S35: Integration of phenotypic and genomic data with spatial information and evolutionary history. All specimens are assigned to their corresponding best fit phenogroup (shapes) and genogroup (colors). Specimens without phenotypic or genomic data (unknown specimens) are shown as asterisks and empty shapes, accordingly. Specimens are shown as tips of the maximum likelihood tree (left) used in the CA model analysis and mapped along latitude and elevation (right). Specimens assigned to a single phenogroup and a single genogroup delineate species that we determined as 'good species'. Specimens assigned to a single phenogroup across multiple genogroups delineate species that we determined as 'phenotypic cryptic species'. Specimens assigned to a single genogroup across multiple phenogroups delineate species that we determined as 'genetic cryptic species'.

Return to Clade III Data integration

4.5 Clade IV

4.5.1 Fig S36: Taxon sampling

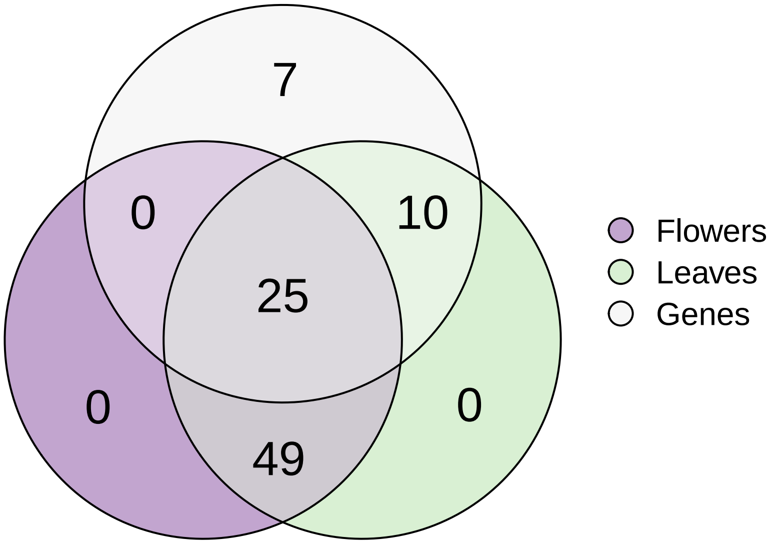

Figure S36: Specimens sampled according to three types of data. Specimens outside the Flowers category represent sterile specimens.

Return to Clade IV Sampling

4.5.2 Fig S37: Geographic distribution

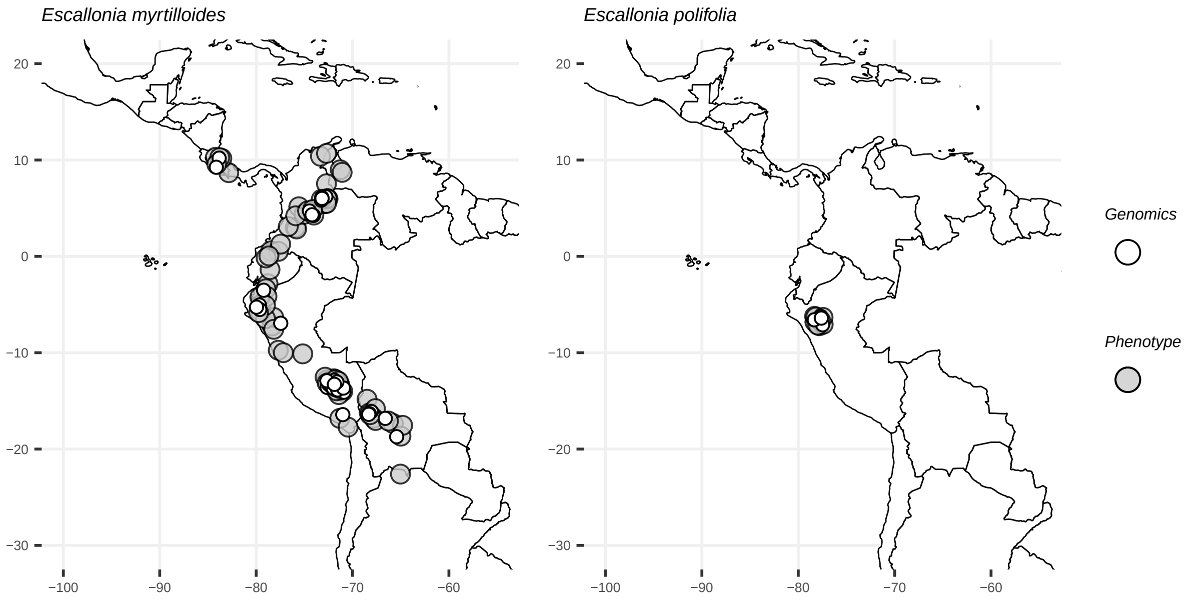

Figure S37: Geographic distribution of specimens sampled for this clade according to taxonomic species. Filled symbols indicate specimens used in phenotypic analyses and empty symbols specimens used in genomic analyses.

Return to Clade IV Sampling

4.5.3 Fig S38: Current state of taxonomic species with phenotypic data

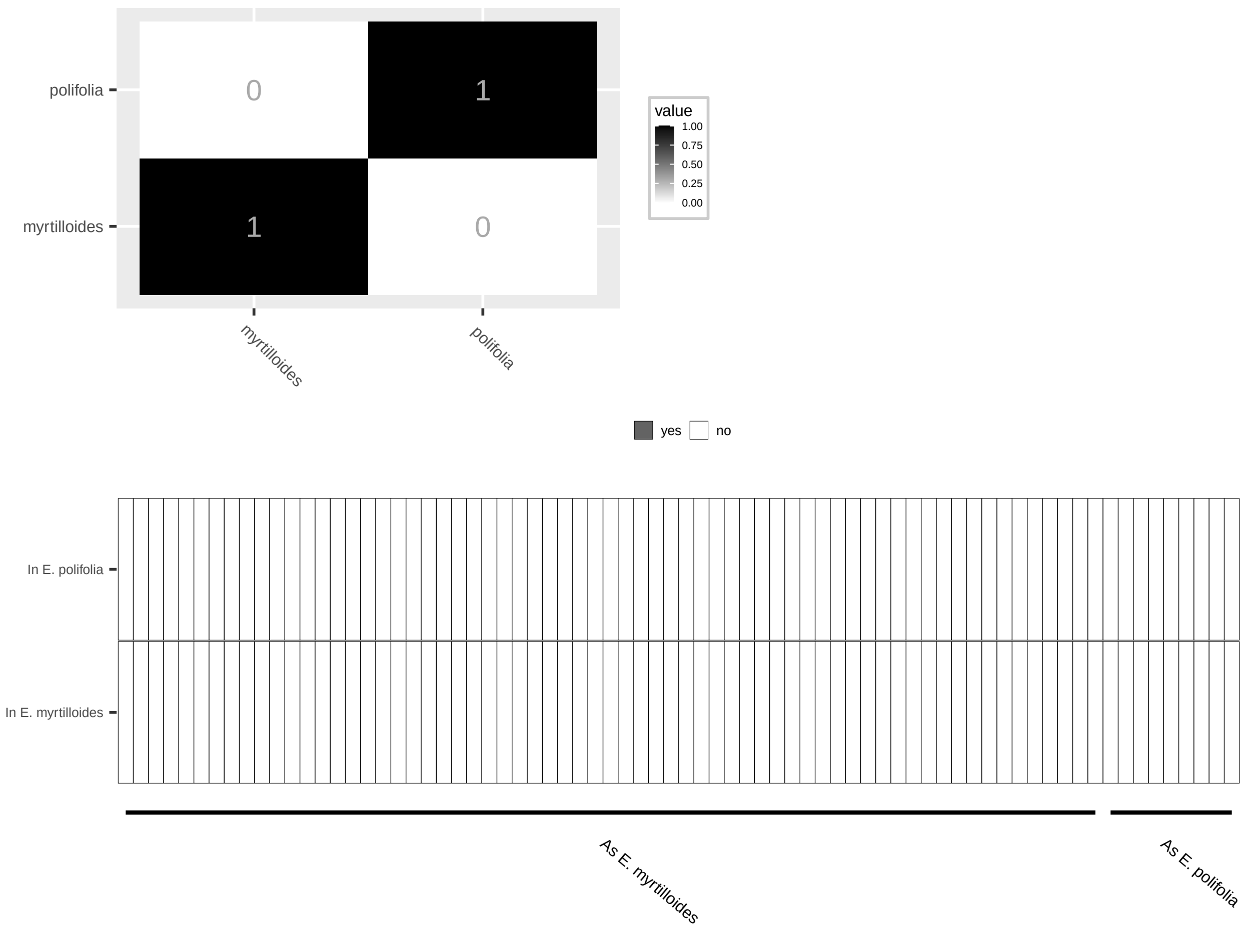

Figure S38: Assessment of current state of taxonomic species with phenotypic data. Top panel: Pairwise overlap among 10-cubes describing geometrically each taxonomic species. Bottom panel: Matching-prediction analysis with each cell along the x-axis representing specimens sorted according to taxonomic species and the 10-cubes corresponding to each taxonomic species along the y-axis. If a specimen matches the prediction of the monograph (i.e., it is inside a 10-cube), the corresponding cell is shaded. If the specimen does not match the prediction, the cell is empty.

Return to Clade IV Current state of taxonomic species

4.5.4 Fig S39: Phenogroup delimitation: Gaussian finite mixture modeling

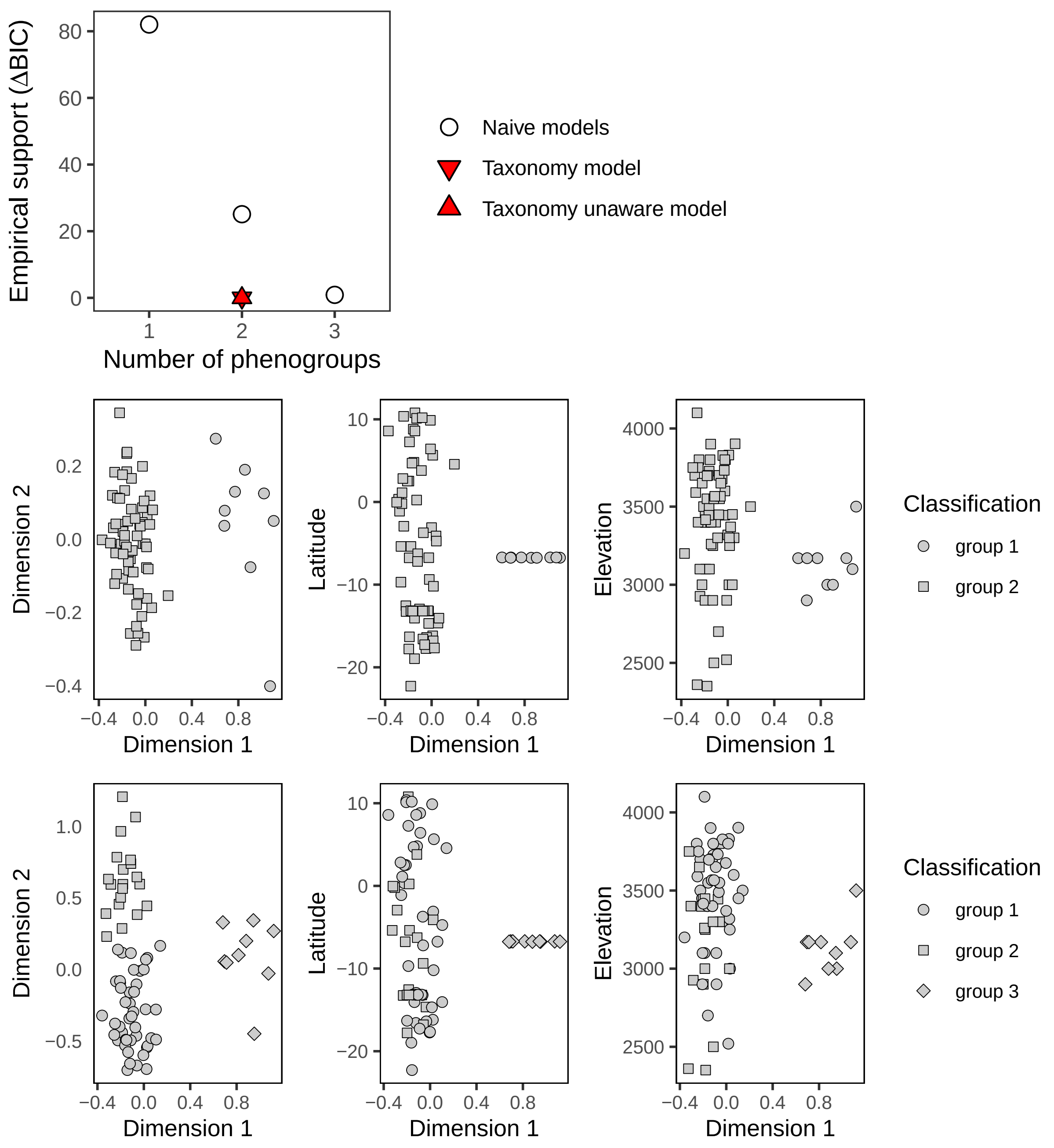

Figure S39: Gaussian finite mixture modeling (GFMM) for phenogroup delimitation and model selection using the Bayesian information criterion (BIC). Top panel: empirical support (ordinate) for Gaussian mixture models (GMM) assuming distinct number of phenogroups (abscissa). Each GMM specifies different number of phenogroups (shapes). Empirical support was measured as difference in BIC relative to the best model ($\Delta BIC = 0$). Bottom panel: Visualization of the phenogroups (shapes) identified by the best fit GMM; left panel shows phenogroups in the space defined by two axes obtained by linear discriminat analysis (to maximize separation and visualization), middle panel shows phenogroups in the space defined by discriminant axis 1 and latitude, and right panel shows phenogroups in the space defined by discriminant axis 1 and elevation.

4.5.5 Fig S40: Sensitivity tests with 75% missing data

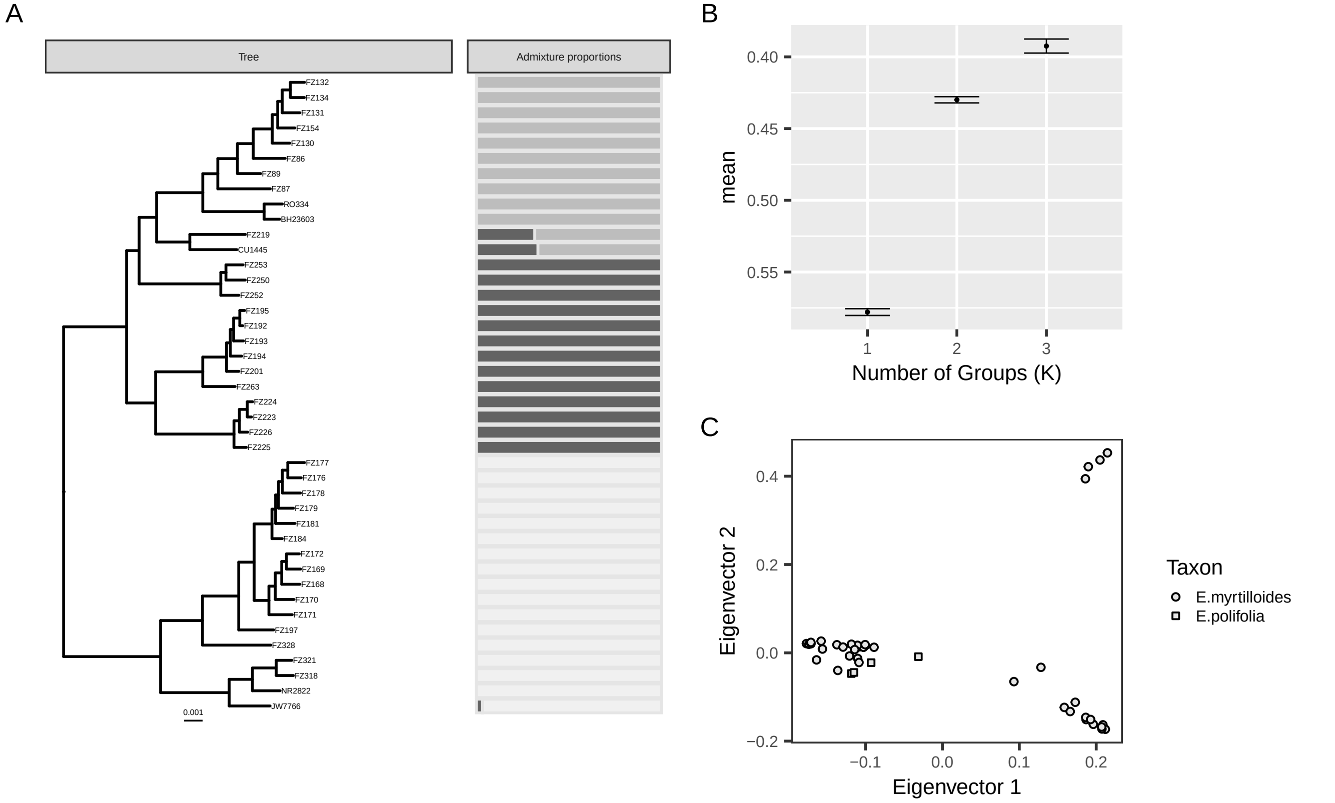

Figure S40: Impact of missing data (75%) on data analysis. A) maximum likelihood phylogenetic tree of specimens assigned to demes according to best `ADMIXTURE` run. B) Mean cross validation error with 95% confidence interval (ordinate) for ten replicate runs of `ADMIXTURE` assuming differnt number of demes (K) (abscissa); C) Scatterplot of Principal Component Analysis (PCA) projected along the first two axes.

Return to Clade IV Genomics: sensitivity tests

4.5.6 Fig S41: Sensitivity tests with 50% missing data

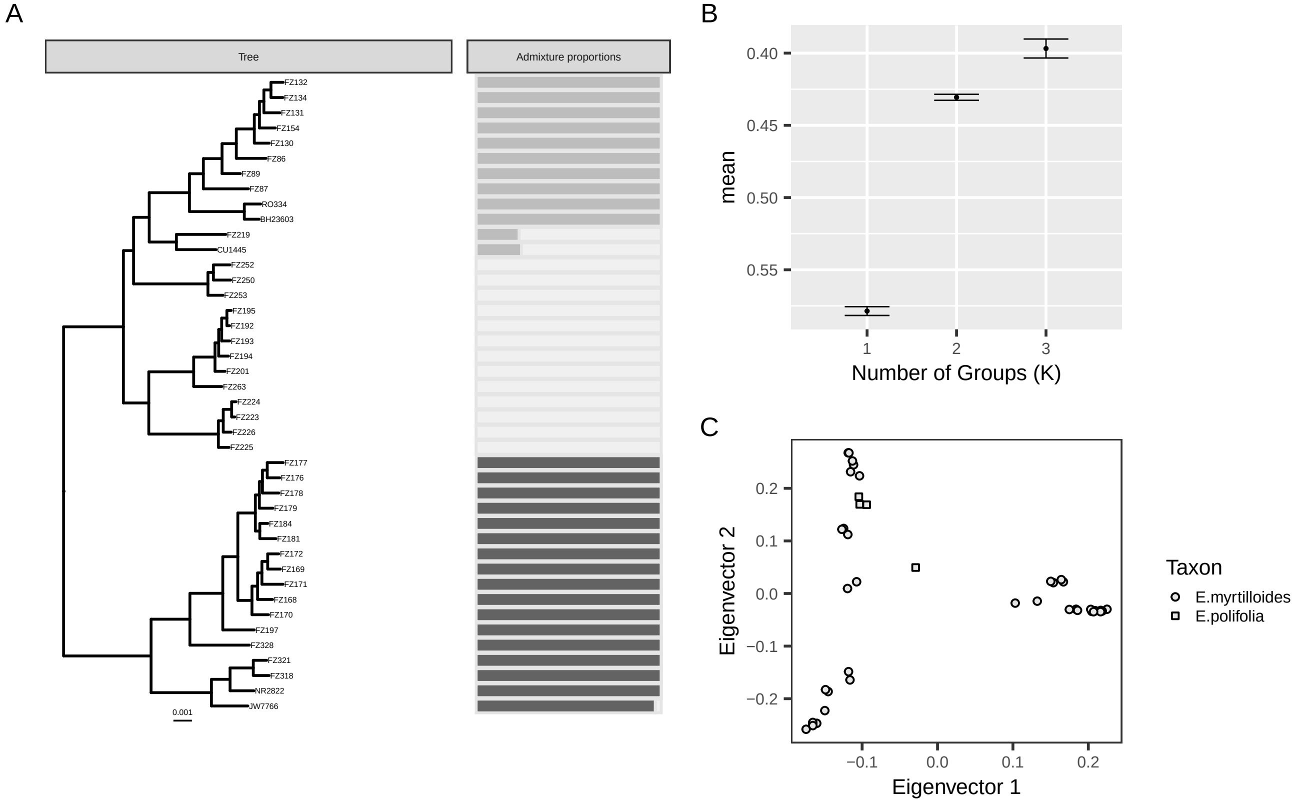

Figure S41: Impact of missing data (50%) on data analysis. A) maximum likelihood phylogenetic tree of specimens assigned to demes according to best `ADMIXTURE` run. B) Mean cross validation error with 95% confidence interval (ordinate) for ten replicate runs of `ADMIXTURE` assuming differnt number of demes (K) (abscissa); C) Scatterplot of Principal Component Analysis (PCA) projected along the first two axes.

Return to Clade IV Genomics: sensitivity tests

4.5.7 Fig S42: Sensitivity tests with 25% missing data

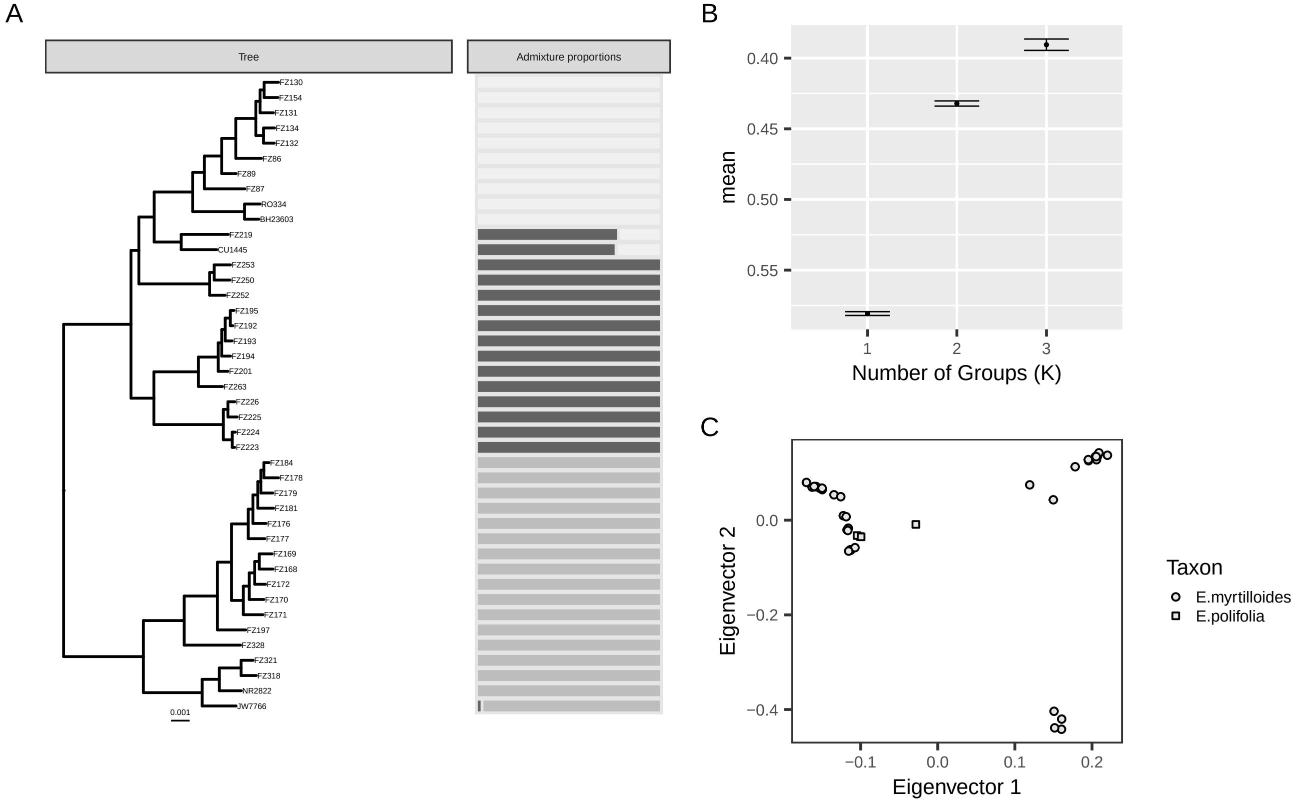

Figure S42: Impact of missing data (25%) on data analysis. A) maximum likelihood phylogenetic tree of specimens assigned to demes according to best `ADMIXTURE` run. B) Mean cross validation error with 95% confidence interval (ordinate) for ten replicate runs of `ADMIXTURE` assuming differnt number of demes (K) (abscissa); C) Scatterplot of Principal Component Analysis (PCA) projected along the first two axes.

Return to Clade IV Genomics: sensitivity tests

4.5.8 Fig S43: Genogroup delimitation: Genotypic cluster model

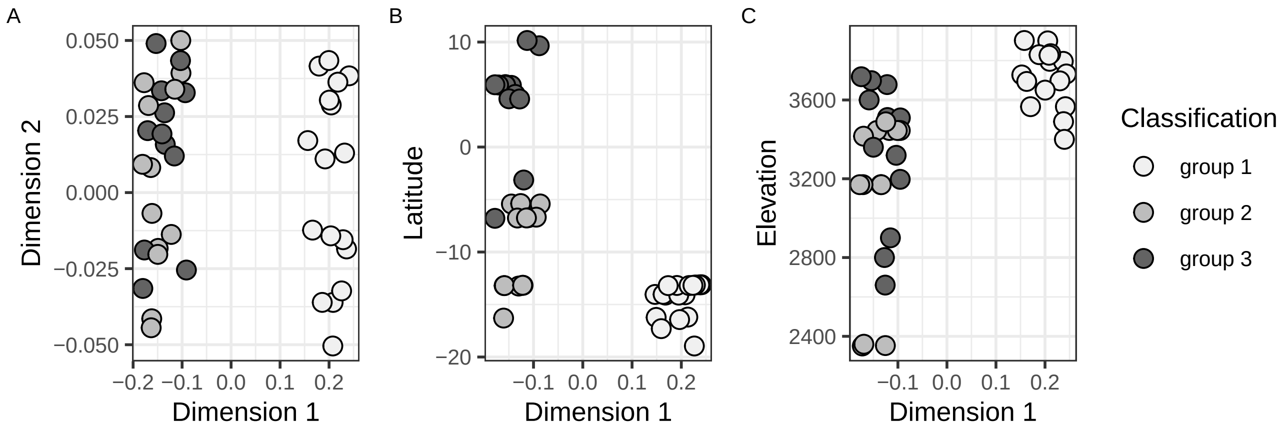

Figure S43: Gaussian finite mixture modeling (GFMM) for genogroup delimitation. Visualization of the genogroups (shades) identified by the best fit Gaussian mixture model (GMM). A) genogroups in the space defined by two axes obtained by non-metric multidimensional scaling (NMDS); B) genogroups in the space defined by NMDS axis 1 and latitude; C) genogroups in the space defined by NMDS axis 1 and elevation.

Return to Clade IV Genomics: model-based species discovery

4.5.9 Fig S44: Genogroup delimitation. Cladogenesis to anagenesis model

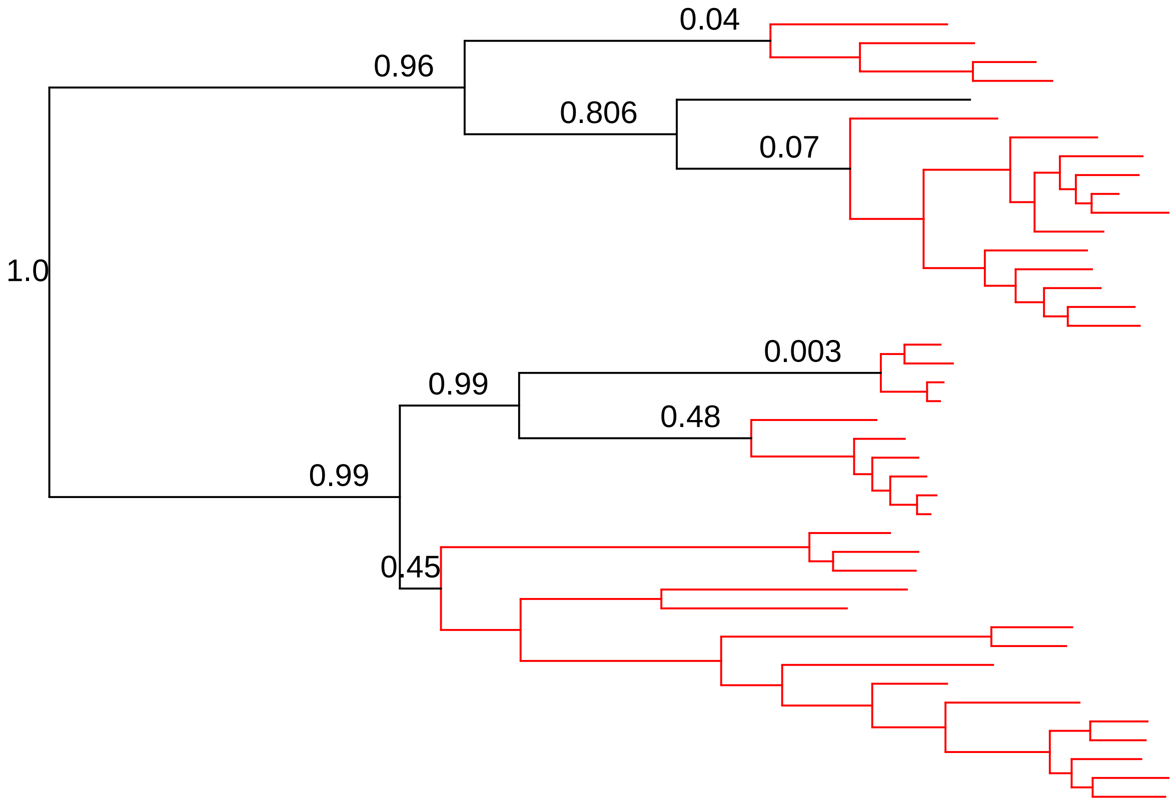

Figure S44: Phylogenetic modeling for genogroup delimitation. Midpoint-rooted phylogenetic tree showing genogroups in red. Values correspond to nodes at the transition point between cladogenesis (between species) to anagenesis (within species). Values closer to 0 indicate that the node was identified as a transition to anagenesis summarized over 500 delimitations.

Return to Clade IV Genomics: model-based species discovery

4.5.10 Fig S45: Genogroup delimitation: Reproductive isolation model

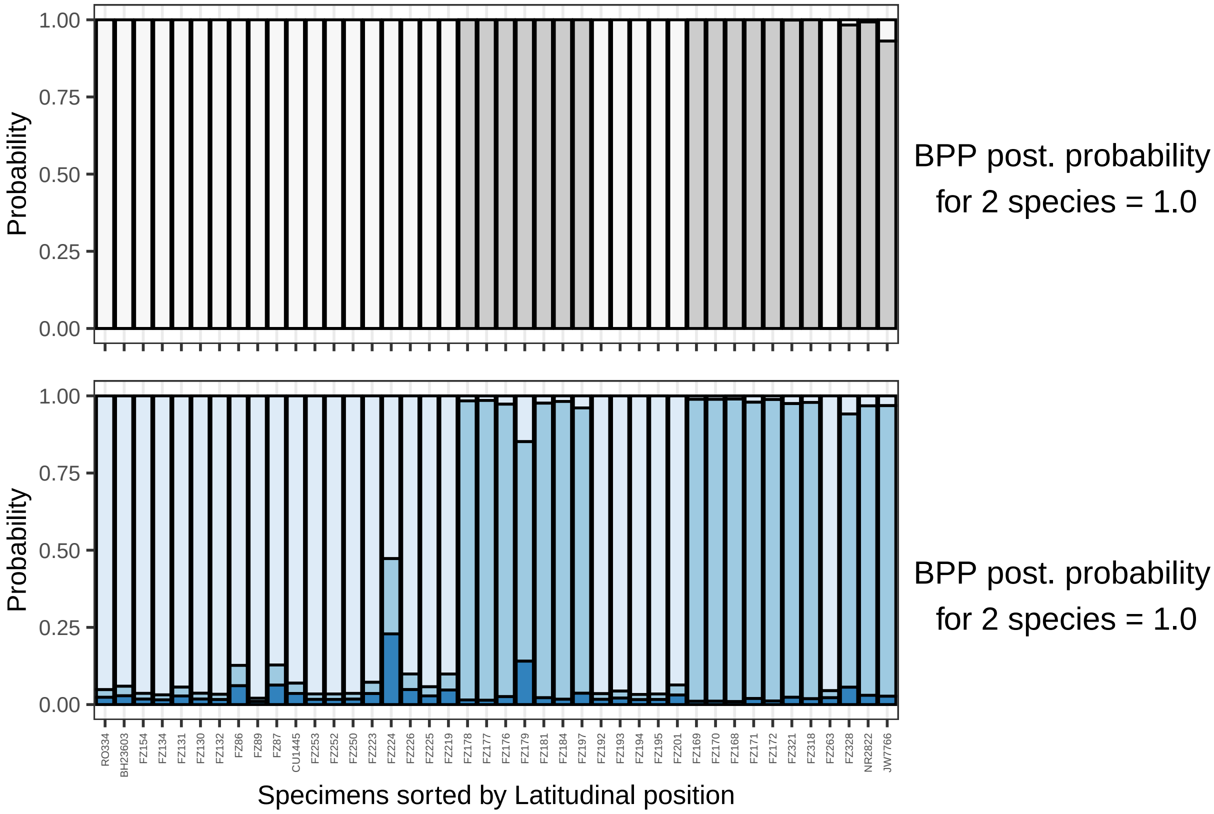

Figure S45: Population genetic modeling for genogroup delimitation. Top panel: assignment of specimens to demes according to `STRUCTURE` and posterior probability of species delimitation modeling according to `BPP` using these demes. Bottom panel: assignment of specimens to demes according to `MAVERICK` and posterior probability of species delimitation modeling according to `BPP` using these demes. Specimens are sorted from north (left) to south (right) according to locality of collection.

Return to Clade IV Genomics: model-based species discovery

4.5.11 Fig S46: Data integration

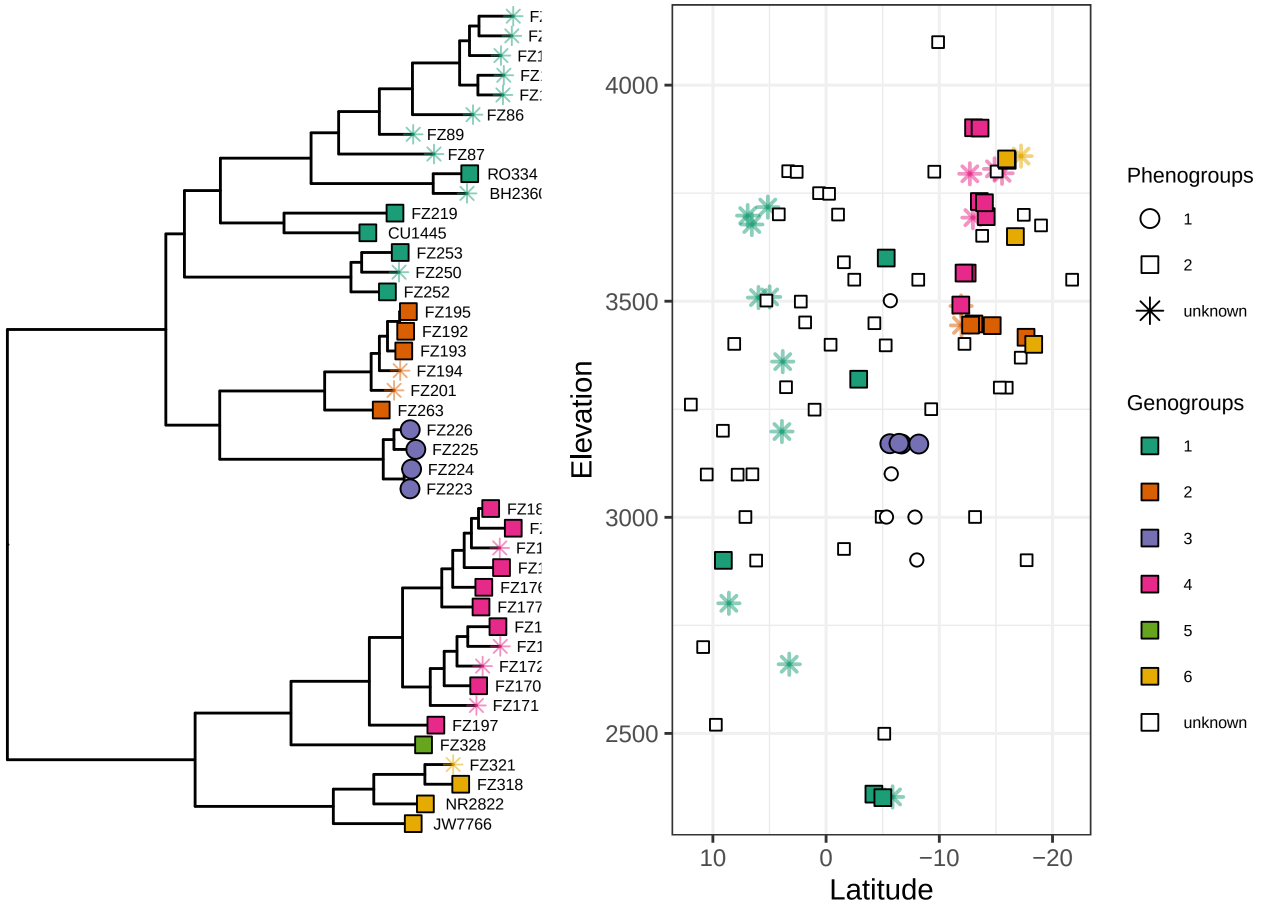

Figure S46: Integration of phenotypic and genomic data with spatial information and evolutionary history. All specimens are assigned to their corresponding best fit phenogroup (shapes) and genogroup (colors). Specimens without phenotypic or genomic data (unknown specimens) are shown as asterisks and empty shapes, accordingly. Specimens are shown as tips of the maximum likelihood tree (left) used in the CA model analysis and mapped along latitude and elevation (right). Specimens assigned to a single phenogroup and a single genogroup delineate species that we determined as 'good species'. Specimens assigned to a single phenogroup across multiple genogroups delineate species that we determined as 'phenotypic cryptic species'. Specimens assigned to a single genogroup across multiple phenogroups delineate species that we determined as 'genetic cryptic species'.

Return to Clade IV Data integration

4.6 Clade V

4.6.1 Fig S47: Taxon sampling

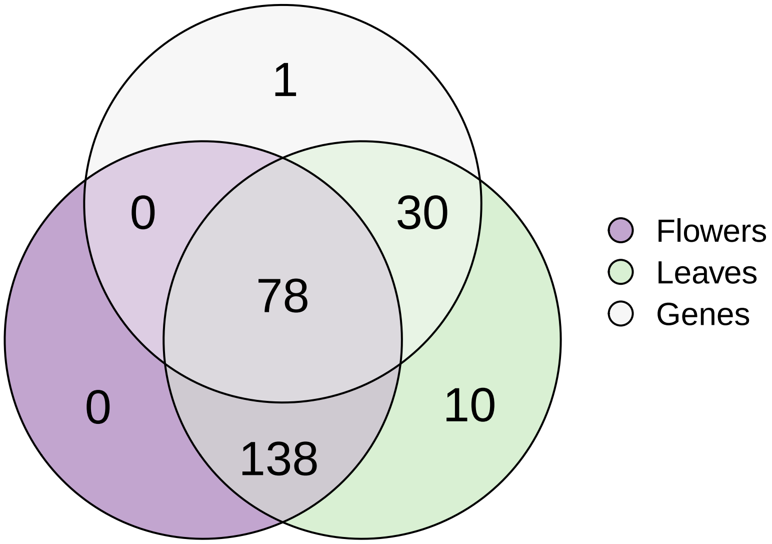

Figure S47: Specimens sampled according to three types of data. Specimens outside the Flowers category represent sterile specimens.

Return to Clade V Sampling

4.6.2 Fig S48: Geographic distribution

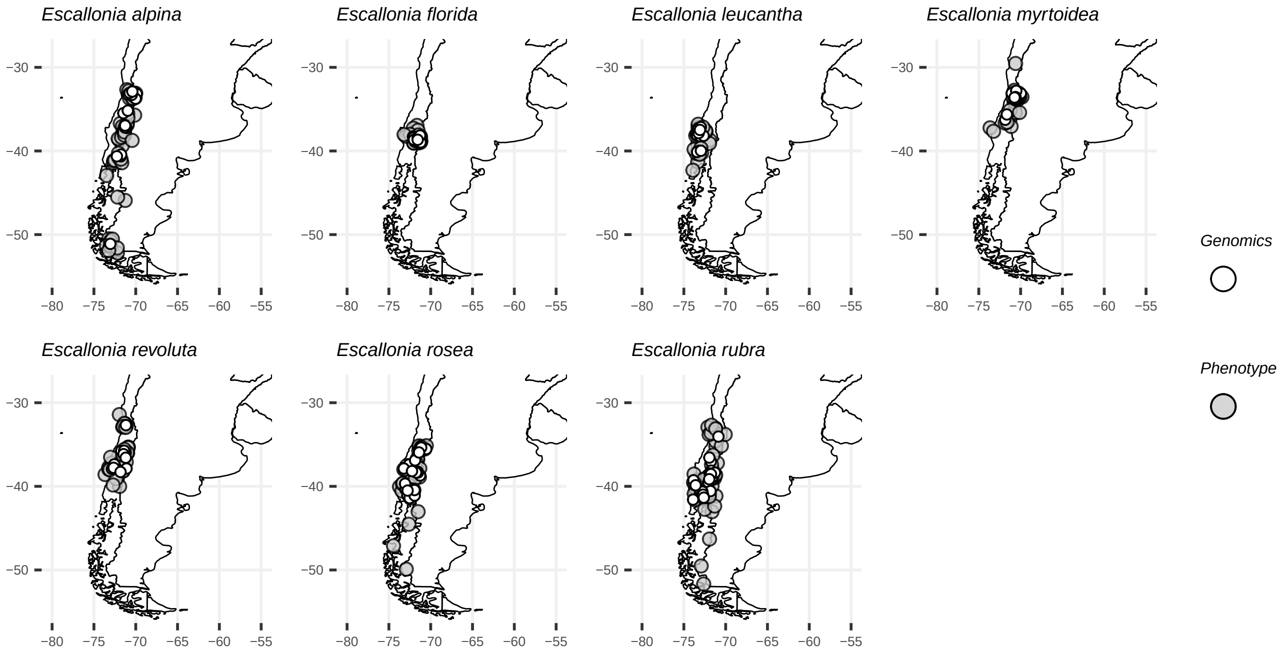

Figure S48: Geographic distribution of specimens sampled for this clade according to taxonomic species. Filled symbols indicate specimens used in phenotypic analyses and empty symbols specimens used in genomic analyses.

Return to Clade V Sampling

4.6.3 Fig S49: Current state of taxonomic species with phenotypic data

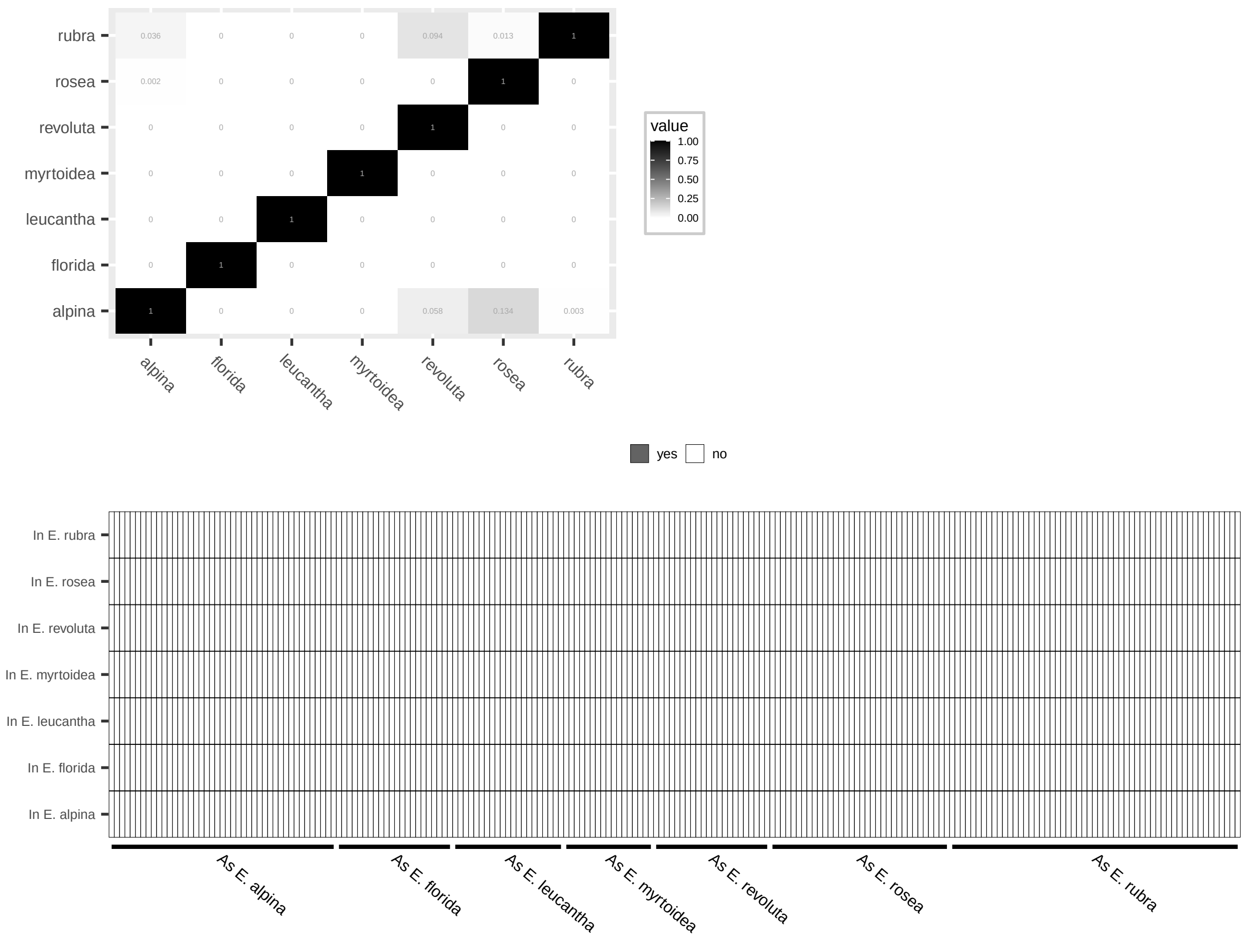

Figure S49: Assessment of current state of taxonomic species with phenotypic data. Top panel: Pairwise overlap among 10-cubes describing geometrically each taxonomic species. Bottom panel: Matching-prediction analysis with each cell along the x-axis representing specimens sorted according to taxonomic species and the 10-cubes corresponding to each taxonomic species along the y-axis. If a specimen matches the prediction of the monograph (i.e., it is inside a 10-cube), the corresponding cell is shaded. If the specimen does not match the prediction, the cell is empty.

Return to Clade V Current state of taxonomic species

4.6.4 Fig S50: Phenogroup delimitation: Gaussian finite mixture modeling

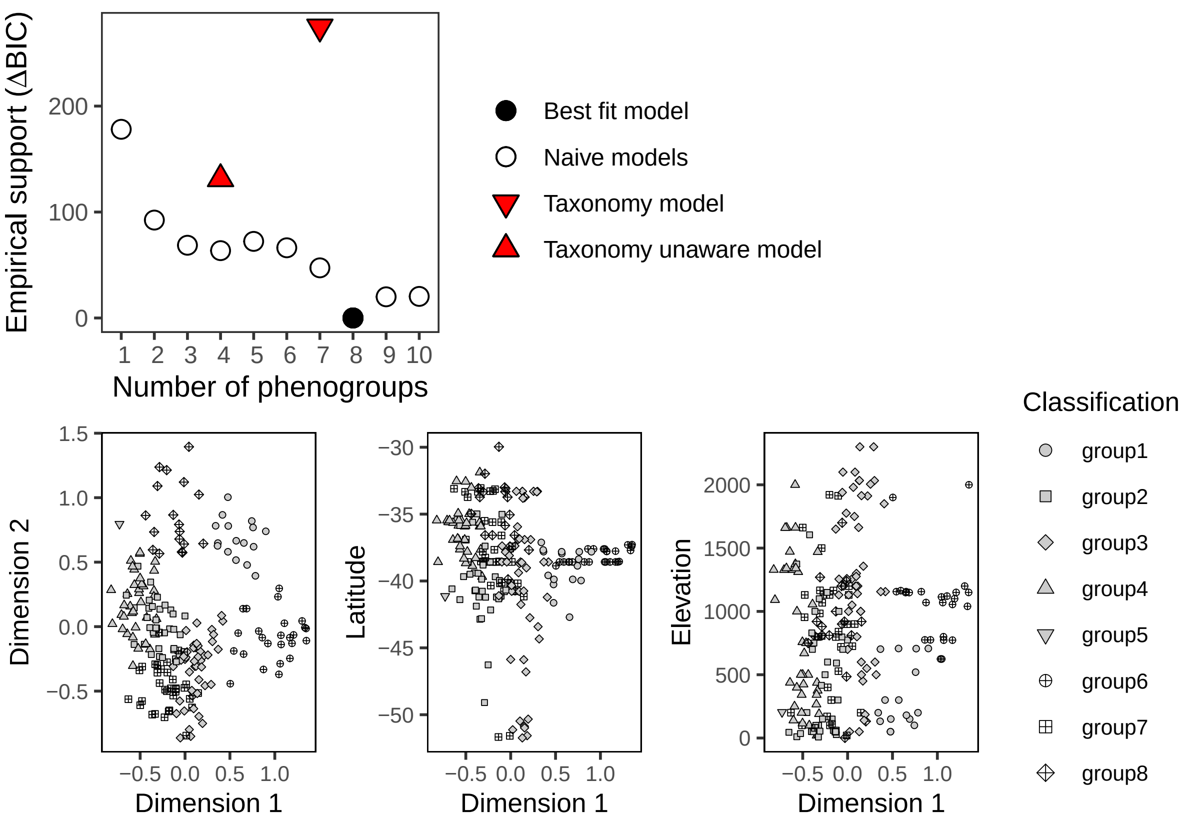

Figure S50: Gaussian finite mixture modeling (GFMM) for phenogroup delimitation and model selection using the Bayesian information criterion (BIC). Top panel: empirical support (ordinate) for Gaussian mixture models (GMM) assuming distinct number of phenogroups (abscissa). Each GMM specifies different number of phenogroups (shapes). Empirical support was measured as difference in BIC relative to the best model (ΔBIC $= 0$). Bottom panel: Visualization of the phenogroups (shapes) identified by the best fit GMM; left panel shows phenogroups in the space defined by two axes obtained by linear discriminat analysis (to maximize separation and visualization), middle panel shows phenogroups in the space defined by discriminant axis 1 and latitude, and right panel shows phenogroups in the space defined by discriminant axis 1 and elevation.

Return to Clade V Phenomics: model-based species discovery

4.6.5 Fig S51: Sensitivity tests with 75% missing data

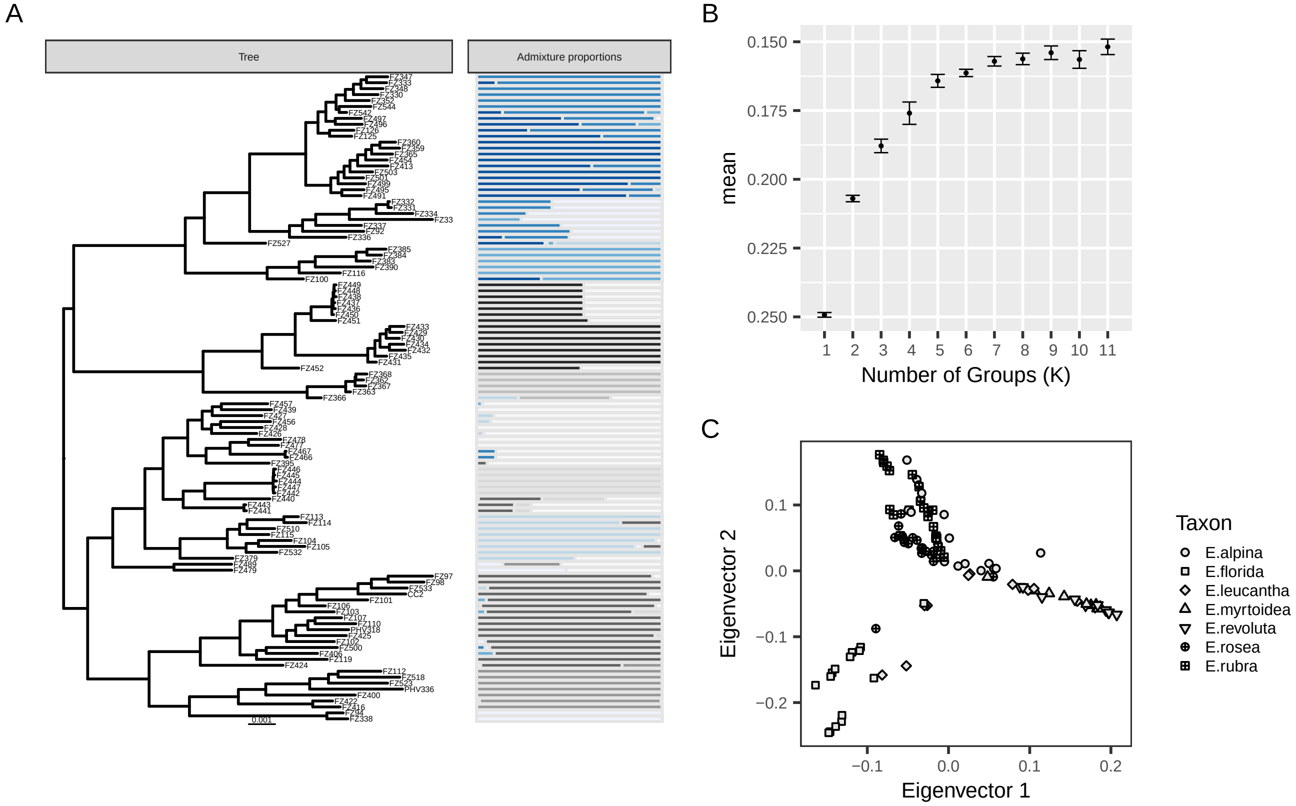

Figure S51: Impact of missing data (75%) on data analysis. A) maximum likelihood phylogenetic tree of specimens assigned to demes according to best `ADMIXTURE` run. B) Mean cross validation error with 95% confidence interval (ordinate) for ten replicate runs of `ADMIXTURE` assuming differnt number of demes (K) (abscissa); C) Scatterplot of Principal Component Analysis (PCA) projected along the first two axes.

Return to Clade V Genomics: sensitivity tests

4.6.6 Fig S52: Sensitivity tests with 50% missing data

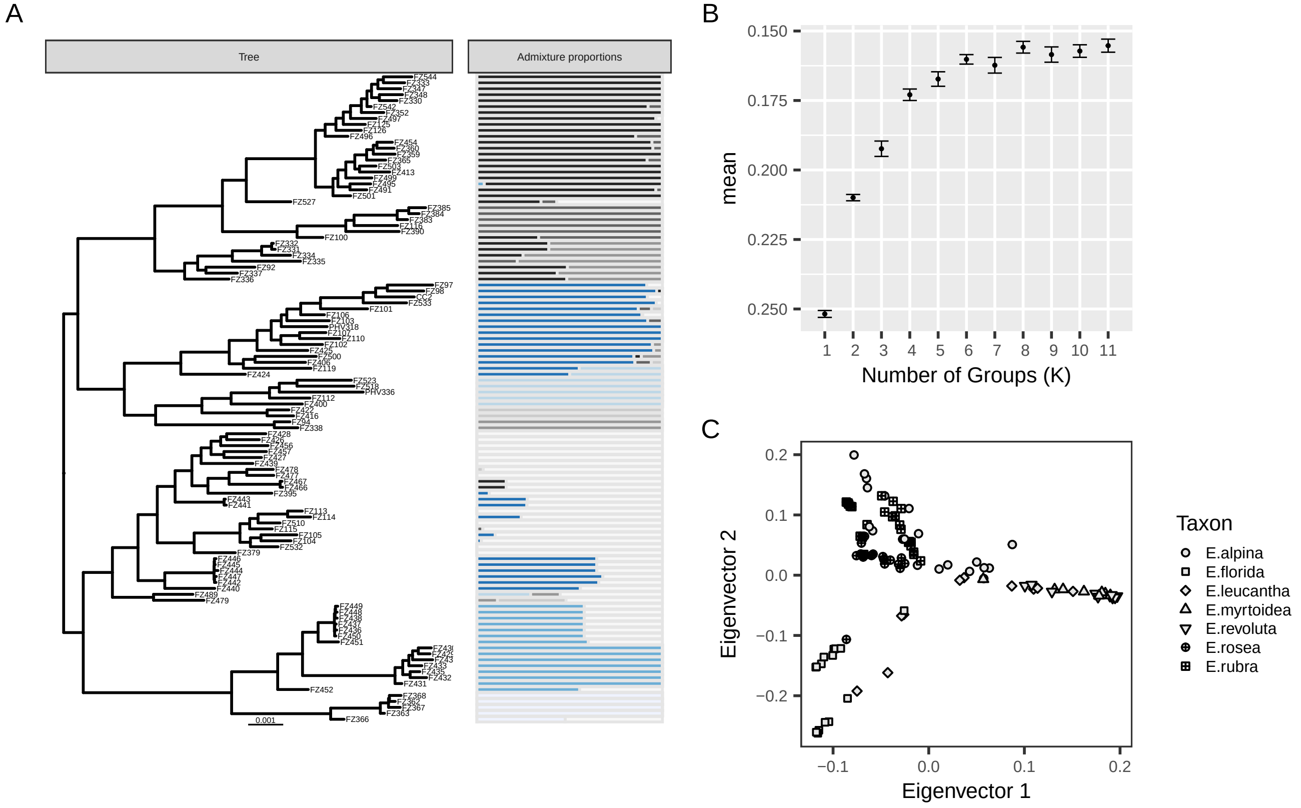

Figure S52: Impact of missing data (50%) on data analysis. A) maximum likelihood phylogenetic tree of specimens assigned to demes according to best `ADMIXTURE` run. B) Mean cross validation error with 95% confidence interval (ordinate) for ten replicate runs of `ADMIXTURE` assuming differnt number of demes (K) (abscissa); C) Scatterplot of Principal Component Analysis (PCA) projected along the first two axes.

Return to Clade V Genomics: sensitivity tests

4.6.7 Fig S53: Sensitivity tests with 25% missing data

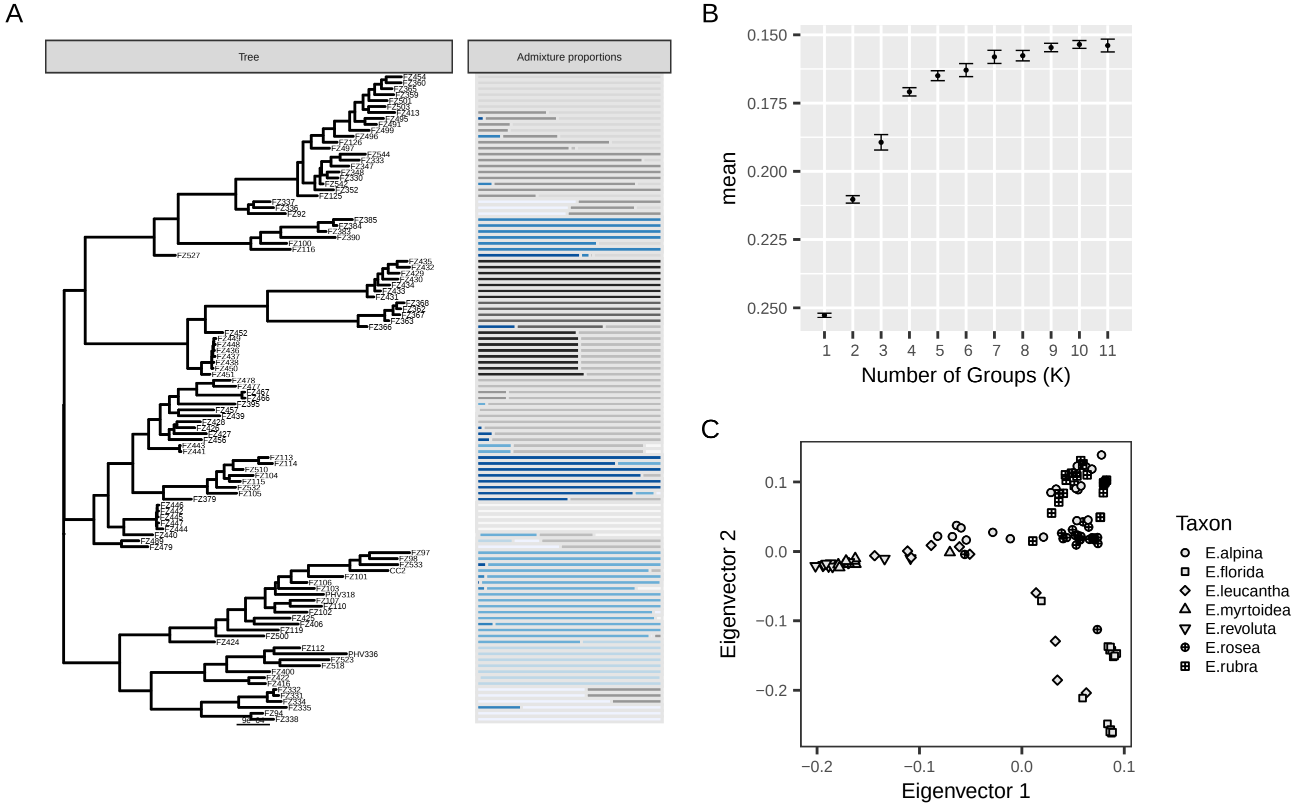

Figure S53: Impact of missing data (25%) on data analysis. A) maximum likelihood phylogenetic tree of specimens assigned to demes according to best `ADMIXTURE` run. B) Mean cross validation error with 95% confidence interval (ordinate) for ten replicate runs of `ADMIXTURE` assuming differnt number of demes (K) (abscissa); C) Scatterplot of Principal Component Analysis (PCA) projected along the first two axes.

Return to Clade V Genomics: sensitivity tests

4.6.8 Fig S54: Genogroup delimitation: Genotypic cluster model

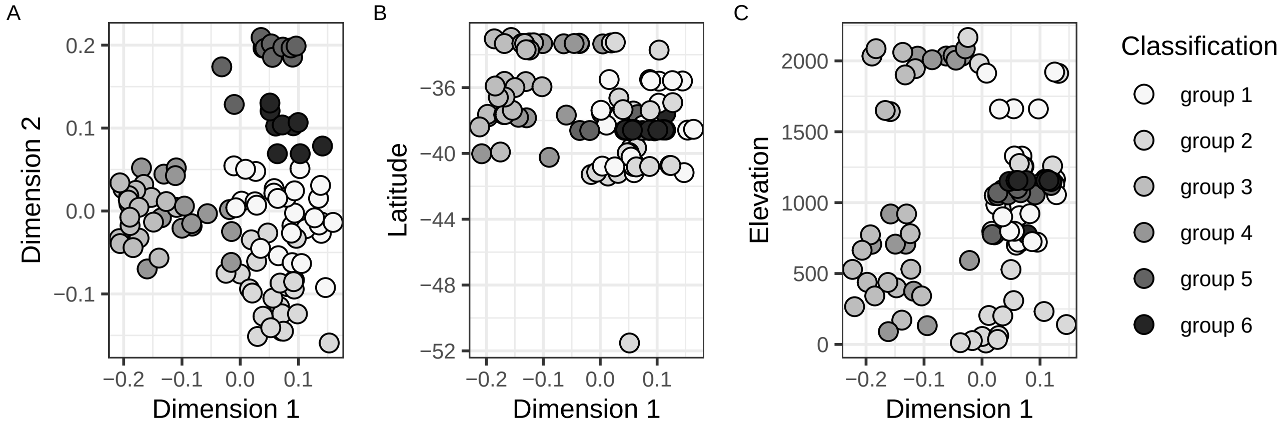

Figure S54: Gaussian finite mixture modeling (GFMM) for genogroup delimitation. Visualization of the genogroups (shades) identified by the best fit Gaussian mixture model (GMM). A) genogroups in the space defined by two axes obtained by non-metric multidimensional scaling (NMDS); B) genogroups in the space defined by NMDS axis 1 and latitude; C) genogroups in the space defined by NMDS axis 1 and elevation.

Return to Clade V Genomics: model-based species discovery

4.6.9 Fig S55: Genogroup delimitation. Cladogenesis to anagenesis model

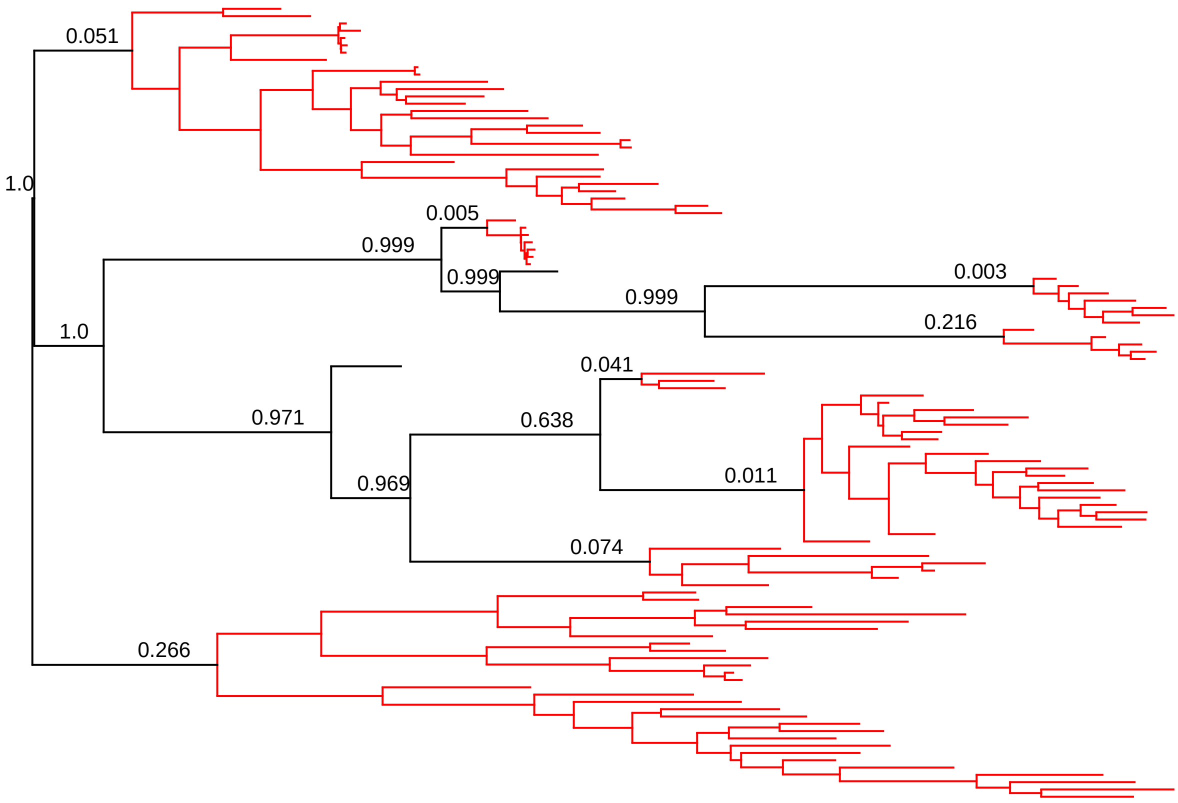

Figure S55: Phylogenetic modeling for genogroup delimitation. Midpoint-rooted phylogenetic tree showing genogroups in red. Values correspond to nodes at the transition point between cladogenesis (between species) to anagenesis (within species). Values closer to 0 indicate that the node was identified as a transition to anagenesis summarized over 500 delimitations.

Return to Clade V Genomics: model-based species discovery

4.6.10 Fig S56: Genogroup delimitation: Reproductive isolation model

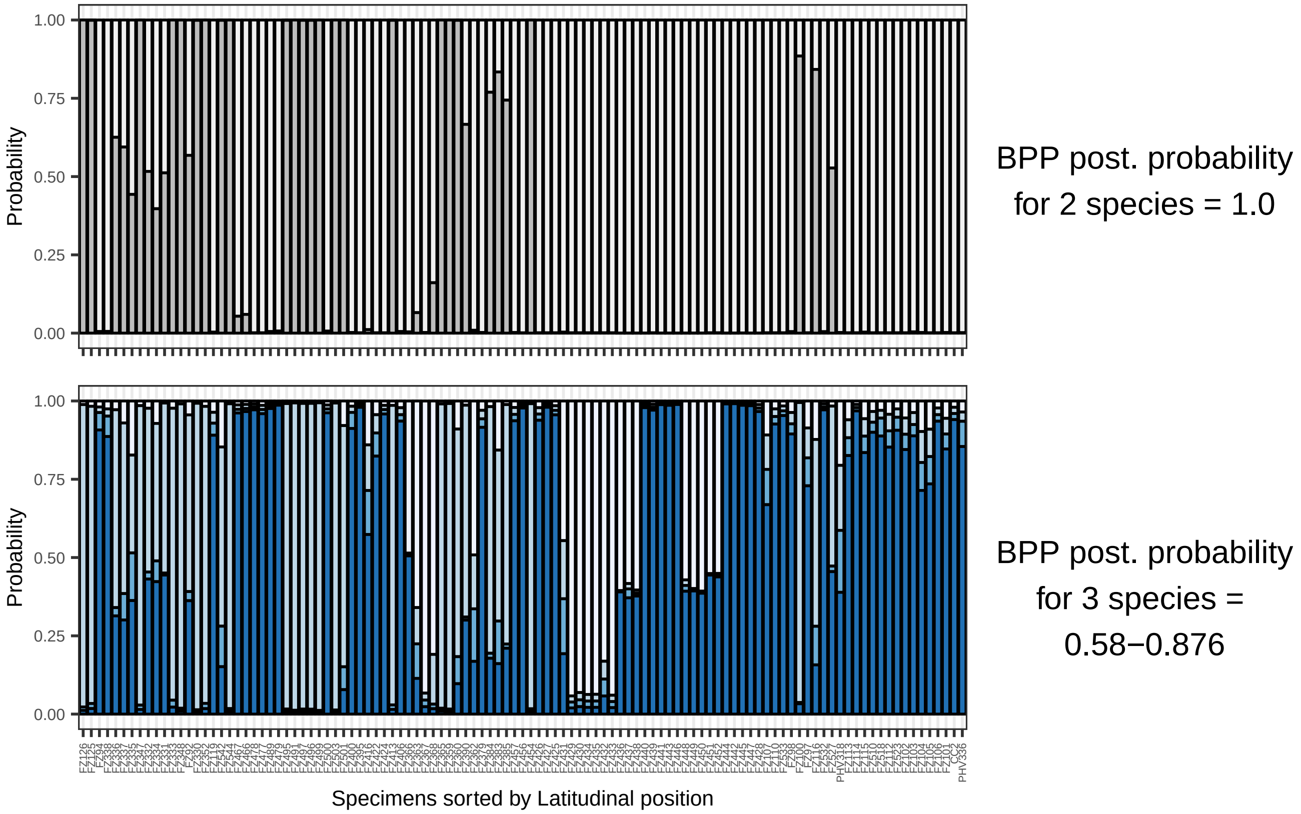

Figure S56: Population genetic modeling for genogroup delimitation. Top panel: assignment of specimens to demes according to `STRUCTURE` and posterior probability of species delimitation modeling according to `BPP` using these demes. Bottom panel: assignment of specimens to demes according to `MAVERICK` and posterior probability of species delimitation modeling according to `BPP` using these demes. Specimens are sorted from north (left) to south (right) according to locality of collection.

Return to Clade V Genomics: model-based species discovery

4.6.11 Fig S57: Data integration

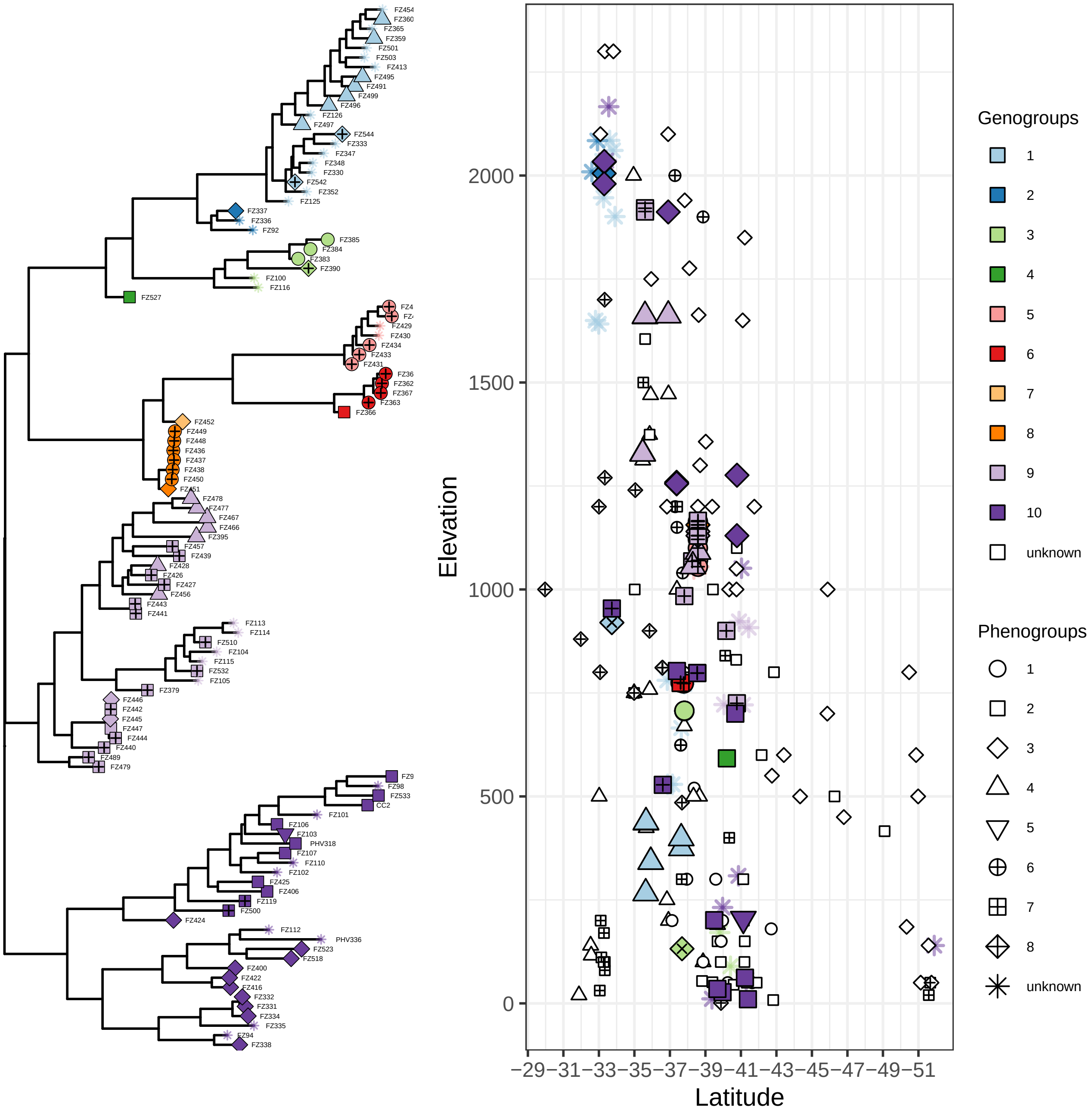

Figure S57: Integration of phenotypic and genomic data with spatial information and evolutionary history. All specimens are assigned to their corresponding best fit phenogroup (shapes) and genogroup (colors). Specimens without phenotypic or genomic data (unknown specimens) are shown as asterisks and empty shapes, accordingly. Specimens are shown as tips of the maximum likelihood tree (left) used in the CA model analysis and mapped along latitude and elevation (right). Specimens assigned to a single phenogroup and a single genogroup delineate species that we determined as 'good species'. Specimens assigned to a single phenogroup across multiple genogroups delineate species that we determined as 'phenotypic cryptic species'. Specimens assigned to a single genogroup across multiple phenogroups delineate species that we determined as 'genetic cryptic species'.

Return to Clade V Data integration

4.7 Clade VI

4.7.1 Fig S58: Taxon sampling

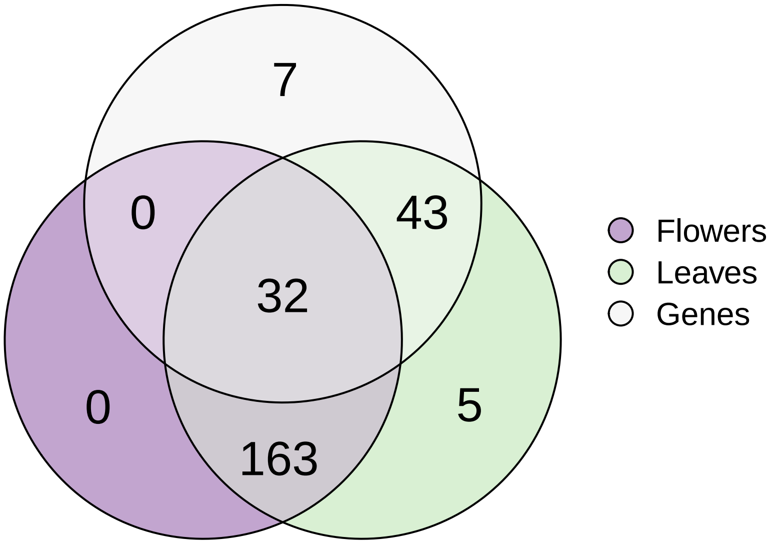

Figure S58: Specimens sampled according to three types of data. Specimens outside the Flowers category represent sterile specimens.

Return to Clade VI Sampling

4.7.2 Fig S59: Geographic distribution

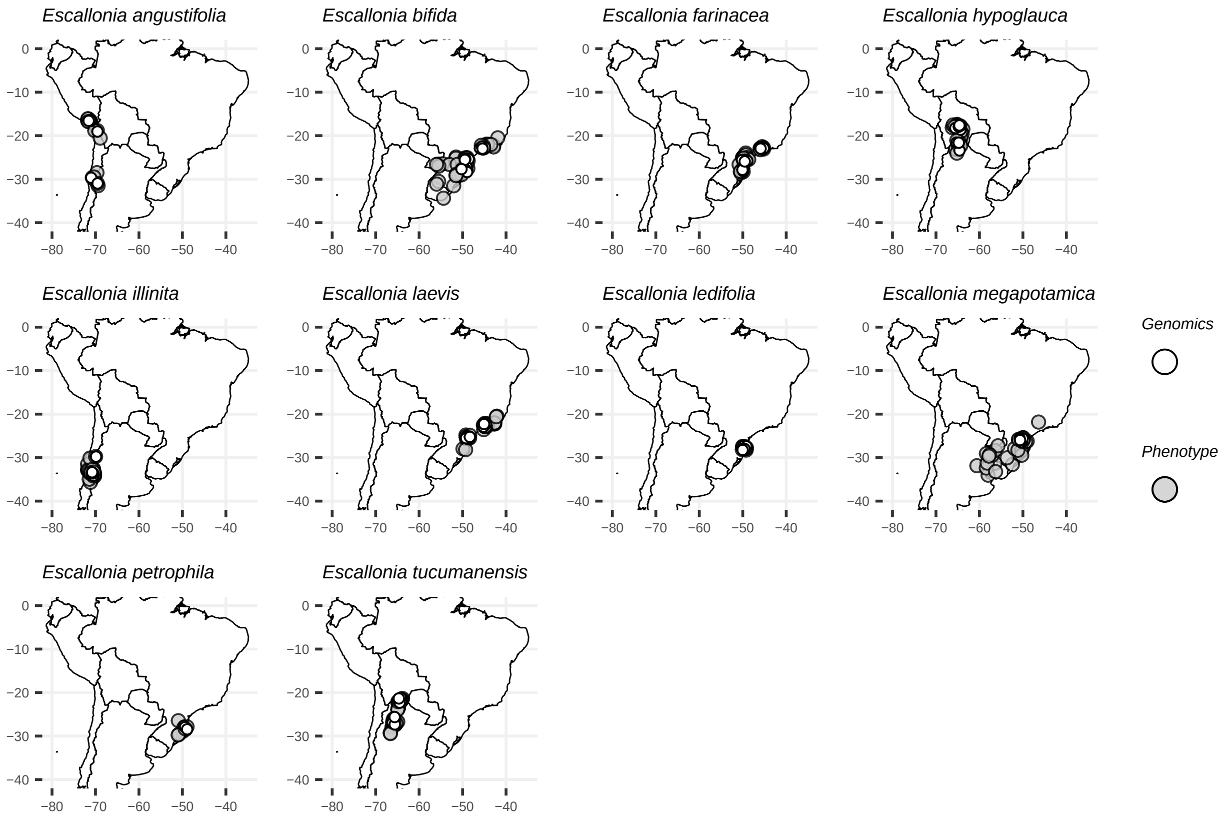

Figure S59: Geographic distribution of specimens sampled for this clade according to taxonomic species. Filled symbols indicate specimens used in phenotypic analyses and empty symbols specimens used in genomic analyses.

Return to Clade VI Sampling

4.7.3 Fig S60: Current state of taxonomic species with phenotypic data

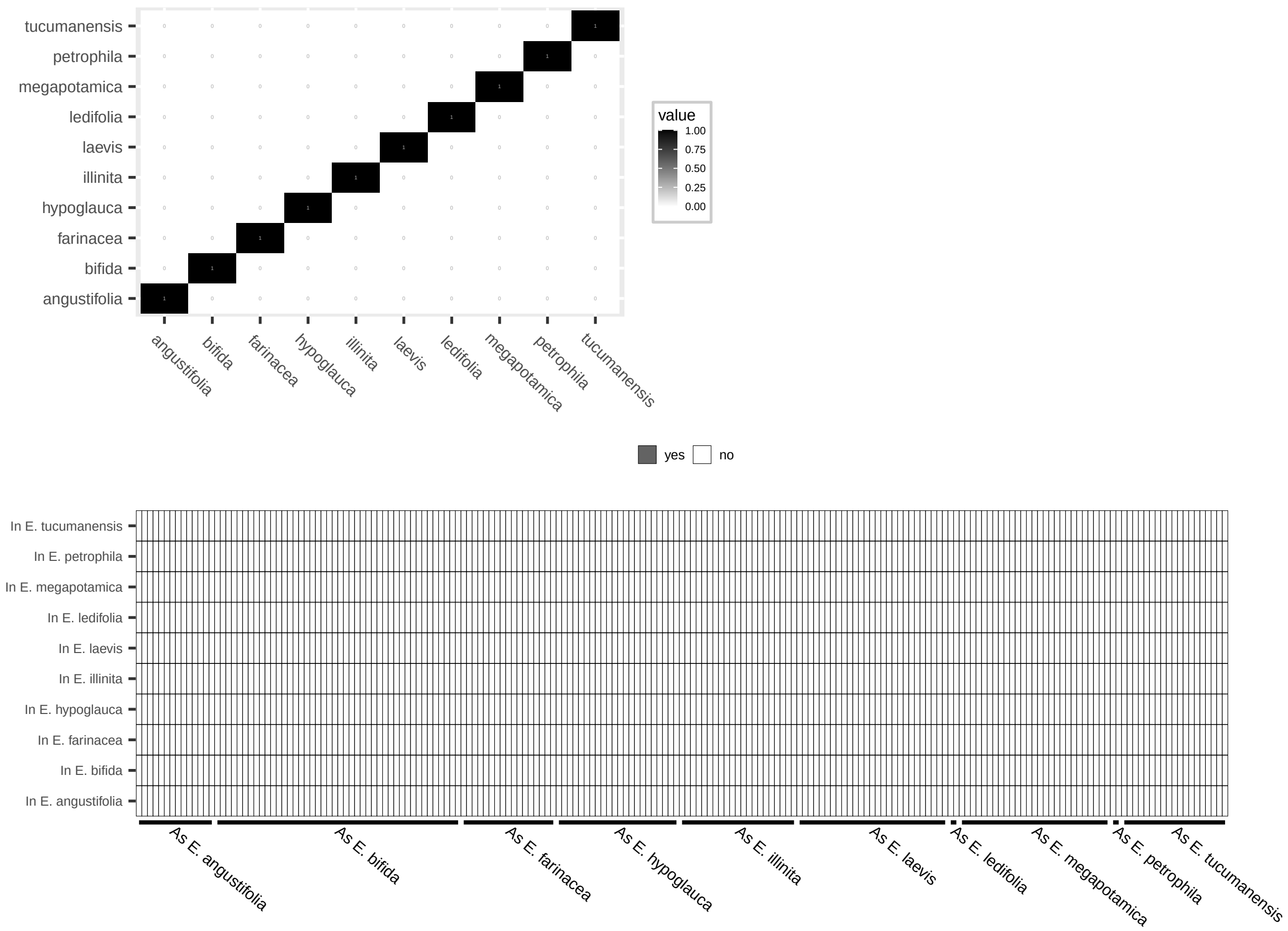

Figure S60: Assessment of current state of taxonomic species with phenotypic data. Top panel: Pairwise overlap among 10-cubes describing geometrically each taxonomic species. Bottom panel: Matching-prediction analysis with each cell along the x-axis representing specimens sorted according to taxonomic species and the 10-cubes corresponding to each taxonomic species along the y-axis. If a specimen matches the prediction of the monograph (i.e., it is inside a 10-cube), the corresponding cell is shaded. If the specimen does not match the prediction, the cell is empty.

Return to Clade VI Current state of taxonomic species

4.7.4 Fig S61: Phenogroup delimitation: Gaussian finite mixture modeling

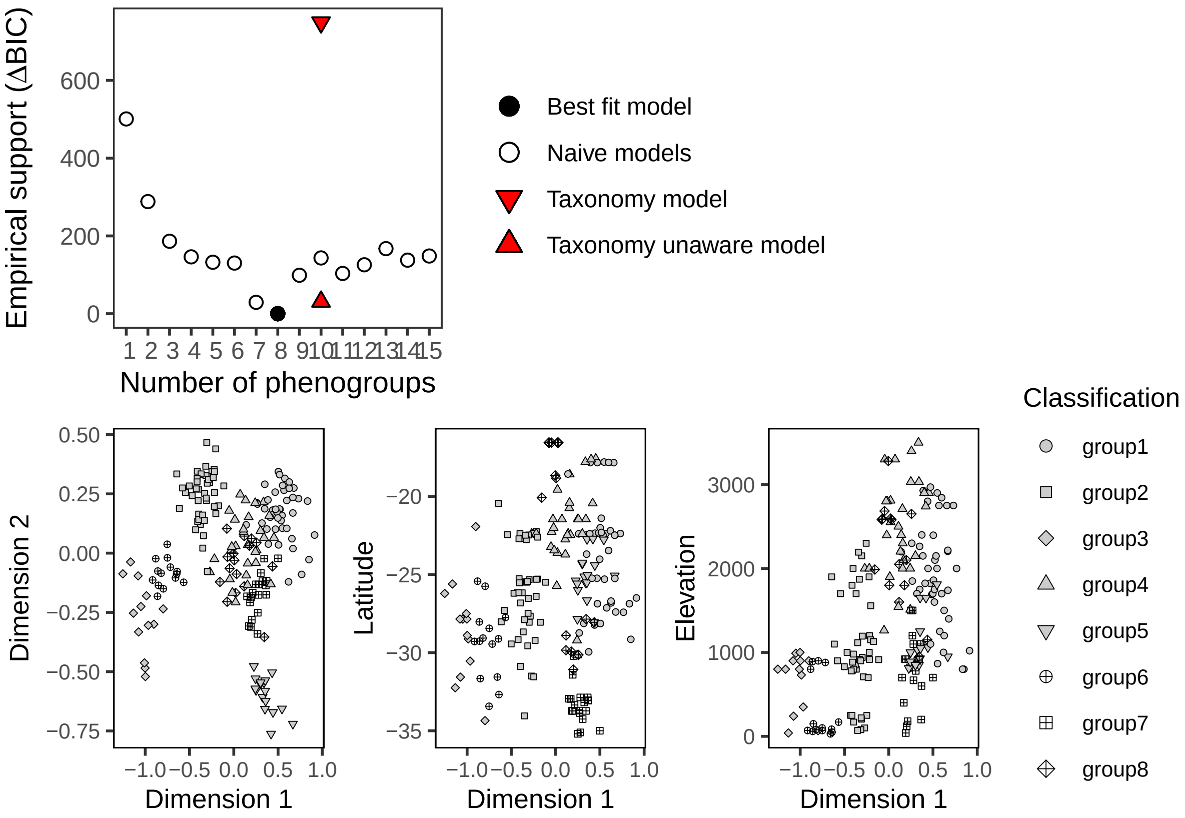

Figure S61: Gaussian finite mixture modeling (GFMM) for phenogroup delimitation and model selection using the Bayesian information criterion (BIC). Top panel: empirical support (ordinate) for Gaussian mixture models (GMM) assuming distinct number of phenogroups (abscissa). Each GMM specifies different number of phenogroups (shapes). Empirical support was measured as difference in BIC relative to the best model ($\Delta BIC = 0$). Bottom panel: Visualization of the phenogroups (shapes) identified by the best fit GMM; left panel shows phenogroups in the space defined by two axes obtained by linear discriminat analysis (to maximize separation and visualization), middle panel shows phenogroups in the space defined by discriminant axis 1 and latitude, and right panel shows phenogroups in the space defined by discriminant axis 1 and elevation.

Return to Clade VI Phenomics: model-based species discovery

4.7.5 Fig S62: Sensitivity tests with 75% missing data

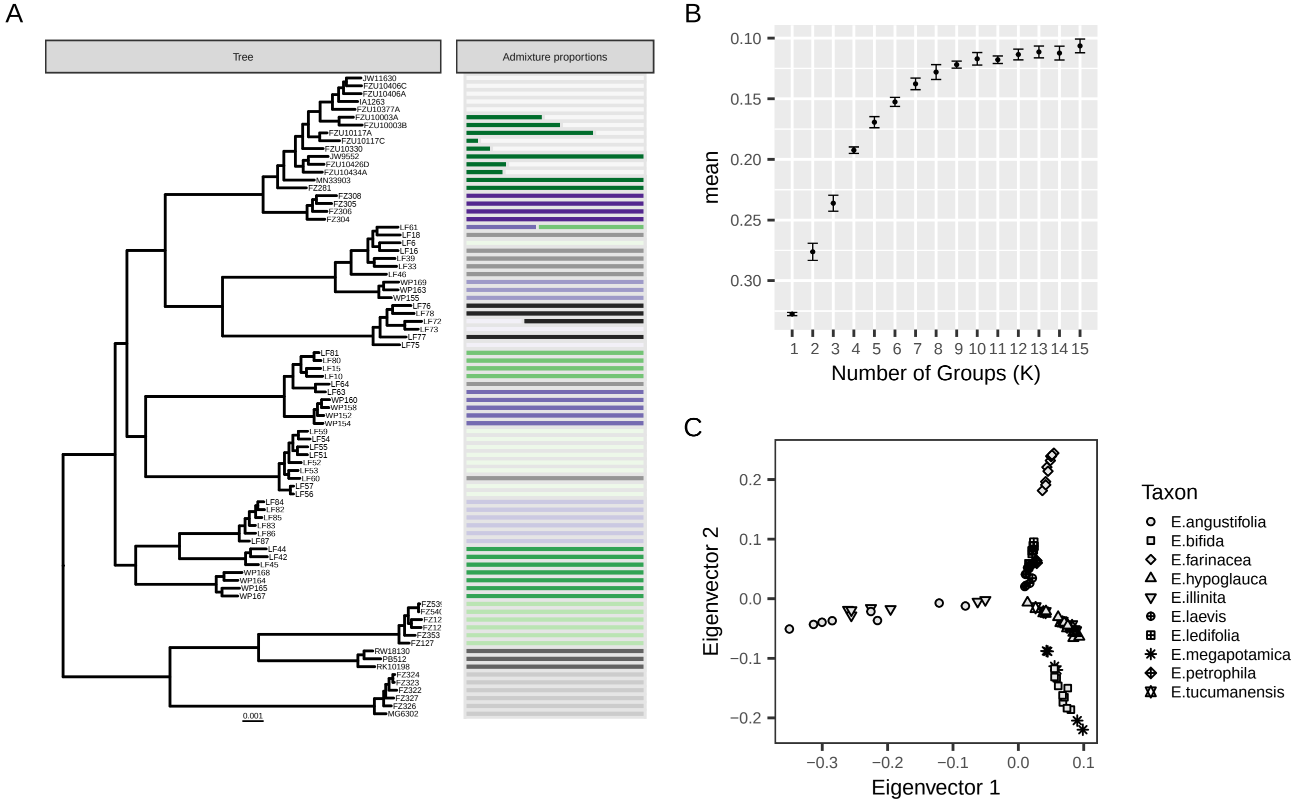

Figure S62: Impact of missing data (75%) on data analysis. A) maximum likelihood phylogenetic tree of specimens assigned to demes according to best `ADMIXTURE` run. B) Mean cross validation error with 95% confidence interval (ordinate) for ten replicate runs of `ADMIXTURE` assuming diffent number of demes (K) (abscissa); C) Scatterplot of Principal Component Analysis (PCA) projected along the first two axes.

Return to Clade VI Genomics: sensitivity tests

4.7.6 Fig S63: Sensitivity tests with 50% missing data

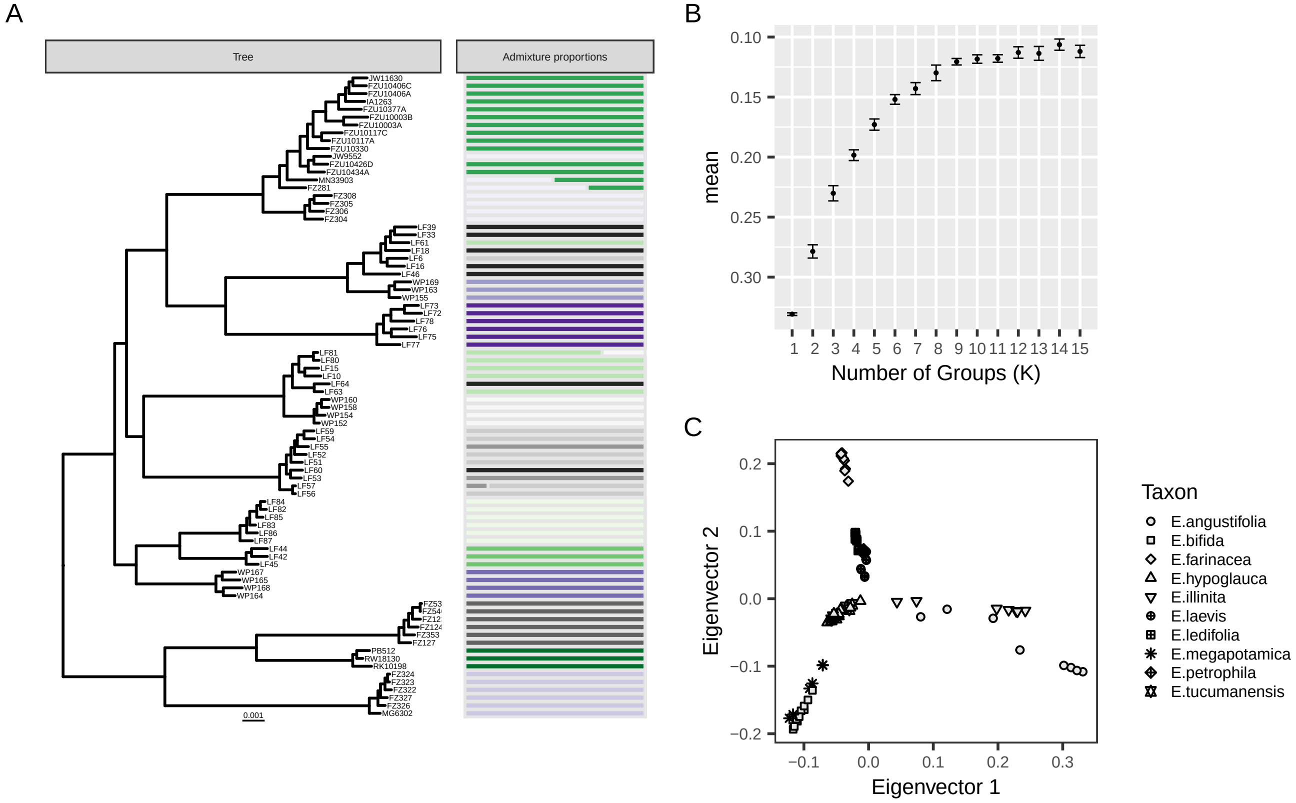

Figure S63: Impact of missing data (50%) on data analysis. A) maximum likelihood phylogenetic tree of specimens assigned to demes according to best `ADMIXTURE` run. B) Mean cross validation error with 95% confidence interval (ordinate) for ten replicate runs of `ADMIXTURE` assuming differnt number of demes (K) (abscissa); C) Scatterplot of Principal Component Analysis (PCA) projected along the first two axes.

Return to Clade VI Genomics: sensitivity tests

4.7.7 Fig S64: Sensitivity tests with 25% missing data

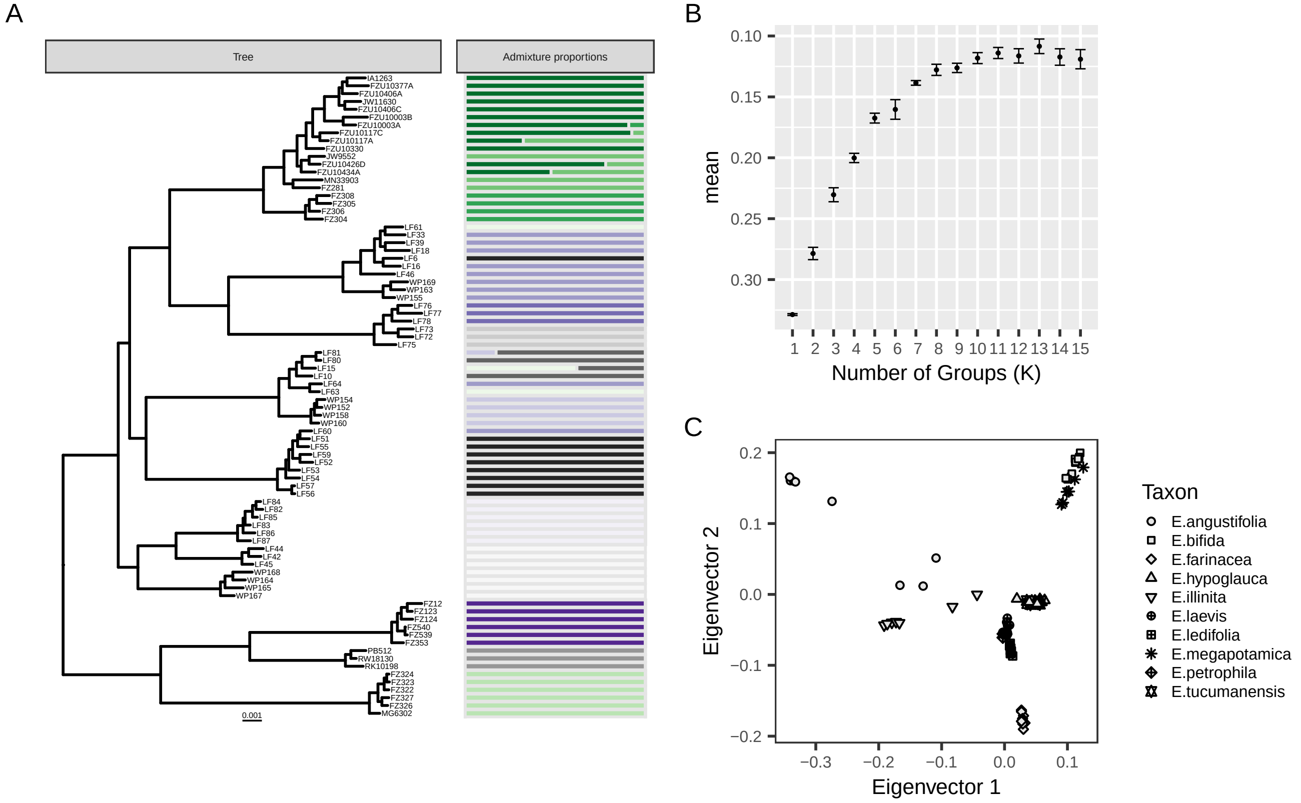

Figure S64: Impact of missing data (25%) on data analysis. A) maximum likelihood phylogenetic tree of specimens assigned to demes according to best `ADMIXTURE` run. B) Mean cross validation error with 95% confidence interval (ordinate) for ten replicate runs of `ADMIXTURE` assuming diffent number of demes (K) (abscissa); C) Scatterplot of Principal Component Analysis (PCA) projected along the first two axes.

Return to Clade VI Genomics: sensitivity tests

4.7.8 Fig S65: Genogroup delimitation: Genotypic cluster model

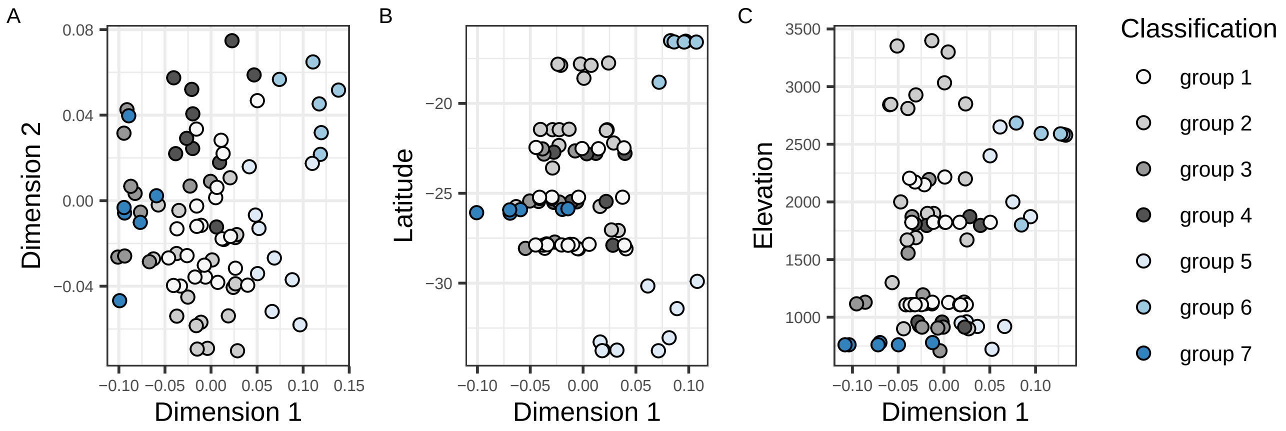

Figure S65: Gaussian finite mixture modeling (GFMM) for genogroup delimitation. Visualization of the genogroups (shades) identified by the best fit Gaussian mixture model (GMM). A) genogroups in the space defined by two axes obtained by non-metric multidimensional scaling (NMDS); B) genogroups in the space defined by NMDS axis 1 and latitude; C) genogroups in the space defined by NMDS axis 1 and elevation.

Return to Clade VI Genomics: model-based species discovery

4.7.9 Fig S66: Genogroup delimitation. Cladogenesis to anagenesis model

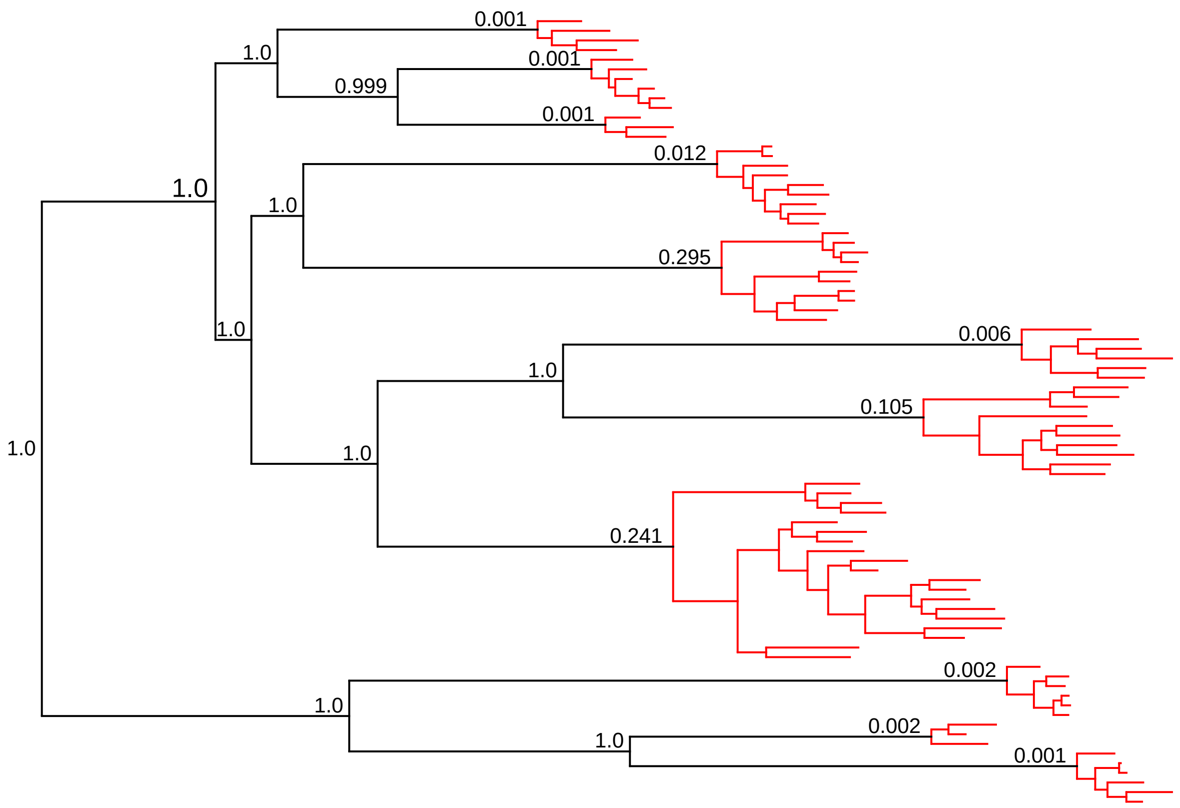

Figure S66: Phylogenetic modeling for genogroup delimitation. Midpoint-rooted phylogenetic tree showing genogroups in red. Values correspond to nodes at the transition point between cladogenesis (between species) to anagenesis (within species). Values closer to 0 indicate that the node was identified as a transition to anagenesis summarized over 500 delimitations.

Return to Clade VI Genomics: model-based species discovery

4.7.10 Fig S67: Genogroup delimitation: Reproductive isolation model

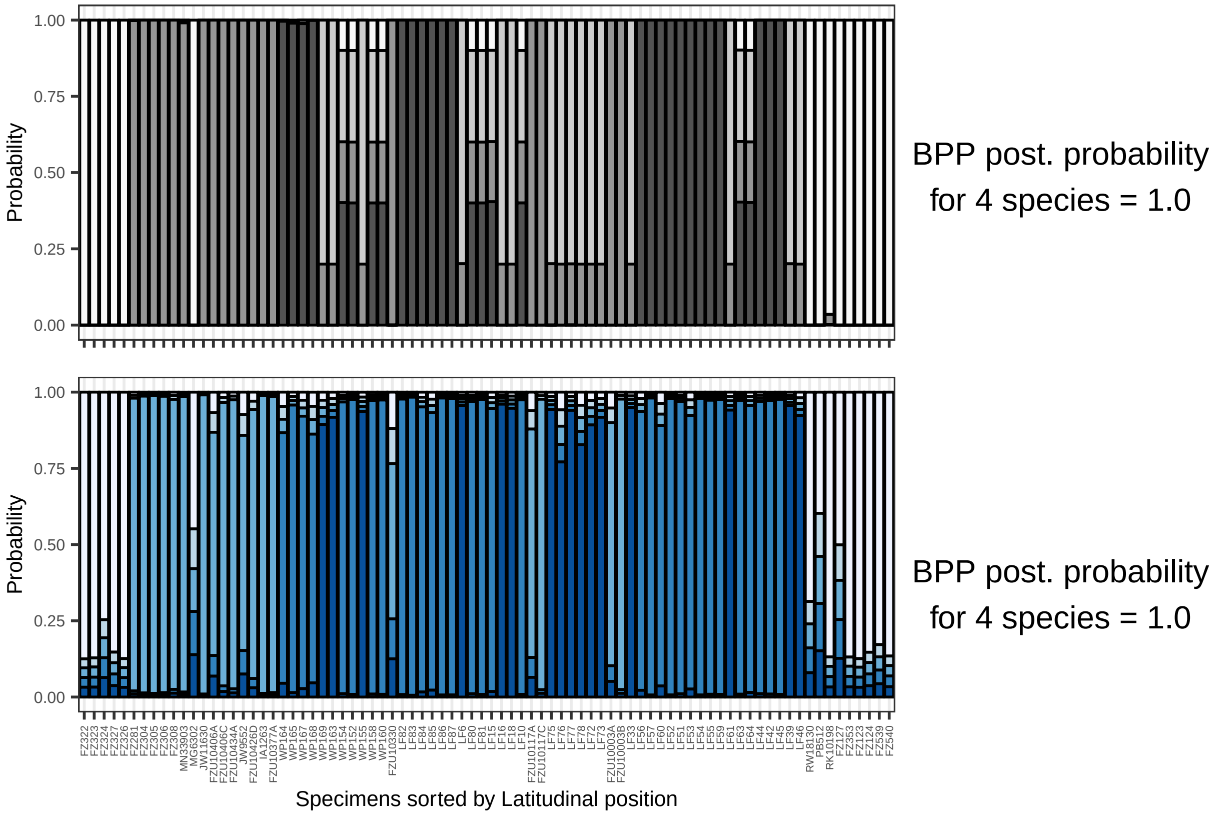

Figure S67: Population genetic modeling for genogroup delimitation. Top panel: assignment of specimens to demes according to `STRUCTURE` and posterior probability of species delimitation modeling according to `BPP` using these demes. Bottom panel: assignment of specimens to demes according to `MAVERICK` and posterior probability of species delimitation modeling according to `BPP` using these demes. Specimens are sorted from north (left) to south (right) according to locality of collection.

Return to Clade VI Genomics: model-based species discovery

4.7.11 Fig S68: Data integration

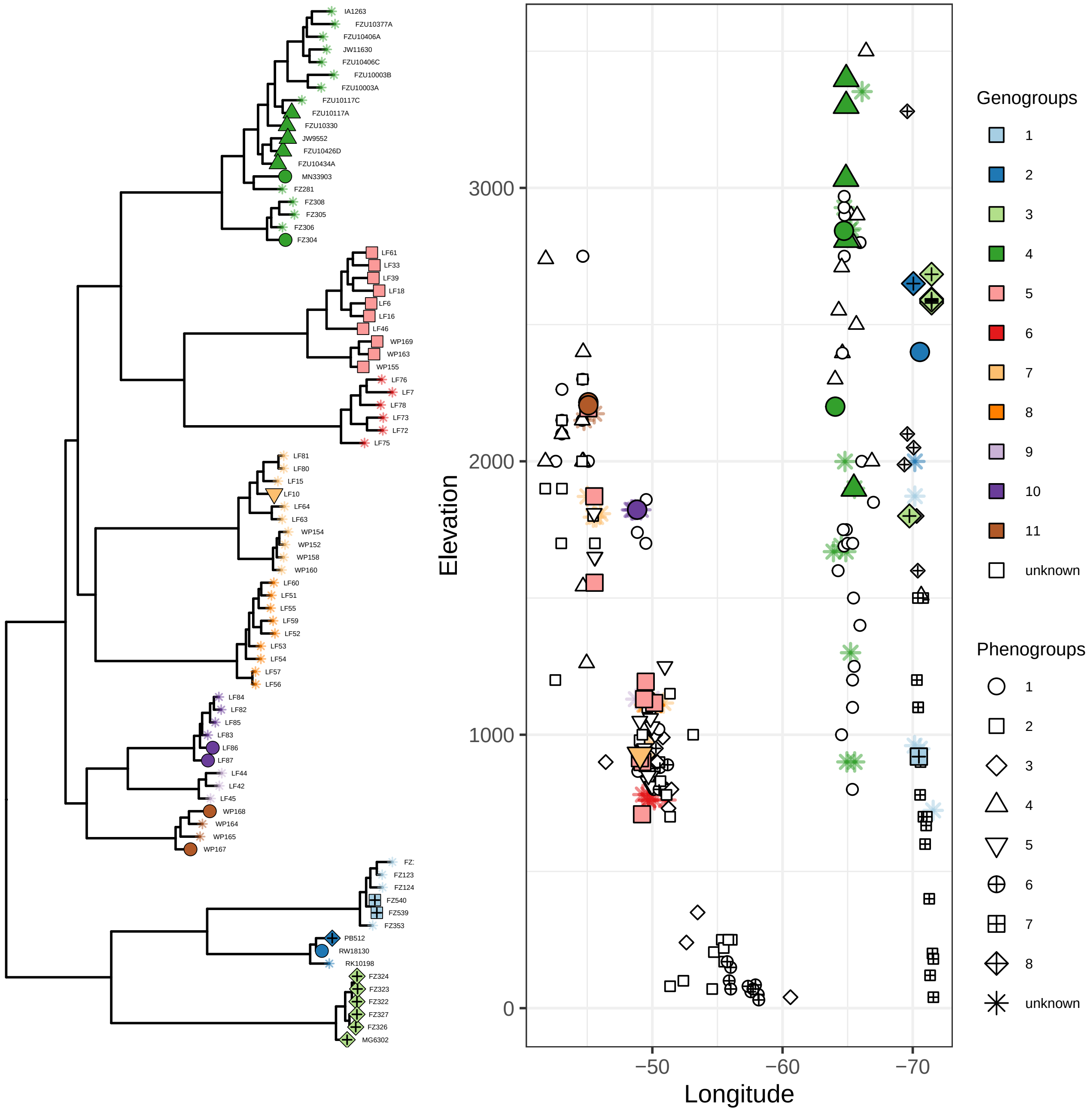

Figure S68: Integration of phenotypic and genomic data with spatial information and evolutionary history. All specimens are assigned to their corresponding best fit phenogroup (shapes) and genogroup (colors). Specimens without phenotypic or genomic data (unknown specimens) are shown as asterisks and empty shapes, accordingly. Specimens are shown as tips of the maximum likelihood tree (left) used in the CA model analysis and mapped along latitude and elevation (right). Specimens assigned to a single phenogroup and a single genogroup delineate species that we determined as 'good species'. Specimens assigned to a single phenogroup across multiple genogroups delineate species that we determined as 'phenotypic cryptic species'. Specimens assigned to a single genogroup across multiple phenogroups delineate species that we determined as 'genetic cryptic species'.

Return to Clade VI Data integration

5 References

1. Sleumer, H. O. Die Gattung Escallonia. *Verhandelingen der Koninklijke Nederlandse Akademie van Wetenschappen, Afd. Natuurkunde* 1–149 (1968).

2. Sede, S. M. & Denham, S. S. Taxonomic Revision of Escallonia (Escalloniaceae) in Argentina. *Systematic Botany* **43**, 364–396 (2018).

3. Doyle, J. J. & Doyle, J. L. *A rapid DNA isolation procedure for small quantities of fresh leaf tissue.* (1987).

4. Cullings, K. Design and testing of a plant-specific PCR primer for ecological and evolutionary studies. *Molecular ecology* **1**, 233–240 (1992).

5. Li, J., Yang, J., Chen, D., Zhang, X. & Tang, Z. An optimized mini-preparation method to obtain high-quality genomic DNA from mature leaves of sunflower. *Genet. Mol. Res* **6**, 1064–1071 (2007).

6. Eaton, D. A. & Overcast, I. Ipyrad: Interactive assembly and analysis of RADseq datasets. *Bioinformatics* **36**, 2592–2594 (2020).

7. Danecek, P. *et al.* The variant call format and VCFtools. *Bioinformatics* **27**, 2156–2158 (2011).

8. Fisher, R. A. XV.—the correlation between relatives on the supposition of mendelian inheritance. *Earth and Environmental Science Transactions of the Royal Society of Edinburgh* **52**, 399–433 (1919).

9. Templeton, A. R. *Population genetics and microevolutionary theory.* (John Wiley & Sons, 2006).

10. Frary, A., Doganlar, S., Daunay, M.-C. & Tanksley, S. D. QTL analysis of morphological traits in eggplant and implications for conservation of gene function during evolution of solanaceous species. *Theoretical and Applied Genetics* **107**, 359–370 (2003).

11. Frary, A., Fritz, L. A. & Tanksley, S. D. A comparative study of the genetic bases of natural variation in tomato leaf, sepal, and petal morphology. *Theoretical and Applied Genetics* **109**, 523–533 (2004).

12. Ferris, K. G. *et al.* Leaf shape evolution has a similar genetic architecture in three edaphic specialists within the mimulus guttatus species complex. *Annals of botany* **116**, 213–223 (2015).

13. Brock, M. T. *et al.* Floral genetic architecture: An examination of QTL architecture underlying floral (co) variation across environments. *Genetics* **186**, 1451–1465 (2010).

14. Abraham, M. C., Metheetrairut, C. & Irish, V. F. Natural variation identifies multiple loci controlling petal shape and size in arabidopsis thaliana. *PLoS One* **8**, e56743 (2013).

15. Zhang, Q. *et al.* The genetic architecture of floral traits in the woody plant prunus mume. *Nature communications* **9**, 1–12 (2018).

16. Futuyma, D. J. Evolution. Sunderland, MA. (2013).

17. Scrucca, L., Fop, M., Murphy, T. B. & Raftery, A. E. mclust 5: Clustering, Classification and Density Estimation Using Gaussian Finite Mixture Models. *The R Journal* 289–317 (2016).

18. Hoang, D. T., Chernomor, O., Von Haeseler, A., Minh, B. Q. & Vinh, L. S. UFBoot2: Improving the ultrafast bootstrap approximation. *Molecular Biology and Evolution* **35**, 518–522 (2018).

19. Minh, B. Q. *et al.* IQ-TREE 2: New models and efficient methods for phylogenetic inference in the genomic era. *Molecular Biology and Evolution* **37**, 1530–1534 (2020).

20. Patterson, N., Price, A. L. & Reich, D. Population structure and eigenanalysis. *PLoS genet* **2**, e190 (2006).

21. Zheng, X. *et al.* A high-performance computing toolset for relatedness and principal component analysis of SNP data. *Bioinformatics* **28**, 3326–3328 (2012).

22. Alexander, D. H., Novembre, J. & Lange, K. Fast model-based estimation of ancestry in unrelated individuals. *Genome Research* **19**, 1655–1664 (2009).

23. Pritchard, J. K., Stephens, M. & Donnelly, P. Inference of population structure using multilocus genotype data. *Genetics* **155**, 945–959 (2000).

24. Besnier, F. & Glover, K. A. ParallelStructure: AR package to distribute parallel runs of the population genetics program STRUCTURE on multi-core computers. *PLoS One* **8**, e70651 (2013).

25. Earl, D. A. & others. STRUCTURE HARVESTER: A website and program for visualizing STRUCTURE output and implementing the evanno method. *Conservation genetics resources* **4**, 359–361 (2012).

26. Evanno, G., Regnaut, S. & Goudet, J. Detecting the number of clusters of individuals using the software STRUCTURE: A simulation study. *Molecular ecology* **14**, 2611–2620 (2005).

27. Jakobsson, M. & Rosenberg, N. A. CLUMPP: A cluster matching and permutation program for dealing with label switching and multimodality in analysis of population structure. *Bioinformatics* **23**, 1801–1806 (2007).

28. Verity, R. & Nichols, R. A. Estimating the number of subpopulations (k) in structured populations. *Genetics* **203**, 1827–1839 (2016).

29. Yang, Z. & Rannala, B. Unguided species delimitation using DNA sequence data from multiple loci. *Molecular Biology and Evolution* **31**, 3125–3135 (2014).

30. Yang, Z., Rannala, B. & Edwards, S. V. Bayesian species delimitation using multilocus sequence data. *Proceedings of the National Academy of Sciences* **107**, 9264–9269 (2010).

32. Minh, B. Q., Hahn, M. W. & Lanfear, R. New methods to calculate concordance factors for phylogenomic datasets. *Molecular biology and evolution* **37**, 2727–2733 (2020).

32. Minh, B. Q., Hahn, M. W. & Lanfear, R. New methods to calculate concordance factors for phylogenomic datasets. *Molecular biology and evolution* **37**, 2727–2733 (2020).

33. Kalyaanamoorthy, S., Minh, B. Q., Wong, T. K., Haeseler, A. von & Jermiin, L. S. ModelFinder: Fast model selection for accurate phylogenetic estimates. *Nature Methods* **14**, 587–589 (2017).

35. Kapli, P. *et al.* Multi-rate poisson tree processes for single-locus species delimitation under maximum likelihood and markov chain monte carlo. *Bioinformatics* **33**, 1630–1638 (2017).

35. Kapli, P. *et al.* Multi-rate poisson tree processes for single-locus species delimitation under maximum likelihood and markov chain monte carlo. *Bioinformatics* **33**, 1630–1638 (2017).

36. Goddman, L. & Kruskal, W. Measures of association for cross classification. *Journal of the American Statistical Association* **49**, 732–764 (1954).

37. Pearson, R. *GoodmanKruskal: Association analysis for categorical variables.* (2016).

38. Kass, R. E. & Raftery, A. E. Bayes factors. *Journal of the american statistical association* **90**, 773–795 (1995).

39. Wiens, J. J. Speciation and ecology revisited: Phylogenetic niche conservatism and the origin of species. *Evolution* **58**, 193–197 (2004).

40. Lotsy, J. Species or linneon. *Genetica* **7**, 487–506 (1925).

41. Cronk, Q. C. & Suarez-Gonzalez, A. The role of interspecific hybridization in adaptive potential at range margins. *Molecular Ecology* **27**, 4653–4656 (2018).

42. Lewis, H. The nature of plant species. *Journal of the Arizona Academy of Science* **1**, 3–7 (1959).

43. Levin, D. A. The nature of plant species. *Science* **204**, 381–384 (1979).

44. Rieseberg, L. H., Wood, T. E. & Baack, E. J. The nature of plant species. *Nature* **440**, 524–527 (2006).

45. Mayr, E. A local flora and the biological species concept. *American Journal of Botany* **79**, 222–238 (1992).

46. Sneath, P. H. & Sokal, R. R. *Numerical taxonomy. The principles and practice of numerical classification.* (1973).

47. Carstens, B. C., Pelletier, T. A., Reid, N. M. & Satler, J. D. How to fail at species delimitation. *Molecular ecology* **22**, 4369–4383 (2013).

48. Barraclough, T. G. *The evolutionary biology of species.* (Oxford University Press, 2019).

49. Cadena, C. D., Zapata, F. & Jiménez, I. Issues and perspectives in species delimitation using phenotypic data: Atlantean evolution in darwin's finches. *Systematic Biology* **67**, 181–194 (2018).

50. Queiroz, K. de. The General Lineage Concept of Species, Species Criteria, and the Process. in *Endless forms: Species and speciation* (eds. Harrison, R. G. & Berlocher, S. H.) 57–75 (1998).

52. Cadena, C. D. & Zapata, F. The genomic revolution and species delimitation in birds (and other organisms): Why phenotypes should not be overlooked. *The Auk* **138**, ukaa069 (2021).

52. Cadena, C. D. & Zapata, F. The genomic revolution and species delimitation in birds (and other organisms): Why phenotypes should not be overlooked. *The Auk* **138**, ukaa069 (2021).

53. Mayr, E. *Populations, species, and evolution: An abridgment of animal species and evolution.* vol. 19 (Harvard University Press, 1970).

54. Levin, D. A. *The origin, expansion, and demise of plant species.* (Oxford University Press on Demand, 2000).

55. Zapata, F. A multilocus phylogenetic analysis of Escallonia (Escalloniaceae): diversification in montane South America. *American Journal of Botany* **100**, 526–545 (2013).

56. Sede, S. M., Dürnhöfer, S. I., Morello, S. & Zapata, F. Phylogenetics of Escallonia (Escalloniaceae) based on plastid DNA sequence data. *Botanical Journal of the Linnean Society* **173**, 442–451 (2013).

58. Mallet, J. Hybridization, ecological races and the nature of species: Empirical evidence for the ease of speciation. *Philosophical Transactions of the Royal Society B: Biological Sciences* **363**, 2971–2986 (2008).

58. Mallet, J. Hybridization, ecological races and the nature of species: Empirical evidence for the ease of speciation. *Philosophical Transactions of the Royal Society B: Biological Sciences* **363**, 2971–2986 (2008).

59. Valdivia, C. E. & Niemeyer, H. M. Do floral syndromes predict specialisation in plant pollination systems? Assessment of diurnal and nocturnal pollination of Escallonia myrtoidea. *New Zealand Journal of Botany* **44**, 135–141 (2006).

60. Zielinski, Q. B. Escallonia: the genus and its chromosomes. *Botanical Gazette* **117**, 166–172 (1955).

61. Sanders, R. W., Stuessy, T. F. & Rodriguez, R. Chromosome numbers from the flora of the juan fernandez islands. *American journal of botany* **70**, 799–810 (1983).

62. Hanson, L., Brown, R. L., Boyd, A., Johnson, M. A. & Bennett, M. D. First nuclear DNA c-values for 28 angiosperm genera. *Annals of Botany* **91**, 31–38 (2003).

63. Eastwood, A. The Escallonias in Golden Gate Park, San Francisco, California: With Descriptions of New Species. *California Academy of Sciences* **13**, 385–391 (1929).

64. Winston, J. E. *Describing species: Practical taxonomic procedure for biologists.* (Columbia University Press, 1999).

65. Bellman, R. Dynamic programming and stochastic control processes. *Information and control* **1**, 228–239 (1958).

66. Hastie, T., Tibshirani, R. & Friedman, J. *The elements of statistical learning: Data mining, inference, and prediction.* (Springer Science & Business Media, 2009).

67. Pineda, Y. M., Cortes, A. J., Madrinan, S. & Jimenez, I. The nature of espeletia species. *bioRxiv* (2020).

68. McDade, L. A. Species concepts and problems in practice: Insight from botanical monographs. *Systematic Botany* 606–622 (1995).

69. Stevens, P. F. Botanical systematics 1950-2000: Change, progress, or both? *Taxon* **49**, 635–659 (2000).

70. McLachlan, G. J. & Peel, D. *Finite mixture models.* (John Wiley & Sons, 2004).

71. Dobzhansky, T. *Genetics and the origin of species.* (Columbia university press, 1937).

72. Barraclough, T. G. & Humphreys, A. M. The evolutionary reality of species and higher taxa in plants: A survey of post-modern opinion and evidence. *New Phytologist* **207**, 291–296 (2015).

73. Coyne, J. A. & Orr, H. A. *Speciation.* vol. 37 (Sinauer Associates Sunderland, MA, 2004).

74. Fišer, C., Robinson, C. T. & Malard, F. Cryptic species as a window into the paradigm shift of the species concept. *Molecular Ecology* **27**, 613–635 (2018).

75. Mallet, J. A species definition for the modern synthesis. *Trends in Ecology & Evolution* **10**, 294–299 (1995).

76. Hausdorf, B. & Hennig, C. Species delimitation using dominant and codominant multilocus markers. *Systematic Biology* **59**, 491–503 (2010).

77. Baum, D. A. & Shaw, K. L. Genealogical perspectives on the species problem. in *Experimental and molecular approaches to plant biosystematics* (ed. Hoch, P. C.) 289–303 (Missouri Botanical Garden Press, 1995).

78. Yang, Z. & Rannala, B. Bayesian species delimitation using multilocus sequence data. *Proceedings of the National Academy of Sciences* **107**, 9264–9269 (2010).

79. Leaché, A. D., Fujita, M. K., Minin, V. N. & Bouckaert, R. R. Species delimitation using genome-wide SNP data. *Systematic biology* **63**, 534–542 (2014).

80. Mason, N. A., Fletcher, N. K., Gill, B. A., Funk, W. C. & Zamudio, K. R. Coalescent-based species delimitation is sensitive to geographic sampling and isolation by distance. *Systematics and Biodiversity* **18**, 269–280 (2020).

81. Baum, D. A. Individuality and the existence of species through time. *Systematic Biology* **47**, 641–653 (1998).

82. De Queiroz, K. Species concepts and species delimitation. *Systematic biology* **56**, 879–886 (2007).

83. Filatov, D. A., Osborne, O. G. & Papadopulos, A. S. Demographic history of speciation in a senecio altitudinal hybrid zone on mt. etna. *Molecular Ecology* **25**, 2467–2481 (2016).

84. Weir, J. T. & Price, T. D. Limits to speciation inferred from times to secondary sympatry and ages of hybridizing species along a latitudinal gradient. *The American Naturalist* **177**, 462–469 (2011).

85. Singhal, S. & Moritz, C. Reproductive isolation between phylogeographic lineages scales with divergence. *Proceedings of the Royal Society B: Biological Sciences* **280**, 20132246 (2013).

86. Struck, T. H. *et al.* Finding evolutionary processes hidden in cryptic species. *Trends in Ecology & Evolution* **33**, 153–163 (2018).

87. Novikova, P. Y. *et al.* Sequencing of the genus arabidopsis identifies a complex history of nonbifurcating speciation and abundant trans-specific polymorphism. *Nature Genetics* **48**, 1077–1082 (2016).

88. Cannon, C. H. & Petit, R. J. The oak syngameon: More than the sum of its parts. *New Phytologist* **226**, 978–983 (2020).

89. Wang, X., He, Z., Shi, S. & Wu, C.-I. Genes and speciation: Is it time to abandon the biological species concept? *National Science Review* **7**, 1387–1397 (2020).

90. Mallet, J., Besansky, N. & Hahn, M. W. How reticulated are species? *BioEssays* **38**, 140–149 (2016).

91. Hipp, A. L. *et al.* Genomic landscape of the global oak phylogeny. *New Phytologist* **226**, 1198–1212 (2020).

92. Barth, J. M. *et al.* Stable species boundaries despite ten million years of hybridization in tropical eels. *Nature Communications* **11**, 1–13 (2020).

93. Harrison, R. G. & Larson, E. L. Hybridization, introgression, and the nature of species boundaries. *Journal of Heredity* **105**, 795–809 (2014).

94. Rundell, R. J. & Price, T. D. Adaptive radiation, nonadaptive radiation, ecological speciation and nonecological speciation. *Trends in Ecology & Evolution* **24**, 394–399 (2009).

95. Nevado, B., Contreras-Ortiz, N., Hughes, C. & Filatov, D. A. Pleistocene glacial cycles drive isolation, gene flow and speciation in the high-elevation andes. *New Phytologist* **219**, 779–793 (2018).

96. Edelman, N. B. *et al.* Genomic architecture and introgression shape a butterfly radiation. *Science* **366**, 594–599 (2019).

A Appendix A: Manuscript in Spanish

Un análisis genómico y fenómico integrado para investigar la naturaleza de las especies vegetales en *Escallonia* (Escalloniaceae)

Sarah J. Jacobs[1,2], Michael C Grundler[1], Claudia L. Henriquez[1], Felipe Zapata[1]*

[1]Department of Ecology and Evolutionary Biology, University of California, Los Angeles, CA 90095

[2]Department of Botany, California Academy of Sciences, San Francisco CA 94118

* Autor para correspondencia: fzapata@ucla.edu

Resumen

Lo que entendemos por especies y si tienen alguna realidad biológica se ha debatido desde los primeros días de la biología evolutiva. Algunos biólogos incluso sugieren que las especies vegetales son creadas por los taxónomos como una división subjetiva y artificial de la naturaleza. Sin embargo, la naturaleza de las especies vegetales rara vez se ha puesto a prueba de forma crítica con datos, ignorando la taxonomía. En este estudio, nosotros integramos datos fenómicos y genómicos recogidos en cientos de individuos a escala continental para investigar esta pregunta en *Escallonia* (Escalloniaceae), un grupo de plantas que incluye 40 especies taxonómicas (las especies propuestas por los taxónomos). En primer lugar, mostramos que las especies taxonómicas pueden ser cuestionables, ya que se ajustan de manera limitada a los patrones de variación fenotípica y genética que muestran los individuos recogidos en la naturaleza. A continuación, utilizamos métodos estadísticos explícitos para la delimitación de las especies, diseñados para datos fenotípicos y genómicos, y mostramos que las especies vegetales existen en *Escallonia* como una propiedad objetiva y discreta de la naturaleza, independiente de la taxonomía. Demostramos que tales especies corresponden de manera limitada con las especies taxonómicas actuales ($< 20\%$) y que los datos fenómicos y genómicos rara vez delimitan entidades congruentes ($< 20\%$). Estas discrepancias sugieren que fuerzas evolutivas adicionales al flujo génico pueden mantener la cohesión de las especies. Proponemos que los datos fenómicos y genómicos analizados en igualdad de condiciones permiten una perspectiva más holística para entender la naturaleza de las especies vegetales al ayudar a delimitar diferentes "tipos de especies". Nuestros resultados alertan los estudios que dan por sentada la exactitud de las especies taxonómicas y ponen en tela de juicio la noción de especies vegetales sin pruebas empíricas.

Introducción

Una pregunta perenne en biología se refiere a la posibilidad de que las especies vegetales no sean reales, sino presumiblemente construcciones artificales de la psique de los taxónomos.[42–44] Los investigadores que han abordado esta pregunta mediante el análsis de datos fenotípicos se han centrado en validar las especies taxonómicas (es decir, las especies propuestas por los taxónomos).[44,45] Esto significa utilizar las especies taxonómicas como referentes estándar para determinar la fuerza de la evidencia empírica en apoyo de la realidad de las especies cuando los investigadores analizan los datos fenotípicos con métodos de taxonomía numérica para identificar las especies.[46] En un artículo muy influyente, Rieseberg et al.[44] recopilaron datos de 400 estudios que utilizaron métodos numéricos para identificar especies de plantas y animales con datos fenotípicos, y evaluaron hasta qué punto las especies delimitadas con métodos estadísticos coincidían con las especies taxonómicas. Este estudio reveló que la validación de las especies taxonómicas es baja ($< 60\%$ de los grupos discretos identificados estadísticamente son congruentes con las especies taxonómicas) aunque aparentemente existen grupos fenotípicos discretos en la mayoría de los grupos taxonómicos.[44] Sin embargo, al utilizar un enfoque de validación de especies, en lugar de un enfoque de descubrimiento de especies,[47,48] este estudio asumió que las especies taxonómicas existen como tal. Desgraciadamente, Rieseberg et al.[44] no tuvieron acceso a enfoques estadísticos útiles para evaluar la realidad de las especies independientemente de la taxonomía ni a datos de secuencias multilocus como

línea de evidencia adicional para investigar la naturaleza de las especies entre taxones. En consecuencia, la pregunta fundamental sobre la realidad de las especies vegetales independiente de la influencia de los taxónomos sigue sin entenderse de manera clara. Hasta la fecha, ningún estudio que integre datos fenotípicos y de ADN de todo el genoma ha evaluado la realidad de las especies de plantas para un grupo que incluye múltiples especies taxonómicas hipotéticas a una amplia escala geográfica. En este estudio, nosotros investigamos esta pregunta a través de análisis de delimitación de especies utilizando datos fenotípicos (aprox. 8.300 medidas cuantitativas) y genómicos (aprox. 1.000.000 de secuencias de ADN) en un conjunto de 848 individuos de *Escallonia* (Escalloniaceae), un grupo de arbustos y árboles que abarca la región montañosa de Sudamérica (Fig. 1; Tabla Suplementaria S1).

Muchos estudios que incorporan el procedimiento de delimitación de especies presentan varias deficiencias relevantes para comprender la naturaleza de las especies vegetales. En primer lugar, la mayoría de los estudios que utilizan datos fenotípicos se basan en enfoques estadísticos desconectados de la teoría biológica y, por lo tanto, se ven comprometidos en la detección de especies biológicamente significativas.[49] En particular, estos estudios suelen utilizar métodos que se basan en análisis gráficos visuales que transmiten poca información sobre las frecuencias de los fenotipos, excluyen rasgos fenotípicos potencialmente importantes para la detección de especies y utilizan medidas de tendencia central que son intrascendentes para evaluar el carácter distintivo de las especies.[49] En segundo lugar, muchos estudios utilizan procedimientos numéricos explícitos para analizar los datos fenotípicos solo cuando analizan "taxones problemáticos" (es decir, complejos de especies, enjambres de híbridos), y por lo tanto pueden proporcionar una perspectiva general distorsionada sobre la naturaleza de las especies vegetales. En tercer lugar, algunos estudios no investigan directamente la naturaleza de las especies vegetales utilizando datos genéticos los cuales tienen una relación explícita con la divergencia evolutiva y el flujo génico, dos criterios relevantes en la delimitación de las especies.[50] Alternativamente, otros estudios se basan exclusivamente en datos genéticos, lo que puede llevar a que no se descubran especies que mantienen la cohesión y la independencia a través de fuerzas evolutivas adicionales al flujo génico.[52] Por último, varios estudios no consideran la delimitación de las especies en un contexto geográfico a pesar del papel central de la geografía en el estudio de la naturaleza de las especies.[53,54] En este estudio, nosotros abordamos estas limitaciones en el examen de la naturaleza de las especies vegetales mediante la integración de múltiples tipos de datos y utilizando enfoques estadísticos adecuados bien fundamentados en la teoría evolutiva, en *Escallonia*, un género típico de plantas con flores, aparentemente compuesto por especies taxonómicas "buenas".[1]

Los árboles y arbustos del género *Escallonia* constituyen un excelente caso de estudio para llevar a cabo este tipo de análisis para investigar la naturaleza de las especies vegetales. Estas plantas se encuentran en una variedad de hábitats a lo largo de los Andes y las montañas del sureste de Brasil, así como en cordilleras aisladas como la Sierra de Córdoba (Argentina), la Sierra Nevada de Santa Marta (Colombia) y la Cordillera de Talamanca (Costa Rica).[55,56] La mayoría de las especies taxonómicas en *Escallonia* tienen rangos geográficos amplios, con algunas especies que tienen poblaciones separadas por miles de kilómetros; unas pocas especies son de distribución geográfica limitada que abarca menos de 200 kilómetros. Algunas especies taxonómicas son comunes localmente, con aproximadamente 30-40 plantas por localidad, mientras que otras son raras, encontrándose pocos individuos en cualquier lugar (F. Zapata, obs. pers.). Varias especies taxonómicas parecen segregarse según el hábitat o la elevación, sin embargo los rangos geográficos de muchas especies se superponen total o parcialmente, de tal manera que los individuos de una especie taxonómica pueden ocurrir dentro del rango de dispersión potencial de gametos (semillas o polen) de otras especies taxonómicas (es decir, las especies taxonómicas exhiben simpatría en mosaico *sensu*).[58]

En todas las especies taxonómicas, el fruto es una cápsula seca que se abre y libera las semillas, las cuales caen y son probablemente dispersadas por el viento o la gravedad. Se conoce muy poco acerca de la biología de la polinización de cualquier especie taxonómica,[59] y a partir de observaciones circunstanciales en el campo, las flores de diferentes especies taxonómicas de *Escallonia* parecen ser visitadas por un grupo diverso de insectos locales que también visitan otras plantas de géneros no relacionados. Es imperativo

realizar estudios que cuantifiquen las barreras de aislamiento reproductivo en *Escallonia* para entender el papel de las señales florales en la especiación. Morfológicamente, las especies taxonómicas de *Escallonia* muestran una variación sustancial en el tamaño y la forma general de las hojas, probablemente asociada a las condiciones ecológicas y a los cambios de hábitat (F. Zapata, sin publicar). Las especies taxonómicas pueden tener flores individuales o inflorescencias con decenas o cientos de flores. Las flores muestran una considerable variación geográfica en el tamaño y la forma de los sépalos, pétalos y ovarios. El color de los pétalos varía de blanco verdoso a rosa o rojo intenso. La morfología y el número de cromosomas ($n = 12$) son los mismos para todas las especies taxonómicas examinadas hasta el momento,[60–62] y los horticultores han generado híbridos artificiales entre especies morfológicamente distintas que no crecen juntas en la naturaleza (por ejemplo,).[63] Sin embargo, no hay casos documentados de especiación híbrida o zonas híbridas estables en la naturaleza.

Por lo tanto, *Escallonia* parece ser un género "típico" de plantas con flores que no se considera único o problemático desde el punto de vista taxonómico. Desde el punto de vista genético, no existen estudios que utilicen datos genómicos que incluyan varios individuos por especie taxonómica para todas las especies del área de distribución geográfica de *Escallonia* (es decir, no se conoce el estado de las especies taxonómicas desde una perspectiva multilocus). Sin embargo, es útil reiterar que no hay hibridación o introgresión natural rampante documentada, no hay casos conocidos de poliploidía y, hasta donde sabemos, no hay agamospermia ni apomixis en este género. Desde una perspectiva morfológica, las especies taxonómicas parecen estar más o menos bien definidas; existe cierta variación, pero el género no es notable o inusual en este sentido. En conjunto, *Escallonia* ofrece una gran oportunidad para estudiar en detalle los patrones geográficos de variación de los rasgos fenotípicos y la genómica para examinar la naturaleza de las especies vegetales.

Dilucidar la naturaleza de las especies vegetales tiene implicaciones más amplias que la taxonomía. En particular, determinar si las especies existen como propiedades objetivas de la naturaleza puede tener repercusiones en otras áreas de la biología que utilizan las especies como unidad de análisis. Además, la comparación de los patrones geográficos de variación en los datos fenotípicos y genéticos puede empezar a arrojar luz sobre el papel de las fuerzas evolutivas que intervienen en el origen, la evolución y la estructura de la biodiversidad.

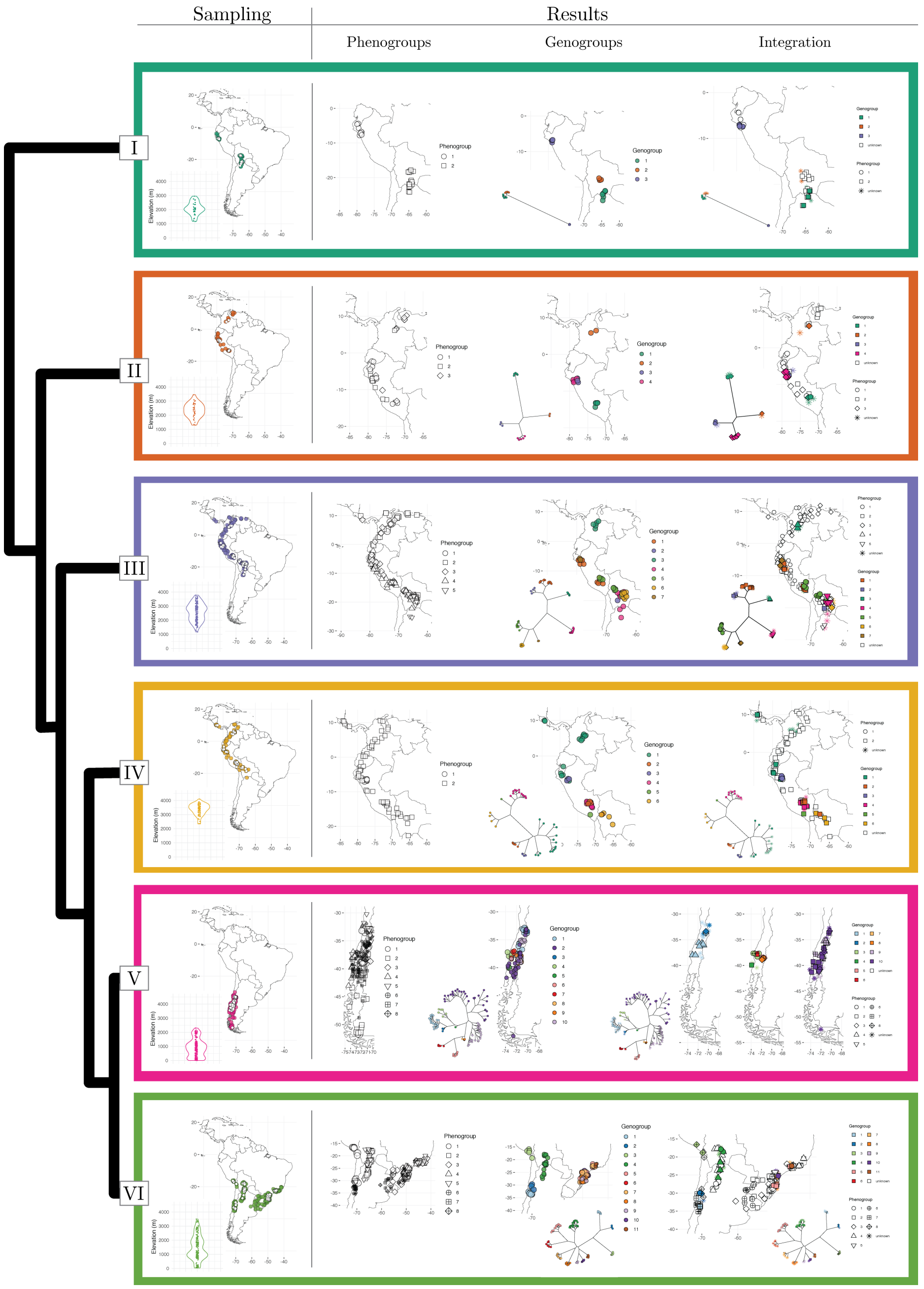

Figure 1: Historia filogenetica, muestreo y delimitacion de especies basada en modelos evolutivos. Arbol de maxima verosimilitud (ML) de Escallonia basado en datos de todo el genoma (izquierda) indicando los seis clados focales (Clade I-VI) de nuestro estudio. Para cada clado, la primera columna de la izquierda muestra el muestreo, con simbolos rellenos que indican los especimenes utilizados en los analisis fenotipicos y simbolos vacios los especimenes utilizados en los analisis genomicos; los recuadros muestran la distribucion de los especimenes a lo largo de la elevacion. La segunda columna a la derecha muestra los resultados del mejor modelo para la delimitacion de especies con datos fenotipicos (aca llamados fenogrupos); los fenogrupos se muestran con simbolos de diferentes formas en el espacio geografico. La tercera columna muestra los resultados del mejor modelo para la delimitacion de especies con datos genomicos (aca llamados genogrupos); los genogrupos se indican con simbolos de diferentes colores y como apice de los arboles ML no enraizados basados en matrices de loci concatenados y mapeados en el espacio geografico. La cuarta columna muestra la integracion de los fenogrupos y genogrupos con la historia evolutiva y la distribucion geografica para dilucidar la naturaleza de las especies vegetales; los especimenes sin datos fenotipicos y genomicos estan designados como especimenes desconocidos.

Resultados y Discusión

A continuación presentamos y discutimos nuestros resultados de manera global en el contexto de toda la radiación de *Escallonia*. Los resultados detallados se pueden encontrar en el material suplementario (en idioma inglés).

El estado actual de las especies taxonómicas

Primero caracterizamos la historia evolutiva de *Escallonia* utilizando diferentes enfoques filogenéticos con un subconjunto de especímenes que abarcan el rango geográfico de estas plantas a lo largo de Sudamérica (Fig. 1; Fig. Suplementaria S1, S2). En todos estos análisis, recuperamos consistentemente seis grupos de especies taxonómicas (en adelante, clados I-VI), en línea con un estudio previo basado en menos loci.[55] Todos los clados están marcadamente restringidos a regiones geográficas, excepto el clado VI; este clado está principalmente restringido al sureste de Brasil, Uruguay y el noreste de Argentina, pero incluye algunas especies en los Andes (Fig. 1). Un examen más detallado de la relación entre la composición de los clados y las distribuciones geográficas y elevaciones dentro de los clados revela que cuando los especímenes de diferentes clados co-ocurren en estrecha proximidad espacial (por ejemplo, los clados I, II, III, IV en los Andes Tropicales), los clados son genéticamente distintos sin mezclarse (Fig. 1; Fig. Suplementaria S1, S2). Además, todos los clados tienen una composición consistente y reciben un fuerte apoyo estadístico cuando utilizamos diferentes enfoques para el análisis filogenético (Ver Métodos). Sin embargo, cuando incluimos múltiples especímenes de la misma especie taxonómica, varios de estos especímenes no son siempre los parientes más cercanos entre sí dentro (es decir, las especies taxonómicas son parafiléticas o polifiléticas; Fig. Suplementaria S2). Este resultado, junto con la marcada concordancia geográfica filogenética y la composición consistente de los clados, sugiere que aunque los clados son evolutivamente distintos, los límites de las especies dentro de los clados se beneficiarían de un análisis más minucioso[55]. Por lo tanto, centramos nuestros análisis subsequentes de la variación fenotípica y genomica para investigar la naturaleza de las especies en *Escallonia* clado por clado.

Para investigar el estado actual de las especies taxonómicas en *Escallonia* a través de los datos fenotípicos, primero nos preguntamos si las especies taxonómicas son cuantitativamente distintas y luego nos preguntamos si los especímenes que se supone pertenecen a una especie taxonómica ocupan el morfoespacio delimitado por la combinación de rasgos que definen cada especie taxonómica. Para estos análisis, utilizamos los rasgos morfológicos -de hojas y flores- proporcionados en la descripción taxonómica de cada especie.[1] Nos centramos en estos rasgos porque las descripciones taxonómicas incluyen los caracteres útiles para distinguir todas las especies y para compararlas con otras especies.[64] Reconocemos que al centrarnos sólo en estos rasgos, podemos estar excluyendo rasgos relacionados con las diferencias funcionales de las especies (por ejemplo, rasgos funcionales de las plantas). Sin embargo, los rasgos utilizados en las descripciones taxonómicas proporcionan un punto de partida lógico para evaluar la naturaleza de las especies. Es a lo largo de tales dimensiones del fenotipo donde los taxónomos han hipotetizado previamente discontinuidades naturales y muchos de estos rasgos (ciertamente los rasgos florales) tienen relevancia biológica con respecto a la función reproductiva. Además, nuestro examen de aproximadamente 3.500 especímenes de herbario y el extenso trabajo de campo confirman una variación sustancial en los rasgos foliares y florales entre las especies taxonómicas.

Primero tabulamos los valores máximos y mínimos de diez rasgos cuantitativos continuos proporcionados en la descripción de cada especie (estos valores se derivan de especímenes no incluidos en el conjunto de datos actual). A continuación, utilizamos estos valores como vértices de un cubo de 10 lados para representar geométricamente cada especie en el espacio fenotípico y estimamos el solapamiento por pares entre todos los cubos de 10 lados dentro de cada clado. Este análisis muestra que las especies taxonómicas dentro de los clados ocupan regiones distintas del espacio fenotípico de 10 dimensiones con poco o ningún solapamiento (Tabla 1, Fig. Suplementaria S5, S16, S27, S38, S49, S60). Seguimos estos análisis basados en la geometría con un análisis de "predicción de coincidencia" en el que evaluamos si

cada espécimen identificado a una especie taxonómica estaba dentro o fuera del cubo de 10 lados de su correspondiente especie basado en medidas cuantitativas de los mismo rasgos morfológicos que definen el cubo de 10 lados (Ver Métodos). En contra de lo esperado, estos análisis muestran que la mayoría (99, 2%) de los especímenes se encuentran fuera de su respectivo cubo de 10 lados. Además, el 98, 4% de los especímenes quedan fuera de cualquier cubo de 10 lados (Tabla 1, Fig. Suplementaria S5, S16, S27, S38, S49, S60). Esto significa que la mayoría de los especímenes tienen al menos una medida que cae fuera del rango de variación proporcionado en sus descripciones taxonómicas. El uso de rangos fijos para los valores de los rasgos en las descripciones de las especies implica que las especies corresponden a formas geométricas con límites definidos (por ejemplo, cubos de 10 lados). Dadas las propiedades estadísticas y matemáticas de los espacios de alta dimensionalidad, una vez que un espécimen supera el límite impuesto por incluso una dimensión del cubo de 10 lados correspondiente a su especie taxonómica, dicho espécimen cae inmediatamente fuera del cubo de 10 lados por completo (por ejemplo, la maldición de la dimensionalidad).[65,66] Debido a que la mayoría de los especímenes examinados aquí caen fuera de su respectivo cubo de 10 lados, sugerimos que las especies taxonómicas en *Escallonia* pueden tener un poder limitado para capturar los patrones multidimensionales de variación fenotípica mostrados por los organismos en la naturaleza.

Es poco probable que este resultado sea un artefacto de la monografía taxonómica[1] porque las descripciones originales de las especies citan un gran número de especímenes examinados que cubren el rango geográfico conocido de todas las especies. Los especímenes incluidos en nuestro análisis se recogieron en las mismas localidades donde se recogieron los especímenes citados en la monografía; incluso medimos algunos de los especímenes de herbario citados en las descripciones originales de las especies. Nuestros resultados ponen de manifiesto la necesidad de incluir datos a nivel de espécimen (y no solo de especie) en las descripciones taxonómicas y monografías en el futuro, y de utilizar enfoques probabilísticos que incorporen la varianza y la covarianza entre los rasgos para definir las especies con el fin de capturar la forma de las especies en la naturaleza. Aunque nuestros resultados se limitan a *Escallonia*, especulamos que esto puede ser un fenómeno generalizado en otros grupos[67] porque las especies de plantas delimitadas y descritas con la morfología rara vez se basan en análisis estadísticos explícitos de la variación fenotípica basados en la teoría biológica.[68,69] Por lo tanto, sugerimos que investigar la naturaleza de las especies vegetales basándose únicamente en la validación de las especies taxonómicas puede ser generalmente problemático.

Table 1: **El estado actual de las especies taxonomicas**

Clade	Taxonomic species	Specimens	Minimum proportion overlap among 10-cubes	Maximum proportion overlap among 10-cubes	Percent specimens matching any 10-cube	Percent specimens matching correct 10-cube
I	2	33	0	0.00	0.0	0.0
II	2	33	0	0.00	0.0	0.0
III	6	130	0	0.02	1.6	0.8
IV	2	74	0	0.00	0.0	0.0
V	7	214	0	0.13	0.0	0.0
VI	10	195	0	0.00	0.0	0.0

Pruebas basadas en modelos evolutivos para identificar especies como entidades objetivas

Utilizamos modelos de mezclas finitas Gaussianas (GFMM)[70] dentro de los clados para determinar tanto el número de especies como la asignación de especímenes a las especies utilizando datos fenotípicos sin información previa sobre la taxonomía. Este marco analítico es adecuado para este problema porque implementa el modelo evolutivo que subyace al uso de la variación fenotípica cuantitativa y continua en el descubrimiento y delimitación de especies.[8,49] Para realizar este análisis, utilizamos los mismos especímenes y los mismos diez rasgos morfológicos diagnósticos que en nuestro análisis anterior (véase más arriba). Primero rotamos la matriz de datos original en ejes ortogonales utilizando estimadores de covarianza

robustos y redujimos la dimensionalidad de los ejes ortogonales a sólo aquellos que optimizaban la forma, la orientación y el número de especies basadas en el fenotipo (en adelante, fenogrupos). Identificamos el mejor modelo de mezclas Gaussianas - GMM (modelo ingenuo) en cada clado en un marco de criterio de información bayesiano (BIC) y de probabilidad de datos completos integrados (ICL). Además, evaluamos el apoyo a modelos alternativos en los que asignamos especímenes a grupos definidos *a priori*, incluyendo especies taxonómicas (modelo Taxonomía), así como fenogrupos que definimos a ojo durante el examen de los especímenes y que eran independientes de la taxonomía (modelo Inconsciente de la Taxonomía). Los resultados de estos análisis se muestran en la Fig. 1 y en la Tabla 2. El modelo ingenuo fue el mejor apoyado para cinco de los seis clados (ΔBIC$>$ 8), mientras que un clado tuvo apoyo (ΔBIC$<$ 1) aunque el modelo no fue el mejor apoyado para este clado (Fig. Suplementaria S39). Estos resultados fueron insensibles al enfoque de selección del modelo (BIC o ICL) (véase el material suplementario). El fuerte rendimiento del modelo ingenuo no es inesperado debido a las severas limitaciones de los otros modelos no estadísticos para porponer límites de las especies sin considerar la forma, la orientación y el solapamiento arbitrario de los grupos en el espacio fenotípico multidimensional[49] (Fig. Suplementaria S6, S17, S28, S39, S50, S61). Esto también es coherente con la predicción de que la naturaleza es, de hecho, discontinua[71,72] a pesar de las sugerencias de que las especies no son entidades objetivas discretas.[43] Además, debido a que la mayoría de los fenogrupos identificados dentro de los clados coocurren localmente en simpatría (Fig. 1; Fig. Suplementaria S6, S17, S28, S39, S50, S61), el estatus de las especies para estos grupos es aceptado bajo una amplia gama de definiciones de especie.[49,50,58,73] Sin embargo, los fenogrupos pueden ocultar especies distintas cuando fenotipos similares han evolucionado (o están evolucionando) de forma independiente.[74] Por lo tanto, la incorporación de información filogenética es beneficiosa para comprender la naturaleza de las especies y decidir si todos los fenogrupos corresponden a especies distintas.

Table 2: **Modelos de mezclas finitas gaussianas (GFMM) para delimitacion de fenogrupos y seleccion de modelos empleando el criterio de informacion bayesiano (BIC)**

Clade	Model	Phenogroups	BIC	Rank	ΔBIC
I	Naive	2	54.03099	1	0.00000
	Taxonomy	2	45.80586	2	8.22513
	Taxonomy unaware	1	33.45654	3	20.57445
II	Naive	3	71.72976	1	0.00000
	Taxonomy unaware	1	47.52785	2	24.20191
	Taxonomy	2	17.77346	3	53.95630
III	Naive	5	387.15280	1	0.00000
	Taxonomy unaware	4	170.83930	2	216.31350
	Taxonomy	6	53.38527	3	333.76753
IV	Taxonomy	2	-115.00390	1	0.00000
	Taxonomy unaware	2	-115.00390	1	0.00000
	Naive	3	-115.89910	2	0.89520
V	Naive	8	-516.72340	1	0.00000
	Taxonomy unaware	4	-648.03900	2	131.31560
	Taxonomy	7	-791.45350	3	274.73010
VI	Naive	8	231.24780	1	0.00000
	Taxonomy unaware	10	200.30380	2	30.94400
	Taxonomy	10	-517.76350	3	749.01130

Para identificar especies y asignar especímenes a especies dentro de clados utilizando datos genéticos, evaluamos el ajuste de tres modelos comunes de delimitación de especies. Estos modelos implementan tres definiciones diferentes de especies, a saber, especies definidas como grupos genotípicos[75,76] (modelo GC), especies definidas como el punto de transición de la cladogénesis a la anagénesis[35,77] (modelo

CA), y especies definidas como linajes reproductivamente aislados[53,78] (modelo RI). Aclaramos que estas definiciones de especies no están vinculadas a ningún mecanismo de especiación concreto. Por ejemplo, bajo diferentes mecanismos de especiación ecológica o geográfica, las especies podrían diagnosticarse como la transición de la cladogénesis a la anagénesis, o como grupos genéticos aislados. Nuestro análisis no es una inferencia del proceso de especiación en sí mismo. Más bien, nuestro estudio es una búsqueda de patrones (es decir, especies), que luego interpretamos a la luz de escenarios de especiación plausibles (véase la sección siguiente). Para este análisis, recopilamos datos de todo el genoma para un subconjunto de los especímenes utilizados en nuestros análisis fenotípicos y comparamos modelos de delimitación de especies que compiten entre sí en un marco bayesiano utilizando factores de Bayes[79] para identificar especies basadas en variación genética (en adelante, genogrupos). Dado que no se han propuesto especies taxonómicas ni otros grupos *a priori* basados en datos genéticos, no evaluamos el apoyo a ningún otro modelo alternativo de delimitación de especies. La Fig. 1 y la Tabla 3 muestran los resultados de estos análisis. En general, el modelo CA superó a los modelos alternativos; en cinco de los seis clados, el modelo CA fue el modelo mejor soportado, mientras que el modelo GC se ajustó mejor sólo para un clado. Además, el modelo CA es adecuado para capturar las especies que acá identificamos dado los resultados de un análsis de adecuación (Tabla S2). En todos los clados, el modelo que mejor se ajustaba identificaba el mayor número de genogrupos. La razón por la que los modelos con más genogrupos se ajustan mejor en todos los clados es probablemente el resultado de la mayor variación genética entre genogrupos que dentro de los mismos, lo cual es evidente como largas ramas en los árboles filogenéticos de especies (Fig. 1). Esto sugiere que los genogrupos son linajes divergentes en trayectorias evolutivas separadas, y es consistente con la hipótesis de que tales linajes son especies distintas.[48,50] Además, varios de estos genogrupos dentro de los clados coexisten localmente en simpatría y, por lo tanto, el estatus de especie para tales grupos se puede aceptar bajo múltiples definiciones de especie.[53,58,73] Sin embargo, en algunos clados los genogrupos forman grupos aislados y alopátricos de especímenes, lo que presumiblemente podría ser el resultado de un muestreo geográfico escaso dentro de una sola especie.[80] Por lo tanto, el peso de la evidencia en apoyo del estatus de especie para estos genogrupos es débil y requiere considerar otras líneas de evidencia en igualdad de condiciones.

Table 3: **Modelos genomicos para la delimitacion de genogrupos y seleccion de modelos empleado factores de Bayes (BF)**

Clade	Model	Genogroups	Marginal Likelihood (log_e)	Rank	BF (2 x log_e)
I	GC	3	-6580.495	1	
	AC	2	-6754.495	2	348.000
	RI	2	-6754.495	2	348.000
II	AC	4	-13460.917	1	
	GC	3	-15036.438	2	3151.042
	RI[a]	3	-15036.438	2	3151.042
	RI[b]	2	-18963.342	3	11004.850
III	AC	7	-8985.782	1	
	RI[a]	5	-10014.260	2	2056.955
	RI[b]	3	-12233.131	3	6494.698
	GC	3	-12233.131	3	6494.698
IV	AC	6	-9601.514	1	
	GC	3	-11546.649	2	3890.271
	RI[a]	2	-12017.878	3	4832.728
	RI[b]	2	-12017.878	3	4832.728
V	AC	10	-4588.693	1	
	GC	6	-5381.361	2	1585.336
	RI[a]	3	-5601.058	3	2024.730
	RI[b]	2	-6085.998	4	2994.610
VI	AC	11	-2921.024	1	
	GC	7	-3627.806	2	1413.564
	RI[a]	4	-4661.351	3	3480.654
	RI[b]	4	-4661.351	3	3480.654

[a] specimens assigned to demes using MAVERICK
[b] specimens assigned to demes using STRUCTURE

Integrando la variación fenotípica y genómica, con información espacial e historia evolutiva.

Con los fenogrupos y genogrupos derivados de los análisis basados en los modelos evolutivos, pudimos examinar la naturaleza de las especies integrando los datos fenotípicos y genómicos en un contexto espacial y evolutivo explícito (Fig. 1; Fig. Suplementaria S13, S24, S35, S46, S57, S68). Para este análisis, primero asignamos cada espécimen a su correspondiente fenogrupo y genogrupo, similar a una tabla de contingencia de dos vías (Fig. 2). Esta asignación permitió identificar la congruencia -o la falta de ella- entre los grupos fenotípicos y genómicos. Algunos especímenes estaban incompletos (por ejemplo, estériles) y no pudieron ser evaluados para todos los rasgos fenotípicos, mientras que otros especímenes fallaron durante el procesamiento para el trabajo genómico (en adelante, especímenes desconocidos); no obstante, la distribución geográfica de estos especímenes desconocidos en relación con los especímenes con ambos tipos de datos puede informar de la asignación más parsimoniosa a fenogrupos o genogrupos (por ejemplo, en el clado IV todos los especímenes desconocidos del norte de Sudamérica probablemente pertenecen al fenogrupo 2 y al genogrupo 1; Fig. 1, panel 2). En general, encontramos que sólo un pequeño porcentaje de fenogrupos corresponden directamente con genogrupos únicos (15%), incluso asumiendo una asignación de grupo concordante para todos los especímenes desconocidos (18%). Por el contrario, encontramos que en la mayoría de los clados un determinado fenogrupo ocurre a través de múltiples genogrupos (por ejemplo, ver el fenogrupo 2 en el clado IV, Fig. 2), y con menos frecuencia que un determinado genogrupo ocurre a través de diferentes fenogrupos (por ejemplo, ver el genogrupo 9 en el clado V, Fig. 2). En conjunto, nuestros resultados sugieren que la proporción de "especies buenas" (es decir, grupos fenotípicos y genómicos distintos y congruentes) en *Escallonia* es notablemente baja, especialmente teniendo en cuenta la noción generalizada en biología de que las "especies buenas" son la norma, y sugieren que otros tipos

de especies, incluidas las “especies fenotípicas crípticas”[74] (es decir, un fenogrupo a través de múltiples genogrupos) y “especies crípticas genéticas”[52] (es decir, un genogrupo a través de múltiples fenogrupos), son más comunes. La existencia de estos diferentes tipos de especies es coherente con la idea de que las propiedades de las especies, como la distinguibilidad morfológica o la exclusividad genealógica de los alelos, pueden evolucionar en diferentes momentos y orden secuencial debido a la naturaleza heterogénea del proceso de especiación.[81,82]

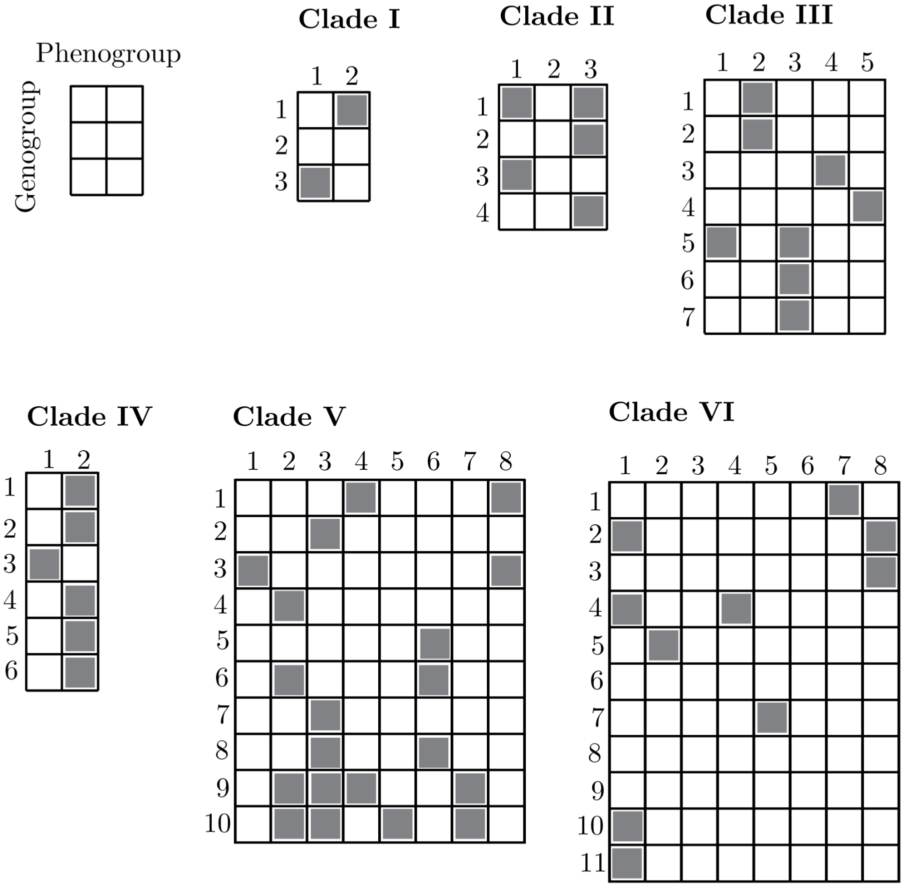

Figure 2: Integracion de la variacion fenotipica y genomica para delimitar especies. Para cada clado (vease la Fig. 1), asignamos especimenes a su correspondiente fenogrupo y genogrupo basandonos en los modelos de mejor ajuste para cada tipo de datos. Las celdas sombreadas muestran los especimenes asignados a una combinacion particular de fenogrupo y genogrupo de mejor ajuste (es decir, cada celda sombreada es una especie). Se reconocen tres tipos de especies. En primer lugar, los especimenes asignados unicamente a un solo fenogrupo y a un solo genogrupo se reconocen como ’especies buenas’ (por ejemplo, fenogrupo 4, genogrupo 3 en el clado III). En segundo lugar, los especimenes asignados a un unico fenogrupo a traves de multiples genogrupos se reconocen como ’especies fenotipicas cripticas’ (por ejemplo, fenogrupo 2, genogrupos 1, 2 en el clado III). En tercer lugar, los especimenes asignados a un unico genogrupo a traves de multiples fenogrupos se reconocen como ’especies geneticas cripticas’ (por ejemplo, fenogrupos 1, 3, genogrupo 5, en el Clado III). Las filas o columnas vacias corresponden a especimenes que no tenian datos fenotipicos y genomicos sobrepuestos y, por lo tanto, se asignaron solo a su correspondiente fenogrupo o genogrupo, segun corresponda (por ejemplo, el genogrupo 2 en el Clado I).

La interpretación de las especies que identificamos en un contexto espacial y filogenético explícito puede dilucidar aún más la naturaleza de las especies vegetales. Nuestra motivación es ofrecer una interpretación del tipo de especies que descubrimos (patrón) a la luz de los mecanismos de especiación plausibles (proceso). No obstante, señalamos que es fundamental seguir trabajando con un muestreo más denso y con enfoques analíticos específicos para inferir el proceso de especiación real. La mayoría de las "especies buenas" se encuentran en simpatría local o se segregan según la elevación con otras especies (Fig. 1, Fig. 2; Fig. Suplementaria S13, S24, S35, S46, S57, S68). Esto sugiere que la selección mediada por el medio ambiente en simpatría o a lo largo de gradientes de elevación en parapatría puede ser una importante fuerza evolutiva que impulsa la especiación[83] o al menos mantiene las diferencias entre especies en *Escallonia.* Aunque estas especies pueden diferir en reasgos florales y foliares, se necesitan estudios sobre la biología reproductiva y el papel de otros factores bióticos y abióticos para desentrañar cómo se originan y se mantienen las "buenas especies" en *Escallonia* en la naturaleza. Alternativamente, es posible que estas especies simplemente estén más adelantadas en el proceso de especiación y hayan acumulado suficientes diferencias.[84,85] Se necesitan más muestreos en combinación con enfoques de datación filogenética y datos experimentales en *Escallonia* para evaluar estas hipótesis con mayor rigor.

Cuando los genogrupos de las "especies fenotípicas crípticas" están relacionados de forma distante, una hipótesis razonable para explicar este patrón es la idea de una evolución convergente en los fenotipos en respuesta a regímenes selectivos similares, ya sea en simpatría o alopatría[86] (por ejemplo, véase el fenogrupo 1, los genogrupos $2, 4, 10, 11$, el clado VI; Fig. 1). *Escallonia* ocurre en hábitats de montaña que muestran condiciones ambientales similares a través de regiones geográficas separadas (por ejemplo, las montañas del sureste de Brasil, el sur de los Andes y los Andes Tropicales de alta elevación).[55] La posibilidad de una evolución replicada de especies con rasgos foliares y florales similares a través de regiones geográficas separadas como un archipiélago de montaña es intrigante y debe ser investigada en detalle. Por el contrario, cuando estos genogrupos son los parientes más cercanos entre sí y no coocurren localmente en simpatría (por ejemplo, véase el fenogrupo 2, genogrupos $1, 2$, clado III; Fig. 1), bajo algunas definiciones de especies tales genogrupos pueden corresponder a poblaciones alopátricas dentro de una misma especie[53] en lugar de ser especies distintas resultantes de una especiación reciente con poco tiempo para la diferenciación fenotípica, o una especiación con conservatismo de nicho.[39,86] Es necesario un muestreo geográfico exhaustivo antes de poder confrontar estas hipótesis con seguridad y entender mejor la naturaleza de estas especies en *Escallonia.*

En todas las "especies genéticas crípticas" que identificamos, los fenogrupos no muestran una fuerte estructura geográfica (por ejemplo, véase el genogrupo 10, los fenogrupos $2, 3, 5, 7$, el clado V; Fig. 1). Esto es consistente con la intrigante posibilidad de que estas especies, que pese a ser fenotípicamente distintas, podrían estar potencialmente interconectadas a través del intercambio de genes,[40,41] probablemente facilitado por su amplio solapamiento en el espacio geográfico.[55] Todavía no se sabe si este patrón refleja especiación con flujo génico o si el flujo génico ocurre después del contacto secundario. Nuestro muestreo actual en *Escallonia* no está diseñado para desentrañar estas posibilidades y se requieren más análisis. Sin embargo, observamos que la evidencia genómica para este tipo de especies se está acumulando rápidamente para otras plantas[87–89] así como para varios taxones a través del árbol de la vida.[52,90] En otros grupos taxonómicos, este tipo de especies incluye tanto especies de divergencia reciente, que posiblemente se diferencian con flujo génico, como especies con más de 10-20 millones de años de divergencia con el subsiguiente flujo génico que se produce tras el contacto secundario.[92] Sin embargo, es necesario estudiar con más detalle cómo se inician y persisten estos grupos de especies, y qué parte de sus genomas se intercambia libremente a través de los límites de las especies sin que éstas colapsen.[93] Además, argumentamos que el enfoque de descubrimiento de especies que empleamos aquí, en el que tanto el fenotipo como el genotipo contribuyen por igual y de forma independiente al patrón de las especies, es esencial para detectar este tipo de grupos de especies donde de otro modo son inaccesibles. *Escallonia* constituye un excelente caso de estudio para abordar estas preguntas críticas en biología, aunque es necesario realizar más muestreos genómicos, fenómicos y geográficos.

Alternativamente, estas "especies genéticas crípticas" pueden ser el resultado de eventos de divergencia rápida impulsados por factores que influyen en los rasgos relevantes para el aislamiento ecológico con poco tiempo para que los alelos se separen completamente entre las especies hermanas.[94] Dado que varios fenogrupos dentro de un genogrupo a veces coocurren en simpatría de mosaico[58] o se sustituyen entre sí a lo largo de la elevación[55] (Resultados suplementarios), es plausible que la rápida divergencia en *Escallonia* haya sido impulsada por nuevas oportunidades ecológicas debidas a los ciclos climáticos y a la orogenia de las montañas.[95] La falta de estudios experimentales sobre la ecología funcional de los rasgos foliares y florales en *Escallonia* nos impide saber qué factores son los responsables de mantener la divergencia fenotípica mostrada por diferentes fenogrupos dentro de un mismo genogrupo. Algunos fenogrupos pueden diferir en rasgos florales que podrían tener relación con los polinizadores. Otros fenogrupos pueden variar más fuertemente en rasgos foliares que podrían estar relacionados con la adaptación a los entornos locales. Por lo tanto, es plausible que diferentes formas de selección mantengan las diferencias fenotípicas y contrarresten los efectos homogeneizadores del flujo génico en las especies nacientes, una posibilidad que requiere más investigación. Por lo tanto, se requieren más muestreos de taxones y genomas en combinación con modelos genómicos poblacionales explícitos que incorporen diferentes formas de selección en *Escallonia* para aislar la señal de la separación incompleta de linajes de la hibridación[96] y modelar el papel de la selección entre especies hermanas y no hermanas en el contacto secundario.

Conclusión

En resumen, nuestros análisis de un conjunto de datos fenotípicos y genómicos a gran escala, utilizando enfoques basados en modelos de última generación para el descubrimiento y la delimitación de especies, revelan que las especies vegetales existen en *Escallonia* como una propiedad de la naturaleza independiente de la taxonomía.[48,72] Sin embargo, el patrón observado de discordancia excesiva entre las especies identificadas con datos fenotípicos y genómicos sugiere que, en ausencia de datos y pruebas explícitas, la suposición prevalente que las entidades fenotípicamente (o genéticamente) distintas son necesariamente "buenas especies" no está justificada. Además, la señal paralela de tal discordancia a través de clados divergentes en *Escallonia* sugiere que esto puede ser un fenómeno generalizado, lo cual es consistente con los patrones emergentes sobre la naturaleza de las especies a través del árbol de la vida.[52,74,88–90,92] El enfoque de descubrimiento de especies que utilizamos aquí, el cual considera explícitamente tanto los datos fenotípicos como los genéticos en igualdad de condiciones, es esencial para revelar patrones útiles para guiar nuestra inferencia de los probables procesos evolutivos en juego en la especiación. Estudios anteriores han propuesto que aproximadamente el 70% de las especies taxonómicas de plantas representan "especies buenas",[44] pero esto nuestro estudio no apoya tal resultado. Por el contrario, nuestros resultados sugieren que el porcentaje de especies taxonómicas de *Escallonia* que corresponden a "especies buenas" puede ser tan bajo como el 17%$ (Tabla 4, Tabla Suplementaria S4, S7, S10, S13, S16, S19). Dado que *Escallonia* parece ser un género "típico" de plantas que no se considera único o problemático desde el punto de vista taxonómico (véase la Introducción), este resultado es notable. No conocemos conjuntos de datos de magnitud similar para otros grupos de plantas, pero especulamos que nuestros resultados pueden ser generalizados. En la medida en que nuestros resultados captan cualquier perspectiva generalizable sobre la naturaleza de las especies vegetales, reforzada por la pobre base teórica que subyace la delimitación de las especies vegetales en general,[68,69] nuestros resultados sugieren que los estudios en otras áreas de la biología que asumen que las especies taxonómicas representan entidades buenas y biológicamente reales pueden necesitar una re-evaluación crítica. Nuestros resultados subrayan la necesidad de realizar más estudios comparativos que combinen datos fenotípicos y genotípicos en otros taxones y a través de escalas espaciales amplias y estrechas para comprender de forma exhaustiva la naturaleza de las especies vegetales y así arrojar luz sobre las fuerzas evolutivas que actúan en la especiación y en el mantenimiento de las especies en la naturaleza.[48] Dados los avances sin precedentes en fenómica, genómica y computación, nunca ha habido un momento más próspero para ser taxónomo que ahora.

Table 4: **Correspondencia entre especies taxonomicas y los mejores fenogrupos y genogrupos.**

Clade	Taxonomic species	Phenogroups	Perfect match taxonomic species to phenogroups	Genogroups	Perfect match taxonomic species to genogroups	Perfect match taxonomic species to phenogroup and genogroup
I	2	2	2	3	1	1
II	2	3	0	4	1	0
III	6	5	1	7	3	1
IV	2	2	2	6	1	1
V	7	8	0	10	0	0
VI	10	8	2	11	5	2

B Appendix B: Analysis Session Information

These are the packages used to run all analyses and generate both the manuscript and supplementary material files:

```
## R version 4.1.1 (2021-08-10)
## Platform: x86_64-apple-darwin17.0 (64-bit)
## Running under: macOS Big Sur 10.16
##
## Matrix products: default
## BLAS:   /Library/Frameworks/R.framework/Versions/4.1/Resources/lib/libRblas.0.dylib
## LAPACK: /Library/Frameworks/R.framework/Versions/4.1/Resources/lib/libRlapack.dylib
##
## locale:
## [1] en_US.UTF-8/en_US.UTF-8/en_US.UTF-8/C/en_US.UTF-8/en_US.UTF-8
##
## attached base packages:
## [1] stats     graphics  grDevices utils     datasets  methods   base
##
## other attached packages:
##  [1] eulerr_6.1.1        gridExtra_2.3       GoodmanKruskal_0.0.3
##  [4] ggstance_0.3.5      RColorBrewer_1.1-2  phytools_0.7-90
##  [7] maps_3.4.0          ape_5.5             treeio_1.16.2
## [10] ggtree_3.0.4        kableExtra_1.3.4    reshape_0.8.8
## [13] knitr_1.36          patchwork_1.1.1     forcats_0.5.1
## [16] stringr_1.4.0       dplyr_1.0.7         purrr_0.3.4
## [19] readr_2.0.2         tidyr_1.1.4         tibble_3.1.5
## [22] ggplot2_3.3.5       tidyverse_1.3.1     float_0.2-6
##
## loaded via a namespace (and not attached):
##  [1] colorspace_2.0-2       ellipsis_0.3.2         fs_1.5.0
##  [4] aplot_0.1.1            rstudioapi_0.13        farver_2.1.0
##  [7] bit64_4.0.5            fansi_0.5.0            lubridate_1.8.0
## [10] xml2_1.3.2             codetools_0.2-18       mnormt_2.0.2
## [13] polyclip_1.10-0        jsonlite_1.7.2         broom_0.7.9
## [16] dbplyr_2.1.1           compiler_4.1.1         httr_1.4.2
## [19] backports_1.2.1        assertthat_0.2.1       Matrix_1.3-4
## [22] fastmap_1.1.0          lazyeval_0.2.2         cli_3.0.1
## [25] htmltools_0.5.2        tools_4.1.1            igraph_1.2.6
## [28] coda_0.19-4            gtable_0.3.0           glue_1.4.2
## [31] reshape2_1.4.4         clusterGeneration_1.3.7 fastmatch_1.1-3
## [34] Rcpp_1.0.7             cellranger_1.1.0       vctrs_0.3.8
## [37] svglite_2.0.0          nlme_3.1-153           polylabelr_0.2.0
## [40] xfun_0.26              rvest_1.0.1            lifecycle_1.0.1
## [43] phangorn_2.7.1         MASS_7.3-54            scales_1.1.1
## [46] vroom_1.5.5            hms_1.1.1              parallel_4.1.1
## [49] expm_0.999-6           yaml_2.2.1             ggfun_0.0.4
## [52] yulab.utils_0.0.2      stringi_1.7.5          plotrix_3.8-2
## [55] tidytree_0.3.5         rlang_0.4.11           pkgconfig_2.0.3
## [58] systemfonts_1.0.2      evaluate_0.14          lattice_0.20-45
```

```
## [61] labeling_0.4.2        cowplot_1.1.1          bit_4.0.4
## [64] tidyselect_1.1.1      plyr_1.8.6             magrittr_2.0.1
## [67] bookdown_0.24         R6_2.5.1               generics_0.1.0
## [70] combinat_0.0-8        DBI_1.1.1              pillar_1.6.3
## [73] haven_2.4.3           withr_2.4.2            scatterplot3d_0.3-41
## [76] modelr_0.1.8          crayon_1.4.1           utf8_1.2.2
## [79] tmvnsim_1.0-2         tzdb_0.1.2             rmarkdown_2.11
## [82] grid_4.1.1            readxl_1.3.1           reprex_2.0.1
## [85] digest_0.6.28         webshot_0.5.2          numDeriv_2016.8-1.1
## [88] gridGraphics_0.5-1    munsell_0.5.0          viridisLite_0.4.0
## [91] ggplotify_0.1.0       quadprog_1.5-8
```
